# Uranium(III) and Uranium(IV) *meta*-Terphenylthiolate Complexes

**DOI:** 10.1021/acs.inorgchem.4c03085

**Published:** 2025-02-07

**Authors:** Benjamin L. L. Réant, John A. Seed, George F. S. Whitehead, Conrad A. P. Goodwin

**Affiliations:** † Centre for Radiochemistry Research, The University of Manchester, Oxford Road, Manchester M13 9PL, U.K.; ‡ Department of Chemistry, The University of Manchester, Oxford Road, Manchester M13 9PL, U.K.

## Abstract

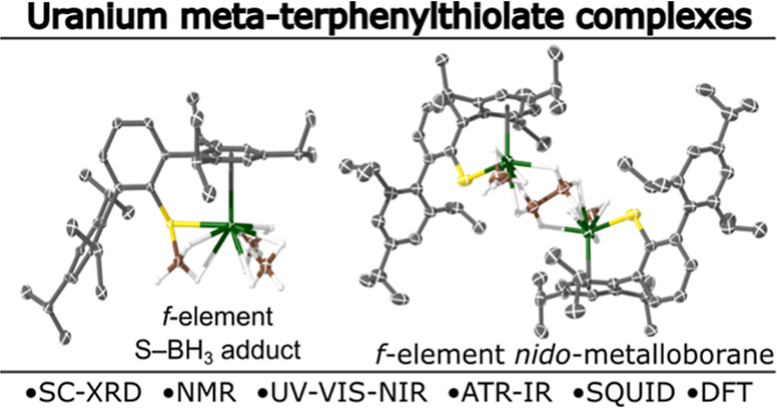

We report the synthesis and characterization of crystalline *m*-terphenylthiolate uranium complexes supported by the bulky ligand system, SAr^*i*Pr6^ (SAr^*i*Pr6^ = {SC_6_H_3_-2,6-(Tripp)_2_}; Tripp = 2,4,6-*i*Pr-C_6_H_2_). Treatment of U^IV^Cl_4_ with 2 equiv of KSAr^*i*Pr6^ in Et_2_O afforded both [U^IV^(SAr^*i*Pr6^)_2_(Cl)_2_] (**1**) and the Et_2_O adduct, [U^IV^(SAr^*i*Pr6^)_2_(Cl)_2_(Et_2_O)_2_] (**1·Et**_**2**_**O**) in poor yield. The reaction between [U^IV^(BH_4_)_4_] and 1 equiv of KSAr^*i*Pr6^ in toluene gave several crystals of the double salt, [U^IV^(μ-SAr^*i*Pr6^)(BH_4_)_2_(μ-BH_4_)(μ^3^-BH_4_)K]_2_ (**2**), and exposing the crude reaction mixture to Et_2_O gave the disulfide dimer, (SAr^*i*Pr6^)_2_. The reaction between [U^IV^(BH_4_)_4_] and 1 equiv of HSAr^*i*Pr6^ in hot toluene gave [U^III^(H_3_B·SAr^*i*Pr6^ κ*S*,*H*,*H*)(BH_4_)_2_] (**3**) which proved resistant to further substitution using either HSAr^*i*Pr6^ or KSAr^*i*Pr6^. Two U(III) *mono*-terphenylthiolates, [U^III^(SAr^*i*Pr6^)(BH_4_)_2_] (**4a**) and [{U^III^(SAr^*i*Pr6^)(BH_4_)}_2_{μ-B_2_H_6_}] (**4b**), were isolated as a mixture from the reaction between [U^III^(BH_3_)_3_(toluene)] and 1 equiv of KSAr^*i*Pr6^, while using 2 equiv of KSAr^*i*Pr6^ gave the *bis*-terphenylthiolate complex [U^III^(SAr^*i*Pr6^)_2_(BH_4_)] (**5**). Complex **4b** is a rare example of a *nido*-metalloborane. Complexes **1**–**5** have been characterized variously by single-crystal and powder X-ray diffraction, multinuclear NMR spectroscopy, infrared spectroscopy, UV–Vis–NIR spectroscopy, SQUID magnetometry, and elemental analyses as appropriate. Quantum chemical calculations have been employed to interpret the nature of the U–S bonding interactions across these complexes.

## Abstract 2

We report the first examples of *m*-terphenylthiolate complexes of uranium which highlight the supporting role of U···arene interactions in the isolation of heteroleptic complexes with this ligand set. We explore protonolysis and salt elimination strategies with uranium borohydride precursors showcasing diverse, and sometimes unexpected, chemistry affording intriguing structures of uranium including a rare S-BH_3_ ligand adduct and the first f-element *nido*-metalloborane complex.

## Introduction

**Figure 1:**
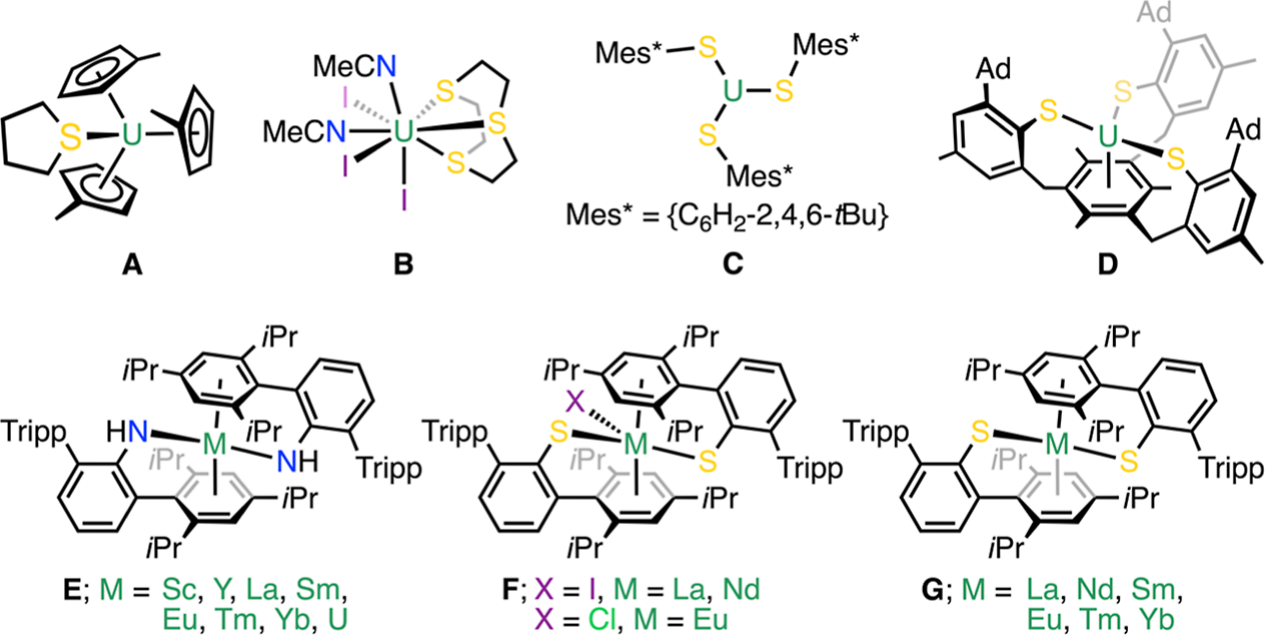
(A–D) Representative examples of U(III) complexes featuring U–S linkages with both dative and anionic donors; (E) previously reported [M^II^(NHAr^*i*Pr6^)_2_] complexes to highlight the binding mode of the EAr^*i*Pr6^ framework (E = N(H) here); (F,G) heteroleptic Ln(III) arylthiolate complexes along with Ln(II) congeners.

The coordination chemistry of uranium (U) with first-row donor ligands such as amides and alkoxides (and their aryl congeners) is a mature area.^1−7^ The favorable hard donor to hard metal acid–base match with these (N and O) first-row elements means that such complexes have largely ionic metal–ligand bonding and can be found in a wide range of oxidation states from U(II) to U(VI).^7−9^ In contrast, the structure, bonding, and reactivity of uranium complexes supported by ligands using heavier second-row donors such as S-donors in (aryl)thiolates are less well developed across all uranium oxidation states.^10,11^ To date, there are just a handful of crystallographically authenticated U^III^–S bonds using either anionic or neutral S-donor ligands (see Figure 1 for complexes **A** to **D** which are representative examples).^12−18^ The small extent of U^III^–S donor chemistry reflects the paucity of U(III) coordination chemistry in general as compared to that of higher oxidation states (IV to VI).^7−9^ Furthermore, the poorer acid–base match using softer donors, such as organo-sulfur anions, results in metal–ligand linkages that are more labile than first-row analogues and thus oftentimes require mild synthetic strategies to install such as protonolysis^17−19^ and σ-bond metathesis,^15,20,21^ though salt elimination routes are known as well.^17,22−24^ The reaction between lower oxidation state U-precursors and mild oxidants such as disulfides is attractive for U–S bond formation but is not suitable for the isolation of U(III) complexes.^25−31^ Comparisons between isostructural rare earth (RE) and U(III) complexes with similarly sized metal ions often show the U–S bond to be shorter than the corresponding RE–S bond by an amount larger than the difference in their ionic radii, which has been interpreted as a sign of increased covalent character;^14,16,17,19,32^ however, paucity of U^III^–S linkages at present means that such studies are rare and represent an attractive synthetic target.

Recent works have shown that Power’s *m*-terphenyl anillide ligand, {NHAr^*i*Pr6^} (Ar^*i*Pr6^ = {C_6_H_3_-2,6-Tripp_2_}; Tripp = 2,4,6-*i*Pr-C_6_H_2_)^33^ supports trivalent and formally divalent RE and uranium complexes of the general form [M(NHAr^*i*Pr6^)_2_(X)_*n*_] (*n* = 0 or 1, X = Cl, I; M = U, Sc, Y, La, Sm, Eu, Tm, Yb), where metal-arene close contacts are rigorously enforced (see Figure 1 complex **E** for divalent examples).^34−36^ For example, some of us showed the Yb(II)···arene interaction in diamagnetic [Yb^II^(NHAr^*i*Pr6^)_2_] is maintained in solution at room temperature whereby the “bound” and “terminal” Tripp groups do not exchange.^34^ Inspired by Meyer’s work on an arene-anchored *tris*-arylthiolate complex that is structurally analogous to an earlier aryloxide variant,^18,37^ we envisaged that substitution of the N(H)-donor in the NHAr^*i*Pr6^ ligand for an S atom (i.e., to give {SAr^*i*Pr6^}) would allow us to assemble uranium terphenyl thiolate complexes supported by U···arene contacts and permit an exploration of both the U–S and U···arene bonding interactions. Toward this goal, Niemeyer and Evans have shown the SAr^*i*Pr6^ ligand supports lanthanide complexes with Ln···arene interactions in the solid state (Figure 1, see **F** and **G**).^38−40^

Here we report the isolation of *mono*- and *bis*-SAr^*i*Pr6^ U(IV) and U(III) complexes using a combination of salt elimination, protonolysis, and thermolysis routes. The U(IV) precursors U^IV^Cl_4_ and [U^IV^(BH_4_)_4_] were both poorly suited to synthesizing U(IV) complexes. For example, salt elimination between 2 equiv of KSAr^*i*Pr6^ and U^IV^Cl_4_ gave a mixture of products, [U^IV^(SAr^*i*Pr6^)_2_(Cl)_2_] (**1**) and [U^IV^(SAr^*i*Pr6^)_2_(Cl)_2_(Et_2_O)_2_] (**1·Et**_**2**_**O**), in low yield; while using [U^IV^(BH_4_)_4_] instead gave the double-salt [U^IV^(μ-SAr^*i*Pr6^)(BH_4_)_2_(μ-BH_4_)(μ^3^-BH_4_)K]_2_ (**2**) under similar conditions. Protonolysis between [U^IV^(BH_4_)_4_] and HSAr^*i*Pr6^ at elevated temperatures resulted in reduction to give [U^III^(H_3_B·SAr^*i*Pr6^-κ*S*,*H*,*H*)(BH_4_)_2_] (**3**) which may be synthesized free of the BH_3_ adduct through salt elimination between [U^III^(BH_4_)_3_(toluene)] and KSAr^*i*Pr6^ to give [U^III^(SAr^*i*Pr6^)(BH_4_)_2_] (**4a**). Complex **4a** undergoes dehydrocoupling to give [{U^III^(SAr^*i*Pr6^)(BH_4_)}_2_(μ-B_2_H_6_)] (**4b**), a rare f-element *nido*-metalloborane, as such **4a** and **4b** could not be isolated separately in pure form. Finally, [U^III^(SAr^*i*Pr6^)_2_(BH_4_)] (**5**) was isolated in good yield from the salt elimination reaction between 2 equiv of KSAr^*i*Pr6^ and [U^III^(BH_4_)_3_(toluene)].

Complexes **3**–**5** highlight the diverse supporting role that borohydrides have in f-element coordination chemistry. All complexes herein have been characterized variously by single-crystal X-ray diffraction, multinuclear NMR, ATR–IR, and UV–Vis–NIR spectroscopies, SQUID magnetometry, and elemental analyses as appropriate. Quantum chemical calculations have been used to examine the U–S and U···arene interactions, revealing polar-covalent U–S interactions in each case. No U···arene δ-bonds were found in the occupied frontier orbitals of any of the complexes, which we attribute to the unusually long U···C_arene_ distances in these structures.

## Results and Discussion

### Synthesis

**Scheme 1:**
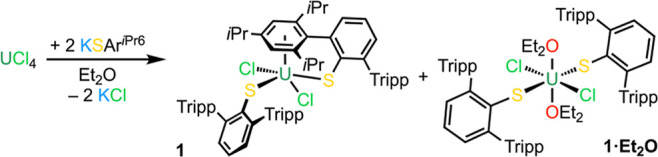
Synthesis of [U^IV^(SAr^*i*Pr6^)_2_(Cl)_2_] (**1**) from U^IV^Cl_4_ and 2 equiv of KSAr^*i*Pr6^ in Et_2_O. Several crystals of [U^IV^(SAr^*i*Pr6^)_2_(Cl)_2_(OEt_2_)_2_] (**1·Et**_**2**_**O**) were isolated on one attempt. Tripp = {C_6_H_2_-2,4,6-*i*Pr_3_}.

We sought to isolate examples of both U(IV) and U(III) arylthiolate complexes to compare the U–S bonding between the two oxidation states. Stirring 2 equiv of KSAr^*i*Pr6^ with U^IV^Cl_4_ in Et_2_O led to the precipitation of a red powder and gave a pale amber solution. Workup and crystallization of the red powder from hot *n*-hexane gave [U^IV^(SAr^*i*Pr6^)_2_(Cl)_2_] (**1**) in low crystalline yield (20%; Scheme 1) which gave satisfactory results by elemental microanalysis. On one attempt, several crystals of [U^IV^(SAr^*i*Pr6^)_2_(Cl)_2_(OEt_2_)_2_] (**1·Et**_**2**_**O**) were isolated as a co-crystallized mixture with unreacted KSAr^*i*Pr6^. Analysis of the amber solution showed the major component in solution to be unreacted KSAr^*i*Pr6^, but attempts to optimize the yield of either **1** or **1·Et**_**2**_**O** were unsuccessful.

**Scheme 2:**
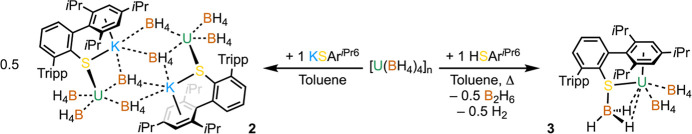
Left: The reaction between [U^IV^(BH_4_)_4_] and 1 equiv of KSAr^*i*Pr6^ in toluene to give the double salt [U^IV^(μ-SAr^*i*Pr6^)(BH_4_)_2_(μ-BH_4_)(μ^3^-BH_4_)K]_2_ (**2**); Right: The reaction between [U^IV^(BH_4_)_4_] and 1 equiv of HSAr^*i*Pr6^ in toluene affords [U^III^(H_3_B·SAr^*i*Pr6^-κ*S*,*H*,*H*)(BH_4_)_2_] (**3**).

The low yield of **1** and the routine presence of unreacted KSAr^*i*Pr6^ from reactions involving U^IV^Cl_4_ prompted us to explore an alternative U(IV) precursor. As [U^IV^(BH_4_)_4_]_*n*_ can be synthesized directly from U^IV^Cl_4_, it seemed a logical choice, and the {BH_4_} anion has been shown to be an excellent leaving group in f-element chemistry.^16,41−48^ The reaction between [U^IV^(BH_4_)_4_]_*n*_ and 1 equiv of KSAr^*i*Pr6^ in toluene gave a single crop of the double salt [U^IV^(μ-SAr^*i*Pr6^)(BH_4_)_2_(μ-BH_4_)(μ^3^-BH_4_)K]_2_ (**2**) as red crystals from hot toluene in very poor crystalline yield as the solvent cooled to room temperature (3% isolated crystalline yield; Scheme 2). The ATR–IR spectrum shows multiple poorly resolved features between ν = 2500 and 2000 cm^–1^, implying a range of binding modes for the {BH_4_} groups, and the spectrum of crude material formed in near-quantitative yield is superposable with that of crystalline **2** which suggests the low isolated crystalline yield is due to low solubility.^49^ An attempt to extract crude **2** into Et_2_O (instead of toluene) gave the sulfide-bridged dimer (SAr^*i*Pr6^)_2_ as the only isolable crystalline product which we have crystallographically characterized for the first time (see the Supporting Information) and which is similar to previously reported Se and Te analogues.^50^ As Et_2_O was transferred directly from a K-mirror, we suggest the U(IV) in the reaction mixture acts as an oxidant in this process rather than there being extraneous oxidants such as peroxides from the solvent.

As elimination of KBH_4_ proved unfavorable in **2**, protonolysis was explored as an alternative route to give U(IV) arylthiolate complexes through the reaction of [U^IV^(BH_4_)_4_]_*n*_ with 1 equiv of HSAr^*i*Pr6^ in hot toluene (Scheme 2). After workup, the brown reaction mixture gave red–green crystals of [U^III^(H_3_B·SAr^*i*Pr6^ κ*S*,*H*,*H*)(BH_4_)_2_] (**3**) in good yield (69%). Complex **3** is the net product of thermolytic reduction of [U^IV^(BH_4_)_4_]_*n*_, deprotonation of HSAr^*i*Pr6^, and capture of the by-product BH_3_ by the S-atom lone pair. The thermolytic reduction of U(IV) to U(III) under the conditions described is not unusual as careful thermolysis of [U^IV^(BH_4_)_4_]_*n*_ gives U(III)-borohydrides.^51^ Doublets at ν = 2467 and 2152 cm^–1^ in the ATR–IR spectrum of **3** are attributed to stretching modes from B–H_t_ (A_1_) {κ^2^-H_3_B·SAr^*i*Pr6^} and B–H_b_ (A_1_ and E) from {κ^3^-BH_4_}, respectively.^49^ Due to difficulty in obtaining a satisfactory elemental analysis of **3**, we examined the isolated crystalline material using powder X-ray diffraction (PXRD) which revealed the presence of an additional crystalline phase. While this phase may be attributed to a polymorph of **3**, we cannot discount the potential presence of an additional complex that was not observed in SC-XRD studies (see the Supporting Information).

**Scheme 3:**
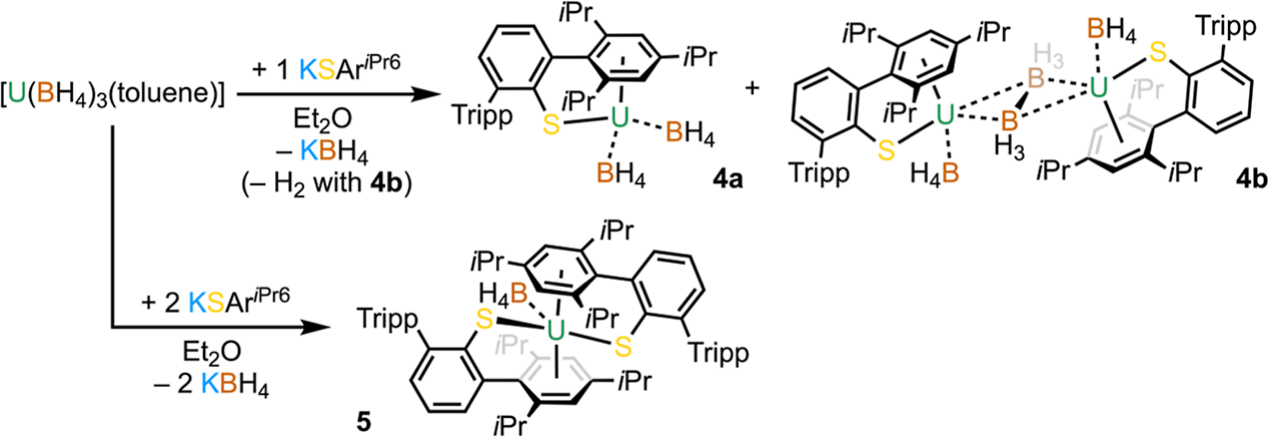
Synthesis of [U^III^(SAr^*i*Pr6^)(BH_4_)_2_] (**4a**) and [{U^III^(SAr^*i*Pr6^)(BH_4_)}_2_(μ-B_2_H_6_)] (**4b**) from [U^III^(BH_4_)_3_(Toluene)] and 1 equiv of KSAr^*i*Pr6^ in Et_2_O and [U^III^(SAr^*i*Pr6^)_2_(BH_4_)] (**5**) from [U^III^(BH_4_)_3_(Toluene)] and 2 equiv of KSAr^*i*Pr6^.

For the isolation of further U(III) complexes, we opted to use salt elimination strategies with a preformed U(III) precursor as this would allow an analogue of **3** to be isolated without the bound BH_3_ moiety. [U^IV^(BH_4_)_4_]_*n*_ was thermolyzed in toluene and dried under vacuum to give [U^III^(BH_4_)_3_(toluene)] as a red microcrystalline powder which was used in situ without further purification.^48,51^ Addition of Et_2_O to a precooled (−98 °C) mixture of [U^III^(BH_4_)_3_(toluene)] and 1 equiv of KSAr^*i*Pr6^ followed by workup and crystallization from *n*-hexane gave dark-red crystals of [U^III^(SAr^*i*Pr6^)(BH_4_)_2_] (**4a**). A second crystalline crop was isolated from *n*-hexane after trituration with hot toluene and was found to be dimeric [{U^III^(SAr^*i*Pr6^)(BH_4_)}_2_(μ-B_2_H_6_)] (**4b**)—see Scheme 3. The combined yield was fair (37%), and the ATR–IR spectra from each crop were almost perfectly superposable (see the Supporting Information), suggesting that both batches contain a mixture of **4a** and **4b** in roughly equal proportions. These spectra show a strong singlet at ν = 2472 cm^–1^ for the {κ^3^-BH_4_} B–H_t_ A_1_ stretching mode, which presents with poorly resolved doublet character (Δ ca. 25 cm^–1^) in some batches, suggesting the presence of a {κ^2^-BH_4_} coordination mode. A doublet at 2154 cm^–1^ (Δ ca. 60 cm^–1^) likely arises from the A_1_ and E bridging B–H_b_ stretching modes of the {κ^3^-BH_4_} groups.^49^ These first two sets of peaks are essentially identical to those found in **3**, which aided the assignment. Notably, the spectra for **4a**/**4b** have an additional feature at ν = 2281 cm^–1^ (singlet) which is absent in **3** and is similar to other *nido*-metalloborane complexes with reported IR data^52−56^ and which we tentatively assign to the κ^2^-BH_3_ stretching mode of the {μ-B_2_H_6_}^2–^ moiety.

With a range of monoarylthiolate complexes in hand which demonstrate the ability of this ligand set to support both U(III) and U(IV)–arene interactions, the synthesis of [U^III^(SAr^*i*Pr6^)_2_(BH_4_)] (**5**) was attempted by reacting 2 equiv of KSAr^*i*Pr6^ with [U^III^(BH_4_)_3_(toluene)] in Et_2_O (Scheme 3). Crystallization from hot hexane gave **5** in a modest yield (52%). The ATR–IR spectrum of microcrystalline **5** shows a distorted doublet (ν = 2480 cm^–1^, A_1_ and B_1_) and a singlet (ν = 2134 cm^–1^, A_1_ and B_2_) for the B–H stretching modes, commensurate with a {κ^2^-BH_4_} group.

### NMR Spectroscopy

The ^1^H NMR spectrum of **1** in *d*_6_-benzene shows broad resonances (full width and half-maximum, , given where appropriate) at δ_H_ = 16.85 ( = 195 Hz, *i*Pr C*H*_3_) and −1.00 ( = 133 Hz, *i*Pr C*H*), along with a sharper singlet at 10.60 ppm (Tripp C*H*), which were tentatively assigned by their relative integrals. The SAr 3,4,5-C*H* groups could not be conclusively located. The *mono*-terphenylthiolate complex **2** shows seven broad peaks spanning δ_H_ = 14.46 to −4.54, commensurate with *C*_2_ symmetry in the *d*_6_-benzene solution, which suggests that both Tripp groups are equivalent on the NMR time scale.

We could not definitively assign the ^1^H NMR spectra of U(III) **3**, **4a**/**4b**. In the case of **3**, we observe a large number of peaks that suggest the complex has low symmetry in solution, presumably because the metal-bound and terminal Tripp groups do not exchange on the NMR time scale.^34^ The ^1^H NMR spectrum of **5** in *d*_6_-benzene displayed broadened resonances between δ_H_ = 8 to −1 for the {SAr^*i*Pr6^} ligand which integrate well for a *C*_2_ symmetric structure in solution whereby the metal-bound and terminal Tripp groups exchange on the NMR time scale, though definitive assignment was not possible. The {BH_4_} group appears at δ_H_ = 126.58 in the ^1^H NMR spectrum, which is shifted from both [U^IV^(BH_4_)_4_] (δ_H_ = 130.86) and [U^III^(BH_4_)_3_(THF)_2_] (δ_H_ = 118.58).

The ^11^B NMR spectrum of U(IV) complex **2** showed a single resonance at δ_B_ = 141.3 ( = 506 Hz), which is similar to that of [U^IV^(BH_4_)_4_]_*n*_ (δ_B_ = 131.6). Due to similarities in their spectra, U(III) complexes **3** and **4a**/**4b** are discussed together. In the case of **3**, the ^11^B NMR spectrum in *d*_6_-benzene revealed three broad singlets (δ_B_ = 102.9, 75.2, 59.0;  = 217, 473, and 242 Hz respectively), two of which are almost identical to features in the spectrum of **4a**/**4b** (δ_B_ = 102.9, 75.4;  = 278, 430 Hz, respectively) and which we tentatively assign to the terminal {κ^3^-*B*H_4_} and bridging {μ-*B*_2_H_6_}^2–^ groups. The resonance at 59.0 ppm in the ^11^B NMR spectrum of **3** is thus assigned to the {κ^2^-H_3_*B*·SAr^*i*Pr6^} ligand, which can also be observed as a weak signal in the spectrum of **4a**/**4b**. Similarly, the presence of peaks at ca. δ_B_ = 75 and 103 in the spectra of all three complexes means that {μ-B_2_H_6_}^2–^ groups are present in all three samples to varying degrees. Toward this argument, a small H_2_ resonance (δ_H_ = 4.47) was observed in the ^1^H NMR spectrum of **3**, which suggests complex **3** may slowly undergo a dehydrocoupling reaction in solution to produce an unidentified complex.^58−60^ We have discounted the presence of the residual U(III) precursor as the ^11^B NMR spectrum of [U^III^(*B*H_4_)_3_(THF)_2_] shows a broad singlet at a significantly different chemical shift (*d*_6_-benzene, δ_B_ = 153;^48^*d*_8_-THF, δ_B_ = 230)^47^ to any of these features. Attempts to monitor the thermal conversion of **4a** to **4b** by ^1^H and ^11^B NMR spectroscopy were inconclusive (see the Supporting Information). For complex **5**, a broad resonance is observed at δ_B_ = 177.8 ( = 344 Hz) which corresponds to the {κ^2^-*B*H_4_} group.

### Molecular Structures

**Figure 2:**
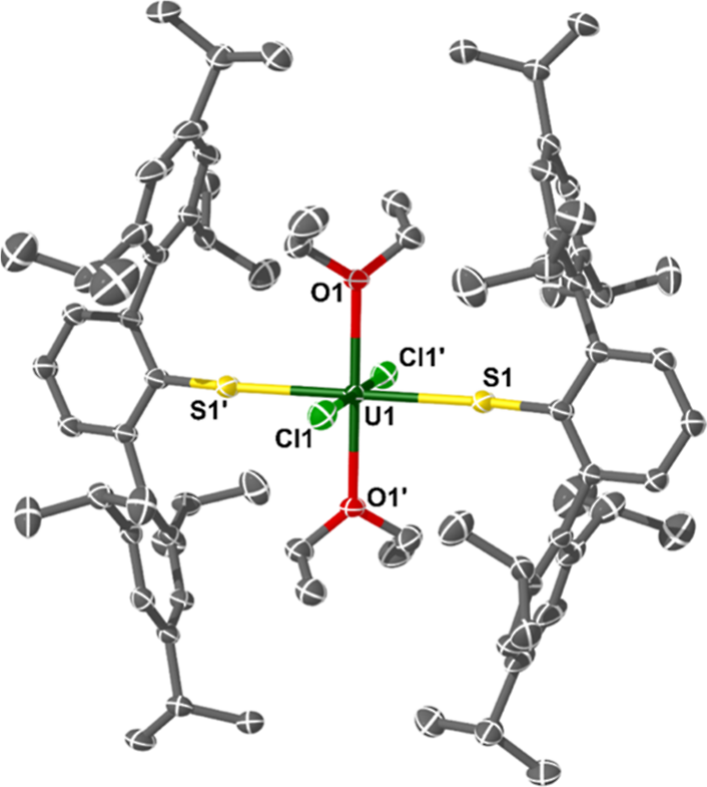
Molecular structure of complex **1·Et**_**2**_**O**. Ellipsoids set at a 50% probability and H-atoms removed for clarity (operations: X, Y, Z; 1–X, 1–Y, 1–Z). Selected bond lengths and angles: U1–S1 = 2.6526(9) Å, U1–Cl1 = 2.5699(10) Å, U1–O1 = 2.368(2) Å, and U1–S1–C_ipso_ = 158.71(14)°.

The molecular structures of complexes **1**, **1·Et**_**2**_**O**, and **2**–**5** were determined by single-crystal X-ray diffraction (SC-XRD) studies, and selected bond metrics are given in Table 1. Complexes **1** and **1·Et**_**2**_**O**, crystallized from hexane and toluene, respectively, in the P1̅ space group (see Figure 2 for the molecular structure of **1·Et**_**2**_**O** and the Supporting Information for **1**). In the case of **1**, the resolution of the data was limited due to weak diffraction at higher angles due to the large unit cell (*V* = 50,235 Å^3^; *Z*′ = 10), though the connectivity is unambiguous and supported by further characterization data. The geometry is best described as *pseudo*-sawhorse with respect to the U, Cl, and S-atoms, while the U-center is capped by a single η^6^-Tripp group in each independent molecule (range U···η^6^-Tripp = 2.588(5)–2.594(5) Å). On the other hand, complex **1·Et**_**2**_**O**, is octahedral (SHAPE analysis = 0.306)^61^ with an inversion center at the U atom. The U–Cl (2.5699(10) Å) distance in **1·Et**_**2**_**O** is significantly longer than the range in **1** (2.524(5)–2.546(4) Å), despite the higher formal coordination number of the latter. When comparing the U–S bond lengths, there are two distinct ligand binding modes in **1**. Where the ligand is bound only to the S atom, the range of U–S distances (2.647(5)–2.670(5) Å) overlaps that of **1·Et**_**2**_**O** (2.6526(9) Å). The second ligand in **1** features an additional U···η^6^-Tripp interaction which results in a significant lengthening of the U–S distances (range 2.682(4)–2.702(5) Å). In all cases with **1** and **1·Et**_**2**_**O**, the U–S distances are somewhat shorter than the sum of the covalent radii for a U–S single bond (2.73 Å)^62^ and are in the middle of the range of terminally bound organo-sulfur U(IV) distances reported in the CCDC (2.61(5) to 3.029(4) Å).^63−65^

**Table 1:** Bond lengths (Å) and angles (deg) for complexes **1**, **1·Et**_**2**_**O**, **2**, **3**, **4a**, **4b**, and **5**.

(Å or deg)	**1**^a^	**1·Et**_**2**_**O**	**2**	**3**	**4a**	**4b**^c^	**5**
U–S	2.647(5)–2.702(5)	2.6526(9)	2.6948(10)	2.8824(9)	2.687(4)	2.721(3)	2.7888(8), 2.7969(7)
U···B			2.489(6)–2.865(7)	2.584(6), 2.603(7)	2.56(2), 2.56(3)	2.556(8), 2.609(6)	2.872(4)
U–TrippC_6_				2.883(4)–2.920(4)	2.815(16)–2.918(17)	2.867(5)–2.899(5)	3.030(3)–3.152(3)
U···η^6^-Tripp_cent_	2.588(5)–2.594(5)			2.5379(15)	2.482(7)	2.5168(19)	2.744(2), 2.747(2)
U–S–C_ipso_	114.8(5)–116.0(5), 122.6(6)–124.3(6)^b^	158.71(14)	124.88(14)	109.54(12)	113.6(5)	111.7(4)	118.53(11)

a Ten crystallographically independent molecules exist in the asymmetric unit, so a range is presented for each metric.

b The structure has one ligand with a U···η^6^-Tripp contact and a second ligand without this contact, the range given for each type separately.

c The {SAr^*i*Pr6^} unit is disordered over two positions, which refined to a ratio of 82:18; only metrics for the highest occupancy unit are given.

**Figure 3:**
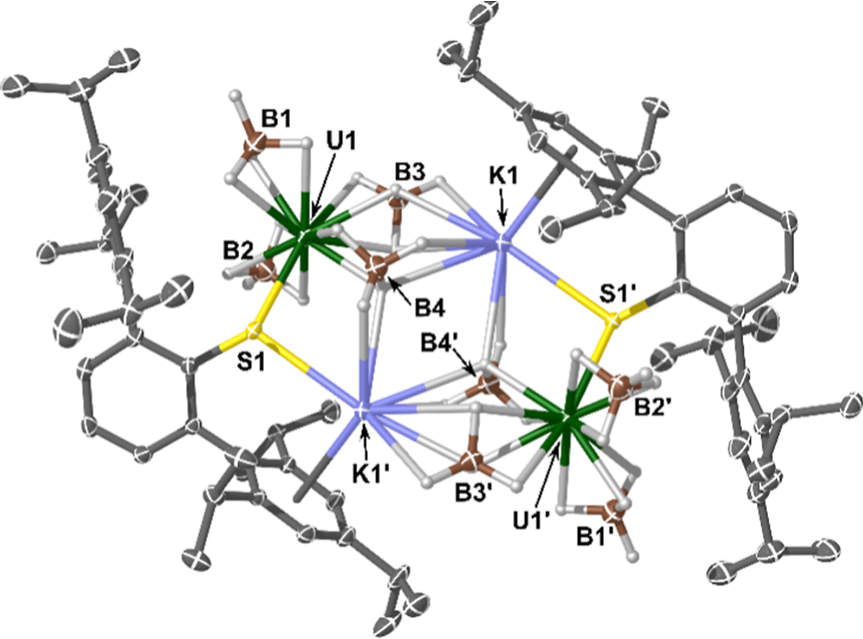
Molecular structure of complex **2**. Ellipsoids set at a 50% probability and non-{BH_4_} H-atoms removed for clarity (operations: X, Y, Z; 1–X, 1–Y, 1–Z). Selected bond lengths and angles: U1–S1 = 2.6948(10) Å, K1–S1 = 3.1109(13) Å, U1···B1 = 2.489(6) Å, U1···B2 = 2.508(7) Å, U1···B3 = 2.557(6) Å, U1···B4 = 2.865(7) Å, K1···η^6^-Tripp_centroid_ = 2.881(2) Å, U1–S1–K1 = 101.94(3)°, and U1–S1–C_ipso_ = 124.88(14)°.

Complex **2** crystallized as a centrosymmetric dimer (*Z*′ = 0.5) comprising two {U^IV^(BH_4_)_4_KSAr^*i*Pr6^} units and is best described as a double salt of [U^IV^(BH_4_)_4_] and [KSAr^*i*Pr6^]—see Figure 3. Each S atom bridges between a U and a K (U–S = 2.6948(10) Å and K–S = 3.1109(13) Å). The U–S bond is slightly longer than in **1** and **1·Et**_**2**_**O** but is shorter than the sum of the covalent radii for a single bond, again implying a polar-covalent bond.^62^ The K–S bond is ca. 0.07 Å longer than in [KSAr^*i*Pr6^]_2_ as a result of coordination to U(IV).^66^ Complex **2** possesses both terminal {κ^3^-BH_4_} units (U···B_BH4_ = 2.489(6) and 2.508(7) Å), and also two different bridging modes: one μ:κ:^3^κ^3^ to both the U and K-atoms (U···B_BH4_ = 2.557(6) Å), whereas the other binds μ^3^:κ:^2^κ:^2^κ^2^ to one U atom (U···B_BH4_ = 2.865(7) Å) and both K-atoms of the dimer—in all cases, these distances compare well to previous structural determinations of parent [U^IV^(BH_4_)_4_]_*n*_^67−71^ and other U(IV)–borohydride complexes.^46,72−94^ Unlike complex **1**, there are no U···η^6^-Tripp interactions in **2**; instead, the softer K atom binds to an η^6^-Tripp group (K···η^6^-Tripp_centroid_ = 2.881(2) Å).

**Figure 4:**
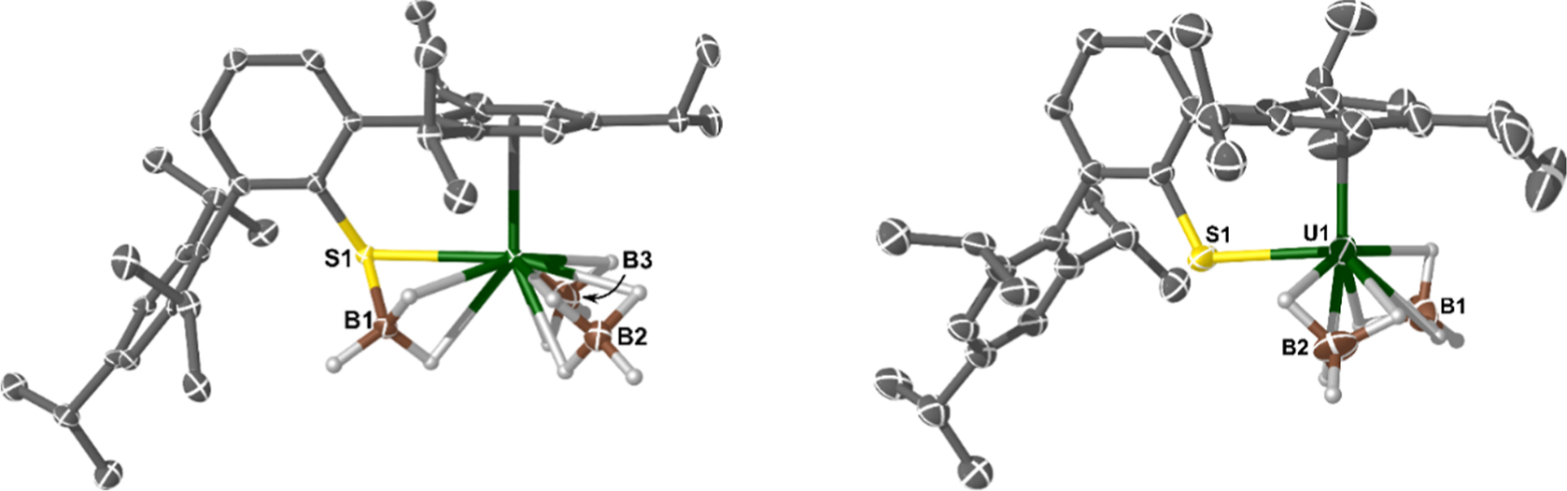
Molecular structure of complexes **3** (left) and **4a** (right). Ellipsoids set at 50% (**3**) and 30% (**4a**) probabilities. Non-{BH_4_} H-atoms and lattice solvent molecules (**3**, toluene) removed for clarity (operations for **3** and **4a**: X, Y, Z). Selected bond lengths and angles for complex **3**: U1–S1 = 2.8824(9) Å, S1–B1 = 1.939(5) Å, U1···B2 = 2.603(7) Å, U1···B3 = 2.584(6) Å, U1···η^6^-Tripp_centroid_ = 2.5379(15) Å, U1–S1–B1 = 64.55(14)°, and U1–S1–C_ipso_ = 109.54(12)°; complex **4a**: U1–S1 = 2.687(4) Å, U1···B1 = 2.56(2) Å, U1···B2 = 2.56(3) Å, U1···η^6^-Tripp_centroid_ = 2.482(7) Å, and U1–S1–C_ipso_ = 113.6(5)°.

The molecular structures of U(III) complexes **3** and **4a** are shown together in Figure 4 (*Z*′ = 1 for both). The U–S bond in **3** (2.8824(9) Å) is elongated compared to **4a** (2.687(4) Å; Δ = 0.195(4) Å) and more so than would be expected from increasing the coordination number at U(IV) (e.g., 6-coordinate = 0.89 Å to 8-coordinate = 1.00 Å; Δ = 0.11 Å).^32^ The coordination of Lewis-acidic BH_3_ reduces the charge density of the S-anion, weakening the U–S interaction and so the U–S distance in **3** is one of the longest for any anionic S-donor to uranium. Conversely, the U–S bond length in **4a** is short compared to other U(III) thiolate complexes. For example, the range of U–S distances in [U^III^(SMes*)_3_] (Mes* = {2,4,6-*t*Bu_3_-C_6_H_2_}) is 2.7127(11) to 2.7247(10) Å, and in Meyer’s complex **A**, the three equivalent U–S distances are 2.7082(7) Å—all of which are longer by a statistically significant amount in **4a**.^17,18^ To the best of our knowledge, complex **4a** has the shortest U–S bond length for any structurally authenticated molecular U(III) complex, and this is likely a reflection of its electron-poor U-center.^65^ The U···B distances in **3** (2.584(6) and 2.603(7) Å) and **4a** (2.56(2) and 2.56(3) Å) are statistically indistinguishable, and the U···η^6^-Tripp_centroid_ distances (**3**, 2.5379(15) Å; **4**, 2.482(7) Å) differ by 0.056(7) Å. These differences suggest that the change in U–S bond length is due to more than just a change in formal coordination number. The S–B bond length in complex **3** (1.939(5) Å) is within the range (1.897(8) to 2.00(2) Å) of the few previously reported complexes containing metal-bound organo-sulfur BH_3_ adducts^95−101^ and to the best of our knowledge is the first example containing an f-element.

**Figure 5:**
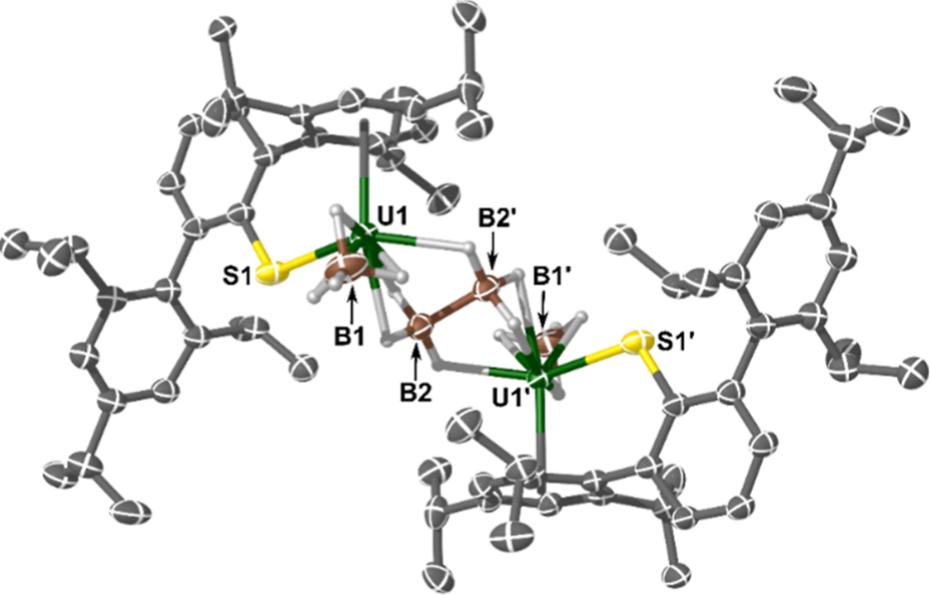
Molecular structure of complex **4b**. Ellipsoids set at a 30% probability. Non-{BH_4_} H-atoms and lattice solvent molecules (pentane) removed for clarity (operations: X, Y, Z; 1–X, 1–Y, 1–Z). Selected bond lengths and angles: U1–S1 = 2.721(3) Å, U1···B1 = 2.556(8) Å, U1···B2 = 2.609(6) Å, U1···B2′ = 2.892(6) Å, U1···η^6^-Tripp_centroid_ = 2.5168(19) Å, and U1–S1–C_ipso_ = 111.7(4)°.

Complex **4b** (shown in Figure 5) crystallized as a dimer (*Z*′ = 0.5) where two {U^III^(SAr^*i*Pr6^)(BH_4_)} units are bridged by a diborane(6) dianion, {B_2_H_6_}^2–^. This structure type is equivalent to *arachno*-tetraborane(10) (B_4_H_10_) wherein two of the {BH_2_} units have been replaced by U(III)-centers, so **4b** is the first example of an f-element *nido*-metalloborane (aside from [U^IV^(BH_4_)_2_{μ-B_2_H_6_}]_*n*_ see Supporting Information).^102^ The {μ-B_2_H_6_}^2–^ in **4b** binds asymmetrically to each U atom (U···B = 2.609(6) and 2.892(6) Å). The B–B bond length of 1.783(12) Å is within the range (1.63(3) to 1.846(4) Å)^60,103,104^ of reported transition metal examples in the CCDC.^65^ The U···B_BH4_ distances (2.556(8) Å) in **4b** are indistinguishable from those in **4a**. The {SAr^*i*Pr6^} ligand in **4b** is disordered over two positions in an 82:18 ratio, giving two distinct U–S bond lengths (2.721(3) and 2.663(17) Å, respectively).

**Figure 6:**
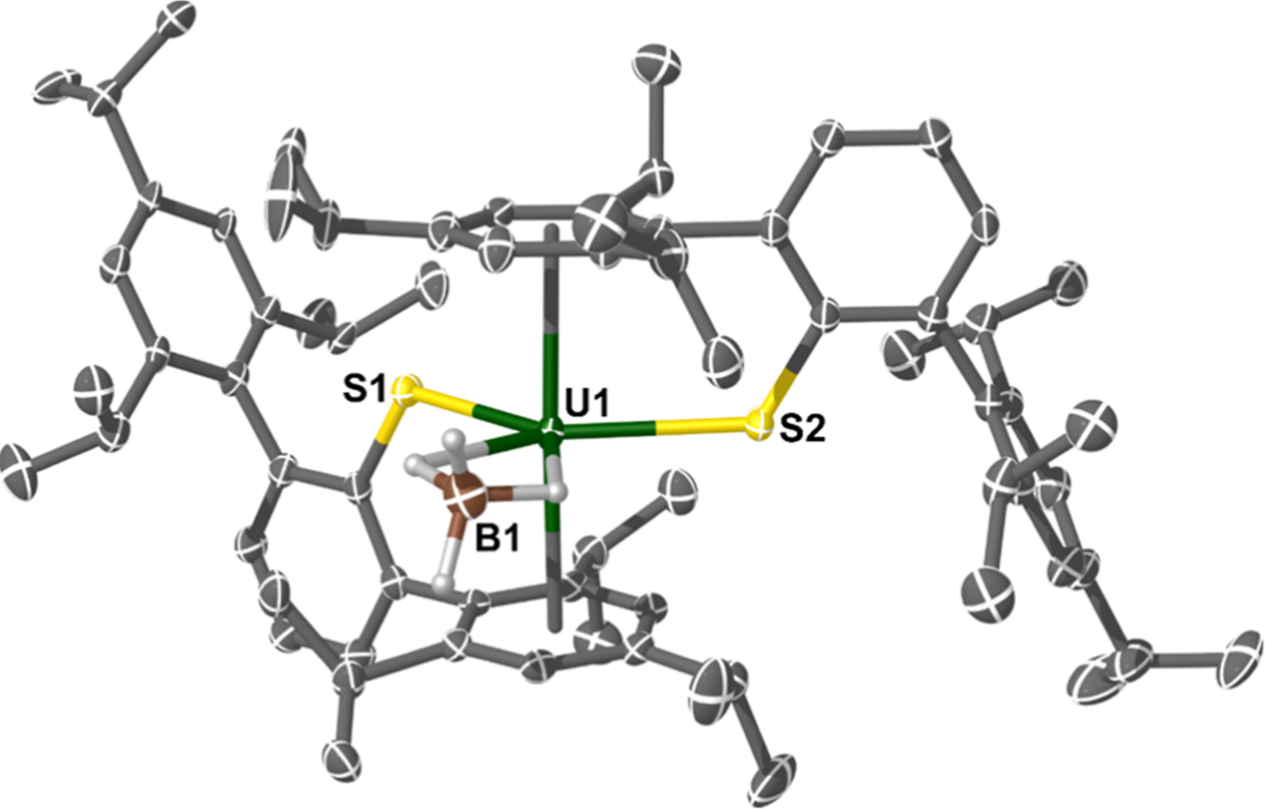
Molecular structure of complex **5**. Ellipsoids set at a 50% probability and non-{BH_4_} H-atoms removed for clarity (operations: X, Y, Z). Selected bond lengths and angles: U1–S1 = 2.7888(8) Å, U1–S2 = 2.7969(7) Å, U1···B1 = 2.872(4) Å, U1···η^6^-Tripp_centroid1_ = 2.744(2) Å, U1···η^6^-Tripp_centroid2_ = 2.747(2) Å, U1···S_2_B-plane = 0.004(2) Å, S1–U1–S2 = 128.23(2)°, U1–S1–C_ipso_ = 118.15(11)°, U1–S2–C_ipso_ = 118.53(11)°, and η^6^-Tripp_centroid1_···U1···η^6^-Tripp_centroid2_ = 176.79(4)°.

The molecular structure of complex **5** (Figure 6) shows the U atom is sandwiched equally between two Tripp arene rings (U···η^6^-Tripp_centroid_ = 2.744(2) and 2.747(2) Å) and is almost perfectly within the S_2_B plane (deviation = 0.004(2), below statistical significance). Despite this, **5** does not possess crystallographic *C*_2_ symmetry (*Z*′ = 1), and the two U–S bonds are distinct (2.7888(8) and 2.7969(7) Å; Δ = 0.0081(10) Å). The U–S lengths in **5** are longer than those in both U(IV) complexes **1** and **2** and the U(III) complexes **4a** and **4b**, whereas those of complex **3** (2.8824(9) Å) are almost 0.1 Å longer than **5**. In both **3** and **5**, these values are longer than the sum of the covalent radii for a U–S single bond (2.73 Å)^62^ and also longer than those in [U^III^(SMes*)_3_] (2.7127(11) to 2.7247(10) Å) and complex **A** (2.7082(7) Å).^17,18^ Lastly, the U–S distances in **5** are shorter than those in [La(SAr^*i*Pr6^)_2_(I)] (2.8235(12) and 2.8173(10) Å) by more than the difference in the ionic radii of U(III) (1.025 Å) and La(III) (1.032 Å), which is often ascribed to covalent contributions to the bonding in uranium complexes that are absent in the lanthanum congeners.^32,40^ The U···B_BH4_ distance in **5** (2.872(4) Å) is larger than the other U(III) and U(IV) complexes presented herein, though it is similar to other terminal and bridging U-{κ^2^-BH_4_} distances previously reported for U(III) complexes.^16,45,47^

### UV–Vis–NIR Spectroscopy

**Figure 7:**
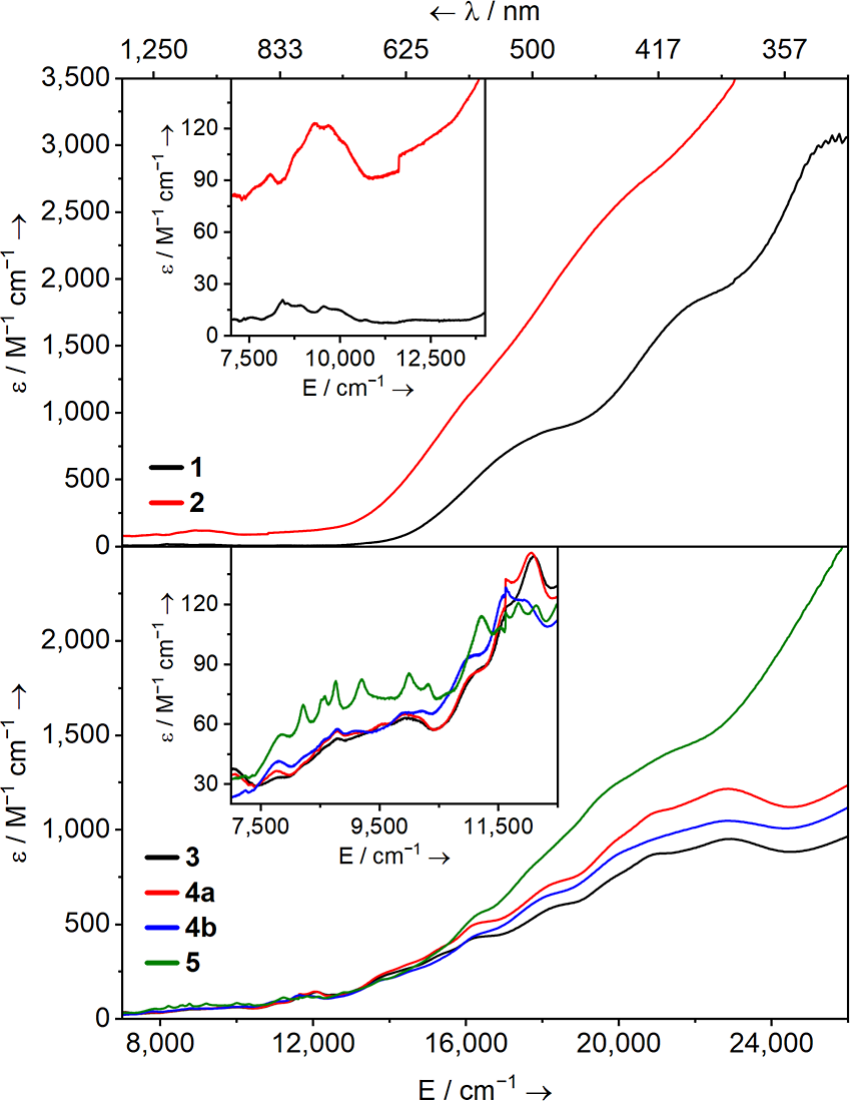
Solution UV–Vis–NIR spectra of U(IV) **1** and **2** (top panel) and **3**–**5** (bottom panel)—all as 1.0 mM (of U content) in Et_2_O at ambient temperature.

The UV–Vis–NIR spectra of complexes **1** and **2**–**5** were recorded at room temperature as Et_2_O solutions and are shown in Figure 7. Note that in the case of **2**, while exposure of the crude reaction mixture to Et_2_O caused decomposition to (SAr^*i*Pr6^)_2_, this was not the case for the isolated crystalline material.

The electronic absorption spectra for **1** and **2** are typical for U(IV): **1** features two broad charge transfer features with maxima at ca. 26,000 cm^–1^ (385 nm, ε ca. 1800 M^–1^ cm^–1^) and 20,000 cm^–1^ (500 nm, ε ca. 880 M^–1^ cm^–1^) which tail off below 14,000 cm^–1^ (715 nm); **2** displays a broad absorbance across the whole visible range which then tails off below 14,000 cm^–1^. The NIR region for both feature weak Laporte-forbidden intraconfigurational f–f transitions (**1**, ε ca. 15 M^–1^ cm^–1^; **2**, ε ca. 100 M^–1^ cm^–1^).^105−108^ The UV–Vis region of complexes **3**, **4a**, and **4b** are remarkably similar, showing multiple strong overlapping absorptions with maxima centered around 22,880 cm^–1^ (437 nm), 21,000 cm^–1^ (476 nm), 19,940 cm^–1^ (502 nm), 18,115 cm^–1^ (552 nm), and ca. 16,195 cm^–1^ (617 nm) with molar absorptivity values (ε) ranging from ca. 400 to 1200 M^–1^ cm^–1^. These broad features then tail into the NIR region below 14,000 cm^–1^ (715 nm). Note that due to the presence of an additional crystalline phase in the PXRD of **3**, the ε values should be taken as approximate. The UV–Vis–NIR spectrum of **5** is similar to that of [U(NHAr^*i*Pr6^)_2_(I)],^36^ with a broad charge transfer feature at ca. 20,512 cm^–1^ (488 nm, ε ca. 1333 M^–1^ cm^–1^). The NIR region of the mono-arylthiolate U(III) complexes **3**, **4a**, and **4b** features a broad series of poorly defined Laporte-forbidden intraconfigurational f–f transitions (ε ca. 60 M^–1^ cm^–1^). On the other hand, complex **5** shows well-defined features between 7830 cm^–1^ (1277 nm) and 12,173 cm^–1^ (821 nm; ε ca. 55–118 M^–1^ cm^–1^).

### SQUID Magnetometry

**Figure 8:**
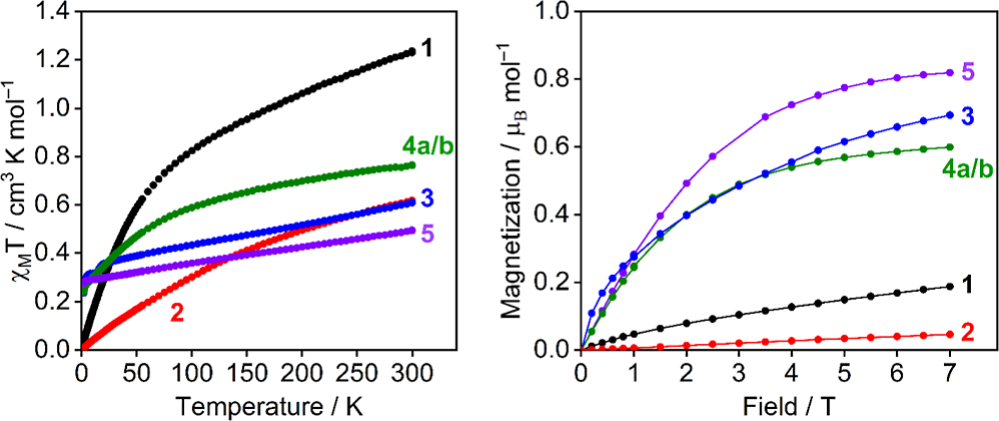
Variable-temperature SQUID magnetic moment data (χ*T*, left) over the temperature range of 1.8–300 K in an applied field of 1000 Oe and magnetization (right) over the field range of 0–70 000 Oe at 1.8 K for **1** (black), **2** (red), **3** (blue), **4a**/**b** (green), and **5** (purple).

We examined the magnetic properties of complexes **1**–**3**, **4b**, and **5** using variable-temperature SQUID magnetometry (see Figure 8 and Supporting Information) to better understand their ground-state electronic structures as room-temperature magnetic moments do not allow for the unambiguous assignment oxidation state in U(III) or U(IV) complexes.^109^

For U(IV) (5f^2^, ^3^H_4_ at the *LS* limit) complexes **1** and **2**, effective magnetic moments of 3.14 μB (1.24 cm^3^ mol^–1^ K; **1**) and 2.22 μB (0.62 cm^3^ mol^–1^ K; **2** per U ion) were measured at 300 K. These values decrease smoothly over the temperature range reaching 0.51 μB (0.03 cm^3^ mol^–1^ K; **1**) and 0.24 μB (0.01 cm^3^ mol^–1^ K; **2** per U ion) at 1.8 K and tend to zero. This behavior is typical of the thermal depopulation of crystal field states of the split ^3^H_4_ ground multiplet into an orbital singlet ground state, which is common for U(IV).^7,109^ An isolated f^2^ ion has an integer spin, so it can be an orbital singlet at low temperatures, subject to temperature-independent paramagnetism (TIP), resulting in non-zero magnetic moments. However, the magnetic profiles of **1** and **2** exhibit notable differences. The curve does not fall as quickly for **1** as it does for **2**, which is characteristic of strongly donating or charge-rich ligands exerting larger crystal field effects on U(IV) ions.^110^ The effective magnetic moments at 300 K for **1** and **2** differ significantly (3.14 versus 2.22 μB both per U ion), and both are markedly reduced from the free-ion value (3.58 μB) which we attribute to crystal field effects resulting in different degrees of excited state mixing into the ground state.^111,112^ Isothermal magnetization versus field measurements at 1.8 K for **1** and **2** exhibit low values of magnetization, with no sign of approaching saturation up to the highest field (0.19 μB mol^–1^ for **1** and 0.05 μB mol^–1^ per U ion for **2** at 7 T and 1.8 K) consistent with their non-Kramers U(IV) formulations. In short, all the data for **2** (see the Supporting Information) suggest it contains two non-interacting U(IV) ions.

For U(III) (5f^3^, ^4^I_9/2_ at the *LS* limit) complexes **3** and **5**, effective magnetic moments of 1.99 μB (0.50 cm^3^ mol^–1^ K; **3**) and 2.47 μB (0.76 cm^3^ mol^–1^ K; **5**) were measured at 300 K. These values exhibit a slow reduction across the temperature range reaching 1.49 μB (0.28 cm^3^ mol^–1^ K; **3**) and 1.39 μB (0.24 cm^3^ mol^–1^ K; **5**) at 1.8 K. This is a result of the thermal depopulation of crystal field states of the ^4^I_9/2_ ground multiplet into an orbital doublet ground state giving rise to higher (nonzero) low-temperature moments, which is typical for U(III).^109,113^ For both, the effective magnetic moment at 300 K is low compared to that of the theoretical U(III) ^4^I_9/2_ free-ion value (3.62 μB); however, this has been observed for other arene-anchored U(III) complexes.^18,114,115^ Isothermal magnetization versus field measurements for **3** and **5** at 1.8 K exhibit magnetization values commensurate with U(III) Kramers ion configurations, approaching saturation up to the highest field (0.82 μB mol^–1^ for **3** and 0.59 μB mol^–1^ for **5** at 7 T and 1.8 K).

In the case of **4a** and **4b**, ATR–IR data suggest that both are present in both batches of crystalline material. Given the difference between these two complexes is one unit of H_2_, their per-uranium magnetic properties should be comparable provided there are no super-exchange interactions through the {B_2_H_6_}^2–^ bridge, so we have collected data for the mixture, henceforth **4a**/**b**. Both the low- and high-temperature magnetic susceptibilities for **4a**/**b** (300 K: 2.21 μB, 0.61 cm^3^ mol^–1^ K; and 1.8 K: 1.38 μB, 0.24 cm^3^ mol^–1^ K—all per U ion) are in good agreement with those of **3** and **5** and therefore for a U(III) composition. Likewise, the isothermal magnetization for **4a**/**b** approaches saturation at the highest fields, reaching 0.69 μB mol^–1^ per U ion at 7T and 1.8 K. The similarity of the per-ion magnetic profile to that of **3** and **5** suggests that if any magnetic communication between the two U-atoms is present, it is very weak and that the magnetic contribution from **4b** to the data is what would be expected from a pair of magnetically isolated U(III) ions.

### Electronic Structure Calculations

To better understand the nature of the U–S bonding and U···arene interactions in these complexes, unrestricted Kohn–Sham density-functional theory (DFT) calculations were performed in ORCA 5.0^116^ using the PBE0 functional^117,118^ on H-atom optimized coordinates for **1·Et**_**2**_**O** (*S* = 1), **2** (*S* = 2), **3**, **4a**, **5** (*S* = 3/2), and **4b** (*S* = 3) derived from the SC-XRD data. In the case of **1**, all coordinates were optimized due to the presence of ten crystallographically independent molecules (see the Supporting Information). For complex **1**, the (mean) SC-XRD and calculated U–S and U–Cl distances differ by ≤0.005 Å except for the U–S linkage for the {SAr^*i*Pr^} ligand with a U···η^6^-Tripp contact which differs by 0.014 Å, though this is within 2σ of the mean and so represents a sound model.

Computational analyses of atomic charges derived from Kohn–Sham molecular orbitals (KS MOs) are fraught with difficulty,^119−122^ and the differing coordination numbers in **1**–**5** preclude meaningful direct comparisons. However, a comparison of the Mulliken charges (*Q*_AM_) and spin populations for the S-atoms (*S*_M_ S) is instructive toward the nature of their interaction with the U-atoms. In a similar fashion, the U-atom spin populations (*S*_M_ U) report whether there is a net exchange of spin density away from, or toward, the metal. These data are summarized in Table 2 along with the Mayer bond orders and the U–S bond lengths from SC-XRD, or calculations in the case of **1**. The U–S MBO values are broadly in agreement with other examples of U(III) and U(IV) thiolate linkages.^18,123^

**Table 2:** Spin population (*S*_M_) and Mulliken atomic charge (*Q*_AM_) analyses and the U–S Mayer bond orders (MBO) and crystallographic bond lengths for the U–S linkages of complexes **1**–**5**.

complex	*S*_M_ U	*Q*_AM_ S	*S*_M_ S	U–S MBO	U–S bond (Å)
**1**	2.23	–0.33	–0.07	1.04	2.678^a^
		–0.45	–0.08	0.86	2.653^a^
**1·Et**_2_**O**	2.23	–0.46	–0.05	0.76	2.6526(9)
		–0.46	–0.05	0.76	
**2**^b^	2.20	–0.51	–0.06	0.82	2.6948(10)
**3**	3.06	–0.17	–0.02	0.50	2.8824(9)
**4a**	3.06	–0.39	–0.05	0.94	2.687(4)
**4b**^b^	3.04	–0.41	–0.04	0.97	2.721(3)
**5**	3.15	–0.41	–0.04	0.71	2.7888(8)
		–0.41	–0.04	0.71	2.7969(7)

a Bond lengths from the fully optimized structure, so no ESD is given.

b Values are given for only one of the U-atoms.

The U-atom spin populations of 2.23, 2.23, and 2.20 for complexes **1**, **1·Et**_**2**_**O**, and **2**, respectively, show they are net importers of spin density as all somewhat exceed the expected values of 2 for U(IV) 5f^2^; whereas values of 3.06, 3.06, and 3.04 for **3**, **4a**, and **4b**, respectively, are in good agreement with their U(III) 5f^3^ configurations. Complex **5** (3.15) is an outlier. These data show that (i) there is a weak positive correlation between the number of S (and Cl) donors and increased U-atom spin population; (ii) there is no clear correlation between the MBO and the U-atom oxidation state; (iii) complex **3**, for which the coordination of BH_3_ to the S-atom withdraws charge, and also complex **5**, possess the lowest U–S MBO values; (iv) complex **5** is the only U(III) complex with a notable deviation of the spin population from the ideal value.

While U···η^6^-arene δ-bonding interactions are a common feature in low-oxidation-state uranium complexes with arene ligands,^7,18,36,115,124−135^ inspection of the KS-MOs in all complexes herein reveals no δ or π U-arene overlap in the occupied orbitals, though most show such an interaction in their first unoccupied orbitals. This is likely due to the arene groups sitting further from the U(III) center (e.g., in **5**, U···η^6^-Tripp_centroid_ = 2.744(2) and 2.747(2) Å) than in similar complexes such as **A** (U···η^6^-Mes_centroid_ = 2.464(1) Å) or a recently reported U(III) *tris*-boryloxide complex (U···η^6^-arene_centroid_ = 2.616(2) Å)—both of which exhibit δ-bonding interactions.^18,115^

**Figure 9:**
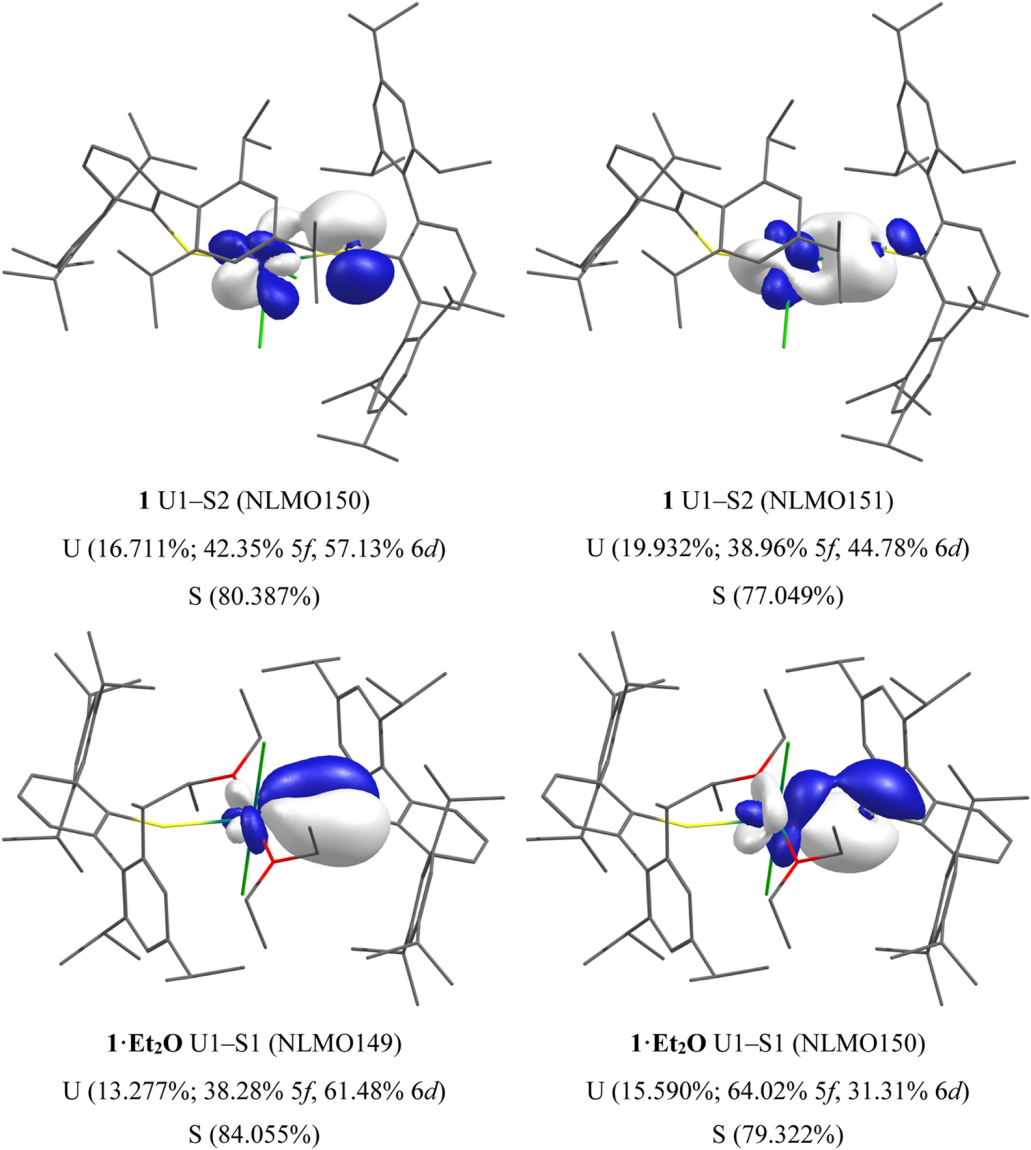
Selected NLMO isosurfaces (0.05 au) for **1** (top) and **1·Et**_**2**_**O** (bottom).

Due to the delocalized nature of KS MOs, we next turn to the analysis of the U–S interactions using the natural bond order (NBO) and quantum theory of atoms in molecules (QTAIM) formalisms. For complex **1**, the two inequivalent U–S bonds decompose into four NLMOs with U-contributions ranging from 14 to 20%, divided almost equally between 6d and 5f contributions in three of the four cases, the last being >60% U 5f—see Figure 9. In **1·Et**_**2**_**O**, the U–S π-bonding is prominent (Figure 9) with a U-content of 13.3% (38.28% 5f, 61.48% 6d) which is dominated by the 6d contribution. The corresponding σ-bond is poorly localized (U, 15.6%; 64.02% 5f, 31.31% 6d) and has a comparatively larger 5f composition than the π-bond. Note that the σ and π notation used here is a guide for the symmetry of interaction and that the U–S overlap is often poorly oriented as has been observed elsewhere due to the low symmetry of the complexes.^136,137^

**Figure 10:**
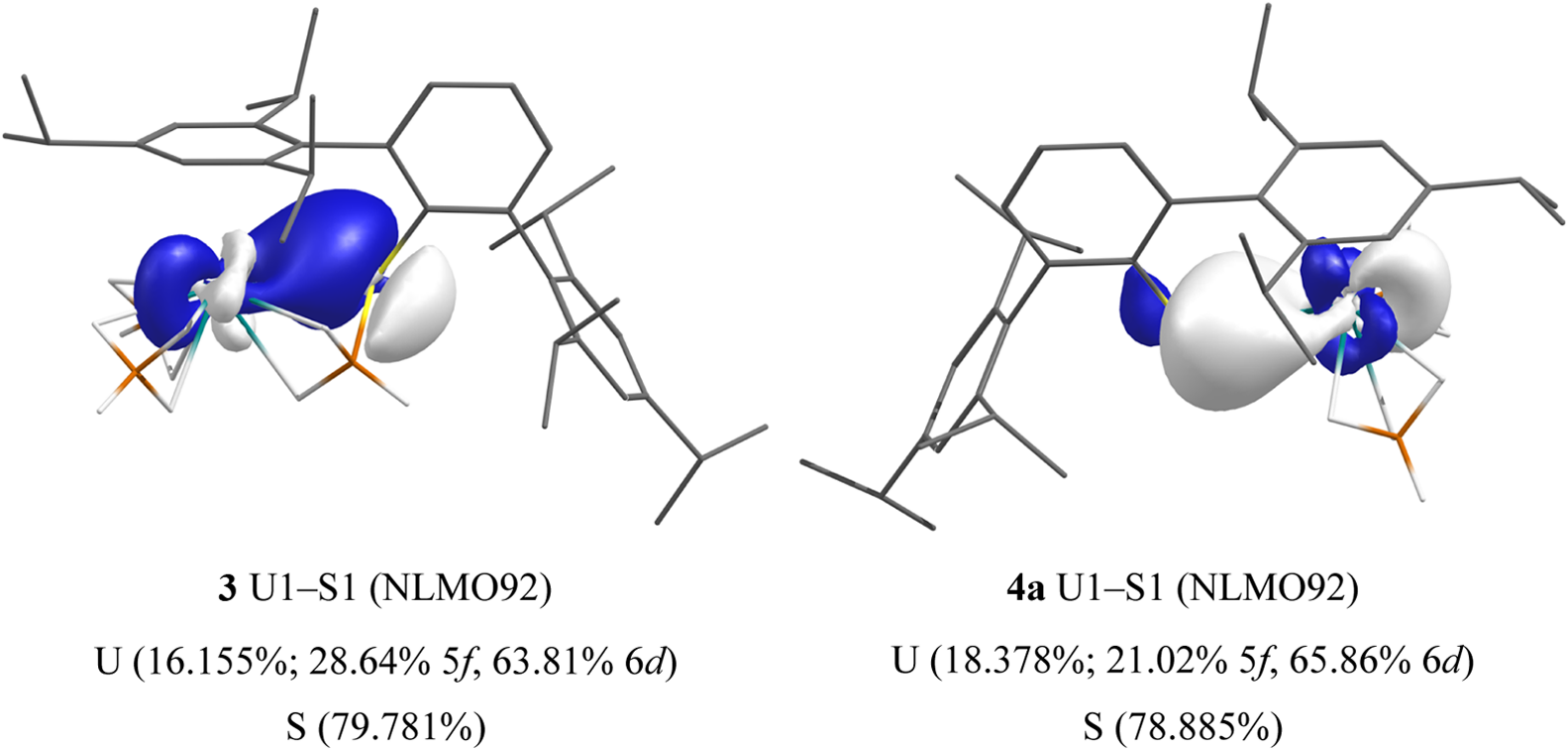
Selected NLMO isosurfaces (0.05 au) for **3** (left) and **4a** (right).

For complex **2**, both σ- and π-components for the U–S bond were found, with the σ-bond (U, 25.431%; 34.53% 5f, 57.02% 6d) bearing a larger uranium (and 6d) component than the corresponding π-bond (U, 14.193%; 61.94% 5f, 37.51% 6d). In complex **3**, the BH_3_ group occupies the S-atom lone pair and only a U–S σ-bond is found (U, 16.155%; 28.64% 5f, 63.81% 6d). Despite the S-atom lone pair being available for U–S π-bonding in complex **4a**, we find two poorly oriented interactions (12.022% and 18.378% U-character) with approximate σ-symmetry which compare well to the U–S σ-bond in **3**—all show dominant U 6d character as would be expected from Bursten’s FEUDAL description of actinide bonding (see Figure 10 for both **3** and **4a**).^138^ Complex **4b** is comparable to **4a**, and so the data are presented in the Supporting Information. Finally, in complex **5**, we find that each U–S interaction is spread across three NLMOs, each showing poor orientation of the respective parent atomic orbitals and low U-content (3.708–10.492%). At low isovalues (<0.03 au), U–S π-bonds can be seen, but clearly the interaction is essentially ionic in nature due to poor overlap. NLMO compositions for **1**–**5** are collated in Table S18.

Bader’s QTAIM^139^ is a topological analysis of the surface density between atoms and provides intuitive definitions and metrics for chemical bonding that are complementary to the NBO and NLMO analysis above.^140^ Here, we focus on the QTAIM charges for the U and S-atoms of each complex,^122^ along with the delocalization [δ(U,S)] and localization [LI(U)] indices, the electron density (ρ), Laplacian of the electron density (∇^2^ρ), and energy density (*H*) at the bond critical point (BCP) for the U–S bonds in each complex to describe the nature of the U–S interaction.

The δ(U,S) values for the U–S bonds in **1** (0.761, 0.807) are larger than for **1·Et**_**2**_**O** (0.690), and this is reflected at the bond critical points (BCPs) where for **1**, both ρ (0.072, 0.072) and H (−0.019, −0.019) are larger in magnitude than in **1·Et**_**2**_**O** (ρ = 0.060, *H* = −0.012). The small ρ values and positive ∇^2^ρ for both **1** and **1·Et**_**2**_**O** describe polar U^IV^–S interactions, while the modest values for δ(U,S) and negative H-values describe a small covalent contribution. The metrics for complex **2**, δ(U,S) (0.699), ρ (0.067), and H (−0.016) lie between those of **1** and **1·Et**_**2**_**O**. These properties are summarized in Table 3 for all complexes.

**Table 3:** QTAIM charges, electron densities (ρ), Laplacian of the electron density (∇^2^ρ), energy densities (Η), delocalization indices (δ), and localization indices (LI) of complexes **1**–**5**.

complex	QTAIM charge (U/S)	ρ	∇^2^ρ	*H*	δ(U,S)	LI(U)	ΔLI(U)^a^
**1**	+2.160/–0.438	0.072	0.0914	–0.019	0.761	87.717	0.283
	+2.160/–0.541	0.073	0.099		0.804		
**1·Et**_2_**O**	+2.354/–0.567	0.060	0.137	–0.012	0.690	87.622	0.378
					0.691		
**2**	+2.271/–0.556	0.067	0.096	–0.016	0.699	87.603	0.397
					0.700		
**3**	+1.968/–0.535	0.044	0.095	–0.007	0.397	88.197	0.803
**4a**	+1.960/–0.498	0.065	0.110	–0.016	0.671	88.295	0.705
**4b**	+1.944/–0.498	0.063	0.104	–0.015	0.634	88.232	0.768
	+1.943/–0.498	0.063	0.104	–0.015	0.635		
**5**	+1.935/–0.533	0.055	0.101	–0.011	0.570	88.475	0.525
		0.054	0.099		0.563		

a Corresponds to the difference between the ideal values for U(IV) (88) and U(III) (89) and the calculated values for each complex.

When comparing U(IV) complexes **1**, **1·Et**_**2**_**O**, and **2** to U(III) complexes **3**, **4a**, **4b**, and **5**, we observe a decrease in all topological metrics commensurate with the decreased effective charge at U(III), though we note that QTAIM metrics are highly dependent on internuclear distances, which complicates this comparison. The effect of the S-bound BH_3_ group in **3** is pronounced as ρ (0.044), *H* (−0.007), and δ(U,S) (0.397) are significantly reduced compared to those in **4a** (0.065, −0.016, and 0.671, respectively). There are negligible differences in the U–S bonds between complexes **4a** and **4b** (see Table 3). Analysis of complex **5** reveals U–S bonding somewhat more ionic than the other complexes herein, though, again, the U–S distance is somewhat longer than for other complexes. Metrics for the U–S bonding in the U(III) complexes here are like those reported elsewhere.^18,123^

The localization index, LI(M), can be used to examine the metal–element linkages. Deviations in the values returned for the metal indicate charge transfer interactions. Values of 88 for U(IV) and 89 for U(III) would be expected in a purely ionic system. Reduced values are found for all complexes, with deviations of 0.28–0.40 for the U(IV) complexes (**1**, **1·Et**_**2**_**O**, and **2**) and 0.52–0.80 for the U(III) systems (**3**, **4a**, **4b**, and **5**), suggesting charge transfer, and this is in line with qualifying covalent assessments made earlier. Meyer and co-workers have used this metric to assess the U(III) arene-anchored *tris*-thiophenolate complex, [U^III^{(SAr^Ad,Me^)_3_Mes}], with a reported LI(U) of 88.25 (a deviation of 0.75).^18^

## Conclusions

In summary, we have expanded the field of molecular uranium organo-sulfur complexes to include *meta*-terphenyl derivatives, demonstrating diverse bonding modes and coordination environments facilitated by the weakly coordinating arene rings of the {SAr^*i*Pr6^} framework. These results include examples of both the shortest and one of the longest U^III^–S distances to date for anionic sulfur donors to uranium.

Limited success was found in using U^IV^Cl_4_ in salt elimination reactions with KSAr^*i*Pr6^ for the isolation of U(IV) complexes, with both [U^IV^(SAr^*i*Pr6^)_2_(Cl)_2_] and [U^IV^(SAr^*i*Pr6^)_2_(Cl)_2_(Et_2_O)_2_] being obtained in low yield. While the reaction between [U^IV^(BH_4_)_4_] and 1 equiv of KSAr^*i*Pr6^ gave the double salt [U^IV^(μ-SAr^*i*Pr6^)(BH_4_)_2_(μ-BH_4_)(μ^3^-BH_4_)K]_2_ in toluene, or oxidative coupling to give (SAr^*i*Pr6^)_2_ in Et_2_O; 1 equiv of HSAr^*i*Pr6^ reacted with [U^IV^(BH_4_)_4_] under thermal conditions in toluene to give the U(III) sulfur–borane complex [U^III^(H_3_B·SAr^*i*Pr6^-κ*S*,*H*,*H*)(BH_4_)_2_], the first such example with an f-element. Salt elimination using [U^III^(BH_4_)_3_(toluene)] and 1 equiv of KSAr^*i*Pr6^ allowed access to borane-free [U^III^(SAr^*i*Pr6^)(BH_4_)_2_] which appears to undergo slow thermolysis to [{U^III^(SAr^*i*Pr6^)(BH_4_)}_2_(μ-B_2_H_6_)]. The latter complex contains a diborane(6) dianion and is the first example of an f-element *nido*-metalloborane. The *bis*-terphenylthiolate complex [U^III^(SAr^*i*Pr6^)_2_(BH_4_)] bearing two U···η^6^-arene interactions was isolated from the reaction between [U^III^(BH_4_)_3_(toluene)] and 2 equiv of KSAr^*i*Pr6^.

Quantum chemical analysis of the U–S bonding in all complexes reveals polar covalent interactions with both σ- and π-contributions to the bonding in some cases, although low symmetry in all complexes precludes efficient orbital overlap. In [U^III^(H_3_B·SAr^*i*Pr6^-κ*S*,*H*,*H*)(BH_4_)_2_], the BH_3_ Lewis-acid cap of the S-donor reduces the U–S interaction. Despite the prevalence of U···η^6^-arene interactions in the molecular structures of most complexes herein, no substantial U–arene π- or δ-bonding interactions were found within the occupied molecular orbitals, and a topological analysis corroborated this.

## Experimental Details

### Equipment, Materials, and Solvents

Caution! The natural-abundance U (assumed standard composition: 0.7204% ^235^U, *t*_1/2_ = 7.04 × 10^8^; 99.2742% ^238^U, *t*_1/2_ = 4.468 × 10^9^) used in this work, along with the α-, β-, and γ-emitting decay products present radiotoxicity (α-particles) and heavy-metal health hazards. Hence, all manipulations of these materials was performed in continuous extraction fume cupboards or positive pressure inert atmosphere gloveboxes located in a dedicated laboratory with contamination monitoring protocols, along with training and materials for decontamination equipment. Additional safeguards include the use of hand-held radiation monitoring equipment.

Unless otherwise described, all syntheses and manipulations were conducted under BOC PureShield argon (99.995%) with rigorous exclusion of oxygen and water using Schlenk line and glovebox techniques in an MBraun Lab Star or a Glovebox Systemtechnik MEGA 4. 3 Å molecular sieves were activated by heating for 8 h at 300 °C and 10^–3^ mbar. THF, *n*-hexane, *n*-pentane, Et_2_O, and toluene were degassed by sparging (N_2_) and dried by passage through neutral alumina columns (INERT Corp.). THF was then degassed under a vacuum and stored over 3 Å molecular sieves for 7 days before use. *n*-Hexane, *n*-pentane, Et_2_O, and toluene were degassed under vacuum, stored over a K mirror, and used immediately. *d*_6_-Benzene (Merck) was dried by refluxing over K metal for 4–5 days then vacuum-transferred to a J. Young valve-appended vessel. U^IV^Cl_4_ and [U^IV^(BH_4_)_4_] were prepared as described previously.^48,141^ KSAr^*i*Pr6^ was prepared from HSAr^*i*Pr6^ and K^0^ in toluene.^38,39^

#### Synthesis of [U^IV^(SAr^*i*Pr6^)2(Cl)_2_] (**1**) and [U^IV^(SAr^*i*Pr6^)_2_(Cl)_2_] (**1·Et**_**2**_**O**)

Et_2_O (20 mL) was added to a precooled (−78 °C) stirring mixture of solid U^IV^Cl_4_ (0.190 g, 0.50 mmol) and KSAr^*i*Pr6^ (0.553 g, 1.00 mmol, 2 equiv) in a glass Schlenk vessel equipped with a PTFE-coated stirrer bar. The mixture was then allowed to warm to room temperature and stirred overnight (18 h), whereupon a pale-red solution formed along with a dark-red precipitate. The solids were allowed to settle before the Et_2_O solution was filtered through a glass microfiber filter disc. Concentration of the clear solution to ca. 2 mL gave colorless solids, which were gently heated in the solution. Storage of this solution at 5 °C gave a small crop of KSAr^*i*Pr6^ as colorless crystals, verified by SC-XRD and NMR spectroscopy (0.052 g, 0.10 mmol). The red solid, which remained after filtration of the Et_2_O supernatant, was extracted with toluene (10 mL) and then dried under vacuum (10^–3^ mbar), which left a bright-red powder. This solid was then extracted into hot hexane (10 mL), concentrated to ca. 5 mL, and stored at −30 °C for 7 days, whereupon a single crop of [U^IV^(SAr^iPr6^)_2_(Cl)_2_] (**1**) formed as red microcrystals (yield = 0.132 g, 0.10 mmol, 20% based on U^IV^Cl_4_). On one occasion, a few crystals of [U^IV^(SAr^*i*Pr6^)_2_(Cl)_2_(OEt_2_)_2_] (**1·Et**_**2**_**O**) were isolated from a concentrated toluene solution stored at −30 °C; however, characterization beyond single-crystal XRD was not possible.

Elemental analysis on C_72_H_98_Cl_2_S_2_U calcd (%): C, 64.70; H, 7.39; N, 0.00. Found (%): C, 65.00; H, 7.52; N, 0.00.

^1^H NMR (*d*_6_-benzene, 400.07 MHz, 298 K): δ 16.85 (br s,  = 195 Hz), 10.60 (s), −1.00 (br s, 133 Hz).

UV–Vis–NIR (Et_2_O): λ_max_ (cm^–1^; ε) A number of weak and broad transitions are present between 1274 nm (7846 cm^–1^) and 950 nm (10,521 cm^–1^) −1187 (8421; 21), 1172 (8532; 19), 1126 (8881; 18), 1048 (9541; 17), 1001 (9995; 15). A broad feature extends from ∼715 nm (14,000 cm^–1^) into the UV region, and beyond our spectral range, a broad shoulder at 500 (20,000; 880) and 385 (26,000; 1800) is present.

FT-IR (ATR, microcrystalline) cm^–1^: 3052 (vw), 2957 (s), 2924 (m), 2865 (m), 1605 (w), 1568 (w), 1459 (m), 1381 (m), 1361 (m), 1317 (m), 1260 (w), 1237 (w), 1169 (w), 1153 (w), 1109 (m), 1071 (w), 1040 (m), 937 (m) 873 (m), 849 (vw), 799 (m), 772 (m), 746 (m), 730 (m), 652 (m), 606 (vw), 588 (vw), 529 (w), 427 (w).

#### Synthesis of [U^IV^(μ-SAr^*i*Pr6^)(BH_4_)_2_(μ-BH_4_)(μ^3^-BH_4_)K]_2_ (**2**)

Method A. Toluene (15 mL) was added to a precooled (−78 °C) stirring mixture of solid [U^IV^(BH_4_)_4_] (0.149 g, 0.50 mmol) and KSAr^*i*Pr6^ (0.276 g, 0.50 mmol, 1 equiv) in a glass Schlenk vessel equipped with a PTFE-coated stirrer bar. The pale-brown mixture was stirred for 5 min at −78 °C before warming to room temperature. The solution became dark brown upon warming and was stirred for 3 h at room temperature, after which the volatiles were removed under vacuum (10^–3^ mbar) to afford a brown solid. The solid was triturated with hot (∼50 °C) hexane (15 mL), affording a brick-red powder after drying. This powder was extracted with toluene (10 mL) through a glass microfiber filter disc. Concentration of the brown solution to ca. 1 mL deposited a small quantity of dark-red microcrystalline material whose identity was confirmed to be [U^IV^(μ-SAr^*i*Pr6^)(BH_4_)_2_(μ-BH_4_)(μ^3^-BH_4_)K]_2_ (**2**) by single-crystal X-ray diffraction. A further small crop was isolated at room temperature (combined yield = 0.022 g, 0.01 mmol, 3%).

Method B. Toluene (10 mL) was added to a precooled (−78 °C) stirring mixture of solid [U^IV^(BH_4_)_4_] (0.149 g, 0.50 mmol) and KSAr^*i*Pr6^ (0.276 g, 0.50 mmol, 1 equiv) in a glass Schlenk vessel equipped with a PTFE-coated stirrer bar. The mixture immediately became dark red/brown with a red precipitate presumed to be [U^IV^(μ-SAr^*i*Pr6^)(BH_4_)_2_(μ-BH_4_)(μ^3^-BH_4_)K]_2_ (**2**). After stirring for 5 min at −78 °C, the mixture was allowed to warm to room temperature and stirred overnight (18 h). The red solids were allowed to settle before filtration through a glass microfiber filter disc. The red solids were then dried under vacuum (10^–3^ mbar, 2 h) to afford **2** as a pale-red powder (yield = 0.113 g, 0.07 mmol, 26%). The ATR–IR spectrum was found to be superposable with that obtained for crystalline **2** isolated by method A. An ATR–IR spectrum gathered from the supernatant after drying under vacuum showed marked differences, but we could not obtain any material suitable for single-crystal X-ray diffraction from it, so we could not determine the structure of this material.

Elemental analysis on C_72_H_130_B_8_K_2_S_2_U_2_ calcd (%): C, 50.85; H, 7.71; N, 0.00. Found (%): C, 51.40; H, 7.78; N, 0.00.

^1^H NMR (*d*_6_-benzene, 400.07 MHz, 298 K): δ 87.15 (s), 27.87 (s), 26.84 (s), 14.46 (br s,  = 64 Hz), 13.70 (br s,  = 120 Hz), 9.11 (br s,  = 17 Hz), 0.81 (br s,  = 62 Hz), 0.63 (br s,  = 33 Hz), −0.38 (br s,  = 17 Hz), −4.54 (br s,  = 88 Hz), −7.88 (s), −9.83 (s), −12.25 (s), −14.82 (s), −18.00 (s), −22.87 (s), −29.49 (s), −54.66 (s), −76.69 (s).

^11^B NMR (*d*_6_-benzene, 128.36 MHz, 298 K): δ 141.3 (br s,  = 506 Hz).

UV–Vis–NIR (Et_2_O): λ_max_ (cm^–1^; ε) 1235 (8097; 93), 1138 (8791; 106), 1079 (9264; 122), 1031 (9699; 121), 985 (10,152; 111). A broad feature extends from ∼715 nm (14,000 cm^–1^) into the UV region and beyond our spectral range.

FT-IR (ATR, microcrystalline) cm^–1^: 2959 (m), 2922 (m), 2867 (m), 2527 (w), 2466 (w), 2410 (w), 2361 (w), 2170 (m), 2142 (m), 2123 (w), 2094 (w), 2092 (w), 2043 (w), 1605 (w), 1566 (w), 1459 (m), 1383 (m), 1358 (m), 1301 (w), 1204 (s), 1169 (s), 1102 (s), 1069 (m), 1042 (m), 939 (m), 884 (m), 849 (w), 799 (m), 774 (m), 746 (m), 729 (m), 652 (w), 600 (w), 547 (w), 524 (w), 501 (w), 462 (s), 448 (s), 421 (s).

#### Synthesis of [U^III^(H_3_B·SAr^*i*Pr6^-κS,H,H)(BH_4_)_2_] (**3**)

Method A. Toluene (10 mL) was added to a precooled (−78 °C) stirring mixture of solid [U^IV^(BH_4_)_4_] (0.149 g, 0.50 mmol) and HSAr^*i*Pr6^ (0.257 g, 0.50 mmol, 1 equiv) in a glass Rotaflo-appended vessel equipped with a PTFE-coated stirrer bar. The pale-black solution was allowed to warm to room temperature followed by heating to 110 °C open to the argon manifold. After reaching 110 °C, the intense brown/black solution was stirred open to the argon manifold for 2 h. The reaction was allowed to cool, and the mixture was concentrated under vacuum (10^–3^ mbar) until the point of incipient crystallization. The solids were redissolved by boiling the solution; then, the vessel was placed in an oil bath at 60 °C. The black solution was filtered while warm through a glass microfiber filter disc, which revealed a small crop of black crystalline material, which remained in the vessel and was determined to be [U^III^(H_3_B·SAr^*i*Pr6^-κ*S*,*H*,*H*)(BH_4_)_2_] (**3**) by SC-XRD. A second crop of **3** was obtained at −30 °C (combined yield = 0.274 g, 0.34 mmol, 69%).

Method B. In a manner identical to Method A with [U^IV^(BH_4_)_4_] (0.149 g, 0.50 mmol) and HSAr^*i*Pr6^ (0.514 g, 1.00 mmol, 2 equiv), a black crystalline material of **3** was isolated after 24 h at 5 °C (yield = 0.326 g, 0.41 mmol, 82%).

Elemental analysis on C_36_H_60_B_3_SU·(C_7_H_8_) calcd (%): C, 58.19; H, 7.72; N, 0.00. Found (%): C, 58.40; H, 8.05; N, 0.00; note that PXRD suggests the presence of an additional crystalline phase across independently synthesized batches of **3** which could not be identified by SC-XRD, but that prolonged drying of the crystalline material did not alter the quantity of toluene present as determined by ^1^H NMR experiments with variable D1 delays (1–10 s).

^1^H NMR (*d*_6_-benzene, 400.07 MHz, 298 K): δ 88.58 (br s,  = 324 Hz), 8.49 (d, *J* = 7.9 Hz), 8.02 (s), 7.44 (s), 7.29 (t, *J* = 7.6 Hz), 6.43 (br s,  = 17 Hz), 5.30 (br s,  = 17 Hz), 4.94 (br s,  = 17 Hz), 4.22 (d, *J* = 7.5 Hz), 3.83 (d, *J* = 6.4 Hz), 3.75 (br s,  = 14 Hz), 2.92 (sept, ^3^*J*_HH_ = 6.8 Hz, 4H, Tripp-2,6-C*H*(CH_3_)_2_), 1.94 (br s,  = 14 Hz), 1.79 (d, ^3^*J*_HH_ = 6.5 Hz, 12H, Tripp-4–CH(C*H*_3_)_2_), 1.27 (d, ^3^*J*_HH_ = 6.8 Hz, 12H, Tripp-2,6–CH(C*H*_3_)_2_), 1.22 (d, ^3^*J*_HH_ = 6.9 Hz, 12H, Tripp-2,6–CH(C*H*_3_)_2_), 0.93 (br s,  = 22 Hz), −1.32 (s), −3.32 (s), −4.20 (s), −4.68 (s), −5.23 (s), −9.01 (s), −22.62 (br s,  = 15 Hz), −22.78 (br s,  = 23 Hz).

^11^B NMR (*d*_6_-benzene, 128.36 MHz, 298 K): δ 102.9 (br s,  = 217 Hz), 75.2 (br s,  = 473 Hz), 59.0 (br s,  = 242 Hz, {κ^2^-H_3_*B*·SAr^*i*Pr6^}).

UV–Vis–NIR (Et_2_O): λ_max_ (cm^–1^; ε) 1217 (8220; 40), 1133 (8830; 53), 1005 (9951; 58), 1003 (9970; 63), 902 (11,087; 85), 825 (12,121; 144), 617 (16,195; 435), 552 (18,115; 585), 502 (19,940; 758), 476 (21,000; 870), 437 (22,880; 950).

FT-IR (ATR, microcrystalline) cm^–1^: 2961 (s), 2929 (m), 2869 (m), 2523 (w), 2479 (m), 2449 (m), 2183 (m), 2127 (m), 1605 (w), 1570 (w), 1459 (s), 1389 (m), 1367 (m), 1315 (m), 1190 (vs), 1169 (vs), 1102 (m), 1077 (s), 1041 (m), 938 (m), 894 (m), 873 (m), 801 (s), 729 (s), 694 (m), 651 (w), 583 (m), 519 (vw), 462 (m).

#### Synthesis of [U^III^(SAr^*i*Pr6^)(BH_4_)_2_] (**4a**) and [{U^III^(SAr^*i*Pr6^)(BH_4_)}_2_(μ-B_2_H_6_)] (**4b**)

Et_2_O (10 mL) was added to a precooled (−98 °C) mixture of solid KSAr^*i*Pr6^ (0.553 g, 1.00 mmol, 1 equiv) and [U^III^(BH_4_)_3_(toluene)] (1 equiv) and a PTFE-coated stirrer bar. The mixture was allowed to warm to room temperature, which gave a dark-red/brown solution and pale solids. This was stirred at room temperature for 18 h, after which the volatiles were removed under vacuum (10^–3^ mbar). The dark-red solid was extracted in warm hexane (20 mL) and filtered through a glass microfiber filter disc. The dark-brown supernatant was concentrated to ca. 6 mL, at which point a significant quantity of solid precipitated. The solids were redissolved by heating, the clear red/brown hexane solution was stored at 5 °C for 8 h and then at −30 °C for 18 h, and then a crop of dark-red crystals was isolated (yield = 0.084 g) from which the structure of [U^III^(SAr^*i*Pr6^)(BH_4_)_2_] (**4a**) was determined. The supernatant was dried under vacuum (10^–3^ mbar), and an attempt was made to obtain a second crop of crystals using toluene as the solvent, but this was unsuccessful. The solids were re-extracted into boiling hexane (10 mL), which was allowed to cool to room temperature. Concentration of this mixture and storage at −30 °C gave dark-red crystals of [{U^III^(SAr^*i*Pr6^)(BH_4_)}_2_(μ-B_2_H_6_)] (**4b**). The ratio of **4a** to **4b** in the crystalline batches could not be accurately determined. Total yield: 0.286 g, 37% based on [U^IV^(BH_4_)_4_] used.

Elemental analysis on C_36_H_57_B_2_SU (**4a**) calcd (%): C = 55.32, H = 7.35, N = 0.00; found (%): C = 55.25, H = 7.50, N = 0.00. Elemental analysis on C_72_H_112_B_4_S_2_U_2_ (**4b**) calc. (%): C = 55.40, H = 7.23, N = 0.00; found (%): C = 55.09, H = 7.58, N = 0.00. Note that the composition per uranium for **4a** and **4b** differs by just H_2_, which would be within the error of an elemental analysis experiment. Thus, reported data are given from the separate crystalline crops from which the SC-XRD data is derived, but each batch of material likely contains both complexes.

Spectroscopic data for the two crystalline batches of **4a** and **4b** were found to be superposable; thus, only **4b** is given below:

^1^H NMR (*d*_6_-benzene, 400.07 MHz, 298 K): δ 8.48 (d, *J* = 7.6 Hz), 8.01 (s), 7.44 (s), 6.24 (br s,  = 24 Hz), 5.28 (br s,  = 30 Hz), 4.92 (br s,  = 26 Hz), 4.28 (d, *J* = 6.1 Hz), 3.79 (br s,  = 14 Hz), 3.68 (br s,  = 18 Hz), 2.92 (br s,  = 31 Hz), 1.94 (s), 1.79 (s), −1.27 (s), −3.21 (s), −4.09 (s), −4.57 (s), −5.14 (s), −8.71 (s), −22.12 (br s,  = 30 Hz), −22.39 (br s,  = 36 Hz).

^11^B NMR (*d*_6_-benzene, 128.36 MHz, 298 K): δ 102.9 (br s,  = 278 Hz), 75.4 (br s,  = 430 Hz).

UV–Vis–NIR (Et_2_O): λ_max_ (cm^–1^; ε) 1291 (7745; 36), 1217 (8220; 40), 1133 (8830; 57), 1005 (9951; 66), 902 (11,087; 83), 825 (12,121; 146), 617 (16,195; 500), 552 (18,115; 700), 502 (19,940; 941), 476 (21,000; 1093), 437 (22,880; 1218).

FT-IR (ATR, microcrystalline) cm^–1^: 3049 (vw), 2959 (s), 2927 (m), 2867 (m), 2570 (vw), 2479 (m), 2452 (m), 2281 (m), 2188 (m), 2129 (m), 1605 (w), 1568 (w), 1459 (m), 1389 (m), 1362 (m), 1315 (m), 1235 (w), 1169 (vs), 1102 (m), 1081 (m), 935 (m), 894 (m), 875 (m), 799 (m), 746 (m), 729 (m), 694 (w), 651 (w), 585 (w), 522 (w), 464 (w).

#### Synthesis of [U^III^(SAr^*i*Pr6^)_2_(BH_4_)] (**5**)

Solid KSAr^*i*Pr6^ (1.106 g, 2.00 mmol, 2 equiv) was added to solid [U^III^(BH_4_)_3_(toluene)] (1 equiv) along with a PTFE-coated stirrer bar, and the vessel was cooled to −78 °C. Et_2_O (20 mL) was added, and the reaction was warmed to room temperature. The dark-red/brown solution was stirred at room temperature for 18 h, after which the volatiles were removed under vacuum (10^–3^ mbar). The dark-red solid was extracted in boiling hexane (15 mL) and filtered through a glass microfiber filter disc. Cooling to room temperature followed by sequential storage at 5 °C for 8 h followed by −30 °C for 48 h gave dark-red crystals of [U^III^(SAr^*i*Pr6^)_2_(BH_4_)] (**5**) over two crystalline crops (combined yield = 0.660 g, 0.52 mmol, 52%).

Elemental analysis on C_72_H_102_BS_2_U·(C_6_H_14_)_0.5_ calcd (%): C, 68.06; H, 8.30; N, 0.00. Found (%): C, 68.20; H, 8.12; N, 0.00. Voids were found in the structure of **5** from the SC-XRD study, although we could not resolve significant density within them. The presence of *n*-hexane, the solvent of crystallization, was determined from ^1^H NMR spectroscopy, though due to the paramagnetic nature of **5**, it is not appropriate to use integration to determine the relative quantity of these two species. PXRD was used in addition to the elemental analysis data to confirm the bulk purity of **5**.

^1^H NMR (*d*_6_-benzene, 400.07 MHz, 298 K): δ 126.58 (br s,  = 455 Hz, 4H, B*H*_4_), 6.70 (br s,  = 47 Hz, 4H), 5.02 (br s,  = 164 Hz, 4H), 3.04–4.26 (br. m, 46H), 2.79 (br s,  = 44 Hz, 8H), −0.25 to −0.42 (br. m, 30H), −3.95 (br s,  = 264 Hz, 4H), −4.68 (br s,  = 273 Hz, 2H).

^11^B NMR (*d*_6_-benzene, 128.36 MHz, 298 K): δ 177.8 (br s,  = 344 Hz).

UV–Vis–NIR (Et_2_O): λ_max_ (cm^–1^; ε) 1283 (7797; 54), 1215 (8230; 69), 1175 (8514; 73), 1165 (8587; 73), 1140 (8772; 81), 1088 (9195; 82), 998 (10,020; 85), 967 (10,341; 79), 890 (11,236; 114), 871 (11,481; 107), 843 (11,862; 121), 823 (12,158; 119), 617 (16,195; 550), 488 (22,512; 1333).

FT-IR (ATR, microcrystalline) cm^–1^: 3045 (vw), 2957 (vs), 2927 (m), 2865 (m), 2478 (w), 2431 (w), 2137 (m), 1607 (w), 1568 (w), 1539 (vw), 1459 (s), 1385 (s), 1360 (m), 1317 (m), 1237 (m), 1194 (m), 1169 (m), 1126 (m), 1109 (m), 1044 (m), 939 (m), 898 (m), 873 (m), 793 (m), 744 (s), 743 (s), 651 (m), 606 (w), 586 (w), 522 (w), 450 (m), 423 (s).

## Data Availability

A preprint of this article was previously deposited on ChemRxiv.^142^ Research data files supporting this publication are available from FigShare at DOI: 10.48420/26321476.

## Supporting Information Available

Details of the starting materials used, additional details for the synthesis of complexes herein, descriptions of sample preparation for spectroscopic measurements, and methodologies for electronic structure calculations (PDF)
