# Supplementary material for: Uranium(III) and Uranium(IV) meta-Terphenylthiolate Complexes
Source: Inorg Chem. 2025 Feb 7;64(7):3161–77. doi: 10.1021/acs.inorgchem.4c03085 (PMC11863384; doi:10.1021/acs.inorgchem.4c03085)
Supplement: Supplementary file 1 — ic4c03085_si_001.pdf [file ic4c03085_si_001.pdf]

Supporting Information for:

Uranium(III) and uranium(IV) *meta*-terphenylthiolate complexes

Benjamin L. L. Réant,[1,2] John A. Seed,[1,2] George F. S. Whitehead,[2] and Conrad A. P. Goodwin*[1,2]

[1] Centre for Radiochemistry Research, The University of Manchester, Oxford Road, Manchester, M13 9PL (UK).

[2] Department of Chemistry, The University of Manchester, Oxford Road, Manchester, M13 9PL (UK).

*Correspondence: *conrad.goodwin@manchester.ac.uk*

Table of Contents

S1. Experimental Details 3
Equipment, materials, and solvents 3
A note on NMR spectroscopy of paramagnetic samples 4
Notes on the synthesis of $[U^{IV}(BH_4)_4]_n$ 6
Ball milling $U^{IV}Cl_4$ + 4 $LiBH_4$ to give $[U^{IV}(BH_4)_4]$ 8
Synthesis of $[U^{III}(BH_4)_3(Toluene)]$ 11
S2. Crystallography 12
General considerations for single crystal X-ray diffraction (SC-XRD) studies 12
Powder X-ray diffraction (PXRD) studies 19
S3. Molecular Structures 25
S4. NMR Spectroscopy Plots 33
Magnetic moments determined by NMR spectroscopy (Evans method) 43
S5. ATR-IR Spectra 44
S6. UV-Vis-NIR Spectra 50
S7. SQUID Magnetometry 54
S8. Density Functional Theory calculations 61
Density Functional Theory – General considerations 61
Molecular orbital compositions and isosurfaces 63
Coordinates 71
S9. References 93

S1. Experimental Details

Equipment, materials, and solvents

Caution! The natural-abundance U (assumed standard composition: 0.7204% ^{235}U, $t_{½}$ = 7.04×10^{8}; 99.2742% ^{238}U, $t_{½}$ = 4.468×10^{9}) used in this work, along with the α-, β-, and γ-emitting decay products represent radiotoxicity (α-particles) and heavy-metal health hazards. Hence, all manipulation of these materials was performed in continuous extraction fume cupboards or positive pressure inert atmosphere gloveboxes located in a dedicated laboratory with contamination monitoring protocols, along with training and materials for decontamination equipment. Additional safeguards include the use of hand-held radiation monitoring equipment.

Unless otherwise described, all syntheses and manipulations were conducted under BOC PureShield argon (99.995%) with rigorous exclusion of oxygen and water using Schlenk line and glove box techniques in an MBraun Lab Star™. 3 Å molecular sieves were activated by heating for 8 hours at 300°C, 10^{-3} mbar. THF, *n*-hexane, *n*-pentane, Et_2O, and toluene were degassed by sparging (N_2) and dried by passage through neutral alumina columns (INERT Corp.). THF was then degassed under vacuum and stored over 3 Å molecular sieves for 7 days before use. *n*-hexane, *n*-pentane, Et_2O, and toluene were degassed under vacuum, stored over a K mirror and used immediately. *d*$_6$-benzene (Merck) was dried by refluxing over K metal for 4-5 days then vacuum transferred to a J. Youngs valve appended vessel. $U^{IV}Cl_4$ and [$U^{IV}(BH_4)_4$] were prepared as described previously,[1,2] and we also report additional details for the preparation of [$U^{IV}(BH_4)_4$] below. KSAr^{iPr6} was prepared from HSAr^{iPr6} and K^0 in toluene.[3,4] Batches of KSAr^{iPr6} usually contained between 0.03 and 0.33 molecules of lattice toluene by ^{1}H NMR spectroscopy, which depended on the length of drying. This was accounted for in the reaction stoichiometries and stated masses are given for the KSAr^{iPr6} content. Exemplar ^{1}H and ^{13}C{^{1}H} NMR spectra of KSAr^{iPr6} are provided in **Figure S1** and **Figure S2**. Glass-fiber filter discs and PTFE-coated stirrer bars were stored in an oven (150°C) for at least 12 hours before use, and glassware was dried under vacuum (10^{-3} mbar) after strong heating with a butane flame. Solution phase UV-Vis-NIR spectra were collected at

ambient temperature using a PerkinElmer Lambda 1050 UV-Vis-NIR spectrometer. Solutions were contained in a low volume (1 mL) screw-capped quartz cuvette (10 × 4 mm path length fluorescence cell), or a 1 mm path length absorbance cell. ATR FT-IR spectra of microcrystalline samples were collected using a Bruker ALPHA II FT-IR spectrometer equipped with a Platinum ATR module with a diamond window. NMR spectroscopic data collection was performed on either a Bruker Avance III (400 MHz), Bruker Advance III HD (400 MHz), or Bruker Ascend (700 MHz) between 295 K to 299 K. Elemental microanalyses (C/H/N) were carried out by Martin Jennings and Anne Davies at the University of Manchester.

A note on NMR spectroscopy of paramagnetic samples

All spectra were referenced to internal solvent residuals (^{1}H and ^{13}C) or externally to 10% TMS in d_3-chloroform *via* Equation S1, which is the IUPAC recommended convention.

Equation S1.

$$\Delta\ (Hz) = \frac{SR^{1H}}{SF^{1H}} \times SF^{NUC}$$

Where SR^{1H} is the spectrum reference frequency (in Hz) of a reference ^{1}H NMR spectrum collected with TMS set to 0 ppm collected under the same experimental conditions; SF^{1H} is the spectrometer frequency (in MHz) for the ^{1}H nucleus; SF^{NUC} is the spectrometer frequency (in MHz) of the nucleus in question. The answer is given in Hz.

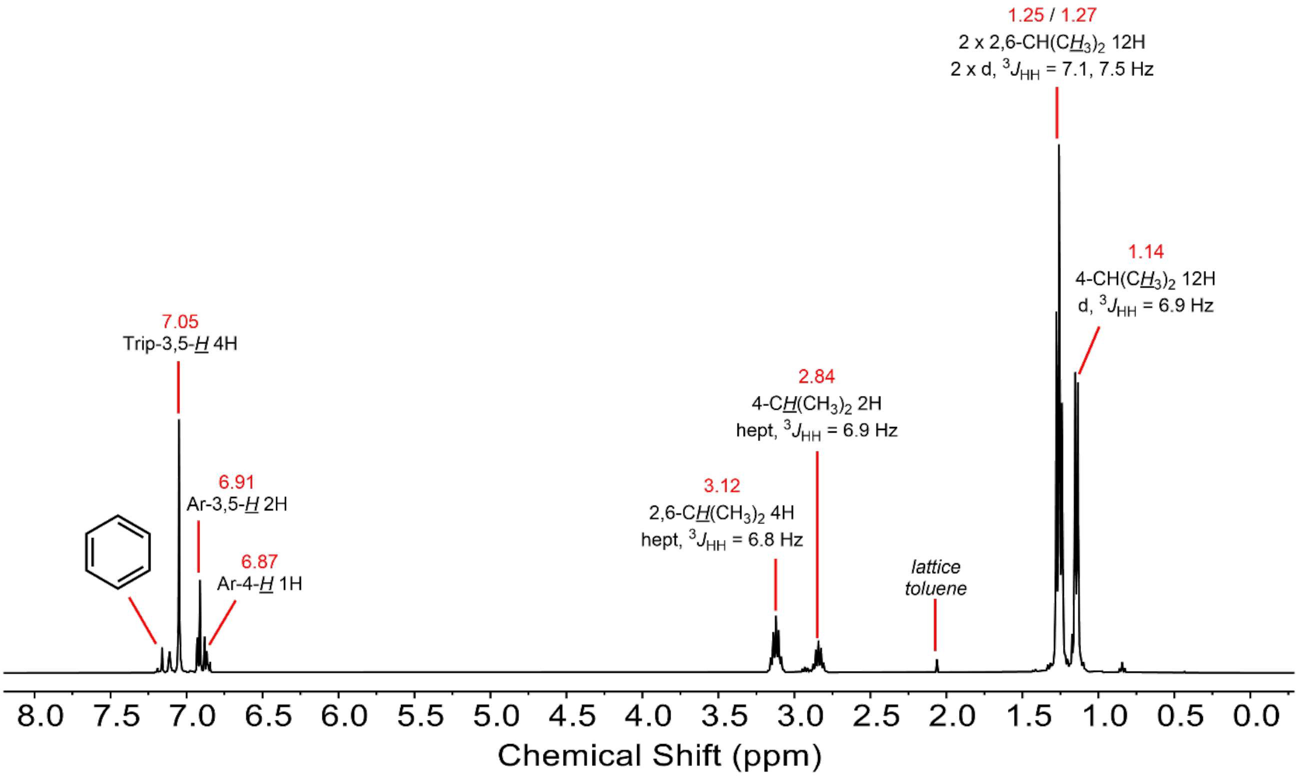

Figure S1. ^{1}H NMR spectrum of KSAr^{iPr6} in d_6-benzene.

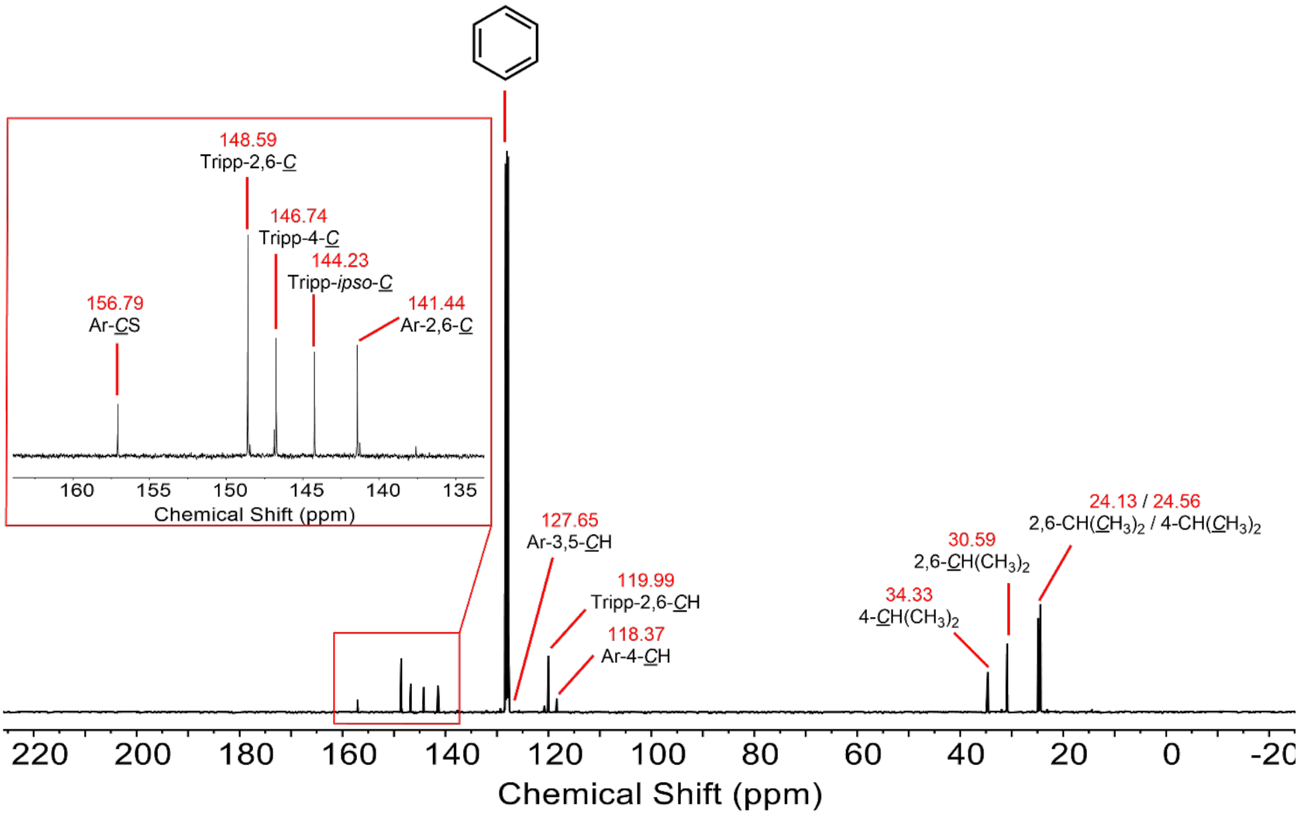

Figure S2. ^{13}C{^{1}H} NMR spectrum of KSAr^{iPr6} in d_6-benzene.

Table S1. Compound numbering, formula, reaction scale (by metal quantity used), yield, and then % yield where appropriate.

	Scale (mmol metal)	Formula	Yield (g)	% yield
**1**	0.50	$[U^{IV}(SAr^{iPr6})_2(Cl)_2]$	0.132	20
**1·Et₂O**	0.50	$[U^{IV}(SAr^{iPr6})_2(Cl)_2(OEt_2)_2]$	–	<1
**2**	0.50 (Method A)	$[U^{IV}(\mu\text{-}SAr^{iPr6})(BH_4)_2(\mu\text{-}BH_4)(\mu^3\text{-}BH_4)K]_2$	0.022	3
	0.50 (Method B)		0.113	26
**3**	0.50 (Method A)	$[U^{III}(H_3B\cdot SAr^{iPr6}\text{-}\kappa S,H,H)(BH_4)_2]$	0.274	69
	1.00 (Method B)		0.326	82
**4a** + **4b**	1.00	$[U^{III}(SAr^{iPr6})(BH_4)_2]$ + $[\{U^{III}(SAr^{iPr6})(BH_4)\}_2(\mu\text{-}B_2H_6)]$	0.084 + 0.202	A
**5**	1.00	$[U^{III}(SAr^{iPr6})_2(BH_4)]$	0.660	52

Note: Yields are representative of a single iteration only, and are intended only to indicate what might be expected from these unoptimized reactions. [A] The ratio of complex **4a** to **4b** in each crystalline batch is unknown, therefore it is not appropriate to provide a yield.

Notes on the synthesis of $[U^{IV}(BH_4)_4]_n$

Daly recently reported the convenient synthesis of $[U^{IV}(BH_4)_4]_n$ by ball-milling $U^{IV}Cl_4$ and $LiBH_4$.[1] This is a useful precursor as (i) the synthesis does not require U-metal, unlike $[UI_4(OEt_2)_2]$;[5,6] (ii) the $\{BH_4\}$ group is synthetically versatile;[7-14] and, (iii) careful thermolysis of $[U^{IV}(BH_4)_4]_n$ gives U(III)-borohydrides (**Scheme 1A**) which Ephritikhine has shown can be isolated as U(III) arene adducts such as $[U^{III}(BH_4)_3(toluene)]$, therefore allowing the same material to be employed in both U(IV) and U(III) chemistry.[15]

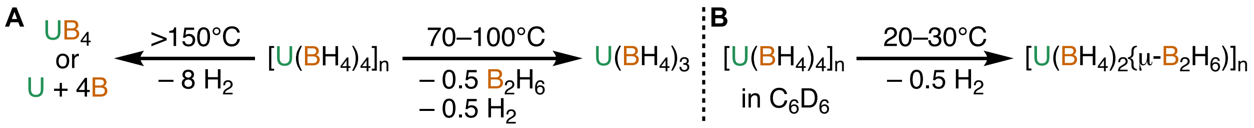

Scheme 1. (A + B) Different pathways for the thermolysis of $[U^{IV}(BH_4)_4]_n$ depending on the reaction conditions.

Two different crystalline phases of $[U^{IV}(BH_4)_4]_n$ can be obtained depending on the temperature of the condensing surface during the sublimation step.[16-20] $[U^{IV}(BH_4)_4]_n$-α forms when the condensation

surface is *ca*. 20°C, has rather poor solubility in ethereal solvents (<20 g·L^{-1} in Et_2O),[21] and produces large dark green/black block-like crystals on the surface of the sublimator (see the **Figure S3** for images). $[U^{IV}(BH_4)_4]_n$-β forms when the condensing surface is at –80°C, has a much higher solubility in organic solvents (up to 44 g·L^{-1} in benzene),[19,20] and takes on a paler green/brown color. The crystal structures of both phases have previously been reported and key parameters are shown in **Table S2**.[17-20]

Table S2. Crystallographic data for the α and β crystalline phases of $[U^{IV}(BH_4)_4]$.

	$[U^{IV}(BH_4)_4]_n$-α	$[U^{IV}(BH_4)_4]_n$-β
CCDC or ICSD Identification code	ICSD 10339 FUZNUJ01	ICSD 63132
Temperature / K	295	295
Crystal system	Tetragonal	Orthorhombic
Space group	$P4_32_12$	*C*mcm
a / Å	7.49(1)	8.014(2)
b / Å	7.49(1)	7.940(3)
c / Å	13.24(1)	11.673(3)
α / °	90	90
β / °	90	90
γ / °	90	90
Volume / Å^3	742.77(1)	742.7(7)
Z	4	4
ρ_{calcg} / g cm^{-3}	2.66	2.66

Herein, we have used both phases without a discernible difference in their reactivities. When a portion of dark green/black crystalline $[U^{IV}(BH_4)_4]_n$-α was left standing in *d*$_6$-benzene at room temperature for 7 days, several dark red crystals deposited and were shown to be $[U^{IV}(BH_4)_2\{\mu\text{-}B_2H_6\}]_n$ by SC-XRD (**Scheme 1B**, and see *Supporting Information* for the molecular structure). No further analysis could be obtained on this material, which was consumed during the SC-XRD study. However, subsequent examination of $[U^{IV}(BH_4)_4]_n$-β in *d*$_6$-benzene (δ_H = 134.84, br. singlet, $h_{1/2}$ = 556 Hz; δ_B = 130.86, quintet $^1J_{BH}$ = 87.0 Hz)[1,17,18,22] by ^{1}H NMR spectroscopy shows that it slowly

liberates H_2 (as evidenced by a peak at δ_H = 4.47 ppm) at room temperature, and this is accompanied by the growth of a broad singlet at δ_B = 31.10, and a complex feature at δ_B = 17.29 which we suggest is due to the formation of a small quantity of $[U^{IV}(BH_4)_2\{\mu\text{-}B_2H_6\}]_n$.

Ball milling $U^{IV}Cl_4$ + 4 $LiBH_4$ to give $[U^{IV}(BH_4)_4]$

Two 25 mL Retsch stainless grinding tubes each containing three small stainless steel balls (diameter = 9 mm), UCl_4 (1.500 g, 3.95 mmol) and $LiBH_4$ (0.344 g, 15.80 mmol, 4 equiv.) were prepared in the glovebox. The sealed grinding tubes were fixed in position into the Retsch Mixer Mill 400, where the ball milling procedure outlined in **Figure S3** was applied. Afterwards, the combined beige-green solid was sublimed at 50 °C (6 x 10^{-3} mbar) for 2 hours to afford a lime green solid of $[U^{IV}(BH_4)_4]_n$ (1.260 g, 4.24 mmol, 54%).

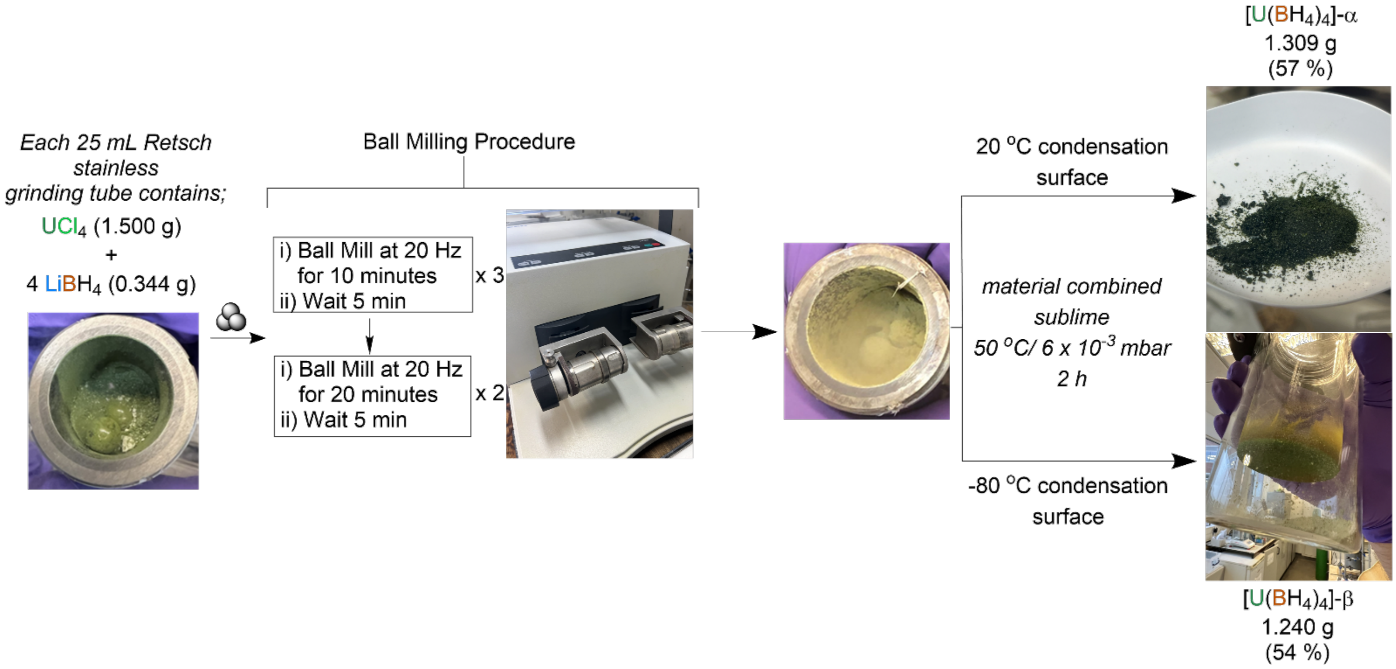

Figure S3. Synthesis of $[U^{IV}(BH_4)_4]_n$-α/β by ball milling $U^{IV}Cl_4$ + 4 $LiBH_4$.

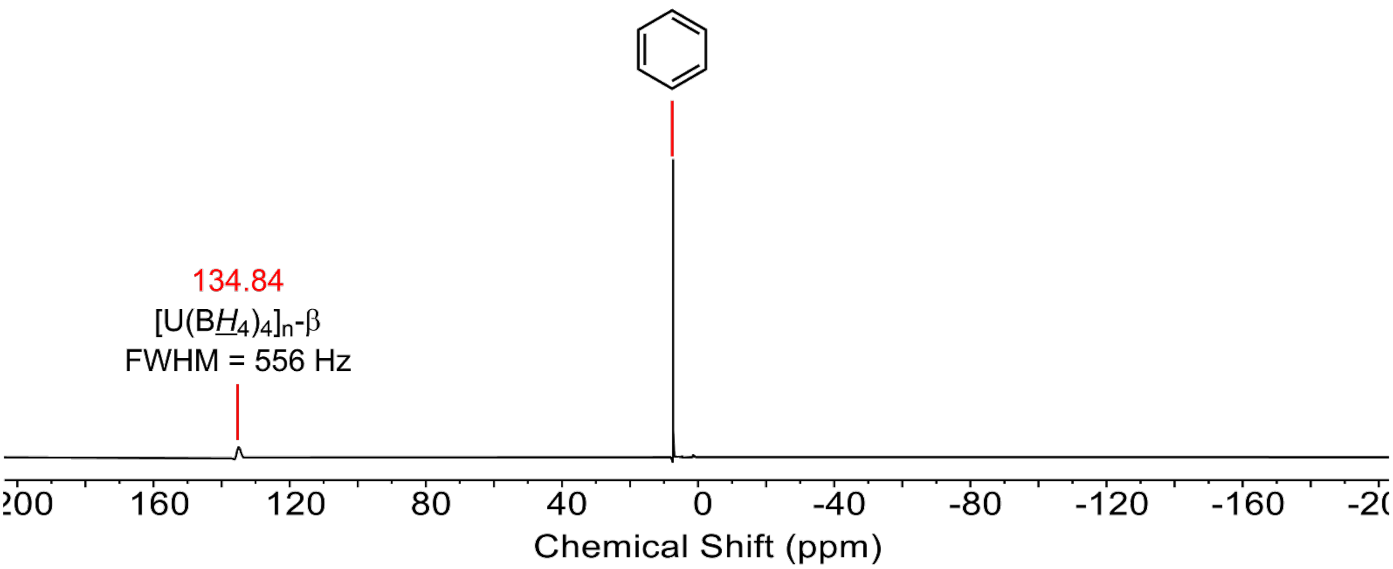

Figure S4. ^{1}H NMR spectrum of $[U^{IV}(BH_4)_4]_n$ in d_6-benzene.

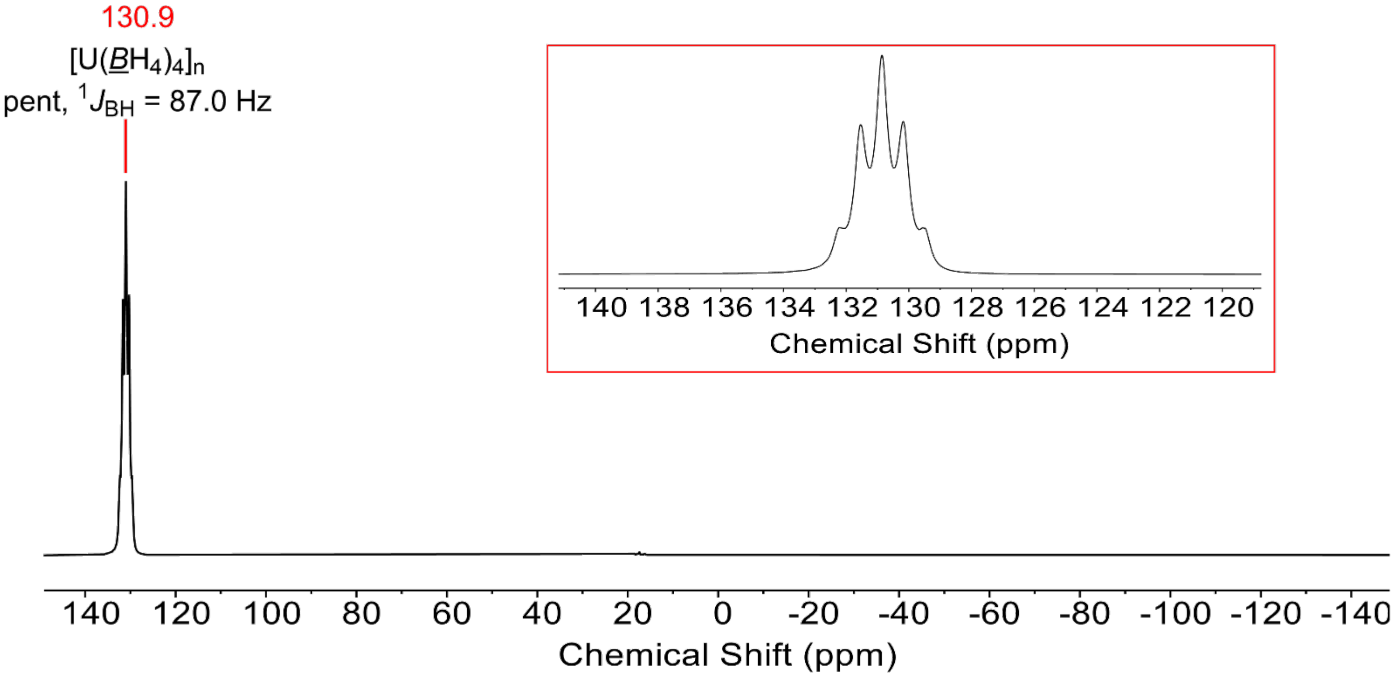

Figure S5. ^{11}B NMR spectrum of $[U^{IV}(BH_4)_4]_n$ in d_6-benzene.

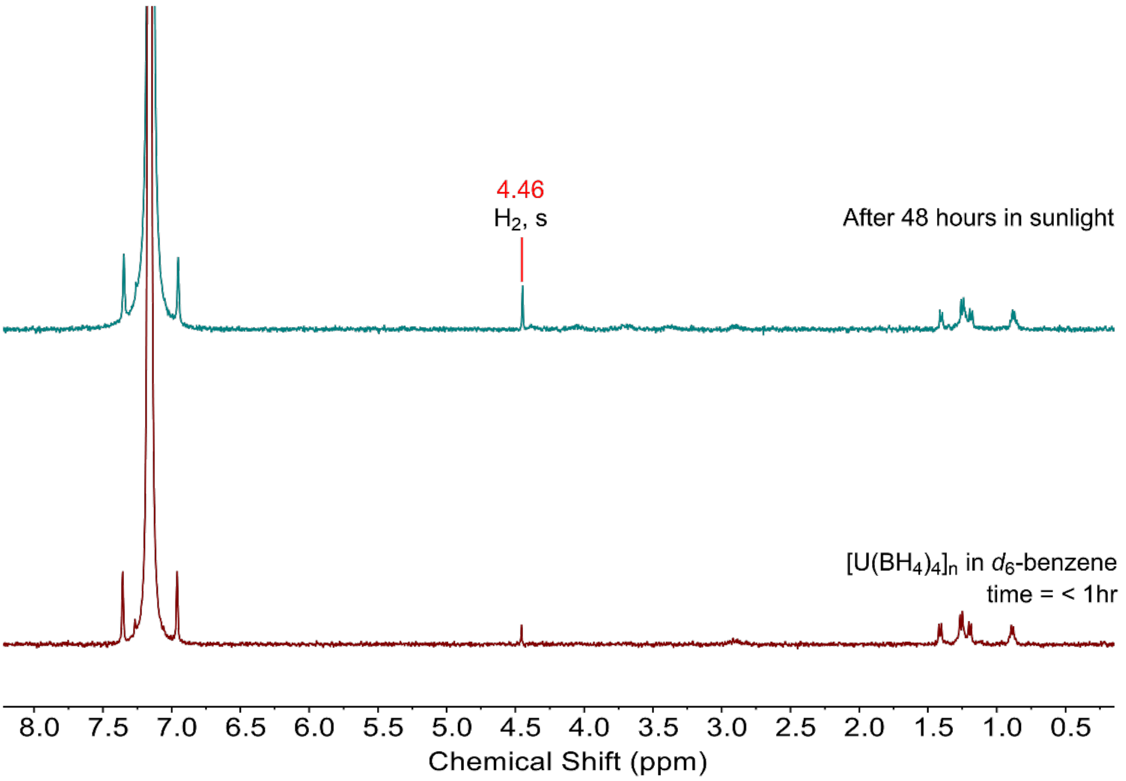

Figure S6. Stacked and zoomed in ^{1}H NMR spectrum of $[U^{IV}(BH_4)_4]_n$ in d_6-benzene, depicting the evolution of dihydrogen within 1 hour (bottom) and after 48 hours (top) with exposure to sunlight.

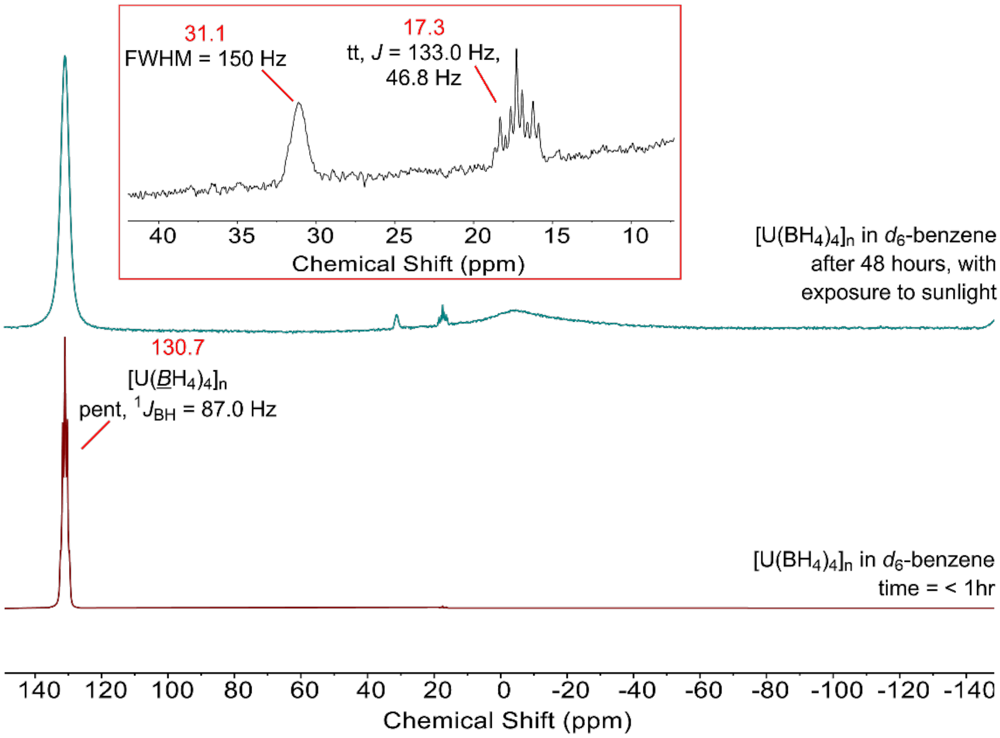

Figure S7. Stacked ^{11}B NMR spectrum of $[U^{IV}(BH_4)_4]_n$ in d_6-benzene, depicting the evolution of H_2 within 1 hour (bottom) and after 48 hours (top) with exposure to sunlight. Inset showing the zoomed in region of the top spectrum.

Synthesis of $[U^{III}(BH_4)_3(Toluene)]$

Several of the proceeding syntheses use $[U^{III}(BH_4)_3(Toluene)]$, which is isolated as a crude powder without further purification. An example synthetic procedure is given below.

Toluene (10 mL) was added to a glass Rotaflo-appended vessel containing $[U^{IV}(BH_4)_4]_n$ (0.297 g, 1.00 mmol) and a PTFE-coated stirrer bar. The light brown/green solution was heated to 120°C open to the argon manifold. After reaching 120°C, the solution rapidly became dark brown in color and stirring was continued open to the argon manifold for 5 minutes before the vessel was sealed and stirring was continued at 120°C. The vessel was periodically vented to the argon manifold to avoid overpressurization. After 30 minutes, the vessel cooled to *ca.* 50°C, and the volatiles were removed under vacuum (10^{-3} mbar) furnishing a dark red powder, presumed to be $[U^{III}(BH_4)_3(toluene)]$,[15] in quantitative yield. This was used without purification.

S2. Crystallography

General considerations for single crystal X-ray diffraction (SC-XRD) studies

Data for **1**, **2**, **3**, **4a**, **4b**, **5**, and $[U(BH_4)_2(\mu\text{-}B_2H_6)]_n$ were collected using a Rigaku XtaLAB synergy DW diffractometer, equipped with a PhotonJet Cu Kα radiation source (λ = 1.54184 Å), using a 4-circle κ goniometer, a HyPix-6000HE hybrid pixel array detector operating in shutterless mode and an Oxford Cryosystems Cryostream 800 nitrogen flow gas system at a temperature of 100K. Data for **1·Et₂O** and $(SAr^{iPr6})_2$ were collected using a Rigaku FR-X DW diffractometer, equipped with an FR-X high-intensity rotating anode Cu Kα radiation source (λ = 1.54184 Å) and VariMAXTM microfocus optics, using an AFC-11 4-circle κ goniometer, a HyPix-6000HE hybrid pixel array detector operating in shutterless mode and an Oxford Cryosystems Cryostream 800 nitrogen flow gas system at a temperature of 100K. Intensities were integrated from data recorded from ω, or ω and φ rotation at the frame width and exposure times outlined in **Table S3**. The crystal data for all complexes is outlined in **Table S4** to **Table S7**.

Table S3. Data collection parameters and CCDC reference codes for all structures herein.

	Formula	Frame width (°)	Exposure time (s)	CCDC ref.
**1**	$[U^{IV}(SAr^{iPr6})_2(Cl)_2]$	1.00	45.00	2352695
**1·Et$_2$O**	$[U^{IV}(SAr^{iPr6})_2(Cl)_2(Et_2O)_2]$	0.50	0.50, 1.20	2352696
**2**	$[U^{IV}(\mu\text{-}SAr^{iPr6})(BH_4)_2(\mu\text{-}BH_4)(\mu^3\text{-}BH_4)K]_2$	0.50	0.50, 1.50	2352697
**3**	$[U^{III}(H_3B{\cdot}SAr^{iPr6}\text{-}\kappa S,H,H)(BH_4)_2]$	0.50	2.00, 5.00	2352698
**4a**	$[U^{III}(SAr^{iPr6})(BH_4)_2]$	0.50	10.00, 40.00	2352699
**4b**	$[\{U^{III}(SAr^{iPr6})(BH_4)\}_2(\mu\text{-}B_2H_6)]$	0.50	2.00, 7.00	2352700
**5**	$[U^{III}(SAr^{iPr6})_2(BH_4)]$	0.50	2.00, 6.00	2352701
–	$[U(BH_4)_2(\mu\text{-}B_2H_6)]_n$	0.50	0.13, 0.53	2352702
–	$(SAr^{iPr6})_2$	0.50	0.50	2352703

CrysAlisPro[23] was used for final unit cell determination and parameters were refined from the observed positions of all strong reflections in each data set. An analytical absorption correction was applied.[23] The Olex2[24] GUI was used for structure solution and refinement utilizing the ShelX software packages.[25,26] The structures were solved using ShelXT[25]; the datasets were refined by

ShelXL[26] using full-matrix least-squares on all unique F^2 values, with anisotropic displacement parameters for all non-hydrogen atoms, and with constrained riding hydrogen geometries; U_{iso}(H) was set at 1.2 (1.5 for methyl groups if applicable) times Ueq of the parent atom. The largest features in final difference syntheses were close to heavy atoms and were of no chemical significance. Olex2[24] combined with POV-Ray,[27] Gimp,[28] and InkScape[29] were employed for molecular graphics. The following CCDC references contain the supplementary crystal data for this article: **1** (2352695), **1·Et$_2$O** (2352696), **2** (2352697), **3** (2352698), **4a** (2352699), **4b** (2352700), **5** (2352701), $[U^{IV}(BH_4)_2\{\mu\text{-}B_2H_6\}]_n$ (2352702), and $(SAr^{iPr6})_2$ (2352703). These data can be obtained free of charge from the Cambridge Crystallographic Data Centre via www.ccdc.cam.ac.uk/data_request/cif.

The combined error from two individual metrics that have their own associated errors (estimated standard deviation, or standard uncertainty used interchangeably here) can be calculated as the root of the sum of the square of each error (**Equation S2**). This is not strictly appropriate for combining more than two individual errors.[30]

Equation S2. $$\sigma_{tot} = \sqrt{\sigma_1{}^2 + \sigma_2{}^2}$$

The combined error for the numerical average for multiple (independent) bond lengths, such as the five independent M–C bonds in an M–($\eta^5C_5H_5$) complex, is calculated using the alternate weighted standard deviation from Parsons and Clegg (**Equation S3**).[30]

Equation S3. $$\sigma_{tot} = 1/\sqrt{\textstyle\sum_{1\rightarrow n} W_n} \qquad W_n = 1/\sigma_n{}^2$$

For the analysis of octahedral distortions in **1·Et$_2$O**, the following definitions are used and were calculated using OctaDist:[31]

$$\zeta = \sum_{i=1}^{6} |d_i - d_{mean}|$$

Equation S3. ζ parameter, where d_i individual M–X bond distances and d_{mean} is mean M–X bond distance.[32]

$$\Delta = \frac{1}{6}\sum_{i=1}^{6}\left(\frac{d_i - d_{mean}}{d_{mean}}\right)^2$$

Equation S4. Δ parameter, where d_i individual M–X bond distances and d_{mean} is mean M–X bond distance.[33]

$$\Sigma = \sum_{i=1}^{12} |90 - \phi_i|$$

Equation S5. Σ parameter, where φ_i is the 12 individual X–M–X angles.[34]

$$\Theta = \sum_{i=1}^{24} |60 - \theta_i|$$

Equation S6. Θ parameter, where θ_i is the 24 individual $X_3 \cdots X_3$ face twisting angles.[35]

Both Σ and Θ would be 0 for a perfect octahedron (all angles would be 90° and the face twist angle is 60°). For a perfect trigonal prism Θ would be 1440° (face twist angles are 0°). Continuous Shape Measurement was performed using SHAPE 2.1.[36]

Table S4. Crystallographic data for **1**, **1·Et$_2$O**, and **2**.

	**1**	**1·Et$_2$O**	**2**
Identification code	ccapg177	lcapg92	ccapg13
Formula	$C_{105.6}H_{136.4}Cl_2S_2U$ [B]	$C_{80}H_{118}Cl_2O_2S_2U$	$C_{72}H_{130}B_8K_2S_2U_2$
Fw	1778.79	1484.79	1700.61
Temperature / K	100.00(10)	99.95(16)	100.2(4)
Crystal system	triclinic	triclinic	triclinic
Space group	P-1	P-1	P-1
a / Å	31.4577(4)	10.4746(3)	11.86511(17)
b / Å	34.7898(5)	14.1605(3)	12.36331(16)
c / Å	47.0942(7)	14.5776(3)	15.26257(18)
α / °	82.7500(10)	66.0087(19)	95.5455(11)
β / °	86.2050(10)	72.434(2)	111.1550(13)
γ / °	79.5760(10)	77.4993(19)	98.0713(11)
Volume / Å^3	50234.7(12)	1872.37(8)	2040.85(5)
Z	20	1	1
ρ_{calcg} / cm^3	1.176	1.317	1.384
μ / mm^{-1}	5.733	7.6	12.722
F(000)	18560	772	856
Crystal size / mm^3	0.143 × 0.086 × 0.069	0.126 × 0.084 × 0.022	0.123 × 0.101 × 0.07
Radiation	Cu Kα (λ = 1.54184)	Cu Kα (λ = 1.54184)	CuKα (λ = 1.54184)
2Θ range / °	4.642 to 80.26	6.872 to 153.176	6.29 to 140.124
Index ranges	-25 ≤ h ≤ 26, -29 ≤ k ≤ 28, -39 ≤ l ≤ 39	-12 ≤ h ≤ 12, -17 ≤ k ≤ 17, -18 ≤ l ≤ 18	-14 ≤ h ≤ 14, -15 ≤ k ≤ 11, -18 ≤ l ≤ 18
No. reflections	231987	44496	33231
Unique reflections	55707 [R_{int} = 0.1194, R_{sigma} = 0.1099]	7212 [R_{int} = 0.0622, R_{sigma} = 0.0395]	7735 [R_{int} = 0.0474, R_{sigma} = 0.0363]
Data / restraints / parameters	55707/22477/6941	7212/672/550	7735/292/452
GOOF on F^2	0.944	1.042	1.046
Final R indexes [I ≥ 2σ (I)] [A]	R_1 = 0.0622, wR_2 = 0.1559	R_1 = 0.0324, wR_2 = 0.0812	R_1 = 0.0323, wR_2 = 0.0801
Final R indexes [all data]	R_1 = 0.1129, wR_2 = 0.1806	R_1 = 0.0325, wR_2 = 0.0815	R_1 = 0.0334, wR_2 = 0.0809
Largest diff. (peak / hole) / e Å^{-3}	0.92/-0.39	1.27/-2.13	2.68/-1.41

[A] $R = \sum||F_O| - |F_C||/\sum|F_O|$; $R_W = [\sum w(F_O^2 - F_C^2)^2/\sum w(F_O^2)^2]^{0.5}$; $S = [\sum w(F_O^2 - F_C^2)^2/(\text{no. data} - \text{no. params})]^{0.5}$ for all data.
[B] Note that the non-integer formula is due to the presence of 35 toluene molecules and 10 complex molecules per asymmetric unit.

Table S5. Crystallographic data for **3**, **4a**, and **4b**.

	**3**	**4a**	**4b**
Identification code	ccapg17	ccapg180	ccapg189
Formula	$C_{43}H_{68}B_3SU$	$C_{36}H_{57}B_2SU$	$C_{77}H_{124}B_4S_2U_2$
Fw	887.49	781.52	1633.17
Temperature / K	100.15	100.00(10)	100.00(10)
Crystal system	triclinic	monoclinic	monoclinic
Space group	P-1	$P2_1/c$	$P2_1/n$
a / Å	9.50438(9)	22.5670(11)	12.3142(2)
b / Å	9.77146(10)	10.0332(8)	22.2378(3)
c / Å	26.26948(19)	16.3355(9)	14.0552(2)
α / °	79.7889(7)	90	90
β / °	82.0605(7)	93.525(6)	95.8100(10)
γ / °	63.0408(10)	90	90
Volume / $Å^3$	2135.48(4)	3691.7(4)	3829.11(10)
Z	2	4	2
ρ_{calcg} / cm^3	1.38	1.406	1.416
μ / mm^{-1}	11.333	13.038	12.593
F(000)	898	1564	1644
Crystal size / mm^3	0.039 × 0.028 × 0.022	0.191 × 0.129 × 0.081	0.095 × 0.053 × 0.042
Radiation	CuKα (λ = 1.54184)	Cu Kα (λ = 1.54184)	CuKα (λ = 1.54184)
2Θ range / °	3.424 to 140.114	7.85 to 96.854	7.468 to 140.142
Index ranges	-11 ≤ h ≤ 11, -11 ≤ k ≤ 11, -32 ≤ l ≤ 31	-21 ≤ h ≤ 21, -8 ≤ k ≤ 4, -15 ≤ l ≤ 15	-14 ≤ h ≤ 15, -27 ≤ k ≤ 27, -17 ≤ l ≤ 13
No. reflections	32362	8530	35572
Unique reflections	8064 [R_{int} = 0.0458, R_{sigma} = 0.0362]	3187 [R_{int} = 0.0576, R_{sigma} = 0.0690]	7255 [R_{int} = 0.0446, R_{sigma} = 0.0348]
Data / restraints / parameters	8064/797/599	3187/1116/498	7255/1120/599
GOOF on F^2	1.078	1.079	1.045
Final R indexes [I ≥ 2σ (I)] [A]	R_1 = 0.0273, wR_2 = 0.0641	R_1 = 0.0609, wR_2 = 0.1513	R_1 = 0.0325, wR_2 = 0.0846
Final R indexes [all data]	R_1 = 0.0297, wR_2 = 0.0648	R_1 = 0.0797, wR_2 = 0.1610	R_1 = 0.0399, wR_2 = 0.0884
Largest diff. (peak / hole) / e $Å^{-3}$	2.26/-1.96	1.73/-1.24	1.24/-0.48

[A] $R = \sum||F_O| - |F_C||/\sum|F_O|$; $R_W = [\sum w(F_O^2 - F_C^2)^2/\sum w(F_O^2)^2]^{0.5}$; $S = [\sum w(F_O^2 - F_C^2)^2/(\text{no. data} - \text{no. params})]^{0.5}$ for all data.

Table S6. Crystallographic data for **5**, **6**, and $[U^{IV}(BH_4)_2\{\mu\text{-}B_2H_6\}]_n$.

	**5**	**6**	$[U^{IV}(BH_4)_2\{\mu\text{-}B_2H_6\}]_n$
Identification code	ccapg14	ccapg10	ccapg10
Formula	$C_{72}H_{102}BS_2U$	$B_4H_{14}U$	$B_4H_{14}U$
Fw	1280.49	295.38	295.38
Temperature / K	99.97(16)	99.97(14)	99.97(14)
Crystal system	triclinic	monoclinic	monoclinic
Space group	P-1	$P2_1/n$	$P2_1/n$
a / Å	13.3689(2)	7.6635(2)	7.6635(2)
b / Å	14.1556(2)	11.8764(4)	11.8764(4)
c / Å	18.4818(3)	8.0272(3)	8.0272(3)
α / °	102.9475(13)	90	90
β / °	94.0338(12)	94.972(3)	94.972(3)
γ / °	99.5134(13)	90	90
Volume / $Å^3$	3340.74(9)	727.84(4)	727.84(4)
Z	2	4	4
ρ_{calcg} / cm^3	1.273	2.696	2.696
μ / mm^{-1}	7.694	61.648	61.648
F(000)	1326	504	504
Crystal size / mm^3	0.086 × 0.079 × 0.045	0.051 × 0.04 × 0.017	0.051 × 0.04 × 0.017
Radiation	CuKα (λ = 1.54184)	CuKα (λ = 1.54184)	CuKα (λ = 1.54184)
2Θ range / °	4.938 to 140.148	13.348 to 159.792	13.348 to 159.792
Index ranges	-16 ≤ h ≤ 16, -17 ≤ k ≤ 16, -21 ≤ l ≤ 22	-9 ≤ h ≤ 9, -15 ≤ k ≤ 13, -9 ≤ l ≤ 10	-9 ≤ h ≤ 9, -15 ≤ k ≤ 13, -9 ≤ l ≤ 10
No. reflections	59748	5381	5381
Unique reflections	12681 [R_{int} = 0.0437, R_{sigma} = 0.0350]	1555 [R_{int} = 0.0499, R_{sigma} = 0.0464]	1555 [R_{int} = 0.0499, R_{sigma} = 0.0464]
Data / restraints / parameters	12681/2293/864	1555/95/89	1555/95/89
GOOF on F^2	1.059	1.173	1.173
Final R indexes [I ≥ 2σ (I)] [A]	R_1 = 0.0278, wR_2 = 0.0693	R_1 = 0.0392, wR_2 = 0.1133	R_1 = 0.0392, wR_2 = 0.1133
Final R indexes [all data]	R_1 = 0.0303, wR_2 = 0.0702	R_1 = 0.0429, wR_2 = 0.1161	R_1 = 0.0429, wR_2 = 0.1161
Largest diff. (peak / hole) / e $Å^{-3}$	3.00/-2.15	1.72/-2.56	1.72/-2.56

[A] $R = \sum||F_O| - |F_C||/\sum|F_O|$; $R_W = [\sum w(F_O^2 - F_C^2)^2/\sum w(F_O^2)^2]^{0.5}$; $S = [\sum w(F_O^2 - F_C^2)^2/(\text{no. data} - \text{no. params})]^{0.5}$ for all data.

Table S7. Crystallographic data for $(SAr^{iPr6})_2$.§[37]

	$(SAr^{iPr6})_2$
Identification code	Icapg69
Formula	$C_{72}H_{98}S_2{\cdot}OC_4H_8$
Fw	1101.74
Temperature / K	100.0(2)
Crystal system	triclinic
Space group	P-1
a / Å	12.6813(5)
b / Å	13.6812(5)
c / Å	20.3253(6)
α / °	100.502(3)
β / °	92.140(3)
γ / °	96.201(3)
Volume / $Å^3$	3441.1(2)
Z	2
ρ_{calcg} / cm^3	1.063
μ / mm^{-1}	0.997
F(000)	1208
Crystal size / mm^3	0.149 × 0.117 × 0.082
Radiation	Cu Kα (λ = 1.54184)
2Θ range / °	4.43 to 140.14
Index ranges	-15 ≤ h ≤ 15, -16 ≤ k ≤ 16, -24 ≤ l ≤ 24
No. reflections	54304
Unique reflections	13039 [R_{int} = 0.0677, R_{sigma} = 0.0652]
Data / restraints / parameters	13039/216/789
GOOF on F^2	1.067
Final R indexes [I ≥ 2σ (I)] [A]	R_1 = 0.0506, wR_2 = 0.1350
Final R indexes [all data]	R_1 = 0.0566, wR_2 = 0.1395
Largest diff. (peak / hole) / e $Å^{-3}$	0.66/-0.40

[A] $R = \sum||F_O| - |F_C||/\sum|F_O|$; $R_W = [\sum w(F_O^2 - F_C^2)^2/\sum w(F_O^2)^2]^{0.5}$; $S = [\sum w(F_O^2 - F_C^2)^2/(\text{no. data} - \text{no. params})]^{0.5}$ for all data.

§ This compound is structurally similar to the Se and Te analogues previously reported by Poleschner and co-workers.[37]

Powder X-ray diffraction (PXRD) studies

Due to ambiguities in the elemental analysis data for complexes **3** and **5**, powder X-ray diffraction (PXRD) was used to determine their bulk purity.

Microcrystalline samples of **3** and **5** were flame sealed inside 0.5 mm outer diameter borosilicate capillaries (Wilmad) and data were collected using a Rigaku FR-X DW diffractometer, equipped with an FR-X high-intensity rotating anode Cu Kα radiation source (λ = 1.54184 Å) and VariMAXTM microfocus optics, using an AFC-11 4-circle κ goniometer, a HyPix-6000HE hybrid pixel array detector operating in shutterless mode and an Oxford Cryosystems Cryostream 800 nitrogen flow gas system at room temperature. Data were collected between 3–50 °θ, for **5** and 3-22° for **3**, with a detector distance of 150 mm and a beam divergence of 0.5 mRad.[38] X-ray data were collected using CrysAlisPro software.[23]

The instrument was calibrated using the collected data, with the instrument model refined using diffraction peak positions measured at multiple detector angles. X-ray data were reduced and integrated using CrysAlisPro software.[23] Peak hunting and unit cell indexing was performed using TOPAS software.[39] Le Bail profile analysis was performed using JANA2020 software.[40]

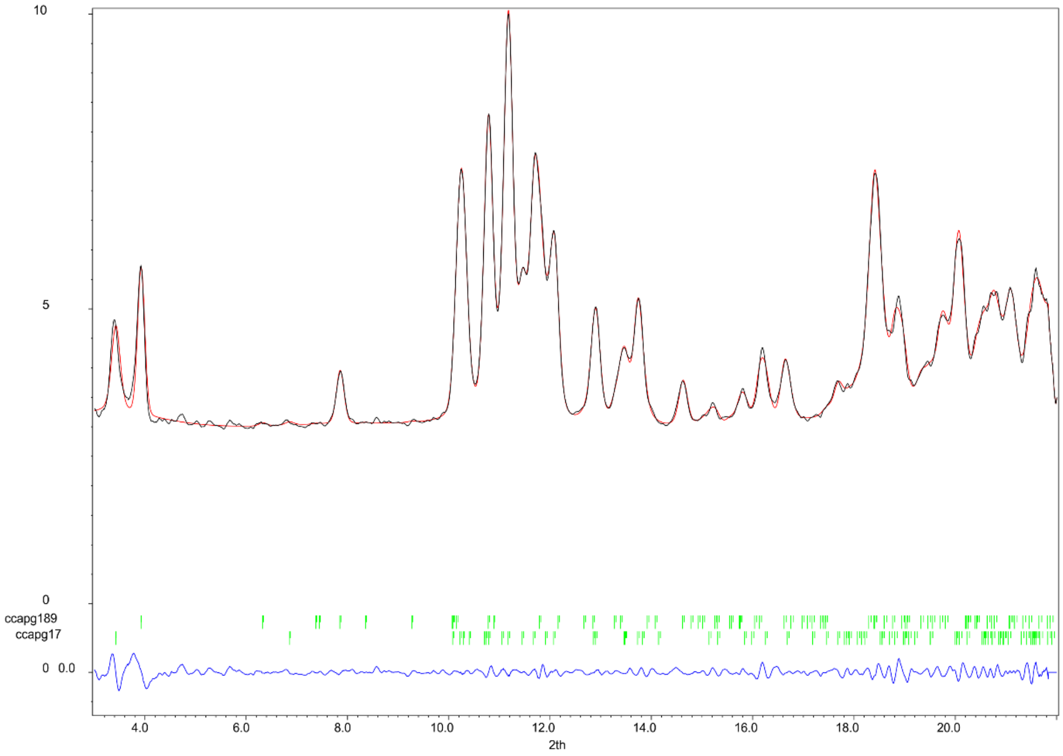

Figure S8. Full XRD pattern for lcapg105, PXRD from a sample of **3** (black = observed data, red = calculated profile, green = reflection positions, blue = obs-cal difference).

Comparison between the PXRD pattern determined from a batch of **3** (lcapg105, **Figure S8** and also **Figure S9** black line) and the pattern calculated from the SC-XRD structure of **3** (**Figure S9** blue line), revealed peaks present in the PXRD pattern which are not accounted for. Therefore, the data was fit to two unit cells, labelled lcapg105a and lcapg105b in **Table S8**.

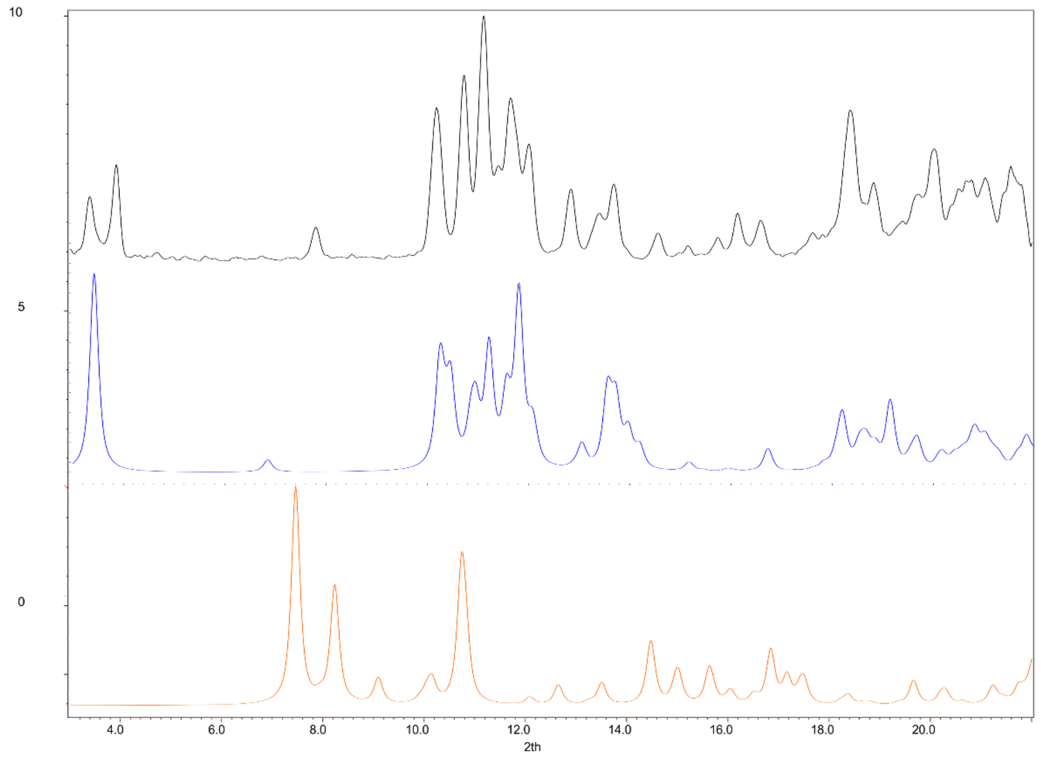

Figure S9. Comparison of observed PXRD pattern (lcapg105, black line) and calculated PXRD pattern from single crystal data for **3** (SC-XRD of **3**, blue line), and also of complex **4b** (SC-XRD of **4b**, red line). Black = observed data, blue = calculated data for **3**, red = calculated data for **4b**, FWHM 0.2).[41]

Table S8. Unit cell comparison between PXRD and SC-XRD data for **3**.

**Name**	PXRD for **3**<br>Cell lcapg105a	SC-XRD for **3**<br>ccapg17	PXRD for **3**<br>Cell lcapg105b	SC-XRD for **4a**<br>ccapg189
a / Å	9.5862(4)	9.50438(9)	12.0286(12)	12.3142(2)
b / Å	9.9553(6)	9.77146(10)	22.5099(12)	22.2378(3)
c / Å	26.2539(12)	26.26948(19)	14.0238(16)	14.0552(2)
α / °	79.865(5)	79.7889(7)	90	90
β / °	81.958(3)	82.0605(7)	95.259(10)	95.8100(10)
γ / °	62.685(4)	63.0408(10)	90	90
Volume / Å^3	2186.4(2)	2135.48(4)	3781.1(6)	3829.11(10)
Temperature / K	295	100	295	100

Cell lapg105a fits with good agreement to the cell for ccapg17 (*i.e.* the SC-XRD structure of **3** which contains a disordered lattice toluene molecule). However, the unit cell is triclinic, so it is easy to fit

to a profile pattern as there are no constraints. The observed and calculated patterns are in reasonable agreement, although there are some additional peaks present.

Due to the presence of minor additional ^{11}B resonances in the ^{11}B NMR spectrum of **3** which could be due to a dimeric species possibly analogous to **4b** (*i.e.* [{U^{III}(H_3B·SAr^{iPr6}-κS,H,H)(BH_4)}$_2$(μ-B_2H_6)] – though such a species was not identified by SC-XRD), and due to the superficial similarities in the cell of lcapg105b to that of ccapg189 (*i.e.* the SC-XRD structure of **4b**), we explored this second unit cell further.

The cell for lcapg105b was initially profile fit using the P1 spacegroup, though we note ccapg189 (**4b**) is monoclinic. The lcapg105b cell was then refined with monoclinic constraints. The major reflections at low angle should be systematically absent if the spacegroup was P21/n. While there is a possibility that lcapg105b is an analogue of **4b** with an S-bound BH_3, unit, it is also possible that it is something completely different and that the peak positions in lcapg105b matching the cell of **4b** is entirely coincidental. Cell lacapg105b, collected at room temperature, is smaller in volume than that of **4b**, collected at 100 K, which could indicate that it is not correct as the cell volume at room temperature should be larger – though we cannot speculate on what the unit cell parameters of putative [{U^{III}(H_3B·SAr^{iPr6}-κS,H,H)(BH_4)}$_2$(μ-B_2H_6)] would be, and have no unambiguous data to suggest such a species is present. In conclusion, while the unit cell for lcapg105a is a good fit for that of complex **3**, the total PXRD pattern is a relatively poor match for ccapg17 alone, and contains (at least) a second component that does not match any of the other complexes herein. We cannot discount the possibility that the additional crystalline phase is simply a polymorph of **3** which does not contain the disordered lattice toluene molecule.

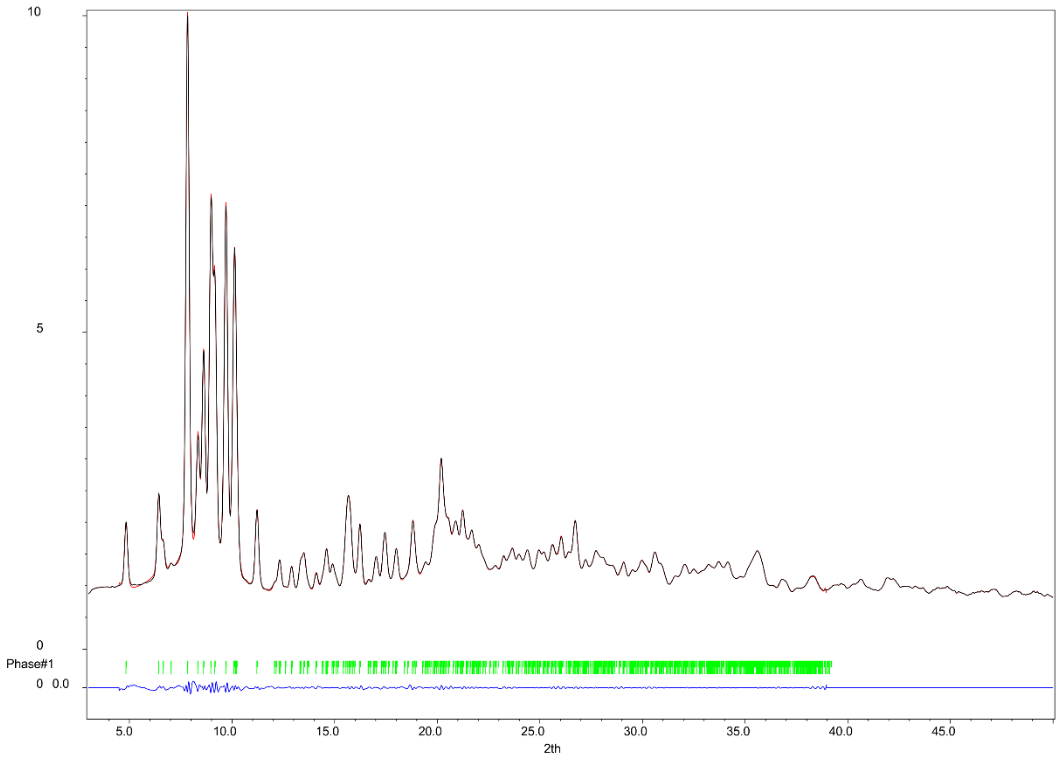

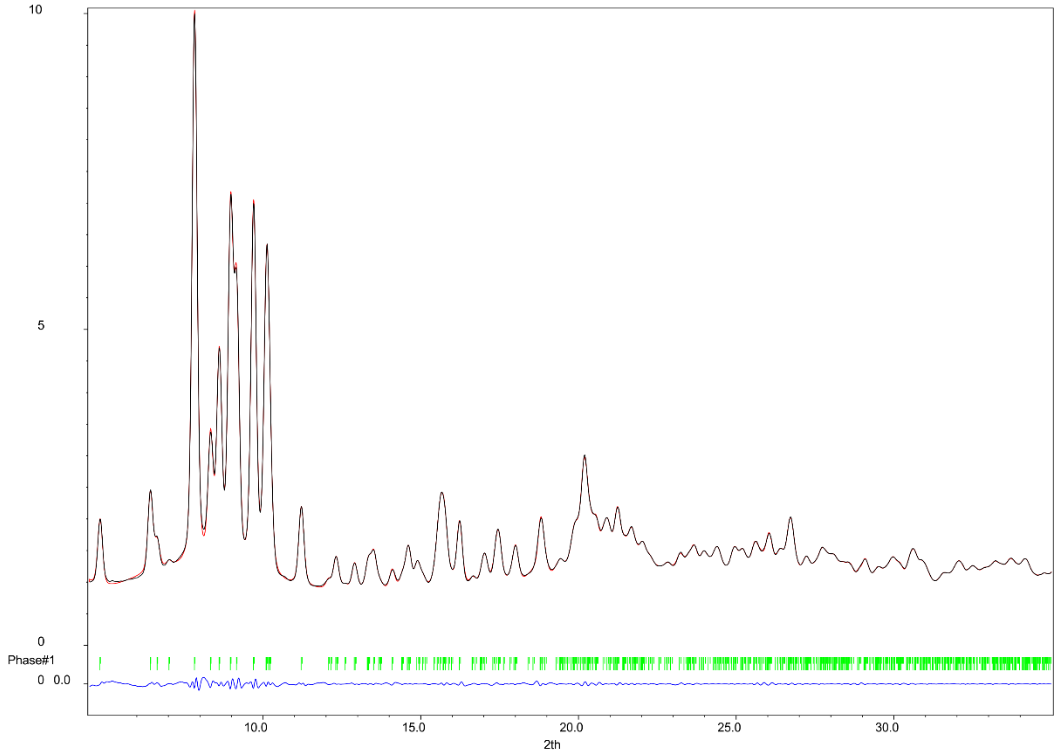

Figure S10. (Top) Full XRD pattern for Icapg103, PXRD from a sample of **5**; (Bottom) Selected range for Le Bail profile fitting (black = observed data, red = calculated profile, green = reflection positions, blue = obs-cal difference).

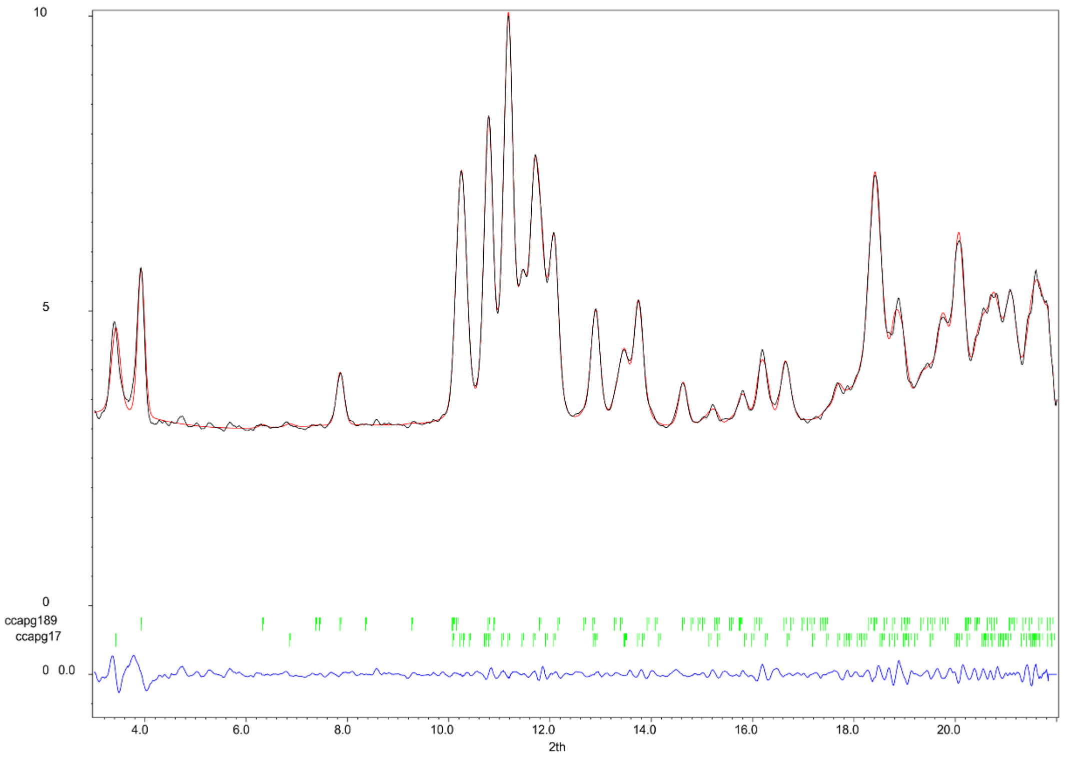

Figure S11. Comparison of observed PXRD pattern (lcapg103) and calculated PXRD pattern from single crystal data for ccapg14 (SC-XRD of **5**; black = observed data, blue = calculated data, FWHM 0.2).[41]

Table S9. Unit cell comparison between PXRD and SC-XRD data for **5**.

**Name**	PXRD for **5** lcapg103	SC-XRD for **5** ccapg14
a / Å	13.5185(3)	13.3689(2)
b / Å	14.3554(3)	14.1556(2)
c / Å	18.8108(4)	18.4818(3)
α / °	103.6016(12)	102.9475(13)
β / °	93.1519(10)	94.0338(12)
γ / °	99.8079(14)	99.5134(13)
Volume / Å^3	3478.98(12)	3340.74(9)
Temperature / K	295	100

S3. Molecular Structures

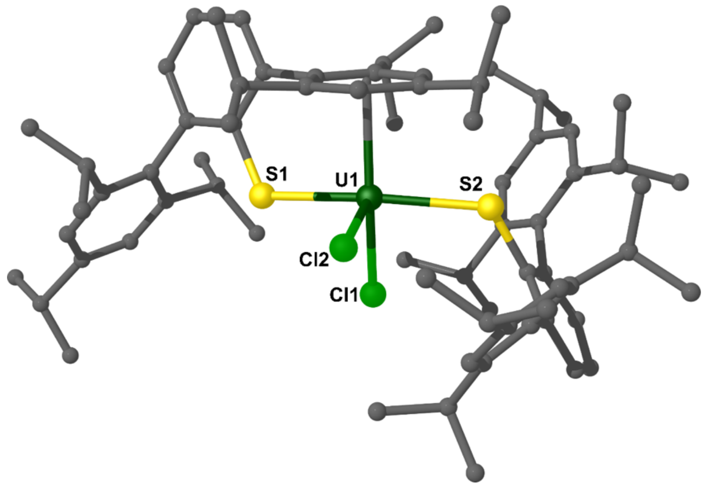

Figure S12. Molecular structure of **1**. Due to diffraction observed only to 1.20 Å, the molecular structure of **1** is represented by the ball-and-stick model. H-atoms removed for clarity.

When inspecting the U–S bond lengths in **1**, it is apparent that there are two distinct series due to the two distinct ligand binding modes. One of the ligand is only bound through the S-atom (U–S range 2.647(5)–2.670(5) Å); the second ligand features an additional U···η^6-Tripp interaction which results in a significant lengthening of the U–S distances (range 2.682(4)–2.702(5) Å).

Table S10. Selected mean metrical parameters for complex **1**.

U centre	U–S (Å)	U–Cl (Å)	S–U–S (°)	Cl–U–Cl (°)	U···η^6-Tripp$_{cent}$ (Å)
**U1A**	2.652(4), 2.686(5)	2.536(5), 2.541(4)	155.53(16)	96.54(16)	2.591(5)
**U1B**	2.650(5), 2.687(5)	2.531(6), 2.535(5)	155.12(19)	95.35(18)	2.589(5)
**U1C**	2.658(5), 2.696(5)	2.534(5), 2.544(4)	153.28(17)	95.82(15)	2.588(5)
**U1D**	2.647(5), 2.701(4)	2.539(5), 2.546(4)	155.52(17)	96.02(16)	2.594(5)
**U1E**	2.670(5), 2.690(5)	2.537(5), 2.539(4)	154.03(18)	95.03(16)	2.591(5)
**U1F**	2.660(5), 2.687(5)	2.524(5), 2.533(4)	153.90(17)	95.25(16)	2.594(5)
**U1G**	2.657(4), 2.691(5)	2.537(4), 2.540(5)	154.05(17)	95.48(17)	2.589(5)
**U1H**	2.651(4), 2.694(5)	2.535(5), 2.542(4)	154.63(17)	95.11(16)	2.593(5)
**U1I**	2.668(5), 2.702(5)	2.543(5), 2.544(4)	154.38(18)	95.86(16)	2.591(6)
**U1J**	2.663(4), 2.682(4)	2.531(5), 2.540(4)	154.35(17)	95.60(17)	2.589(6)
**Total range**	U–S1: 2.682(4)–2.702(5) U–S2: 2.647(5)–2.670(5)	2.524(5)–2.546(4)	153.28(17)–155.53(16)	95.03(16)–96.54(16)	2.588(5)–2.594(5)

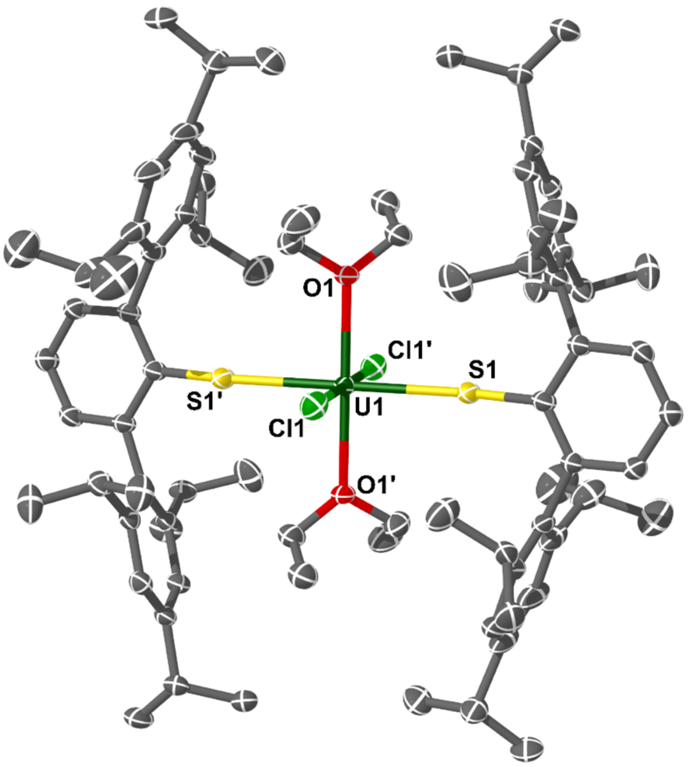

Figure S13. Molecular structure of complex **1·Et₂O**. Ellipsoids set at 50% probability and H-atoms removed for clarity (operations: 1–X, 1–Y, 1–Z). Selected bond lengths and angles: U1–S1 = 2.6526(9) Å, U1–Cl1 = 2.5699(10) Å, U1–O1 = 2.368(2) Å, U1–S1–C_{ipso} = 158.71(14)°.

Table S11. Continuous Shape parameters for complex **1·Et_2O**.[36]

Structure	Octahedral	Trigonal prismatic
**1·Et_2O**	0.306	16.315

Table S12. Octahedral distortion parameters for complex **1·Et_2O**.[42]

Structure	ζ	Δ	Σ	Θ
**1·Et_2O**	0.647771	0.002226	22.681645	77.942807

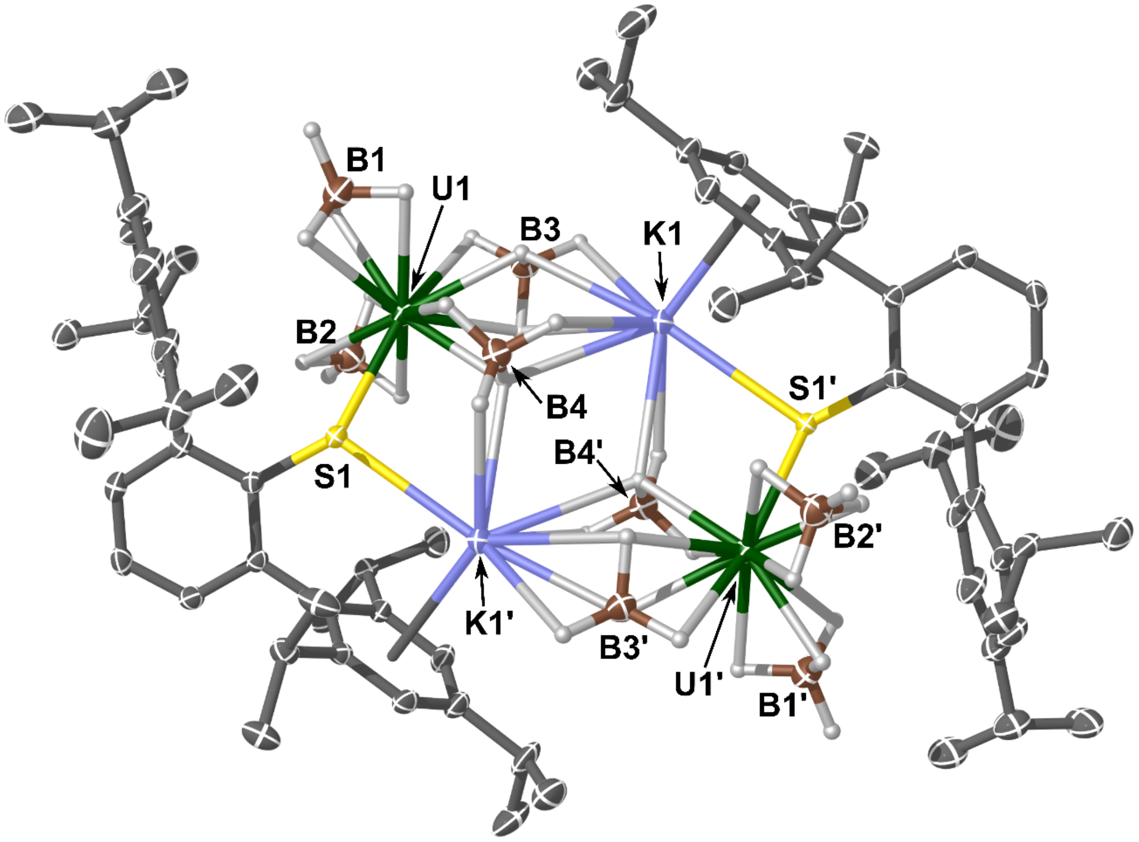

Figure S14. Molecular structure of complex **2**. Ellipsoids set at 50% probability and non-BH_4 H-atoms removed for clarity (operations: 1–X, 1–Y, 1–Z). Selected bond lengths and angles: U1–S1 = 2.6948(10) Å, K1–S1 = 3.1109(13) Å, U1···B1 = 2.489(6) Å, U1···B2 = 2.508(7) Å, U1···B3 = 2.557(6) Å, U1···B4 = 2.865(7) Å, K1···$Tripp_{centroid}$ = 2.881(2) Å, U1–S1–K1 = 101.94(3)°, U1–S1–C_{ipso} = 124.88(14)°.

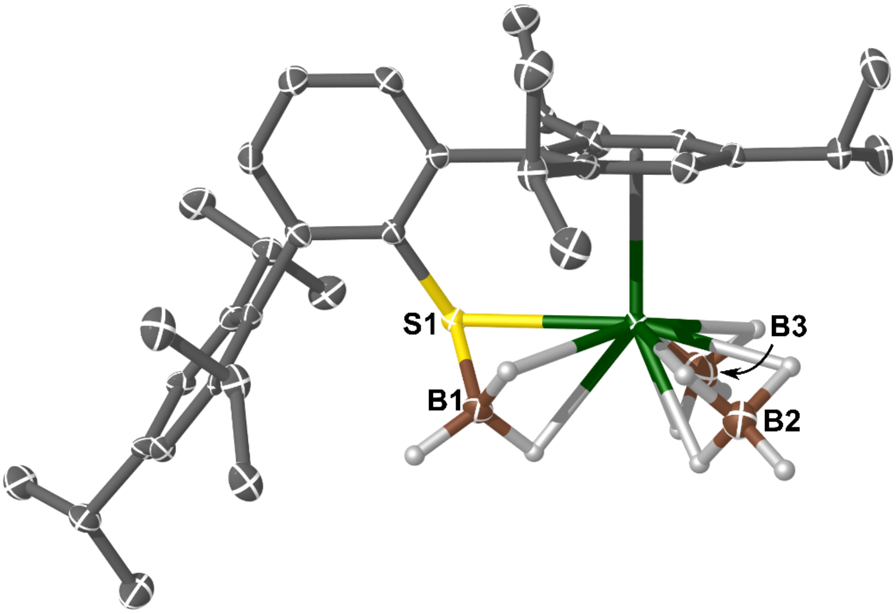

Figure S15. Molecular structure of complex **3**. Ellipsoids set at 50% probability. Non-BH H-atoms and lattice toluene removed for clarity (operations: X, Y, Z). Selected bond lengths and angles: U1–S1 = 2.8824(9) Å, S1–B1 = 1.939(5) Å, U1···B2 = 2.603(7) Å, U1···B3 = 2.584(6) Å, U1···Tripp$_{centroid}$ = 2.5379(15) Å, U1–S1–B1 = 64.55(14)°, U1–S1–C$_{ipso}$ = 109.54(12)°.

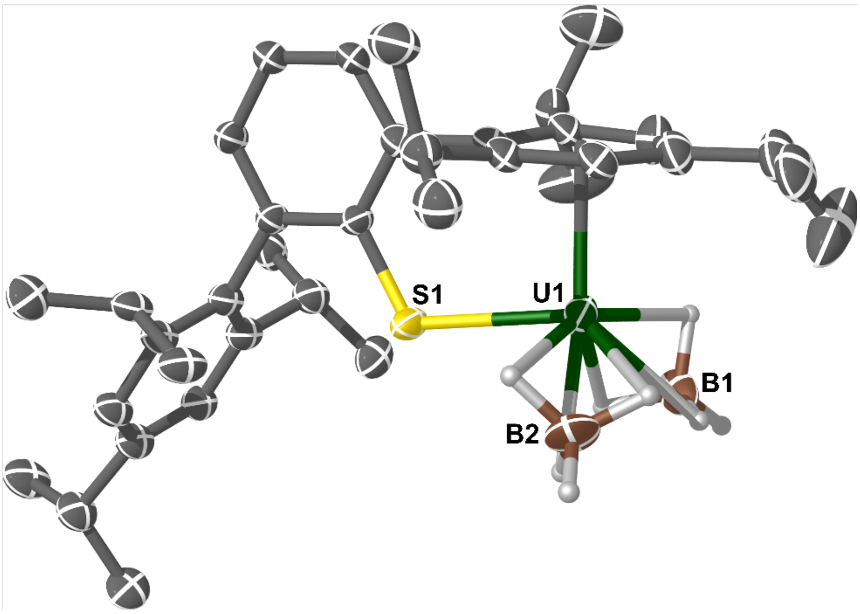

Figure S16. Molecular structure of complex **4a**. Ellipsoids set at 30% probability and non-BH_4 H-atoms removed for clarity (operations: X, Y, Z). Selected bond lengths and angles: U1–S1 = 2.687(4) Å, U1···B1 = 2.56(2) Å, U1···B2 = 2.56(3) Å, U1···Tripp$_{centroid}$ = 2.482(7) Å, U1–S1–C$_{ipso}$ = 113.6(5)°.

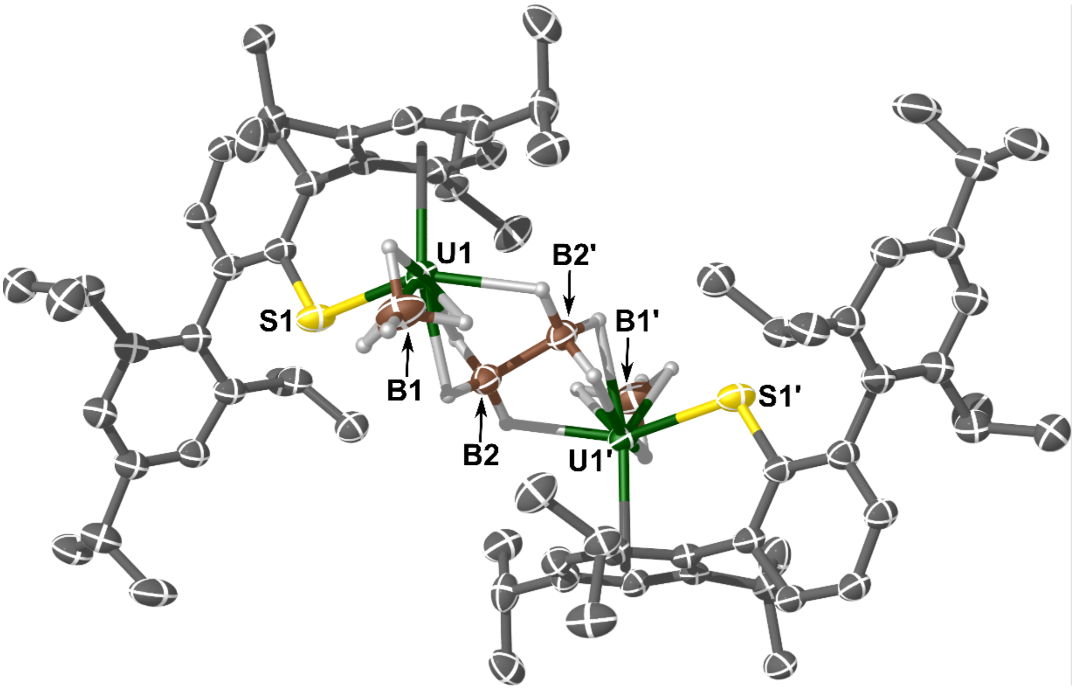

Figure S17. Molecular structure of complex **4b**. Ellipsoids set at 30% probability. Non-BH_4 H-atoms and lattice pentane removed for clarity (1–X, 1–Y, 1–Z). Selected bond lengths and angles: U1–S1 = 2.721(3) Å, U1···B1 = 2.556(8) Å, U1···B2 = 2.609(6) Å, U1···B2′ = 2.892(6) Å, U1···$Tripp_{centroid}$ = 2.5168(19) Å, U1–S1–C_{ipso} = 111.7(4)°.

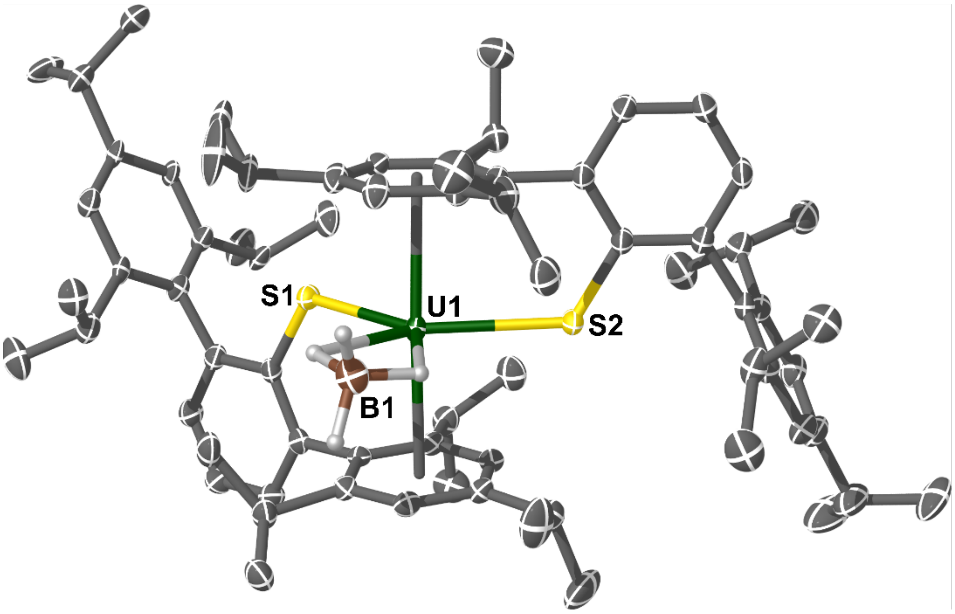

Figure S18. Molecular structure of complex **5**. Ellipsoids set at 50% probability and non-BH_4 H-atoms removed for clarity (operations: X, Y, Z). Selected bond lengths and angles: U1–S1 = 2.7888(8) Å, U1–S2 = 2.7969(7) Å, U1···B1 = 2.872(4) Å, U1···$Tripp_{centroid1}$ = 2.744(2) Å, U1···$Tripp_{centroid2}$ = 2.747(2) Å, U1···S_2B-plane = 0.004(2) Å, S1–U1–S2 = 128.23(2)°, U1–S1–C_{ipso} = 118.15(11)°, U1–S2–C_{ipso} = 118.53(11)°, $Tripp_{centroid1}$···U1···$Tripp_{centroid2}$ = 176.79(4)°.

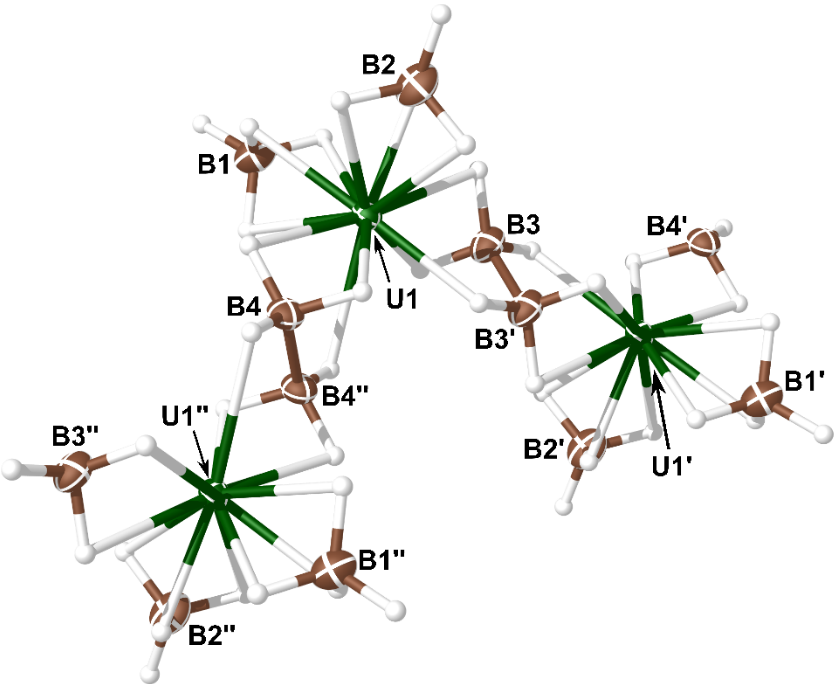

Figure S19. Molecular structure of $[U^{IV}(BH_4)_2\{\mu\text{-}B_2H_6\}]_n$. Ellipsoids set at 50% probability (operations: X, Y, Z; atom' 1–X, 1–Y, 1–Z; atom'' 1–X, 1–Y, 2–Z). Selected bond lengths: U1–B1 = 2.484(15) Å, U1–B2 = 2.497(15) Å, U1–B3 = 2.565(14) Å, U1–B4 = 2.570(15) Å.

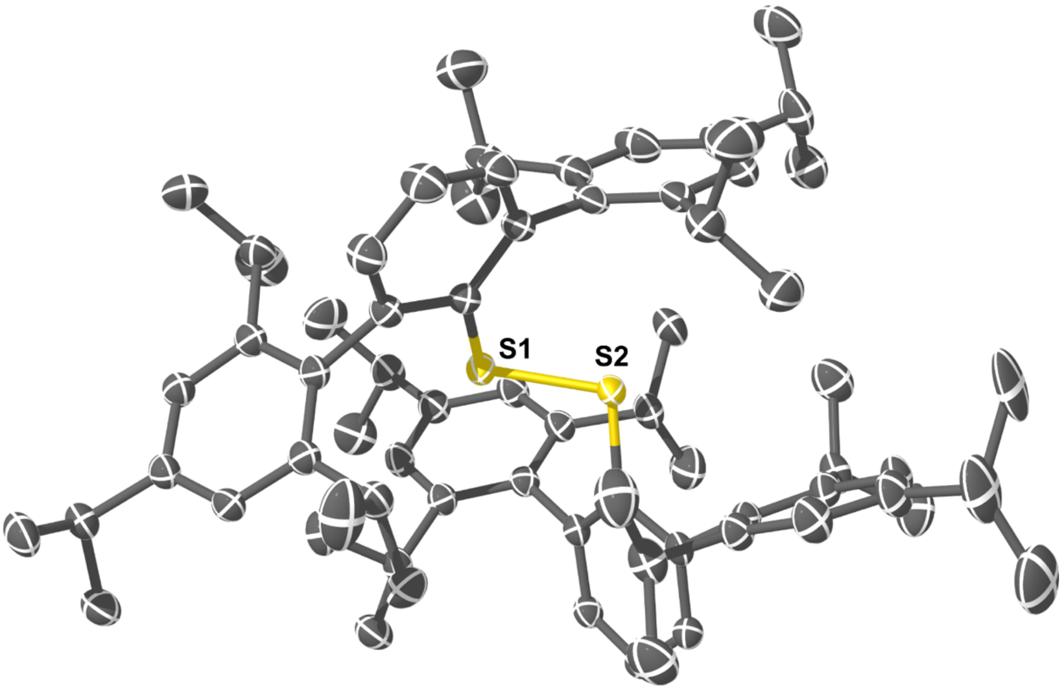

Figure S20. Molecular structure of $\{SAr^{iPr6}\}_2$. Ellipsoids set at 50% probability and H-atoms removed for clarity (operations: X, Y, Z). Selected bond lengths: S1–S2 = 2.0920(5) Å.

Table S13. Selected bond lengths (Å) and angles (°) for complexes **1**, **1·Et$_2$O**, **2**, **3**, **4a**, **4b**, and **5**.

(Å or °)	1	1·Et$_2$O	2	3	4a	4b	5
**U–S**	2.647(5)–2.702(5)	2.6526(9)	2.6948(10)	2.8824(9)	2.687(4)	2.721(3), 2.663(17)[B]	2.7888(8), 2.7969(7)
**U···B**	–	–	2.489(6) – 2.865(7)	2.584(6), 2.603(7)	2.56(2), 2.56(3)	2.556(8), 2.609(6)	2.872(4)
**U–TrippC$_6$**	N/D	–	–	2.883(4) – 2.920(4)	2.815(16) – 2.918(17)	2.867(5) – 2.899(5)	3.030(3) – 3.152(3)
**U···η^6-Tripp$_{cent}$**	2.588(5)–2.594	–	–	2.5379(15)	2.482(7)	2.5168(19)	2.744(2), 2.747(2)
**U–S–C$_{ipso}$**	[A]115.3(5), 123.3(6)	158.71(14)	124.88(14)	109.54(12)	113.6(5)	111.7(4), 114.0(11)[B]	118.53(11)

[A] Mean value of all ten independent molecules, and the ESD is given as the standard deviation of all the values. [B] Two disordered S-atoms are present which refined to a ratio of 0.82/0.18, the highest occupancy value is given first. N/D = Not determined.

Table S14. Uranium-arene bound contacts (Å) for complexes **3**, **4a**, **4b**, and **5**.

(Å)	3	4a	4b	5
**U1–C7,C43**	2.889(4)	2.844(16)	2.883(4)	3.075(3), 3.091(3)
**U1–C8,C44**	2.920(4)	2.918(17)	2.899(5)	3.132(3), 3.055(3)
**U1–C9,C45**	2.883(4)	2.84(2)	2.867(5)	3.103(3), 3.030(3)
**U1–C10,C46**	2.901(4)	2.835(19)	2.875(5)	–, 3.071(3)
**U1–C11,C47**	2.897(4)	2.815(16)	2.886(5)	3.048(3), 3.105(4)
**U1–C12,C48**	2.917(4)	2.851(17)	2.898(4)	3.056(3), 3.152(3)
**Mean**	2.901	2.851	2.885	3.083, 3.084
**Sandard deviation of the values**	*0.015*	*0.035*	*0.013*	*0.035*, *0.043*

Table S15. C–C bond lengths (Å) for U bound arene(s) in complexes **3**, **4a**, **4b**, and **5**

U bound arene C–C (Å)	3	4a	4b	5
**C7–C8, C43–C44**	1.419(5)	1.41(2)	1.438(6)	1.413(5), 1.411(5)
**C8–C9, C44–C45**	1.410(5)	1.40(2)	1.389(7)	1.408(5), 1.404(5)
**C9–C10, C45–C46**	1.396(6)	1.38(2)	1.397(7)	1.398(5), 1.392(5)
**C10–C11, C46–C47**	1.405(6)	1.41(2)	1.420(7)	1.405(5), 1.397(5)
**C11–C12, C47–C48**	1.405(5)	1.40(2)	1.394(7)	1.398(5), 1.401(5)
**C12–C7, C48–C43**	1.419(5)	1.39(2)	1.424(7)	1.416(4), 1.415(5)
**Mean**	1.409	1.398	1.410	1.406, 1.403
**Sandard deviation of the values**	*0.009*	*0.012*	*0.020*	*0.008, 0.009*

Table S16. C–C bond lengths (Å) for the non-bound arene(s) in complexes **3**, **4a**, **4b**, and **5**

Non-bound arene C–C (Å)	3	4a	4b	5
**C22–C23, C57–C58**	1.3900	1.3900	1.372(8)	1.3900, 1.403(5)
**C23–C24, C58–C59**	1.3900	1.3900	1.414(8)	1.3900, 1.393(5)
**C24–C25, C59–C60**	1.3900	1.3900	1.389(11)	1.3900, 1.385(6)
**C25–C26, C60–C61**	1.3900	1.3900	1.343(12)	1.3900, 1.391(6)
**C26–C27, C61–C62**	1.3900	1.3900	1.396(9)	1.3900, 1.399(5)
**C27–C22, C62–C57**	1.3900	1.3900	1.406(8)	1.3900, 1.408(5)
**Mean**	1.390	1.390	1.387	1.390, 1.397
**Sandard deviation of the values**	*0.000*	*0.000*	*0.026*	*0.000, 0.008*

S4. NMR Spectroscopy Plots

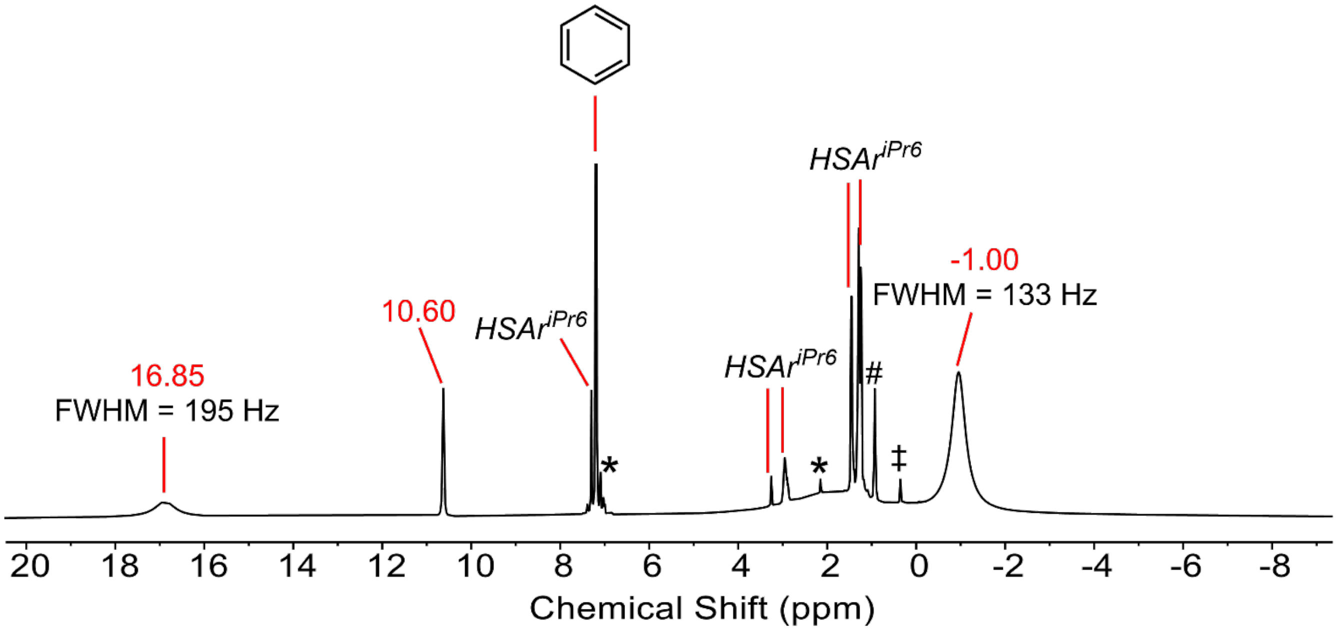

Figure S21. ^{1}H NMR spectrum of [$U^{IV}(SAr^{iPr6})_2(Cl)_2$] (**1**) in d_6-benzene between +20 and –10 ppm. *, #, and ‡ denote residual toluene, *n*-hexane, and silicone grease, respectively.

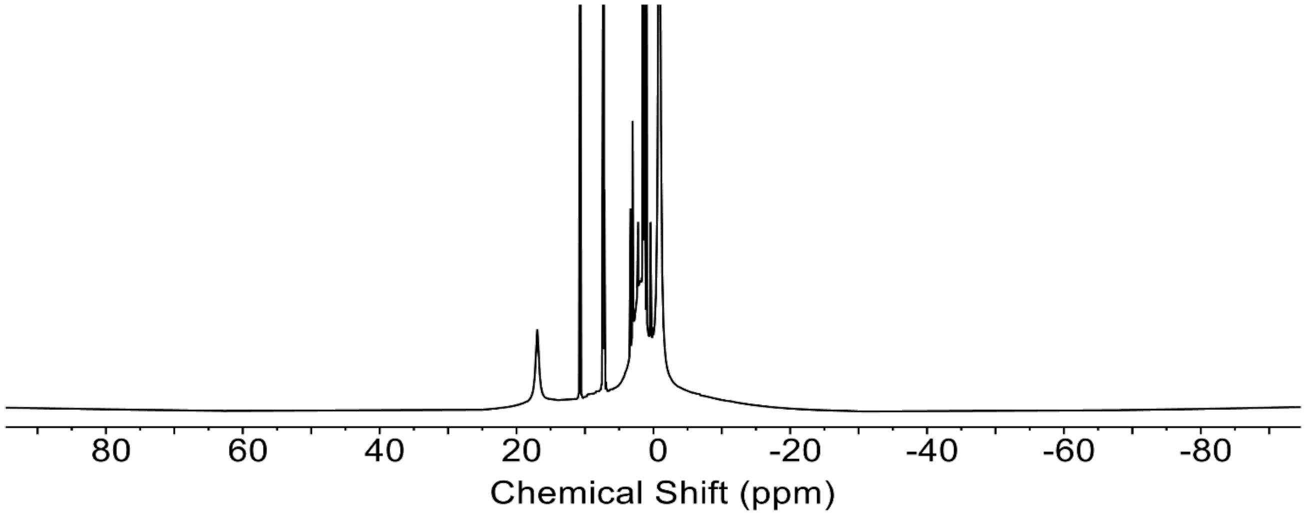

Figure S22. ^{1}H NMR spectrum of [$U^{IV}(SAr^{iPr6})_2(Cl)_2$] (**1**) in d_6-benzene showing the whole scan range (+100 to –100 ppm).

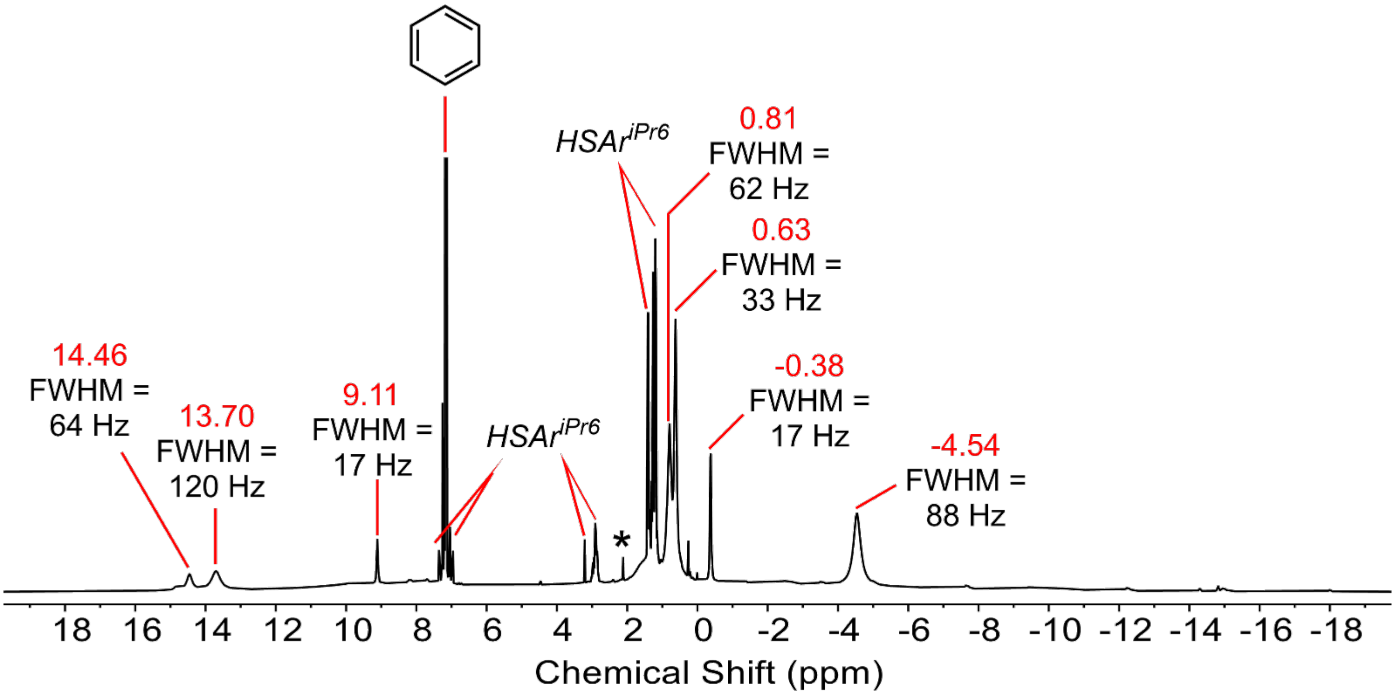

Figure S23. ^{1}H NMR spectrum of $[U^{IV}(\mu\text{-SAr}^{iPr6})(BH_4)_2(\mu\text{-}BH_4)(\mu^3\text{-}BH_4)K]_2$ (**2**) in d_6-benzene between +20 and –20 ppm. * denotes residual toluene.

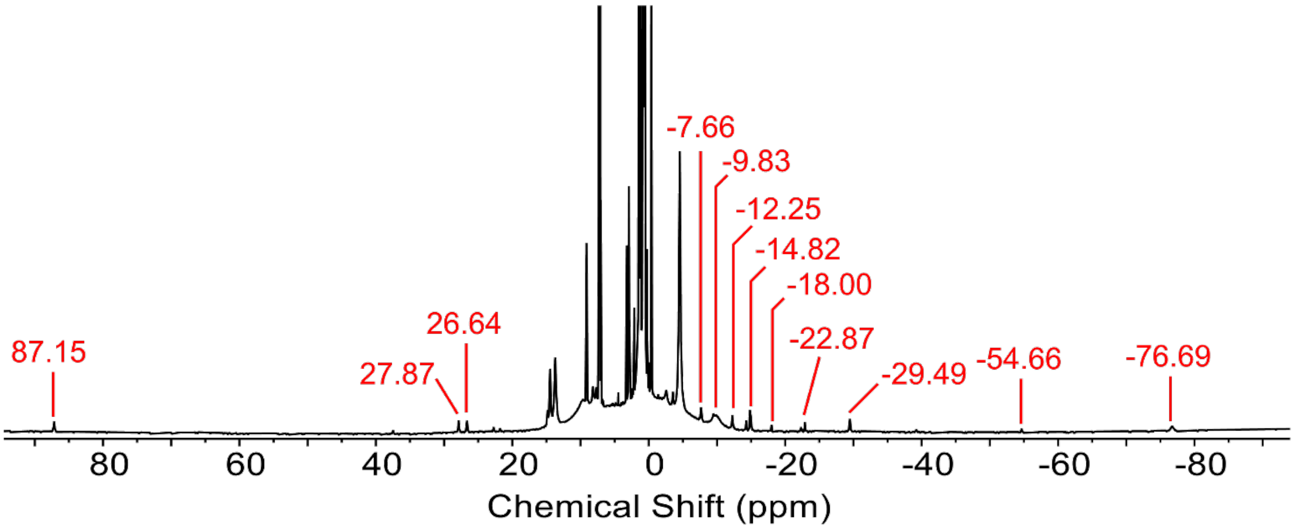

Figure S24. ^{1}H NMR spectrum of $[U^{IV}(\mu\text{-SAr}^{iPr6})(BH_4)_2(\mu\text{-}BH_4)(\mu^3\text{-}BH_4)K]_2$ (**2**) in d_6-benzene showing the whole scan range (+100 to –100 ppm).

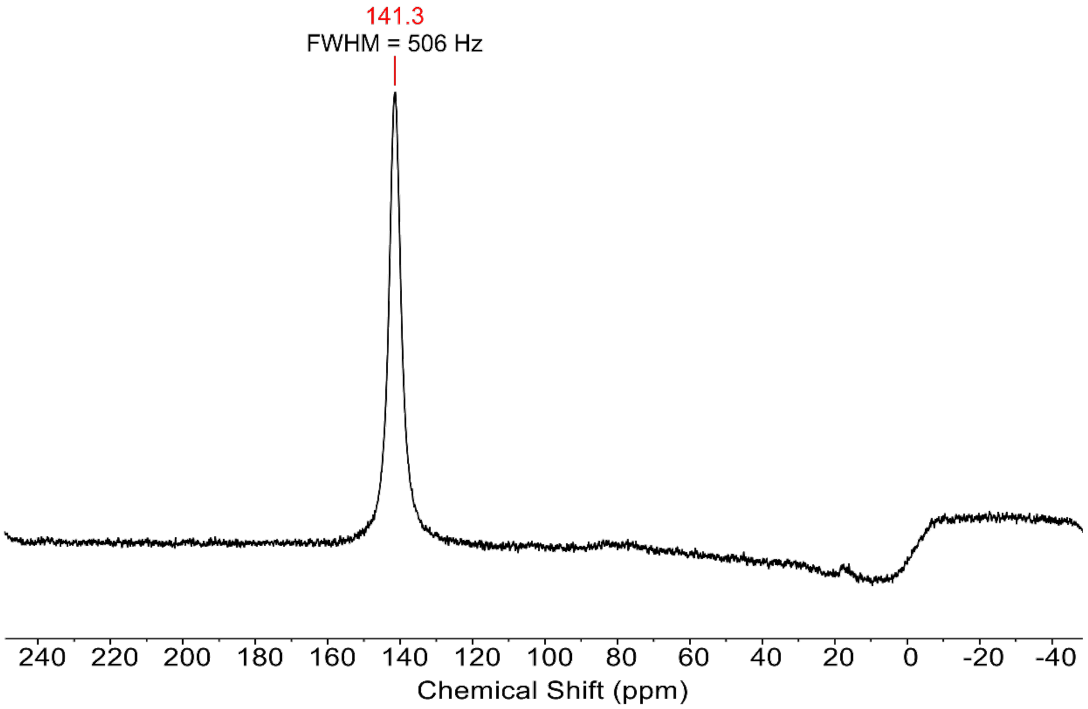

Figure S25. ^{11}B NMR spectrum of $[U^{IV}(\mu\text{-}SAr^{iPr6})(BH_4)_2(\mu\text{-}BH_4)(\mu^3\text{-}BH_4)K]_2$ (**2**) in d_6-benzene.

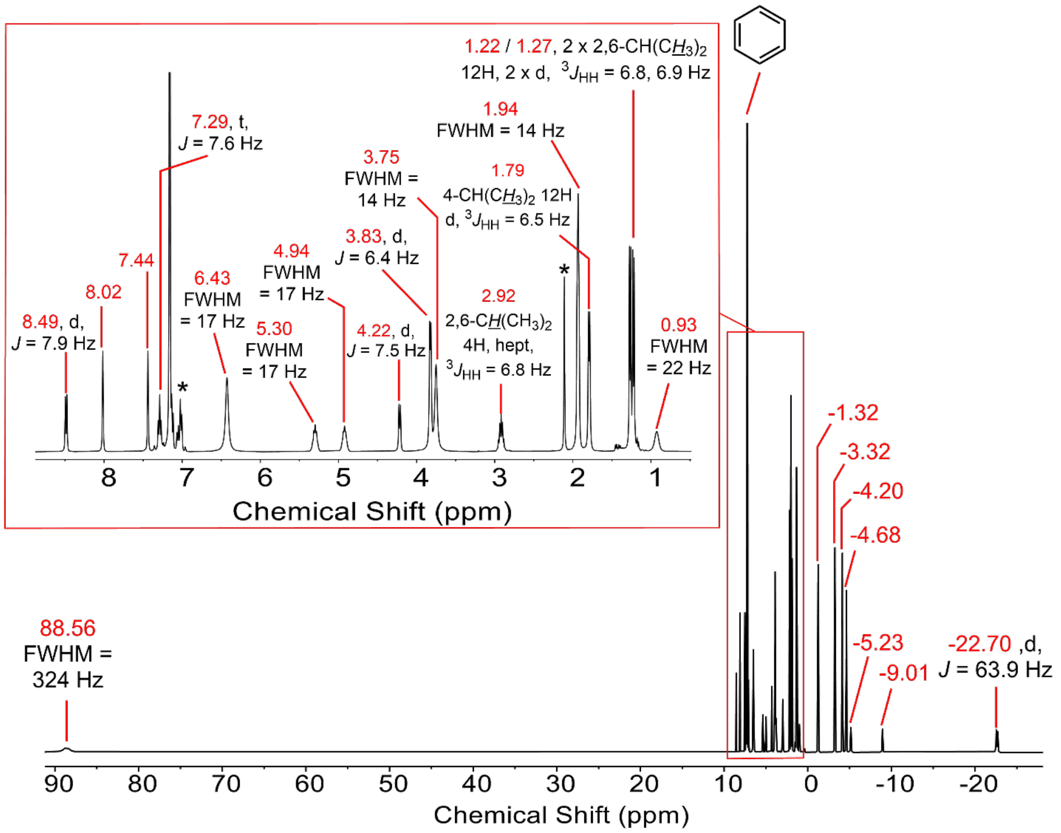

Figure S26. ^1H NMR spectrum of $[U^{III}(H_3B{\cdot}SAr^{iPr6}\text{-}\kappa S,H,H)(BH_4)_2]$ (**3**) in d_6-benzene between +90 and –30 ppm. * denotes residual toluene.

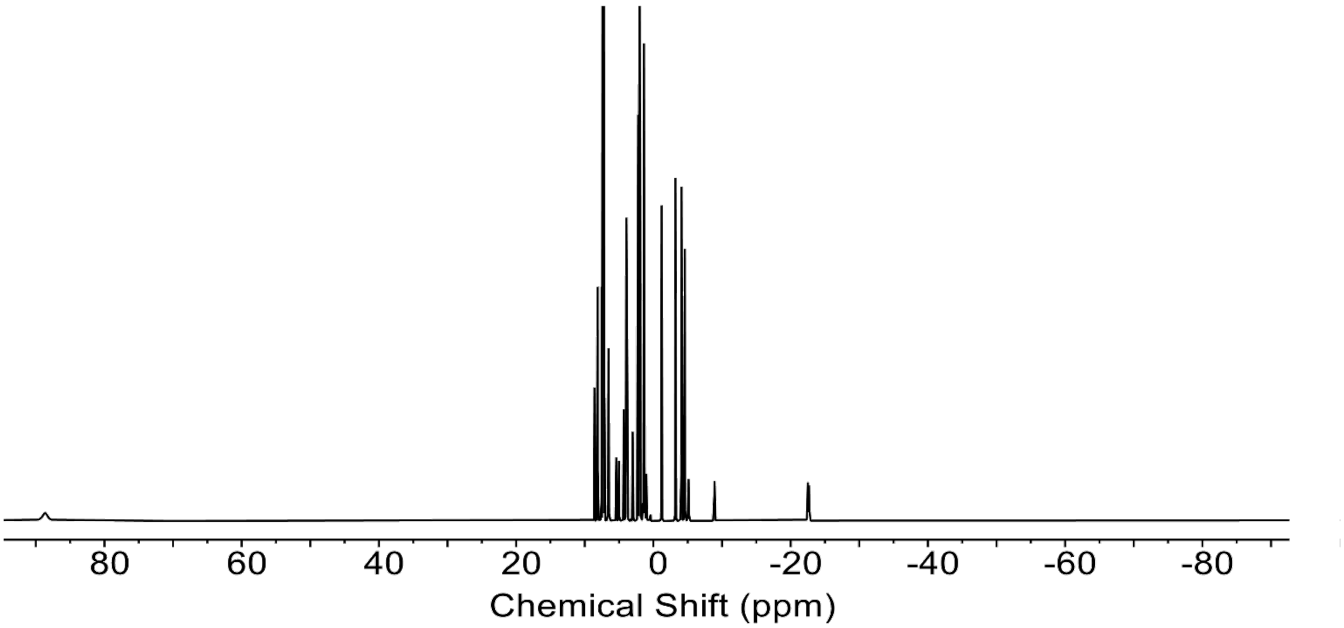

Figure S27. ^{1}H NMR spectrum of [U^{III}(H$_3$B·SAr^{iPr6}-κ*S*,*H*,*H*)(BH$_4$)$_2$] (**3**) in *d*$_6$-benzene showing the whole scan range (+100 to –100 ppm).

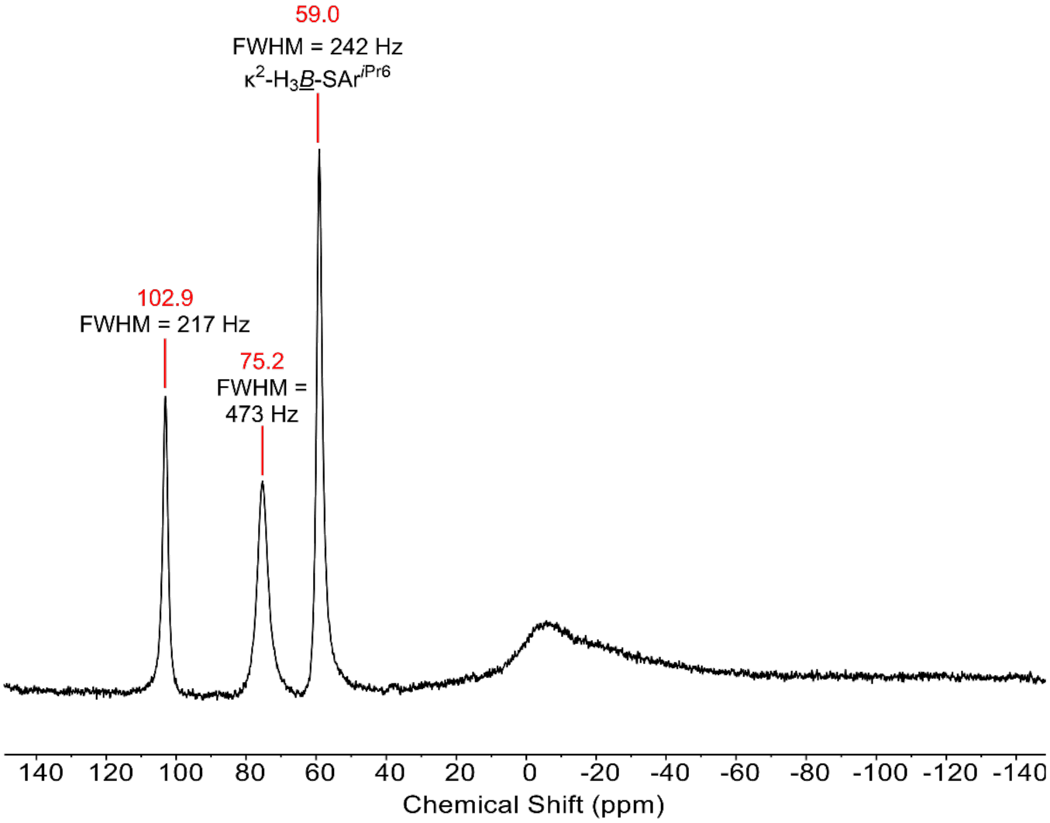

Figure S28. ^{11}B NMR spectrum of [U^{III}(H$_3$B·SAr^{iPr6}-κ*S*,*H*,*H*)(BH$_4$)$_2$] (**3**) in *d*$_6$-benzene. The feature between 0 and –40 ppm is due to the borosilicate NMR tube.

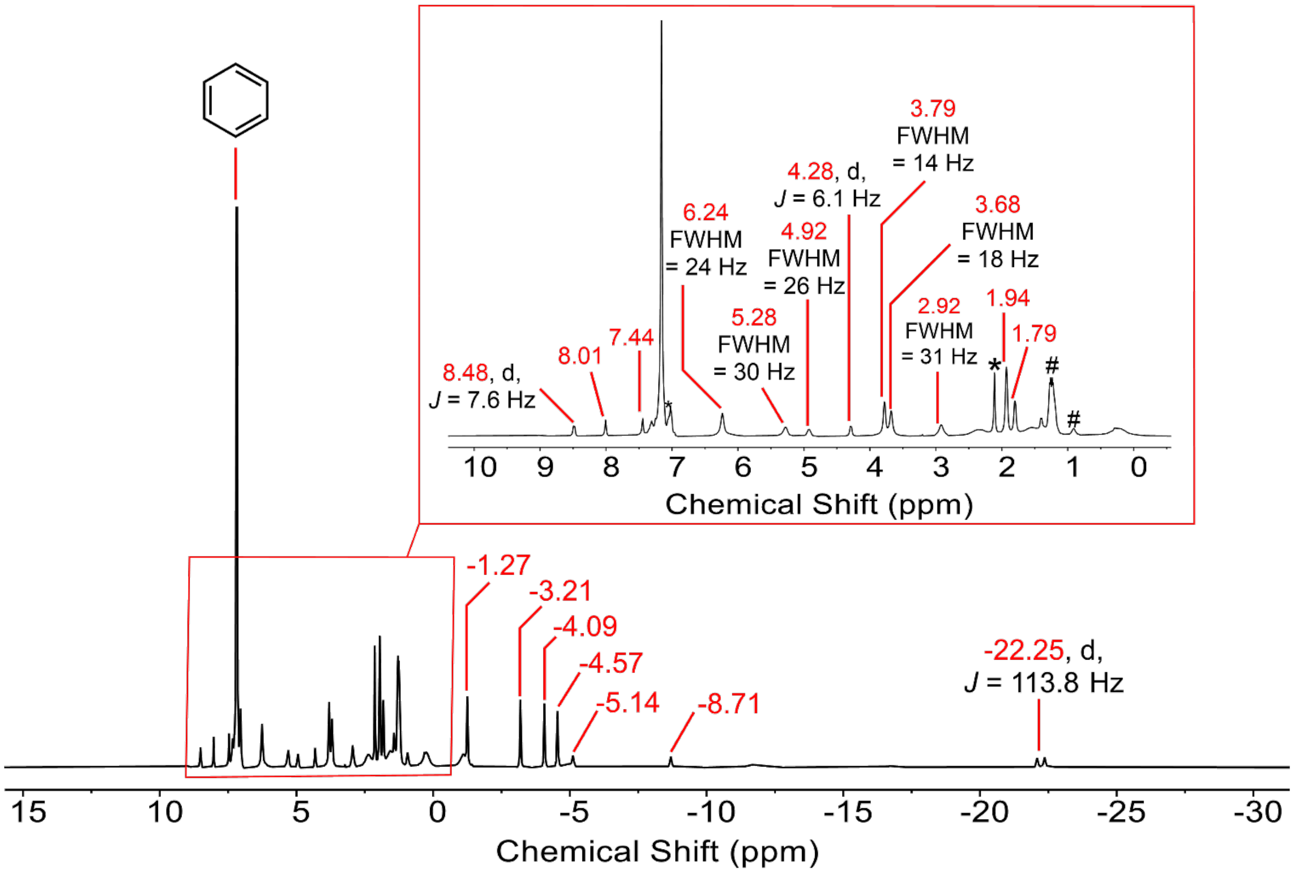

Figure S29. ^{1}H NMR spectrum of [U^{III}(SAr^{iPr6})(BH$_4$)$_2$] (**4a**) and [{U^{III}(SAr^{iPr6})(BH$_4$)}$_2$(μ-B$_2$H$_6$)] (**4b**) in *d*$_6$-benzene between +15 and –30 ppm. * and # denotes residual toluene and *n*-hexane respectively.

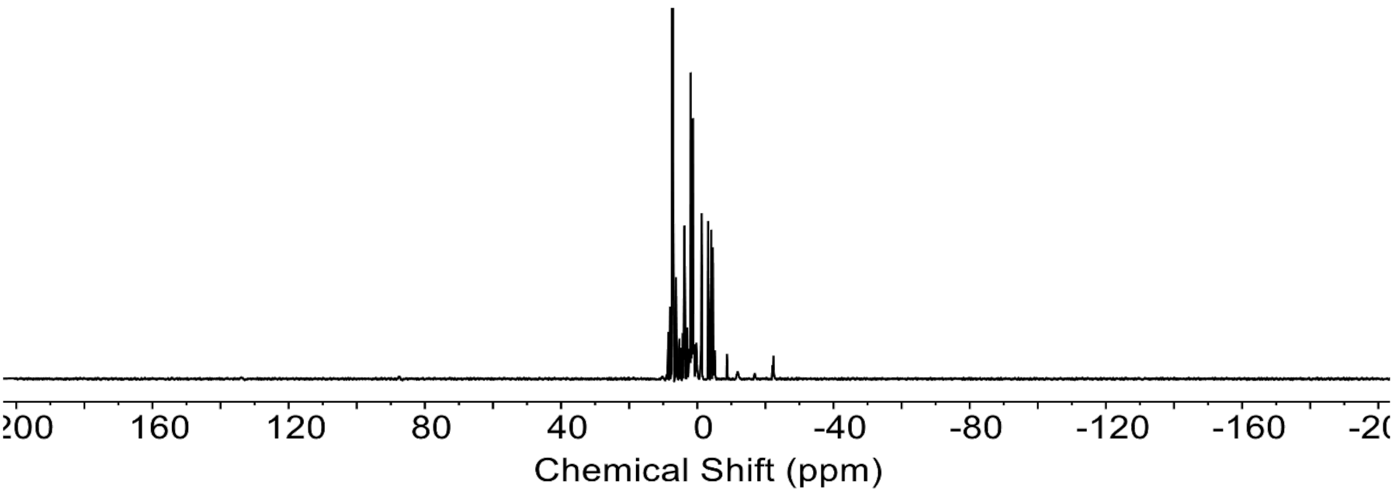

Figure S30. ^{1}H NMR spectrum of [U^{III}(SAr^{iPr6})(BH$_4$)$_2$] (**4a**) and [{U^{III}(SAr^{iPr6})(BH$_4$)}$_2$(μ-B$_2$H$_6$)] (**4b**) in *d*$_6$-benzene showing the whole scan range (+200 to –200 ppm).

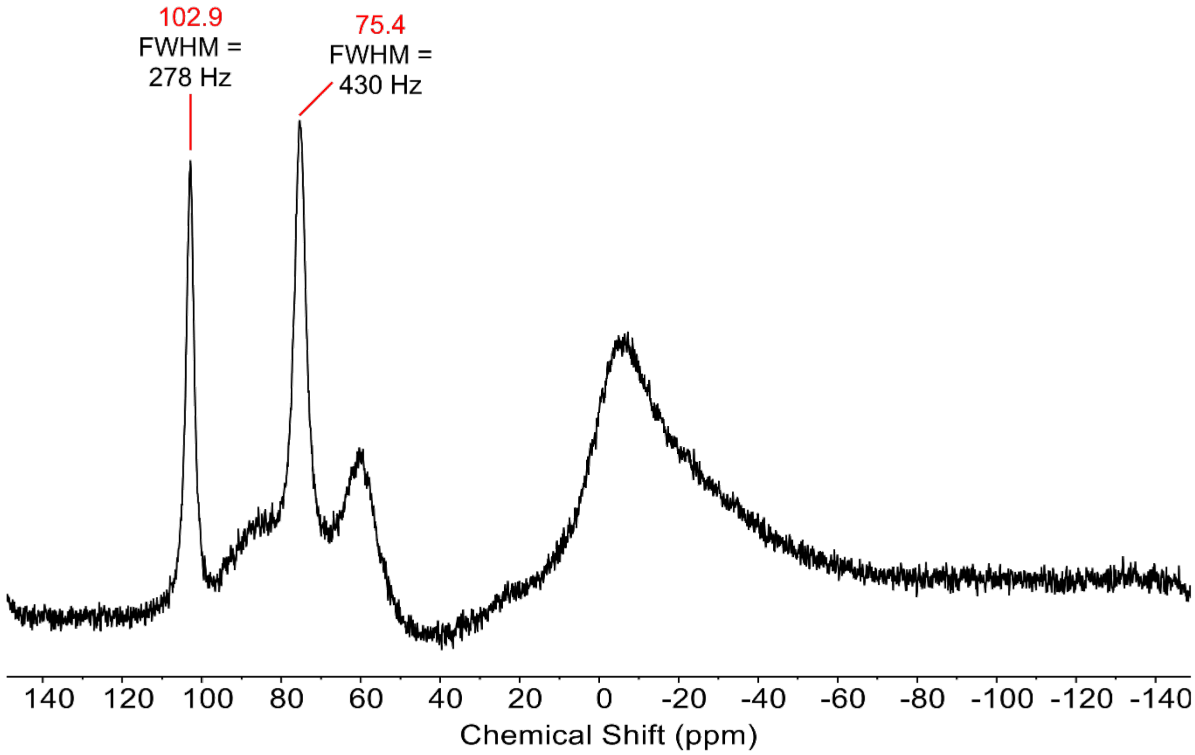

Figure S31. ^{11}B NMR spectrum of [U^{III}(SAr^{iPr6})(BH_4)$_2$] (**4a**) and [{U^{III}(SAr^{iPr6})(BH_4)}$_2$(μ-B_2H_6)] (**4b**) in *d*$_6$-benzene. The feature between 0 and –40 ppm is due to the borosilicate NMR tube.

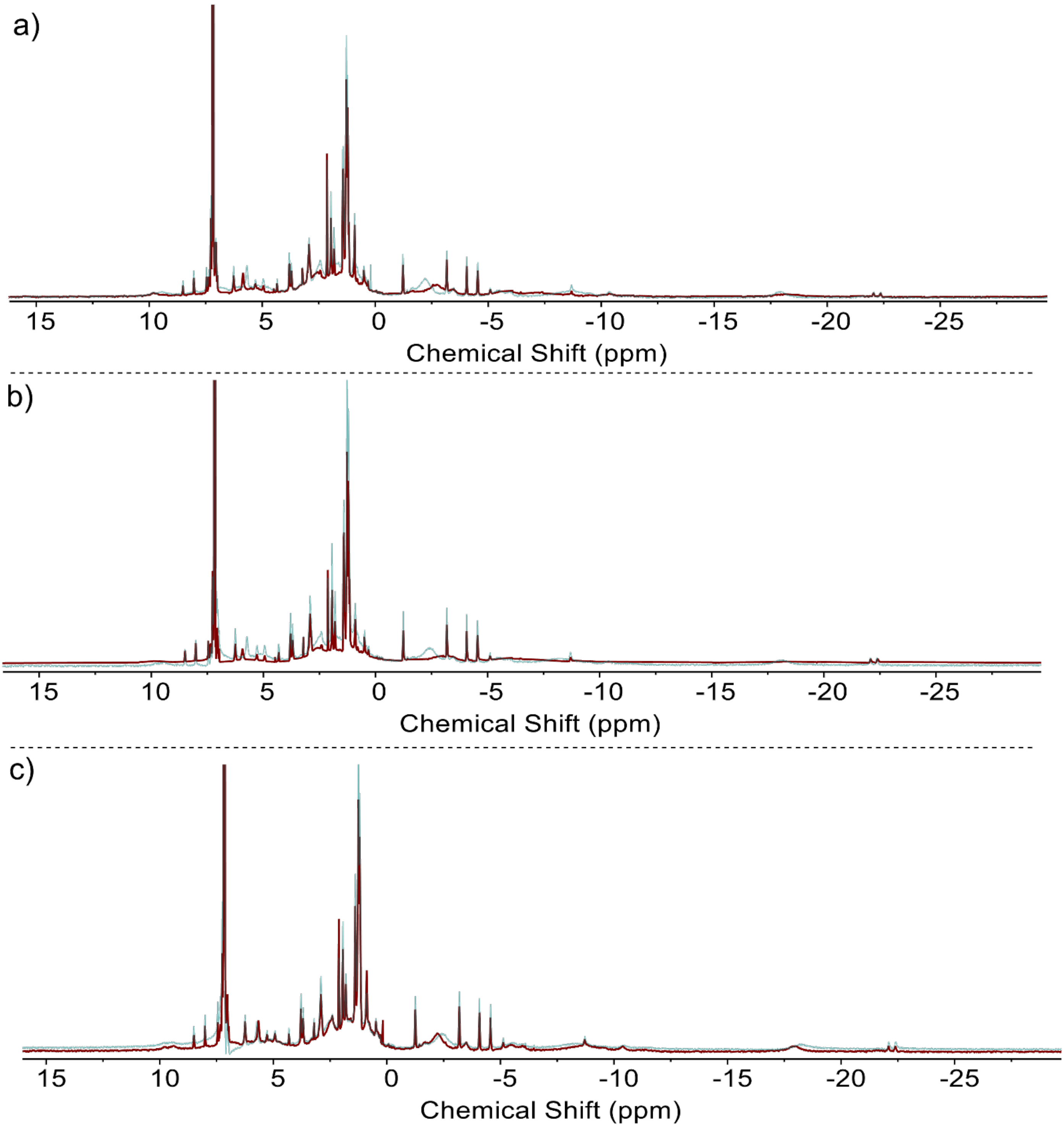

Figure S32. From the reaction of $[U^{III}(BH_4)_3(toluene)]$ + 1 $KSAr^{iPr6}$. ^1H NMR spectra in d_6-benzene between +15 and –30 ppm are shown for: **Panel a**) crop 1 of dark red crystals, from which the structure of **4a** was collected (red: immediately after dissolution at room temperature, blue: after 18 hours at 60 °C); **Panel b**) crop 2 of dark red crystals, from which the structure of **4b** was collected, (red: immediately after dissolution at room temperature, blue: after 18 hours at 60 °C); **Panel c**) after 18 hours at 60 °C (red: crop 1, blue: crop 2). ^1H NMR spectra show no changes between the two crops, and not after elevated temperatures suggesting that **4a** and **4b** are present in both crops.

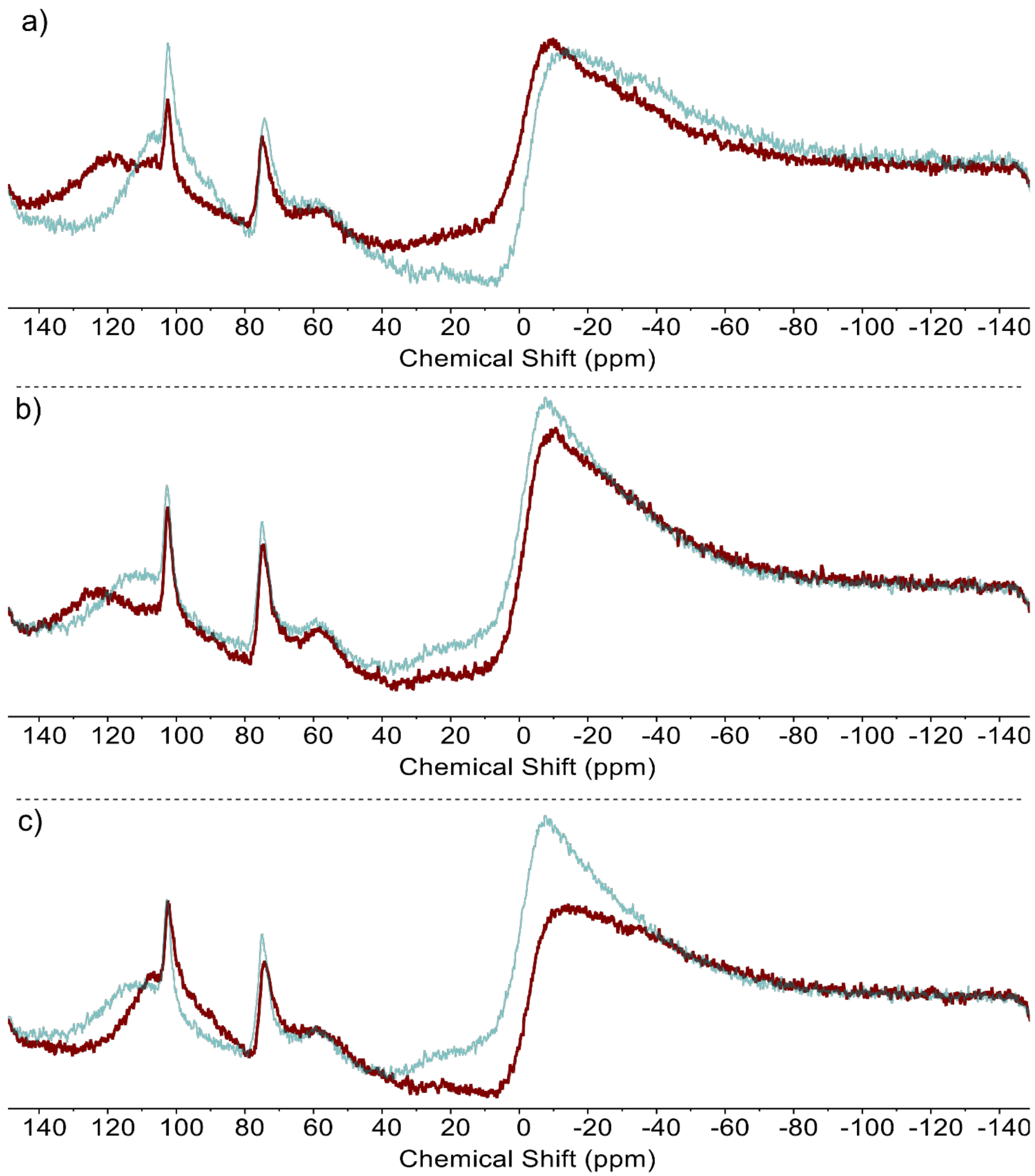

Figure S33. From the reaction of [$U^{III}(BH_4)_3$(toluene)] + 1 $KSAr^{iPr6}$: ^{11}B NMR spectrum in d_6-benzene between +150 and –150 ppm are shown for: **Panel a**) crop 1 of dark red crystals, from which the structure of **4a** was collected (red: immediately after dissolution at room temperature, blue: after 18 hours at 60 °C); **Panel b**) crop 2 of dark red crystals, from which the structure of **4b** was collected, (red: immediately after dissolution at room temperature, blue: after 18 hours at 60 °C); **Panel c**) after 18 hours at 60 °C (red: crop 1, blue: crop 2). ^{11}B NMR spectra show no changes between the two crops, and not after elevated temperatures suggesting that **4a** and **4b** are present in both crops.

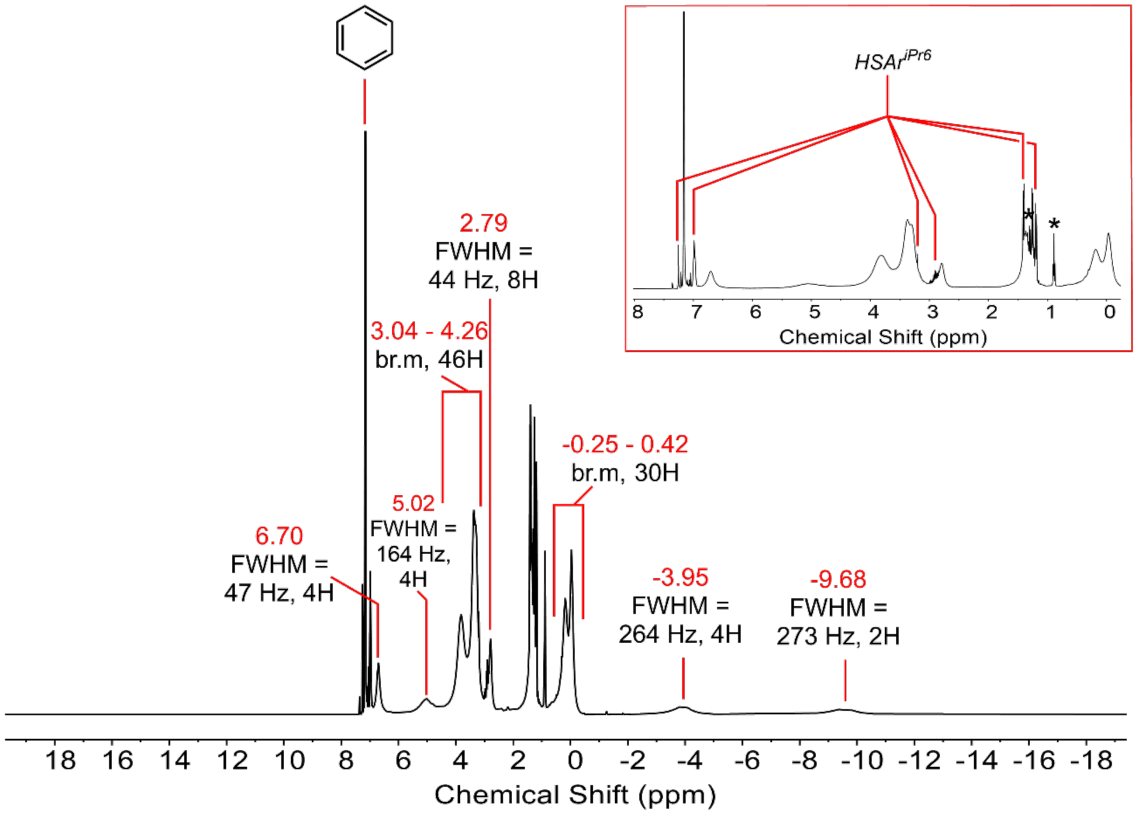

Figure S34. ^{1}H NMR spectrum of [$U^{III}(SAr^{iPr6})_2(BH_4)$] (**5**) in d_6-benzene between +20 and –20 ppm. * denotes residual *n*-hexane.

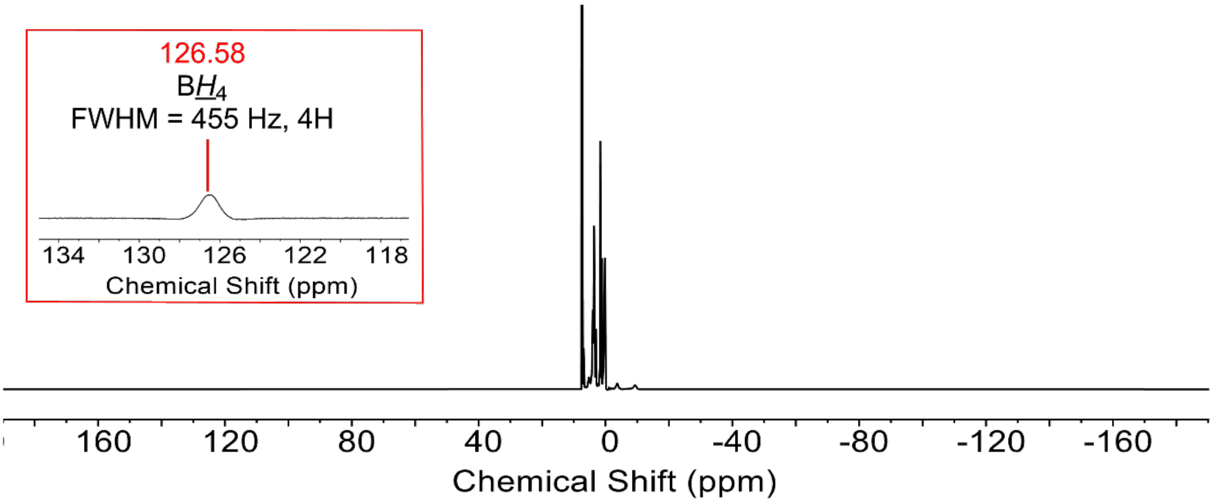

Figure S35. ^{1}H NMR spectrum of [$U^{III}(SAr^{iPr6})_2(BH_4)$] (**5**) in d_6-benzene showing the whole scan range (+200 to –200 ppm).

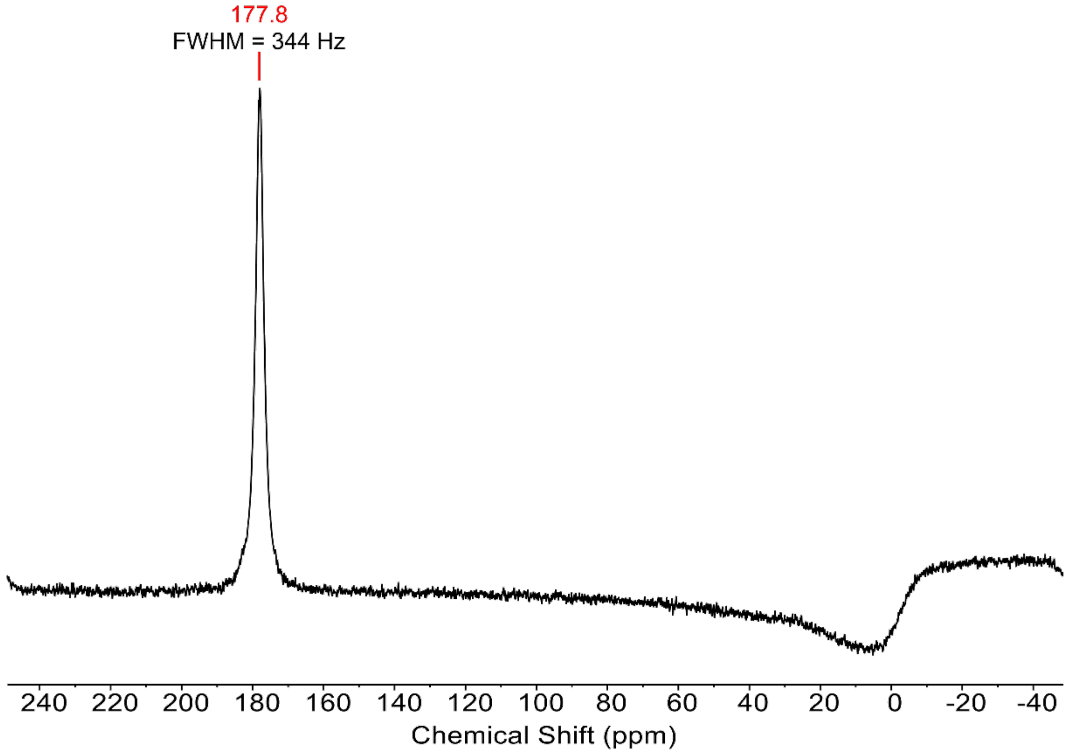

Figure S36. ^{11}B NMR spectrum of [U^{III}(SAr^{iPr6})$_2$(BH_4)] (**5**) in d_6-benzene. The feature between 0 and –40 ppm is due to the borosilicate NMR tube.

Magnetic moments determined by NMR spectroscopy (Evans method)

Table S17. Data for the determination of the magnetic moments of complexes **1**, and **2–5**.

Sample / peak	μ_{eff} / B.M mol^{-1} (per U)	[a] Sample mass / g	Solvent mass / g	M_r / g mol^{-1}	[b] Δ peak / Hz
[$U^{IV}(SAr^{iPr6})_2(Cl)_2$] (**1**)	2.664(10)	0.0146	0.5381	1336.62	74.62
[U^{IV}(μ-SAr^{iPr6})$(BH_4)_2$(μ-BH_4)(μ^3-BH_4)K]$_2$ (**2**)	1.707(5)	0.0158	0.5848	850.35	40.29
[U^{III}(H_3B·SAr^{iPr6}-κ*S*,*H*,*H*)$(BH_4)_2$] (**3**)	2.146(8)	0.0145	0.5532	795.39	80.49
[$U^{III}(SAr^{iPr6})_2(BH_4)$] (**5**)	2.780(11)	0.0146	0.5494	1280.57	85.97

[a] The small masses engender large errors in this methodology, the results should be cautiously interpreted along with other data. [b] Spectrometer frequency 400.070 MHz. Diamagnetic correction of M_r / −2,000,000 applied. ρd_6-benzene = 0.950 g mL^{-1}.

S5. ATR-IR Spectra

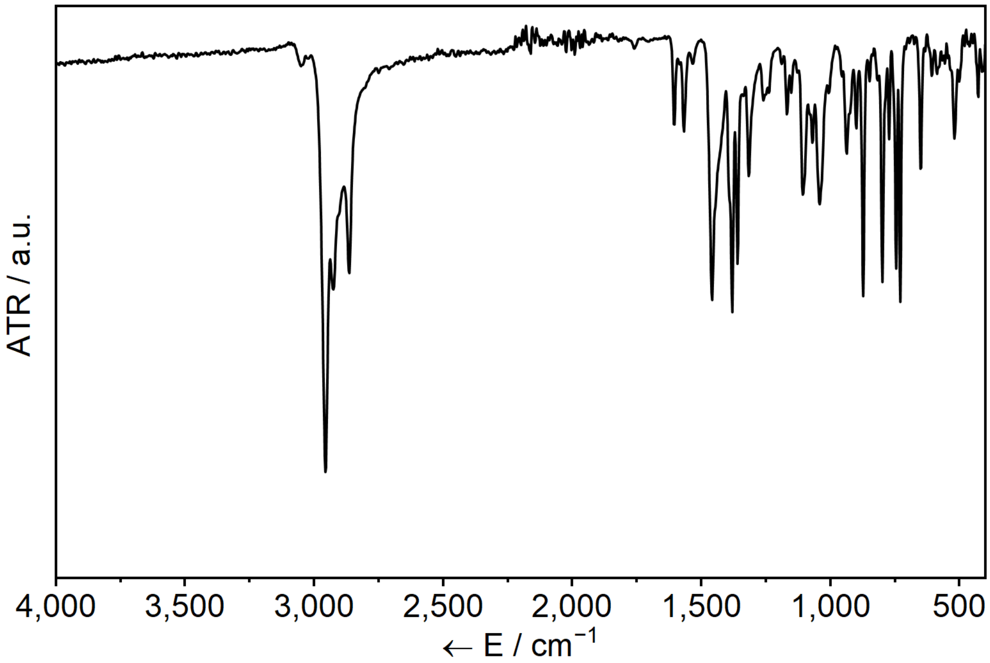

Figure S37. ATR-IR spectrum of microcrystalline [U^{IV}(SAr^{iPr6})$_2$(Cl)$_2$] (**1**).

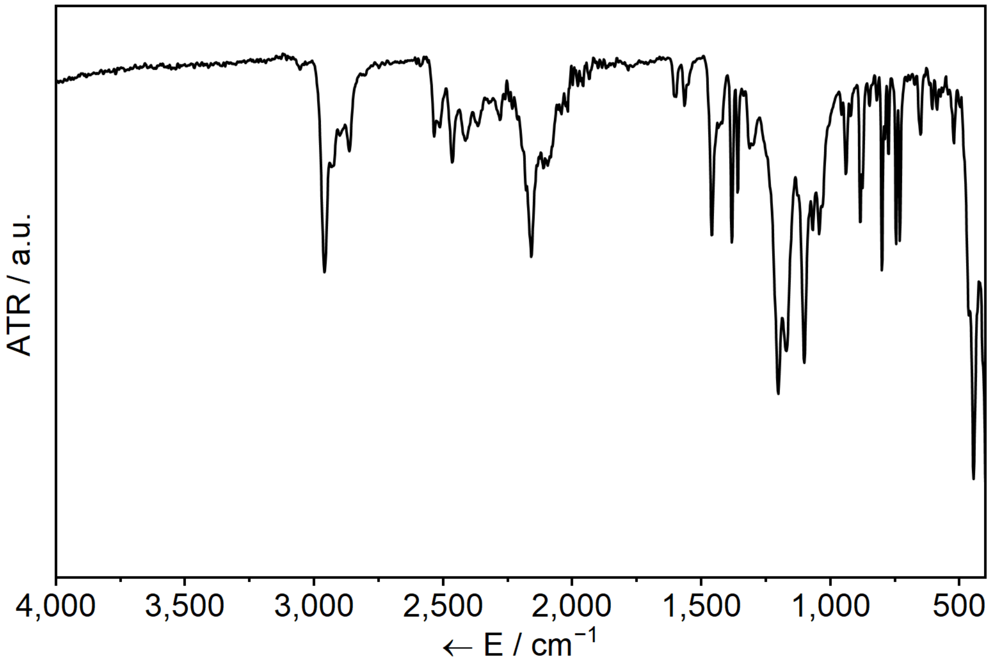

Figure S38. ATR-IR spectrum of microcrystalline [U^{IV}(μ-SAr^{iPr6})(BH_4)$_2$(μ-BH_4)($μ^3$-BH_4)K]$_2$ (**2**) obtained by crushing single crystals.

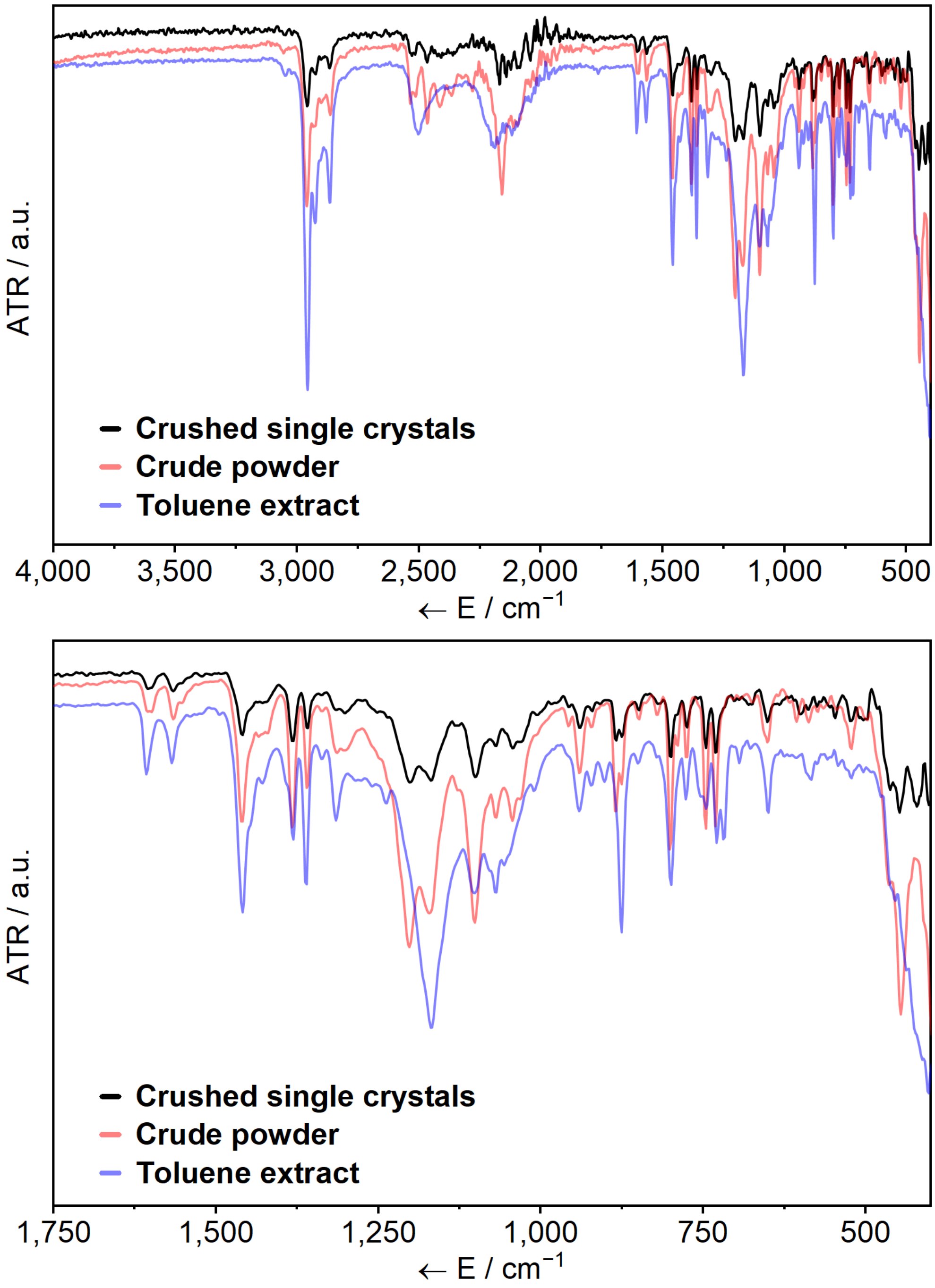

Figure S39. Combined ATR-IR spectra of crushed single crystals of complex **2** (black), complex **2** isolated as a crude powder (red), and the toluene supernatant from washing the crude material (blue). The top spectrum shows the full range collected (4,000–400 cm^{-1}), the bottom spectrum shows between 1,750–400 cm^{-1}.

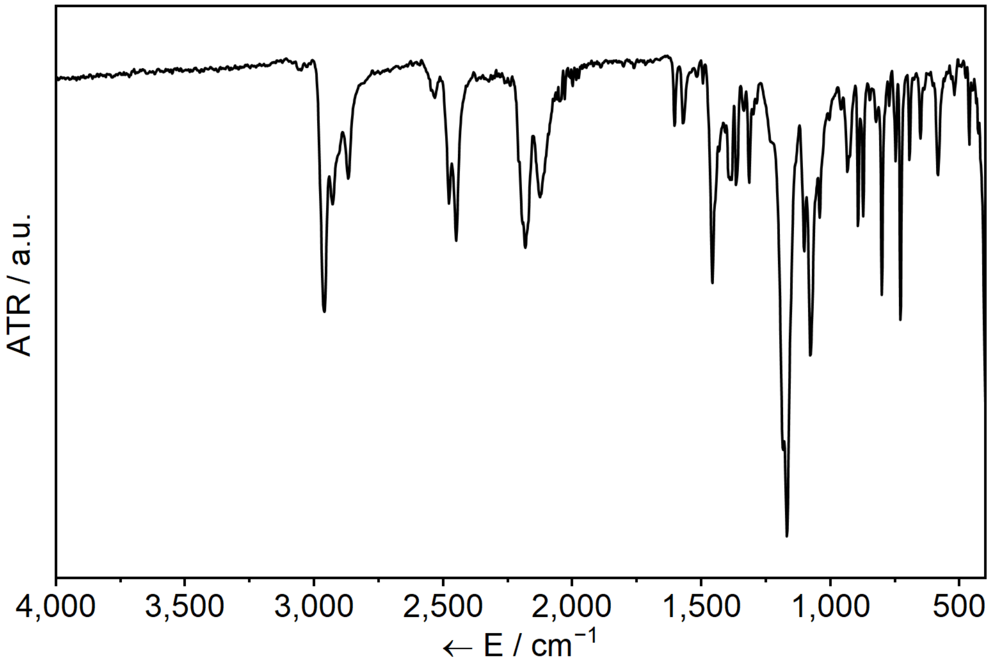

Figure S40. Crystalline ATR-IR spectrum of [U^{III}($H_3B{\cdot}SAr^{iPr6}$-κ*S*,*H*,*H*)($(BH_4)_2$)] (**3**).

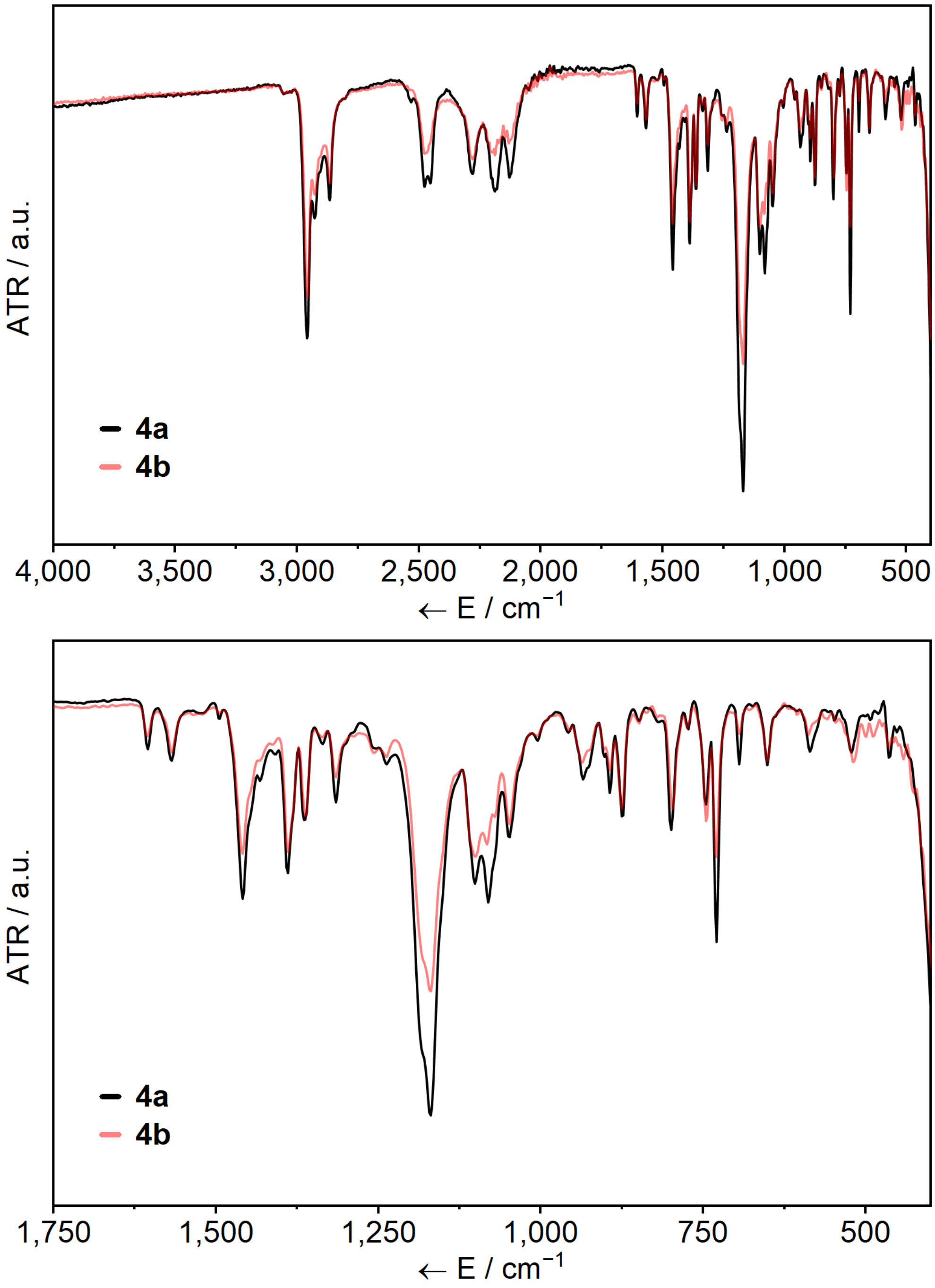

Figure S41. Combined ATR-IR spectra of both crystalline crops from the reaction that produced [U^{III}(SAr^{iPr6})(BH$_4$)$_2$] (**4a**) and [{U^{III}(SAr^{iPr6})(BH$_4$)}$_2$(μ-B$_2$H$_6$)] (**4b**). The top spectrum shows the full range collected (4,000–400 cm^{-1}), the bottom spectrum shows between 1,750–400 cm^{-1}.

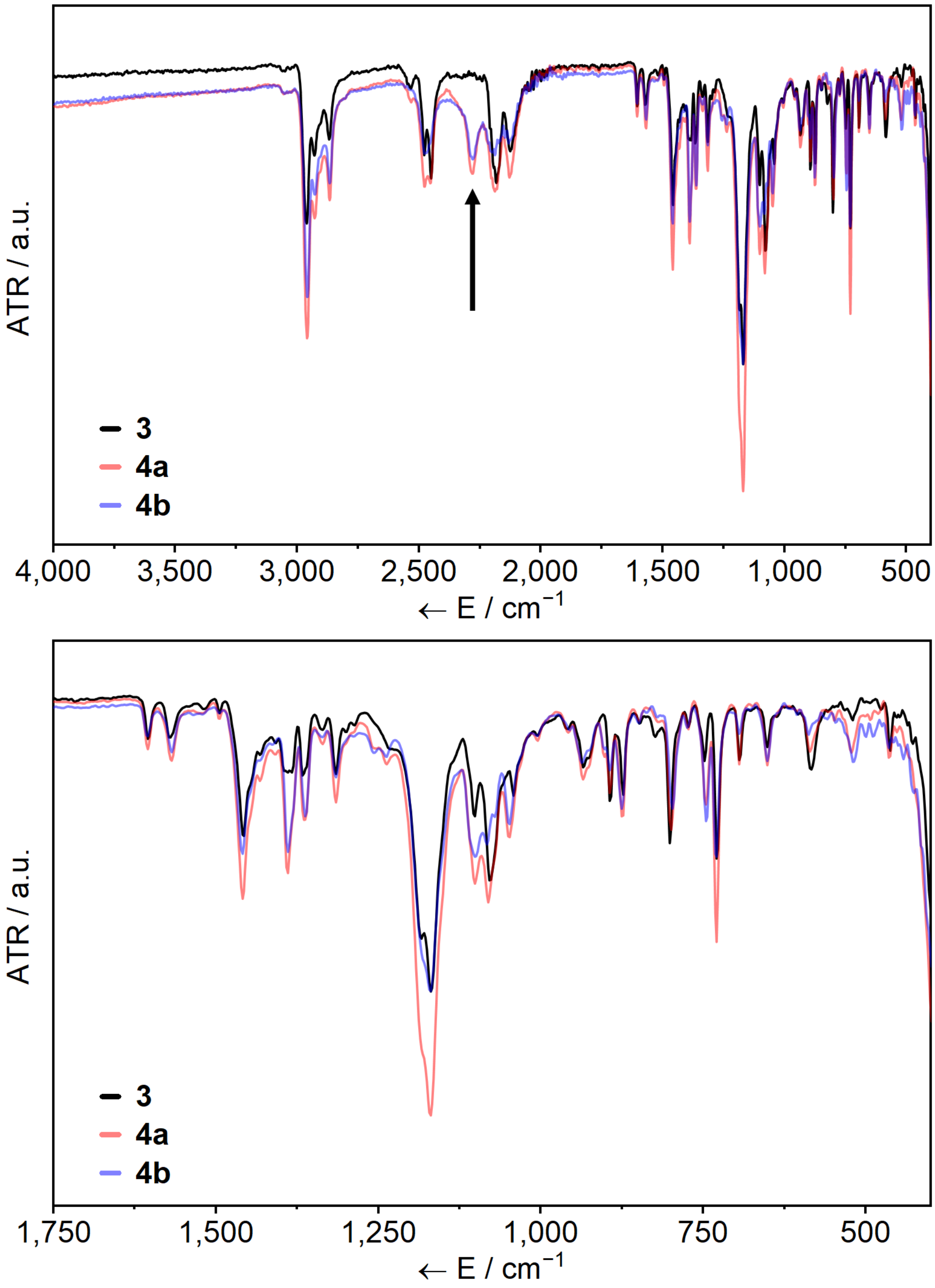

Figure S42. Combined ATR-IR spectra of [U^{III}($H_3B\cdot SAr^{iPr6}$-κ*S*,*H*,*H*)(BH_4)$_2$] (**3**), and both crystalline crops from the reaction that produced [U^{III}(SAr^{iPr6})(BH_4)$_2$] (**4a**) and [{U^{III}(SAr^{iPr6})(BH_4)}$_2$(μ-B_2H_6)] (**4b**). The top spectrum shows the full range collected (4,000–400 cm^{-1}), the bottom spectrum shows between 1,750–400 cm^{-1}. The black arrow denotes the major spectral difference between **3**, and **4a**/**4b**.

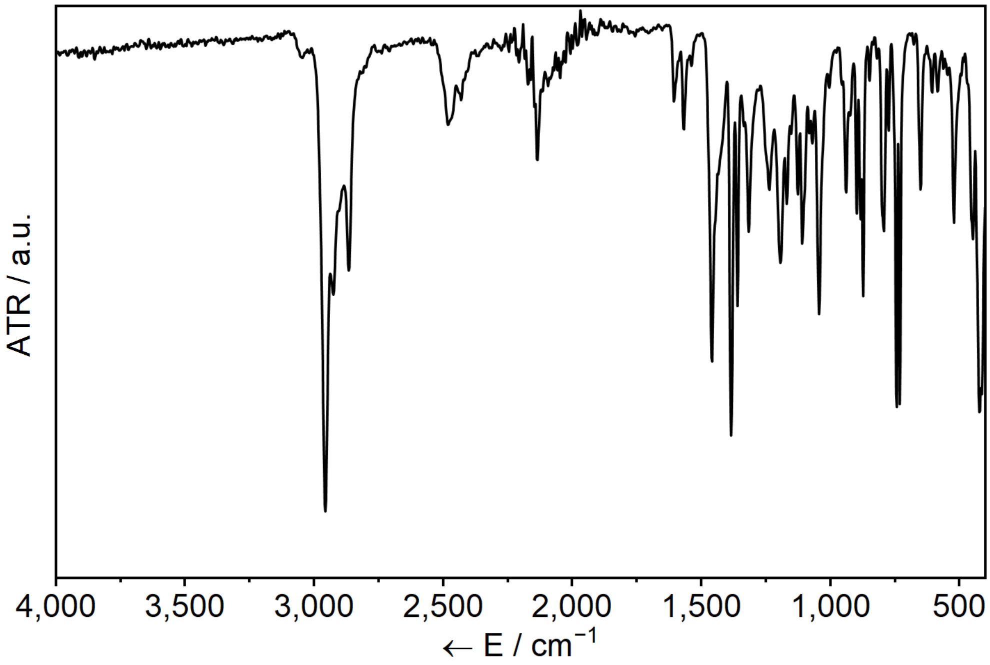

Figure S43. ATR-IR spectrum of microcrystalline [U^{III}(SAr^{iPr6})$_2$(BH_4)] (**5**).

S6. UV-Vis-NIR Spectra

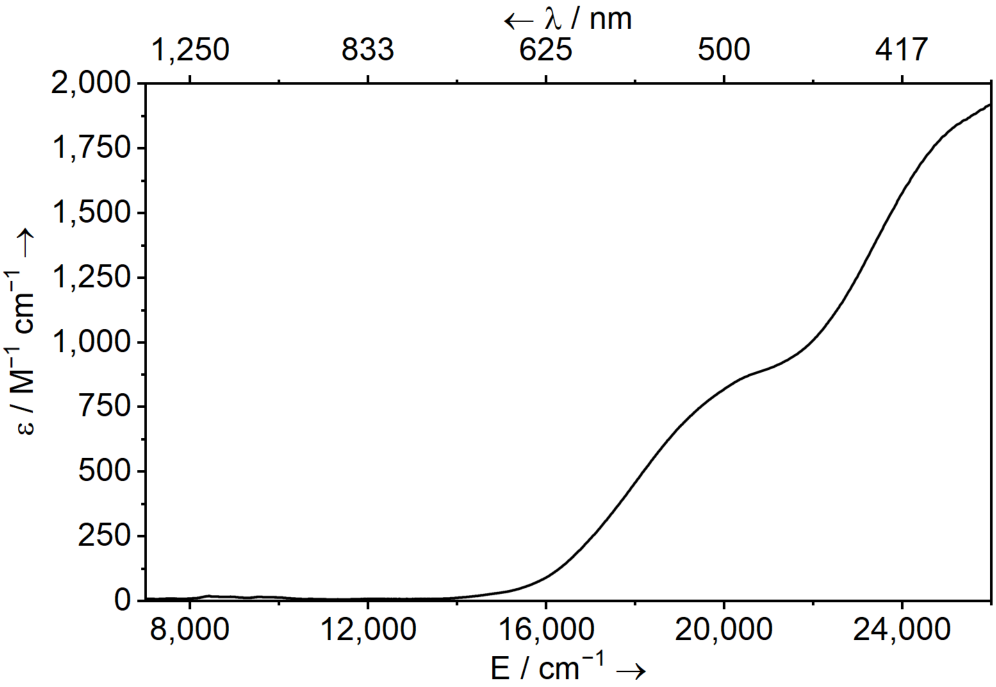

Figure S44. Solution UV-Vis-NIR spectrum of $[U^{IV}(SAr^{iPr6})_2(Cl)_2]$ (**1**) (1.0 mM) in Et_2O shown between 7,000–26,000 cm^{-1} (1,429–385 nm) at ambient temperature.

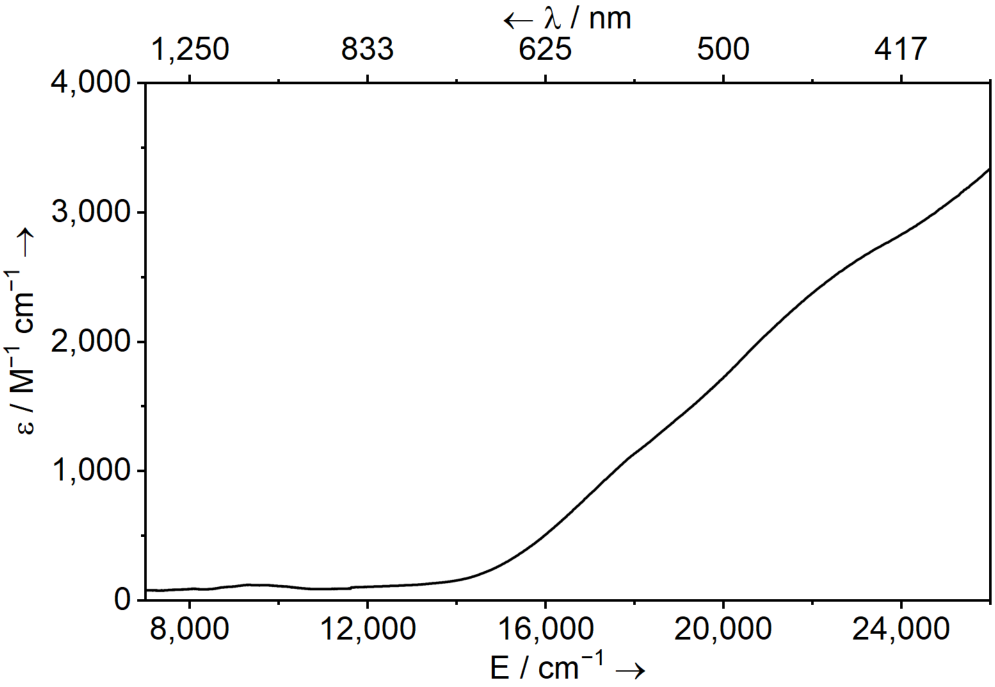

Figure S45. Solution UV-Vis-NIR spectrum of $[U^{IV}(\mu\text{-}SAr^{iPr6})(BH_4)_2(\mu\text{-}BH_4)(\mu^3\text{-}BH_4)K]_2$ (**2**) (0.5 mM) in Et_2O shown between 7,000–26,000 cm^{-1} (1,429–385 nm) at ambient temperature.

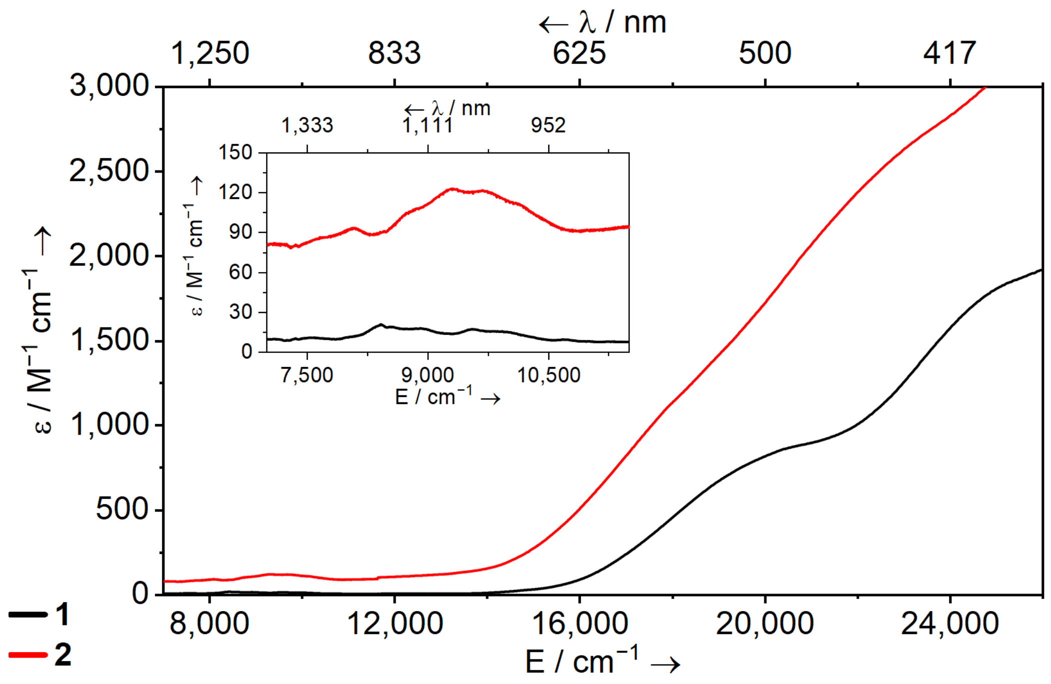

Figure S46. Combined solution UV-Vis-NIR spectra of both U(IV) complexes, **1** (1.0 mM), and **2** (0.5 mM) in Et_2O shown between 7,000–26,000 cm^{-1} (1,429–385 nm) at ambient temperature.

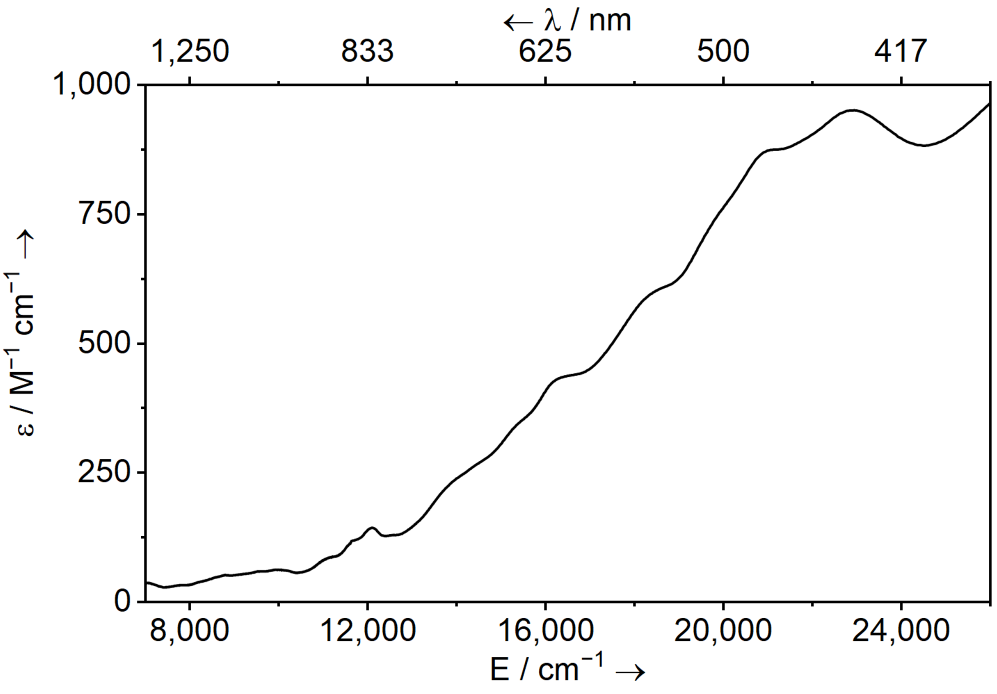

Figure S47. Solution UV-Vis-NIR spectrum of [U^{III}(H_3B·SAr^{iPr6}-κ*S*,*H*,*H*)($(BH_4)_2$)] (**3**) (1.0 mM) in Et_2O shown between 7,000–26,000 cm^{-1} (1,429–385 nm) at ambient temperature.

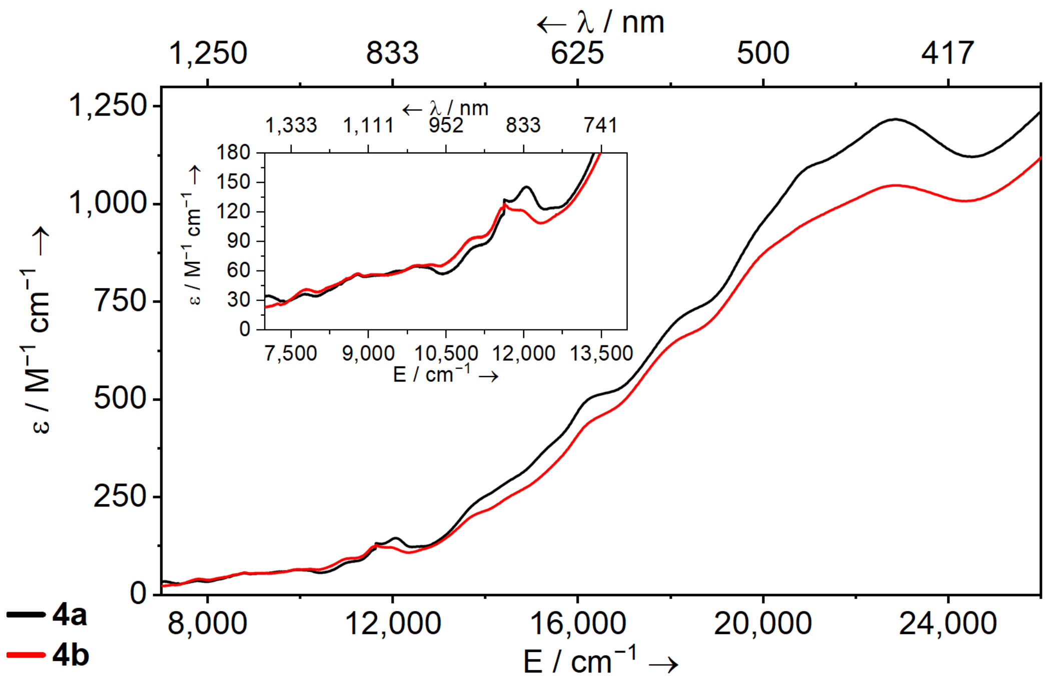

Figure S48. Combined solution UV-Vis-NIR spectra of both crystalline crops from the reaction that produced [U^{III}(SAr^{iPr6})(BH_4)$_2$] (**4a**) and [{U^{III}(SAr^{iPr6})(BH_4)}$_2$(μ-B_2H_6)] (**4b**) (*ca*. 1.0 mM) in Et_2O shown between 7,000–26,000 cm^{-1} (1,429–385 nm) at ambient temperature. The concentration is approximate due to the mass difference (H_2) between **4a** and **4b**, but in practice other sources of error are much larger.

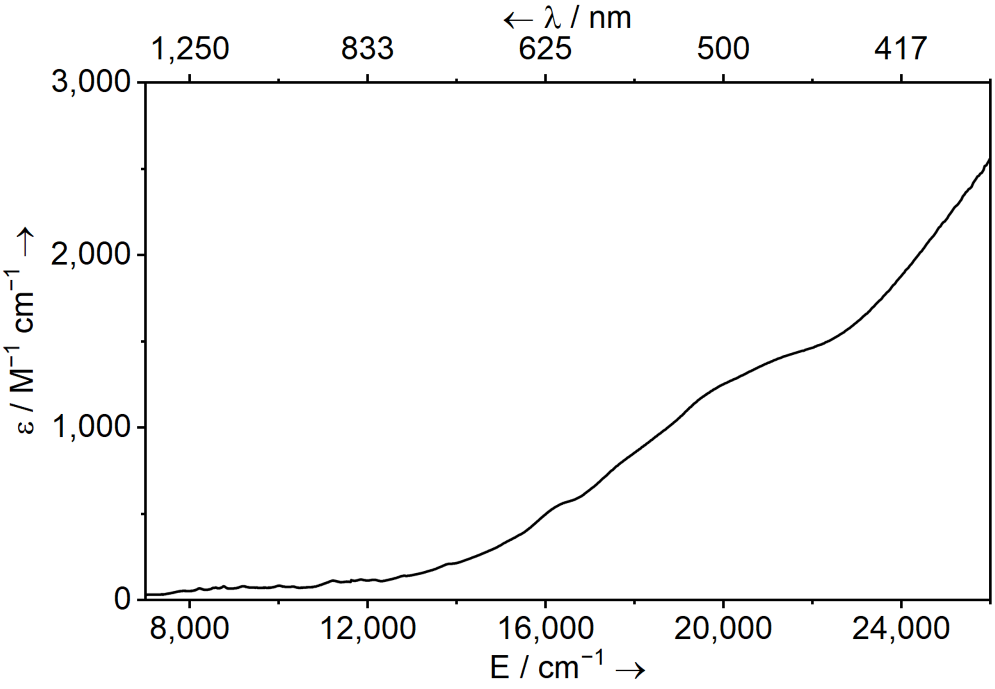

Figure S49. Solution UV-Vis-NIR spectrum of [U^{III}(SAr^{iPr6})$_2$(BH_4)] (**5**) (1.0 mM) in Et_2O shown between 7,000–26,000 cm^{-1} (1,429–385 nm) at ambient temperature.

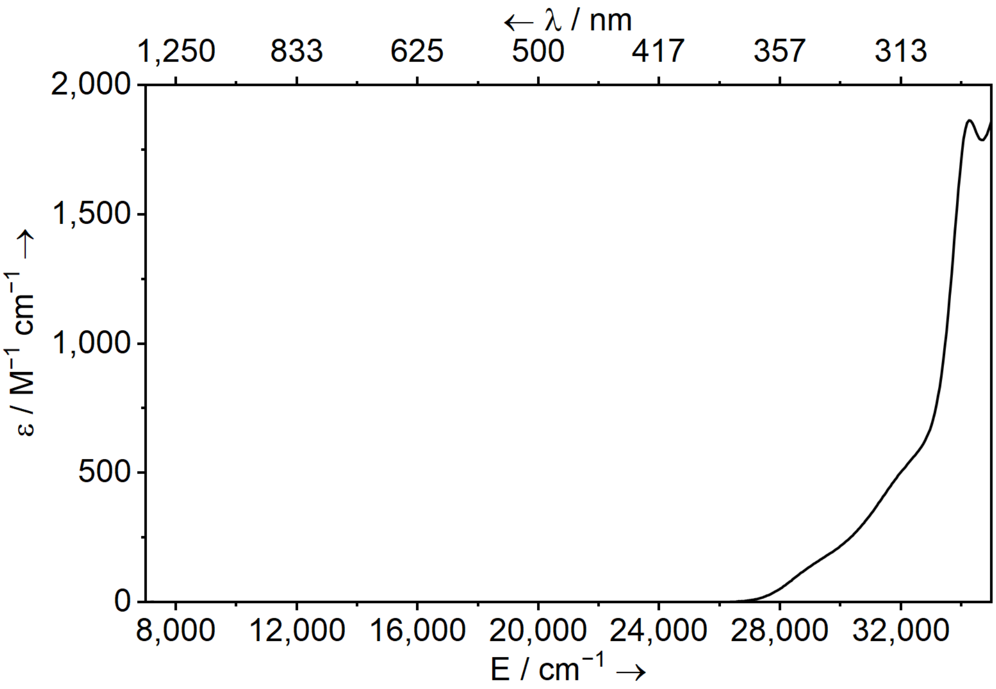

Figure S50. Solution UV-Vis-NIR spectrum of HSAr^{iPr6} (1.0 mM) in Et_2O shown between 7,000–35,000 cm^{-1} (1,429–286 nm) at ambient temperature.

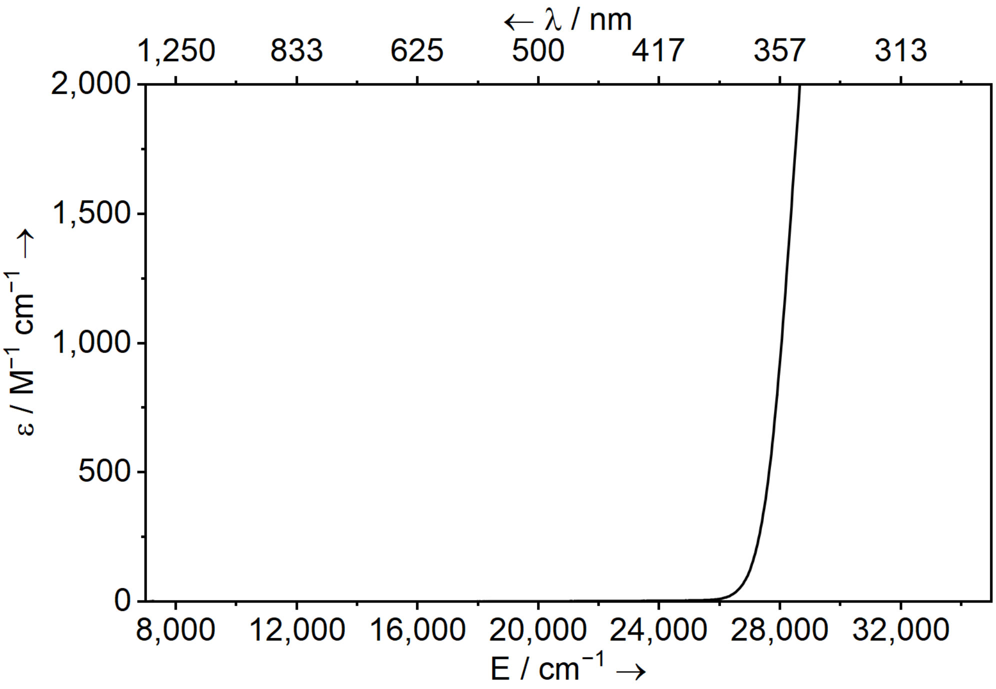

Figure S51. Solution UV-Vis-NIR spectrum of KSAr^{iPr6} (1.0 mM) in Et_2O shown between 7,000–35,000 cm^{-1} (1,429–286 nm) at ambient temperature.

S7. SQUID Magnetometry

Variable-temperature magnetic moment data were recorded in an applied direct current (DC) field of 0.1 Tesla on a Quantum Design MPMS3 superconducting quantum interference device magnetometer using recrystallized powdered samples. Measurements were performed in dc scan mode using 40 mm scan length and 6 s scan time. Samples were carefully checked for purity and data reproducibility between independently prepared batches. Samples were crushed with a mortar and pestle under an argon atmosphere and immobilized in an eicosane matrix within 400 MHz Wilmad borosilicate NMR tubes to prevent sample reorientation during measurements. The tube was flame-sealed under dynamic vacuum ($1x10^{-3}$ mbar) to a length of approximately 3 cm and mounted in the centre of a drinking straw, with the straw fixed to the end of an MPMS 3 sample rod. Care was taken to ensure complete thermalization of the sample before each data point was measured by employing delays at each temperature point as well as a slow cooling rate (5 K/min from 300 to 100 K; 1 K/min from 100 to 1.8 K). The sample was held at 1.8 K for 60 minutes before isothermal magnetization measurements to account for slow thermal equilibration of the sample. Diamagnetic corrections were applied using tabulated Pascal constants. Measurements were corrected for the effect of the blank sample holders (flame sealed Wilmad NMR tube and straw) and eicosane matrix.

Table S18. Mass of complex and eicosane used in samples analyzed by SQUID magnetometry.

Complex	Chemical formula [A]	M_r / g mol^{-1}	Sample mass / g	Eicosane mass / g
$[U^{IV}(SAr^{iPr6})_2(Cl)_2]$ (**1**)	$C_{72}H_{98}Cl_2S_2U$	1336.62	29.6	16.0
$[U^{IV}(\mu\text{-}SAr^{iPr6})(BH_4)_2(\mu\text{-}BH_4)(\mu^3\text{-}BH_4)K]_2$ (**2**)	$C_{72}H_{130}B_8K_2S_2U_2$	1700.61	31.6	18.2
$[U^{III}(H_3B{\cdot}SAr^{iPr6}\text{-}\kappa S,H,H)(BH_4)_2]$ (**3**)	$C_{36}H_{60}B_3SU$	795.39	27.8	19.8
$[U^{III}(SAr^{iPr6})(BH_4)_2]$ (**4a**) / $[\{U^{III}(SAr^{iPr6})(BH_4)\}_2(\mu\text{-}B_2H_6)]$ (**4b**) [B]	$C_{36}H_{57}B_2SU$ / $C_{72}H_{112}B_4S_2U_2$	781.56 / 1561.11	28.0	14.8
$[U^{III}(SAr^{iPr6})_2(BH_4)]$ (**5**)	$C_{72}H_{102}BS_2U$	1280.57	27.5	18.2

[A] Note that the chemical formulae in Table S18 do not necessarily align with those in the crystallographic tables (Table S4 to Table S6) as the latter include lattice solvents, whereas the former do not. This is because elemental analysis and NMR spectroscopic data suggests complete or partial removal of lattice solvents under vacuum prior to preparation of the samples for SQUID magnetometry. [B] In the following sections we refer to both complex **4a** and **4b** together as our ATR-IR and NMR spectroscopic data suggests crystalline batches of each are contaminated with both complexes. We find no signs of coupling between the two U-atoms present in the **4b** content, and their mass difference is just that of H_2, and so is well within the error of the measurements.

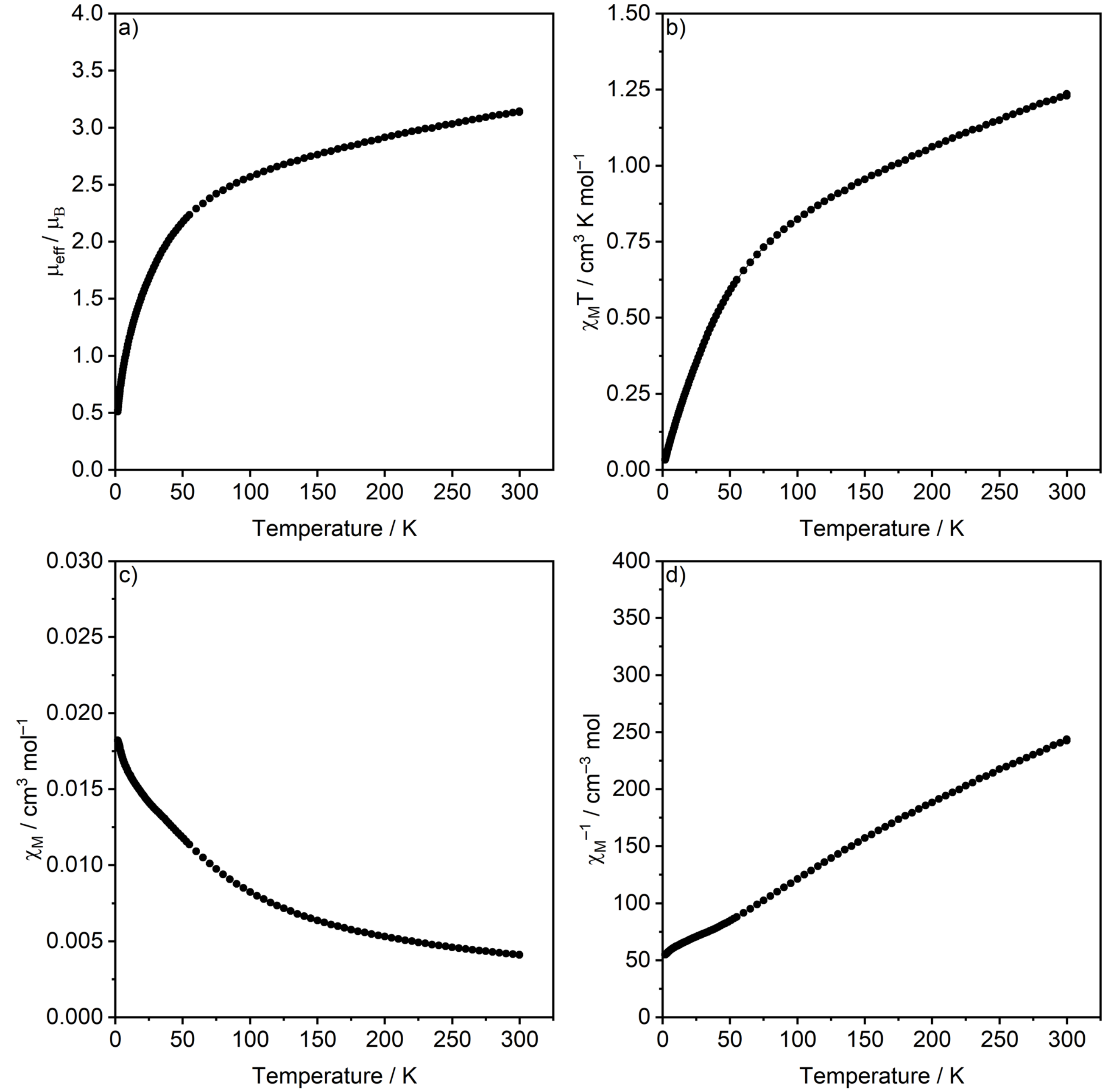

Figure S52. Variable-temperature SQUID magnetometry of **1** over the temperature range 1.8–300K: a) μ_{eff} vs T; b) χT vs T; c) χ vs T; d) χ^{-1} vs T.

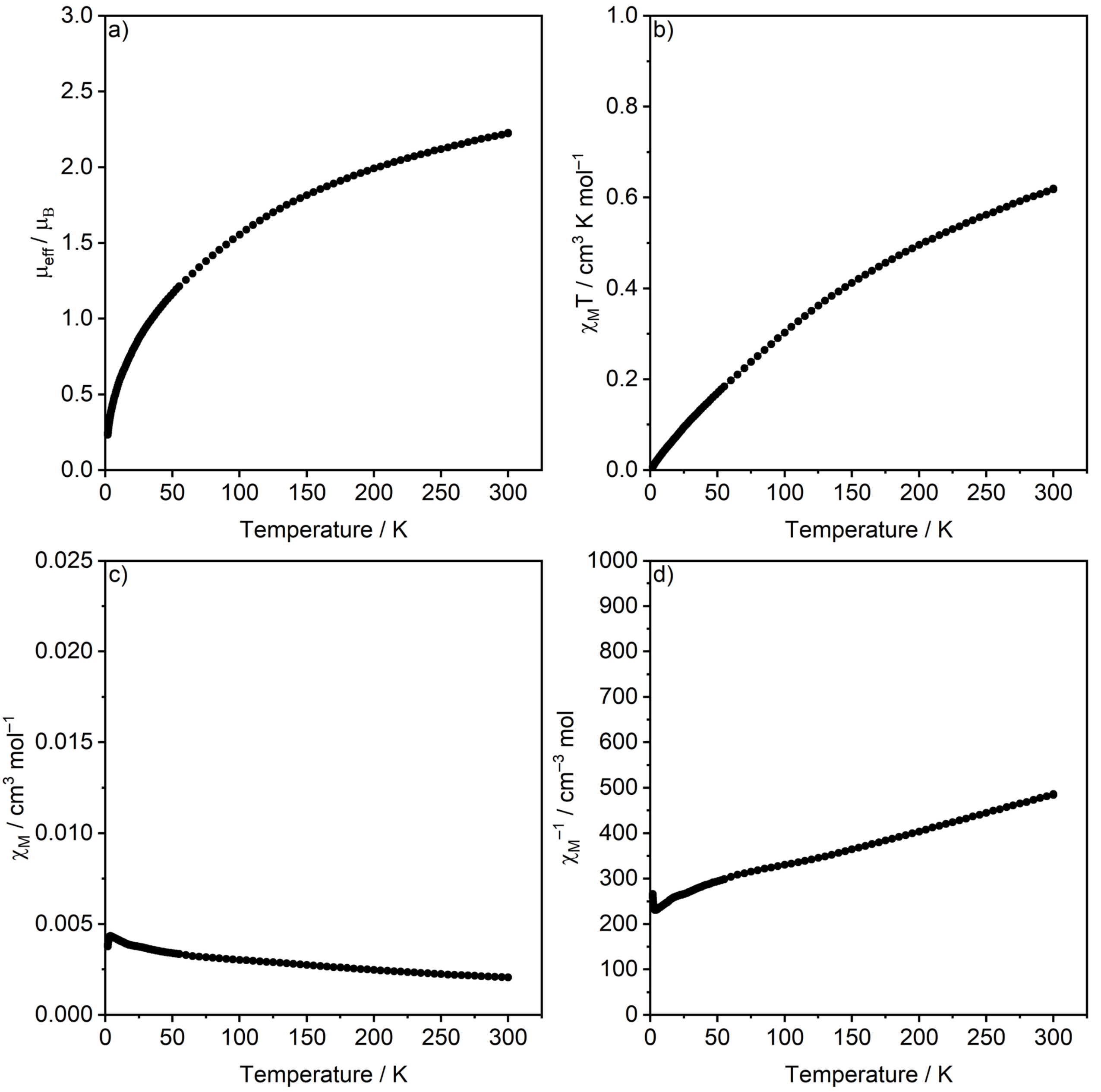

Figure S53. Variable-temperature SQUID magnetometry of **2** over the temperature range 1.8–300K: a) μ_{eff} vs T; b) χT vs T; c) χ vs T; d) χ^{-1} vs T.

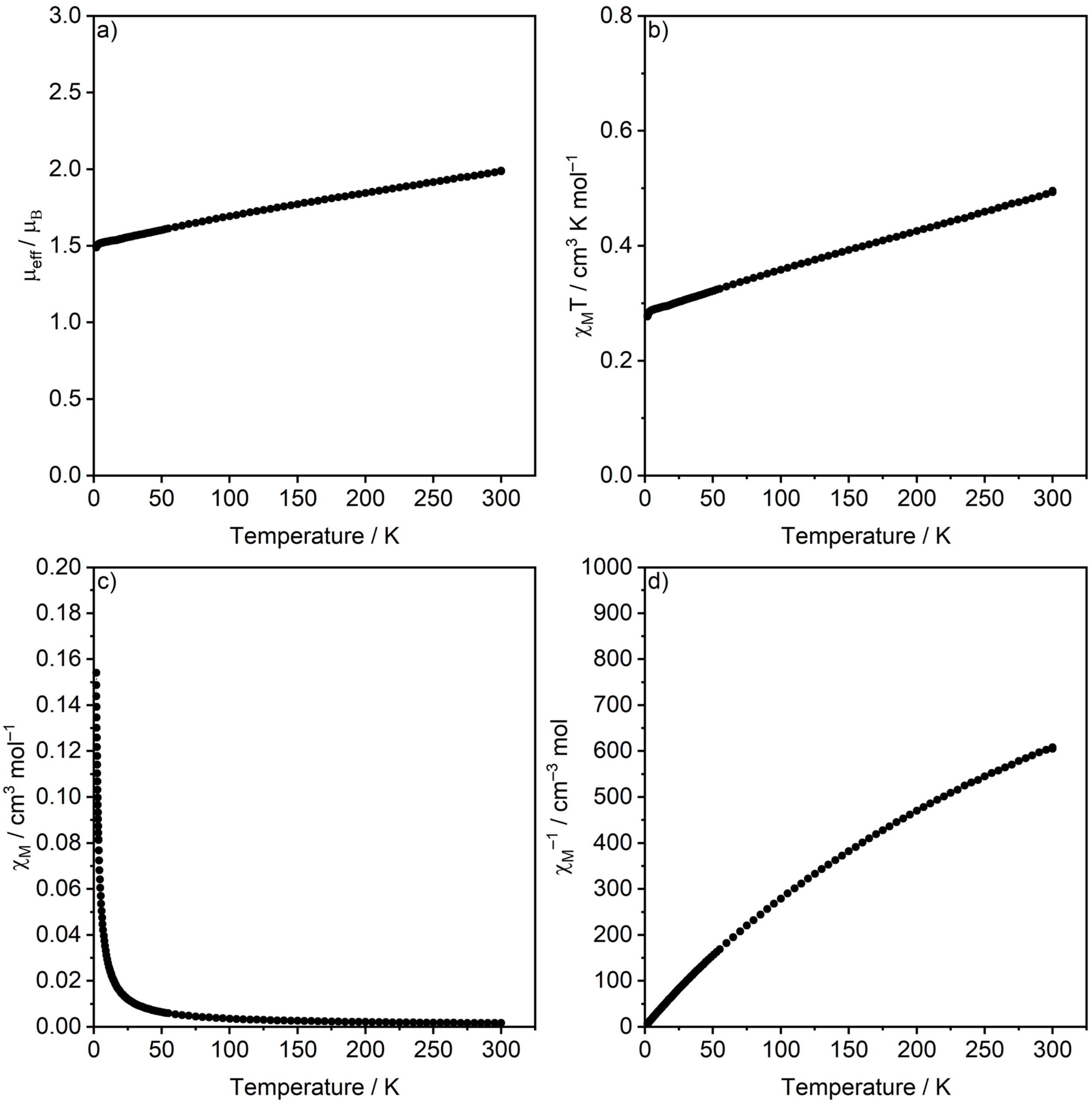

Figure S54. Variable-temperature SQUID magnetometry of **3** over the temperature range 1.8–300K: a) μ_{eff} vs T; b) χT vs T; c) χ vs T; d) χ^{-1} vs T.

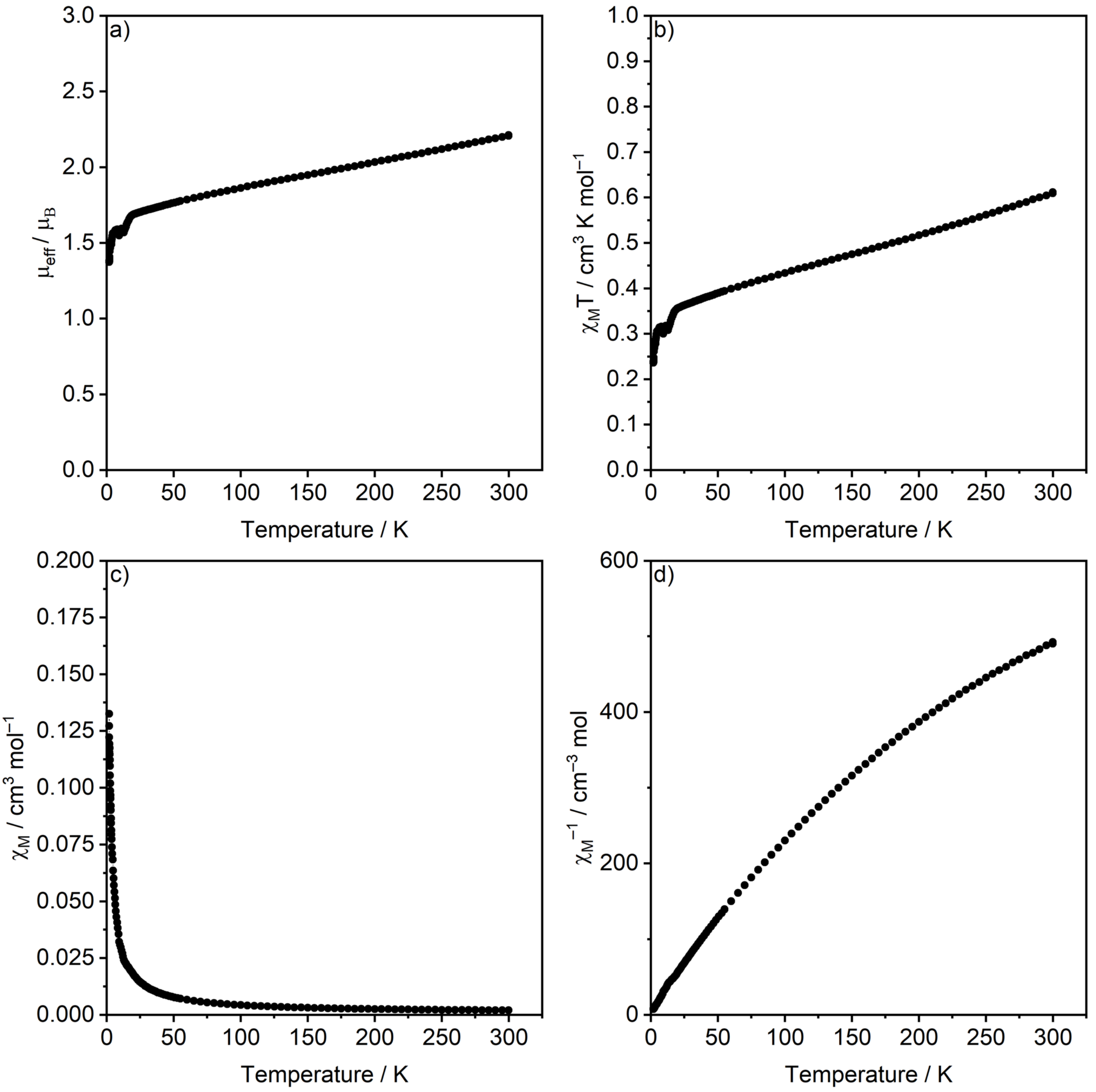

Figure S55. Variable-temperature SQUID magnetometry of **4a**/**b** over the temperature range 1.8–300K: a) μ_{eff} vs T; b) χT vs T; c) χ vs T; d) χ^{-1} vs T.

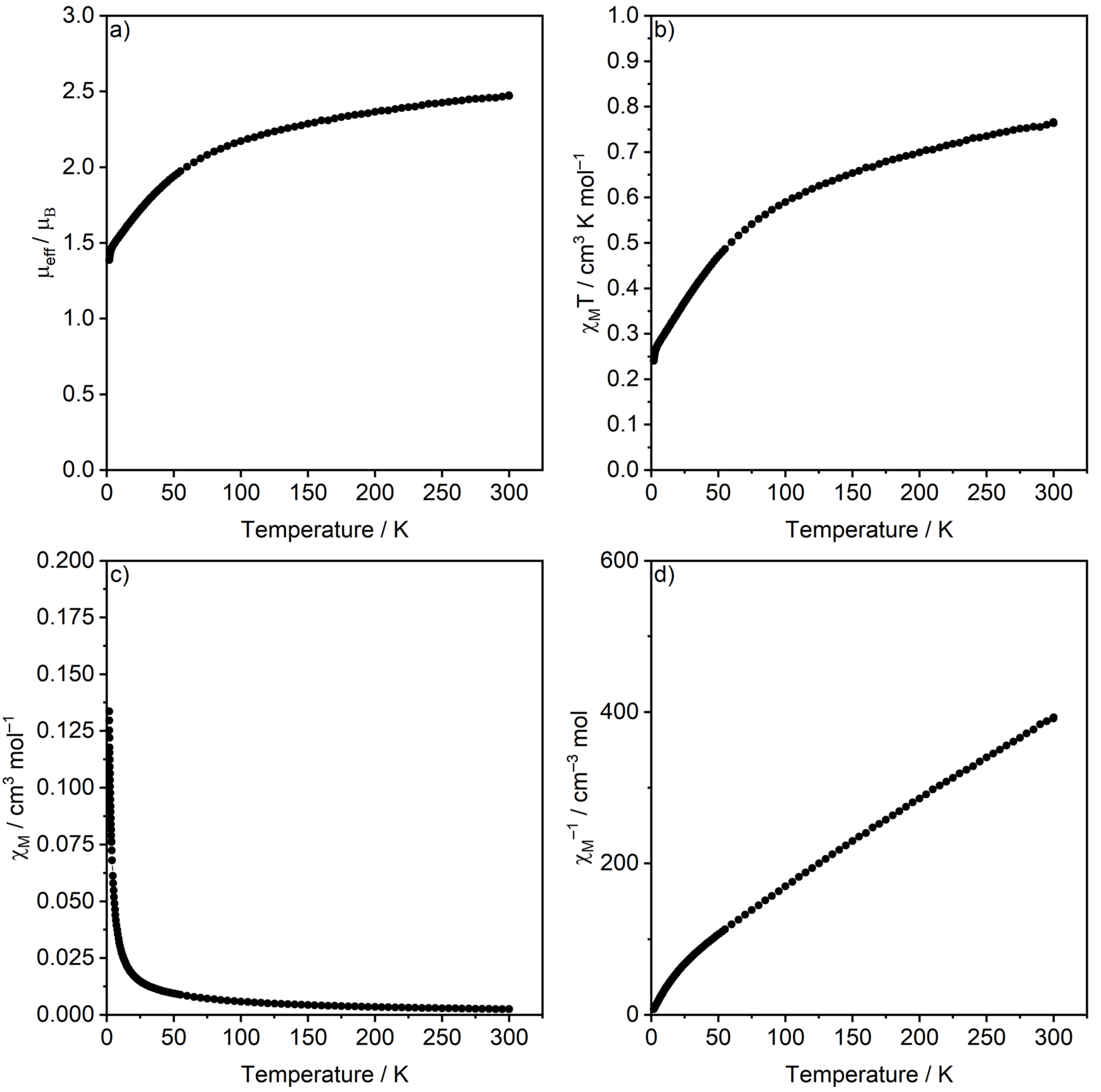

Figure S56. Variable-temperature SQUID magnetometry of **5** over the temperature range 1.8–300K: a) μ_{eff} vs T; b) χT vs T; c) χ vs T; d) χ^{-1} vs T.

S8. Density Functional Theory calculations

Density Functional Theory – General considerations

Unrestricted Kohn-Sham calculations were performed on **1·Et$_2$O** (S = 1), **2** (S = 2), **3**, **4a**, **5** (S = $^3/_2$), **4b** (S = 3) at the DFT level using the ORCA 5.0 quantum chemistry program suite.[43] H-atom geometry optimizations were performed using the PBE0 hybrid density functional,[44,45] with Grimme's D3BJ dispersion correction,[46,47] and the resolution of the identity 'chain of spheres' (RIJCOSX) approximation.[48,49] U was treated with a segmented all-electron relativistically contracted (SARC) DKH (Douglas–Kroll–Hess) basis set at the triple-ζ level (DKH-ZORA-TZVP),[50] while a split-valence polarized basis set (DKH-def2-SVP) was used for all other atoms.[51] A SARC/J auxiliary basis set was used for each atom type as appropriate.[50,52] No symmetry constraints were imposed. The SlowConv switch was often used to ensure SCF convergence, along with the DefGrid3 (tight integration grid) switch. The full structure of complex **1** was optimized using these same parameters and the structure was inspected using numerical frequency analysis, which revealed no imaginary vibrational modes which indicated the structure is a true local minimum.

Single point energies and properties were evaluated with the PBE0 hybrid functional, and a larger triple-ζ basis set (SARC-ZORA-TZVP)[50] and the ZORA (Zeroth Order Regular Approximation) scalar relativistic Hamiltonian.[53-55] All were used with their default settings within ORCA 5.0.4. We have chosen these approaches due to their success in describing similar complexes in the literature.[56] Electron density-based analysis of metal–ligand bonding utilized the AIMAll software package,[57] using WFX files generated from the DFT calculations (in ORCA 5.0.4). The default settings were used to complete the electron density topology and integration of atomic basins. The NBO (7.0) software package was used to compute NLMOs to analyse bonding orbitals.[58]

Table S19. Mean bond lengths (standard deviations in parentheses) from SC-XRD data for all ten independent molecular units in the asymmetric unit for complex **1**, and lengths from the geometry optimized structure.

Bond	Mean length (Å)	Standard deviation	Calculated length (Å)	Difference (XRD – Calculated) (Å)
U–S1	2.692	0.007	2.678	0.014
U–S2	2.657	0.008	2.653	0.004
U–Cl1	2.540	0.004	2.545	–0.005
U–Cl2	2.535	0.006	2.540	–0.005

Molecular orbital compositions and isosurfaces

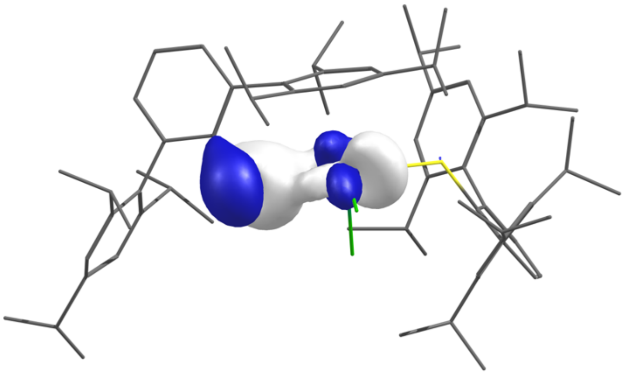

NLMO147: U1–S1

U (14.801%; 46.97% 5*f*, 49.21% 6*d*), S (78.508%)

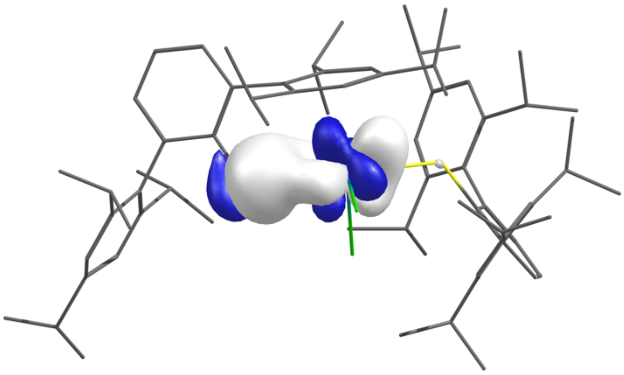

NLMO148: U1–S1

U (20.414%; 62.08% 5*f*, 28.67% 6*d*), S (76.400%)

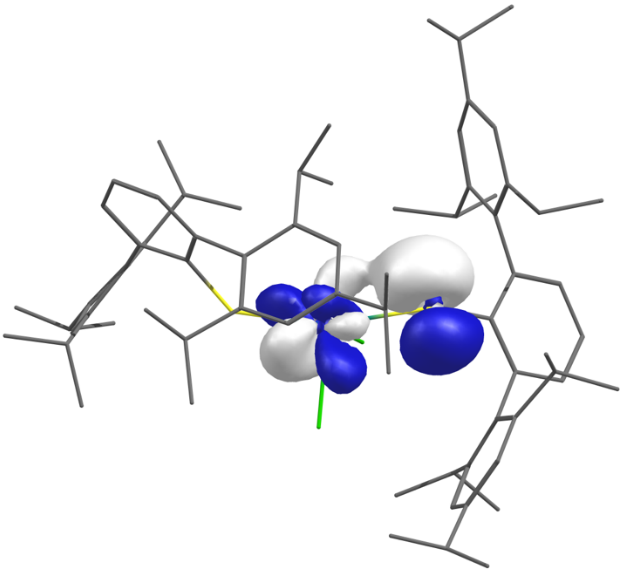

NLMO150: U1–S2 (glancing / S lone pair)

U (16.711%; 42.35% 5*f*, 57.13% 6*d*), S (80.387%)

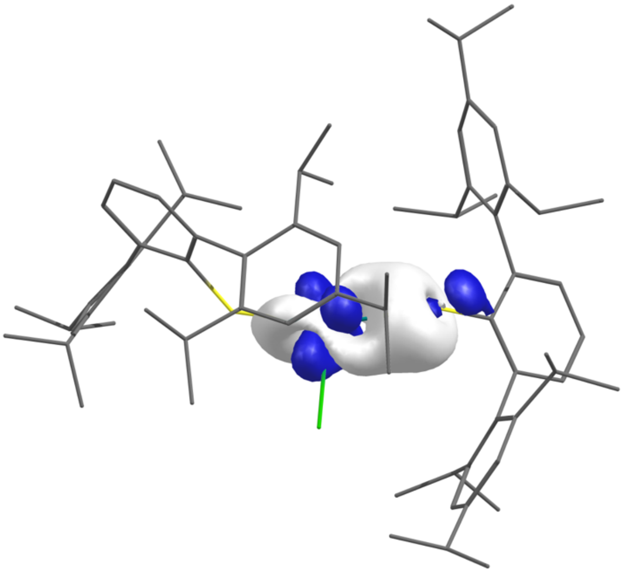

NLMO151: U1–S2

U (19.932%; 38.96% 5*f*, 44.78% 6*d*), S (77.049%)

Figure S57. Selected NLMO isosurfaces (0.05 a.u.) for complex **1**.

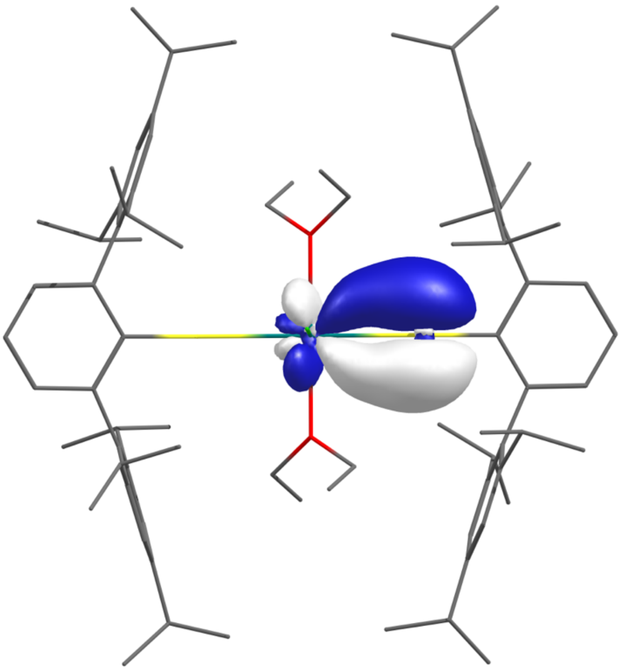

NLMO149: U1–S1

U (13.277%; 38.28% 5*f*, 61.48% 6*d*), S (84.055%)

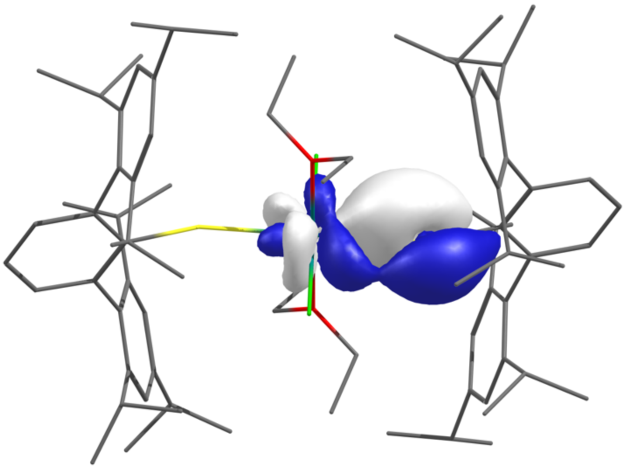

NLMO150: U1–S1

U (15.590%; 64.02% 5*f*, 31.31% 6*d*), S (79.322%)

Figure S58. Selected NLMO isosurfaces (0.05 a.u.) for complex **1·Et$_2$O**.

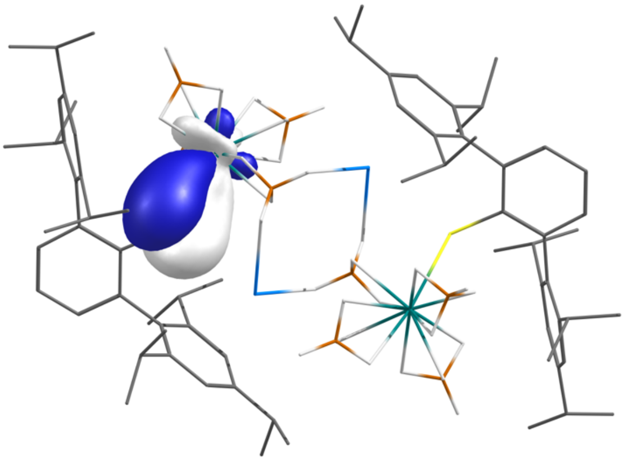

NLMO200: U1–S1

U (14.193%; 61.94% 5*f*, 37.51% 6*d*), S (82.336%)

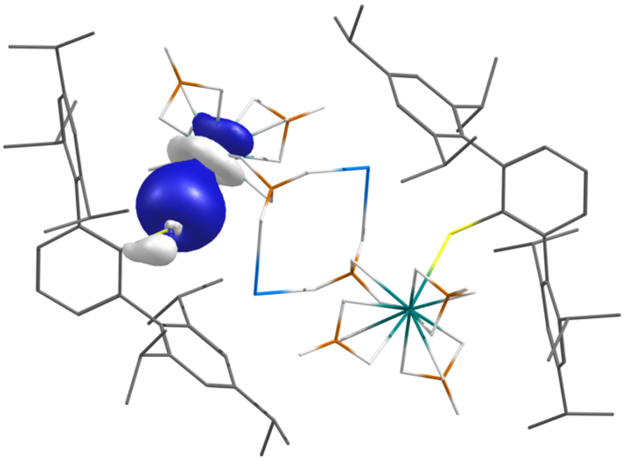

NLMO203 U1–S1

U (25.431%; 34.53% 5*f*, 57.02% 6*d*), S (72.248%)

Figure S59. Selected NLMO isosurfaces (0.05 a.u.) for complex **2**. Note that an equivalent set of NLMOs are present for the U2–S2 interaction.

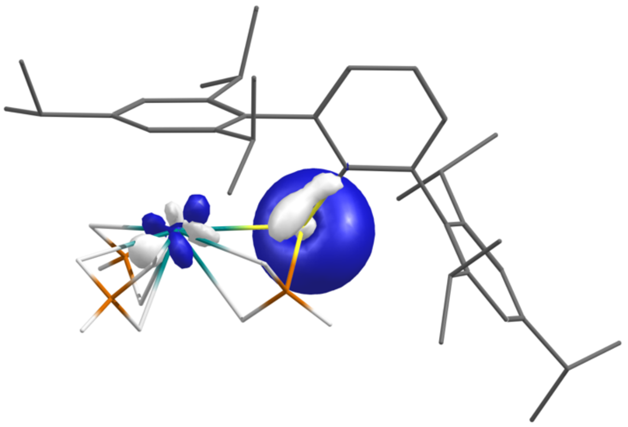

NLMO90: S1 lone pair

U (1.149%; 55.24% 5*f*, 39.79% 6*d*), S (96.319%)

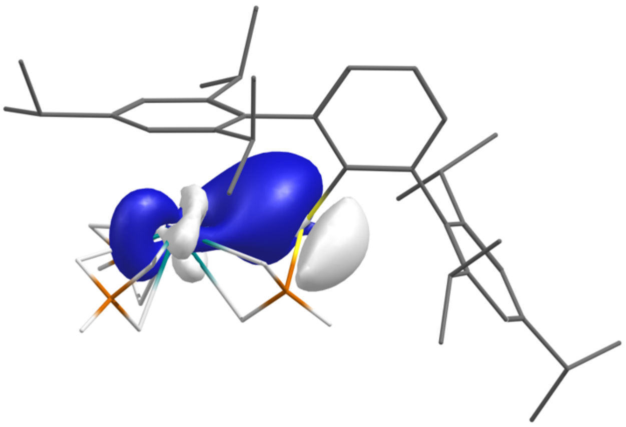

NLMO92: U1–S1

U (16.155%; 28.64% 5*f*, 63.81% 6*d*), S (79.781%)

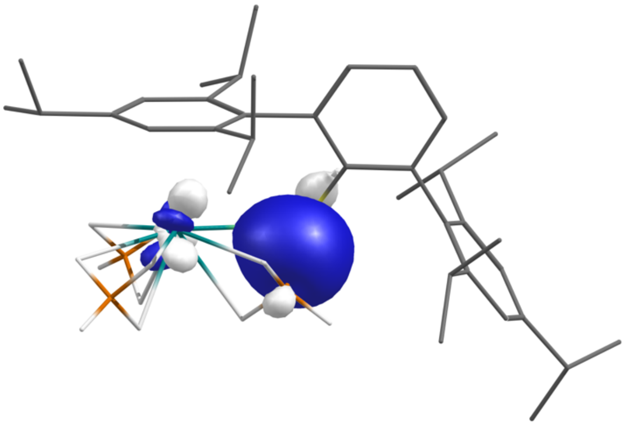

NLMO94: S1–B1

U (6.069%; 50.15% 5*f*, 45.88% 6*d*), S (63.342%), B (28.871%)

Figure S60. Selected NLMO isosurfaces (0.05 a.u.) for complex **3**.

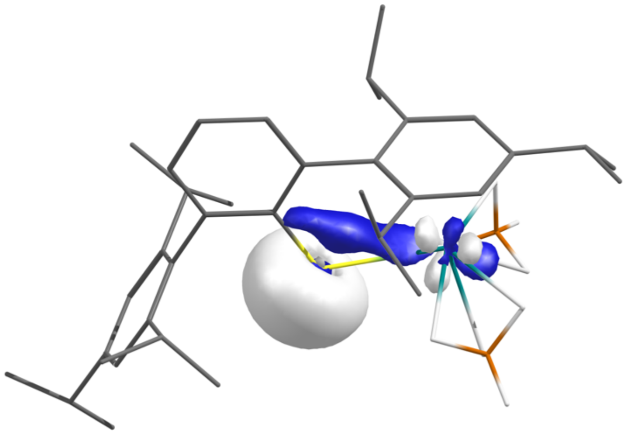

NLMO90: U1–S1 (glancing, S lone pair)

U (2.817%; 40.59% 5*f*, 56.15% 6*d*), S (95.675%)

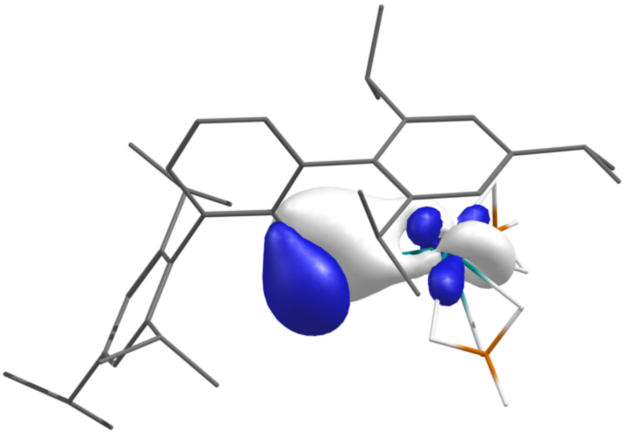

NLMO91: U1–S1

U (12.022%; 33.95% 5*f*, 60.43% 6*d*), S (80.903%)

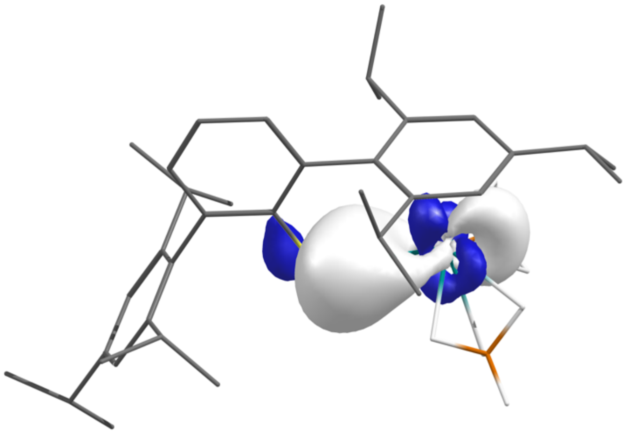

NLMO92: U1–S1

U (18.378%; 21.02% 5*f*, 65.86% 6*d*), S (78.885%)

Figure S61. Selected NLMO isosurfaces (0.05 a.u.) for complex **4a**.

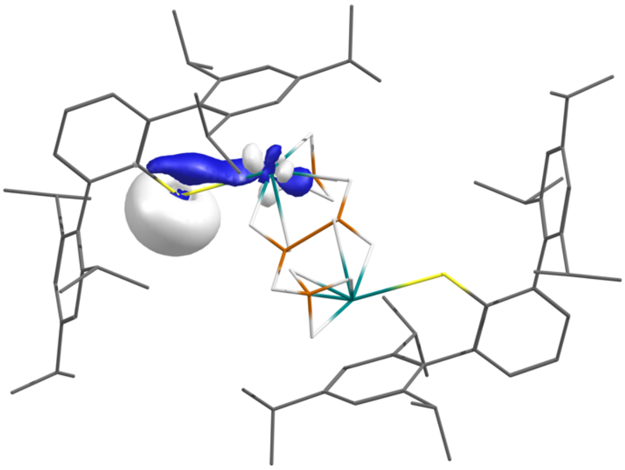

NLMO176: U1–S1 (glancing, S lone pair)

U (2.714%; 43.53% 5*f*, 54.62% 6*d*), S (95.723%)

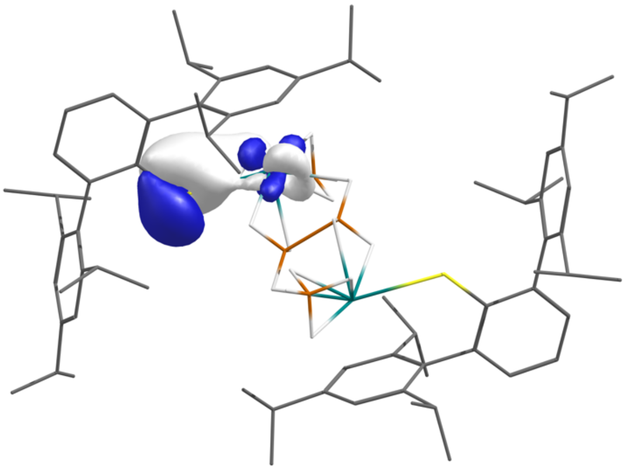

NLMO177: U1–S1

U (11.972%; 37.19% 5*f*, 55.61% 6*d*), S (81.629%)

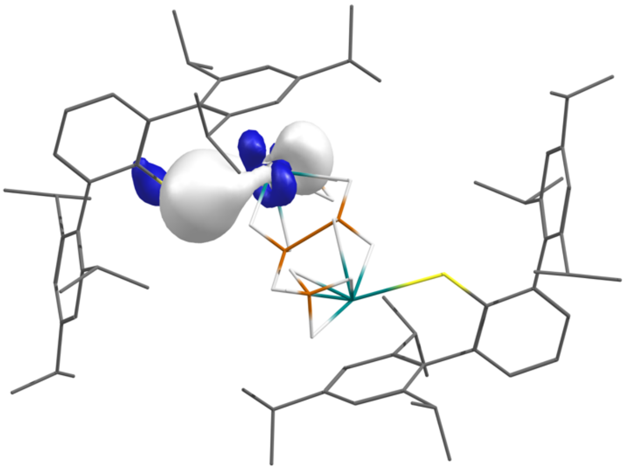

NLMO184: U1–S1

U (18.558%; 19.45% 5*f*, 66.83% 6*d*), S (78.114%)

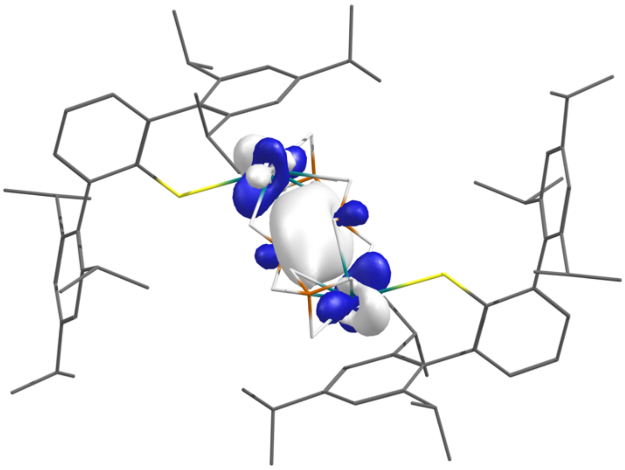

NLMO291: B95–B189 σ

–

Figure S62. Selected NLMO isosurfaces (0.05 a.u.) for complex **4b**. Note that an equivalent set of NLMOs are present for the U2–S2 interaction.

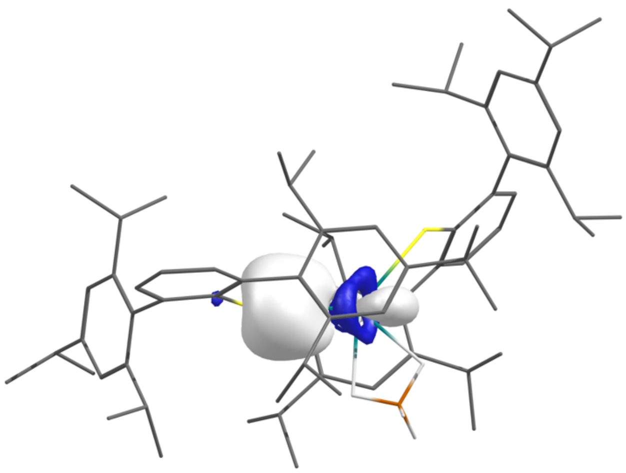

NLMO131: U1–S1

U (10.492%; 31.22% 5*f*, 44.92% 6*d*), S (87.758%)

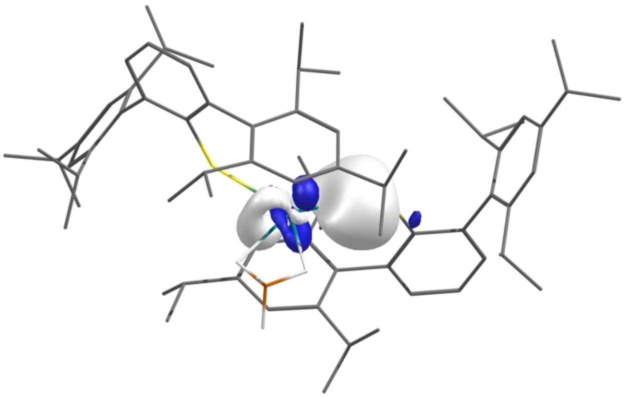

NLMO134: U1–S1

U (9.878%; 36.30% 5*f*, 43.79% 6*d*), S (88.048%)

Figure S63. Selected NLMO isosurfaces (0.05 a.u.) for complex **5**.

Table S20. Natural Localized Molecular Orbitals (NLMOs) computed σ- and π-contributions to the U–S bonds for complexes **1–5**.

Complex	Bond and approximate symmetry	U (%)	S (%)	U contribution (%)				S contribution (%)	
				s	*p*	*d*	*f*	*s*	*p*
**1**	U–S1 σ	20.414	76.400	9.19	0.05	28.67	62.08	19.09	80.54
	U–S1 σ	14.801	78.508	3.70	0.21	49.12	46.97	5.55	94.14
	U–S2 σ	19.932	77.049	16.12	0.15	44.78	38.96	25.64	74.06
	U–S2 σ	16.711	80.387	0.39	0.12	57.13	42.35	2.40	97.42
**1·Et_2O**	U–S1 π	13.277	84.055	0.08	0.16	61.48	38.28	0.00	99.93
	U–S1 σ	15.590	79.322	4.47	0.19	31.31	64.02	6.45	93.46
	U–S2 π	12.924	84.141	0.06	0.23	46.91	52.81	0.02	99.91
	U–S2 σ	14.924	79.780	6.75	0.14	47.13	45.97	6.26	93.65
**2**	U–S π	14.193	82.336	0.41	0.15	37.51	61.94	1.52	98.41
	U–S σ	25.431	72.248	8.36	0.09	57.02	34.53	28.67	71.12
**3**	U–S σ	16.155	79.781	7.41	0.14	63.81	28.64	12.28	87.39
**4a**	U–S σ	12.022	80.903	5.49	0.12	60.43	33.95	6.82	92.97
	U–S σ	18.378	78.885	13.08	0.04	65.86	21.02	27.66	72.01
**4b**	U1–S1 σ	11.972	81.629	7.07	0.14	55.61	37.19	10.05	89.76
	U1–S1 σ	18.558	78.114	13.65	0.07	66.83	19.45	26.78	72.89
**5**	U–S1 π	7.768	84.060	0.12	0.58	48.03	51.27	0.00	99.84
	U–S1 σ	10.492	87.758	23.80	0.07	44.92	31.22	45.61	54.13
	U–S2 π	8.003	83.791	0.05	0.59	44.91	54.44	0.00	99.84
	U–S2 σ	9.878	88.048	19.83	0.07	43.79	36.30	43.84	55.90

Coordinates

Complex 1

U	-0.000074117436	-0.001117896341	-0.001365495277
Cl	0.276229056765	0.739269926548	-2.420681172936
Cl	-0.472927971621	-2.445346965261	-0.506924138175
S	2.672969079064	-0.004064376311	-0.166616290715
S	-2.437258497505	1.020701054113	-0.233828467629
C	0.416920720919	1.349175600784	2.516339642293
C	-0.918110496644	1.004570977717	2.543219351307
H	-1.657118883085	1.804380161661	2.497331658299
C	-1.380016671216	-0.296994340166	2.648739171612
C	-0.433385090872	-1.285836427603	2.668944917013
H	-0.769228376810	-2.319216614405	2.717282180170
C	0.936902595382	-1.068660858729	2.616163110311
C	1.378769068156	0.297308429683	2.558009306519
C	0.890057354754	2.789125044168	2.378172946595
H	1.943814229509	2.821368228594	2.672450220933
C	0.751937138599	3.243259829045	0.942123320172
H	1.049036259811	4.291505524567	0.822232310175
H	1.401306525218	2.660422449823	0.268872970218
H	-0.289714382398	3.174514073016	0.593294875438
C	0.082179761121	3.713852193186	3.272999504123
H	0.002392408320	3.331845806727	4.298709597409
H	0.570903884289	4.691640476933	3.322857550954
H	-0.929240772143	3.875588661100	2.880825278211
C	-2.844123024274	-0.539536252584	2.778190403847
H	-3.329709045654	0.197468250243	2.121465482679
C	-3.254428708793	-1.924877662355	2.279458873326
H	-4.344371030418	-2.028368336032	2.307107817039
H	-2.934599551522	-2.083878785147	1.243379488272
H	-2.840414043724	-2.727434782810	2.903895150056
C	-3.325563463168	-0.255910341653	4.214393406522
H	-4.415660552697	-0.358878497353	4.267661441369
H	-2.879527562509	-0.960869446163	4.928266924822
H	-3.063638140175	0.761198646649	4.529966467051
C	1.948165950470	-2.204116848775	2.814691624542
H	2.811752988256	-1.974271674596	2.176711325318
C	1.405131178157	-3.617754035465	2.395264492642
H	2.229976859254	-4.337309308240	2.438744537360
H	0.624781522328	-3.964932482436	3.084062903880
H	1.003311149529	-3.606475793935	1.377306553875
C	2.425217611159	-2.255169956396	4.285815560685
H	3.156021863423	-3.063797903076	4.403547533603
H	2.902096218686	-1.321599218213	4.596067833947
H	1.580837381061	-2.458327745015	4.957001741060
C	3.500332195665	0.482230763068	1.312736768327
C	2.832562344311	0.602820646385	2.547163715106
C	3.520935581125	0.941060175697	3.680680038039
H	2.976403922252	1.042763998327	4.620286520086
C	4.854692134156	1.191917437856	3.634808172294
H	5.389414881857	1.480509234361	4.538751845109
C	5.556147121027	1.035894682499	2.438372229017
H	6.629647215142	1.209504732016	2.393935707169

C	4.875737759989	0.687038750630	1.268969418592
C	5.724836289399	0.573482371154	0.003761852365
C	5.935783919048	1.649735091133	-0.854982648927
C	6.740351912191	1.545510230066	-1.944689577706
H	6.880943974815	2.399244460946	-2.603459390969
C	7.473798827075	0.375095603897	-2.133739817554
C	7.208389522686	-0.743980245719	-1.390032725616
H	7.714720765519	-1.681775066523	-1.625138235108
C	6.362006313857	-0.679337094564	-0.255295719630
C	5.238991415030	2.965697892723	-0.574024706853
H	4.618339598150	2.823597325117	0.320431057139
C	4.308172002348	3.343066685600	-1.709255670328
H	3.578573482203	2.547324734903	-1.902721474328
H	3.753526258094	4.260777126485	-1.473428139257
H	4.859517065335	3.525633588615	-2.641497002836
C	6.220141975119	4.079691433733	-0.271409815245
H	5.690910689751	5.009424086239	-0.024897740529
H	6.869228087875	3.824268660373	0.575850540274
H	6.869446206577	4.287654444931	-1.132505802123
C	8.439013273062	0.200823736559	-3.392194288874
H	8.285218209800	-0.853392415768	-3.680467916460
C	7.971515027650	1.019975779429	-4.488488557100
H	6.909669709607	0.856650538864	-4.707343683234
H	8.104916547854	2.094990815359	-4.297617830414
H	8.530533043146	0.811648638303	-5.412734063592
C	9.807299409049	0.366279024857	-3.025464914196
H	10.103861641271	-0.300420717525	-2.205424311023
H	10.488295473712	0.163855407300	-3.865915798983
H	10.023082846486	1.392016969183	-2.688005546130
C	6.132484102023	-1.909953137500	0.633662821055
H	5.356549847616	-1.615820659669	1.354344278222
C	5.581429462614	-3.103285745405	-0.126692307424
H	4.664245646336	-2.839433646909	-0.666908529954
H	6.303978181651	-3.488429530610	-0.859085451827
H	5.347218335507	-3.921883040760	0.565993931577
C	7.347954732554	-2.314483720325	1.439946784006
H	7.717176398528	-1.487567461778	2.059601271182
H	7.106518744586	-3.154136781288	2.104041212963
H	8.174029456677	-2.631308785543	0.788340174713
C	-3.377745198620	-1.776176868317	-2.854549329623
C	-3.579475480698	-3.084871788454	-2.467042096083
H	-3.037094620559	-3.885947539712	-2.968298405216
C	-4.463880914981	-3.384625514443	-1.471384526015
C	-5.163963524453	-2.361963345538	-0.871318014434
H	-5.896954946226	-2.615221727219	-0.105666583558
C	-5.002923751759	-1.033439321397	-1.211159247465
C	-4.018217688168	-0.733217869087	-2.201586834661
C	-2.428616445797	-1.501014105123	-4.061663347850
H	-2.125352215063	-0.447931981518	-3.982925670874
C	-3.166111662074	-1.684921897528	-5.394197062633
H	-4.059297312993	-1.055281623252	-5.467486736194
H	-2.505242532478	-1.434292223116	-6.233778082853
H	-3.486473298491	-2.728785906640	-5.515599657434
C	-1.162030534762	-2.335067571809	-4.075151338667
H	-0.603607124327	-2.242544307684	-3.139671359723

H	-1.371831729145	-3.400090933245	-4.243122565183
H	-0.509181403737	-2.001712207392	-4.891279870847
C	-4.628435162119	-4.785604582976	-0.997283742674
H	-4.368048141457	-5.401758126842	-1.877820817472
C	-6.026832494332	-5.206999774900	-0.637876824096
H	-6.743315821989	-4.954624521511	-1.429147972361
H	-6.067435866079	-6.291254056792	-0.476048021749
H	-6.379099425452	-4.734158061714	0.290310848087
C	-3.655521324277	-5.146273252093	0.039506365117
H	-3.692913683862	-6.218699949501	0.274897785900
H	-2.628351785926	-4.902411093825	-0.259684714255
H	-3.846031776230	-4.602337616125	0.977371767584
C	-5.892608216197	0.071691109520	-0.630557393938
H	-5.335258795136	1.011214234845	-0.682666159060
C	-7.198297495950	0.247570979382	-1.468080392250
H	-7.801203350754	1.067963367453	-1.059466172982
H	-6.970573144648	0.475781919602	-2.515399514547
H	-7.793095597258	-0.674101691215	-1.438967225034
C	-6.300161081193	-0.159010208553	0.832445649775
H	-6.888268541258	0.693151199984	1.194892779655
H	-6.921528698203	-1.056151909609	0.945394437362
H	-5.422433251921	-0.263911955364	1.478569914937
C	-3.131170902636	1.633676434850	-1.780537632793
C	-3.840860821235	0.715433479208	-2.563068081216
C	-4.484379455711	1.189351297111	-3.725916113596
H	-5.047608397608	0.502725161168	-4.352047637469
C	-4.428549401155	2.536734281472	-4.007754604536
H	-4.936254785198	2.897198519751	-4.904532962756
C	-3.773629642215	3.451403512343	-3.217927367803
H	-3.785530837326	4.513487151841	-3.447686096339
C	-3.148286318740	2.992414480782	-2.044203571393
C	-2.576257132436	4.068985241683	-1.151693427610
C	-3.271693794355	4.374644383696	0.064141644949
C	-2.843959891585	5.423774796222	0.868746976612
H	-3.396363342958	5.651628123566	1.779238001847
C	-1.732647028386	6.143112595257	0.540998132544
C	-1.132132519009	5.862124164254	-0.655887578476
H	-0.272735887817	6.476311482700	-0.940501188409
C	-1.467367257416	4.816254524370	-1.477223294900
C	-4.575583294596	3.597344168468	0.468222724364
H	-4.560049146790	2.624392739752	-0.025028488645
C	-5.782626541079	4.395592940702	-0.015040739729
H	-5.753290335560	4.529705732291	-1.103142516373
H	-6.722297217756	3.884510282217	0.236167076072
H	-5.802193294930	5.390757572468	0.447815904351
C	-4.636450240488	3.362876763513	2.001163310940
H	-3.719948541319	2.875786510696	2.354046998827
H	-4.769355222617	4.299775027351	2.554568442897
H	-5.484129374648	2.711293352541	2.247262902867
C	-1.357370179838	7.318624843721	1.368137689638
H	-0.378958108942	7.646510318108	0.969911334295
C	-2.277227911820	8.479426649618	1.164920003030
H	-2.394488159121	8.730106530608	0.103419386591
H	-3.281747868755	8.265530793376	1.558591564349
H	-1.904354654388	9.371541031632	1.684431068239

C	-1.175374144801	7.070022593523	2.762800160830
H	-0.879016781804	7.982576356274	3.297296066342
H	-2.093061340686	6.714711780599	3.256582175742
H	-0.405266232429	6.321037003451	2.964647085464
C	-0.719770954715	4.540990806290	-2.772205740556
H	-1.097793441176	3.602300100358	-3.191226453145
C	-0.968242538449	5.682902835754	-3.785811662293
H	-0.466930986799	5.468568239481	-4.737911244057
H	-2.036456791759	5.823116293201	-3.986774175481
H	-0.574291238529	6.632397005905	-3.401002798693
C	0.804089960141	4.376046651259	-2.557998578870
H	1.299135307026	4.166212433441	-3.513538420412
H	1.251241997554	5.286724121597	-2.139043313281
H	1.019036761541	3.539093811018	-1.888083410574

Complex 1·Et_2O

U	-0.000696754953	-0.000155095266	0.000434579856
S	0.000509998010	0.172800005721	2.648989998275
S	-0.000369998887	-0.172850002898	-2.649080000739
Cl	0.000059998155	-2.574159997241	-0.000019997661
Cl	0.000059998067	2.574199998084	-0.000019997555
O	-2.370229997238	-0.067429989603	-0.008720004842
O	2.370360000191	0.067379992893	0.008620006321
C	-0.028920000451	-0.364060008916	4.349019983095
C	-1.256330000013	-0.414539993921	5.052070002793
C	-1.251469995870	-0.773429997007	6.397969997512
C	-0.082289997737	-1.058130003374	7.083299996340
C	1.121369989451	-0.964989978158	6.394309995937
C	1.169030041155	-0.624940030041	5.046450097287
C	-2.592810006405	-0.064630004071	4.473890014959
C	-2.975799991459	1.278670004691	4.333089979045
C	-4.292870002001	1.569429996789	3.964299983620
C	-5.228500007021	0.577820005794	3.720160064703
C	-4.823459999238	-0.746180008195	3.853509975168
C	-3.527249995559	-1.087319995170	4.241729986539
C	-2.010500005394	2.429859997581	4.608830006242
C	-1.922259997273	3.400690002966	3.429410000436
C	-2.394099999574	3.189050001098	5.877960002681
C	-6.663029986676	0.913530005784	3.340699931503
C	-7.011299997586	0.422190003880	1.943680021708
C	-7.648040008949	0.354129995556	4.360320016575
C	-3.149700002420	-2.555370001326	4.347680006760
C	-2.774739997471	-3.098830002178	2.963219996835
C	-4.230499998795	-3.406490000731	5.002080000231
C	2.541739910187	-0.468109963421	4.459709802545
C	3.127320046948	0.807509983674	4.465170070103
C	4.460390048464	0.941259979631	4.057740180060
C	5.206119917804	-0.159659969856	3.656779727242
C	4.610420061952	-1.403700019851	3.681880201011
C	3.279870005086	-1.612090002238	4.093970018476
C	2.376279963331	2.015280005799	4.983559940459
C	2.468929993722	3.260599992218	4.088360004860
C	2.831410014932	2.340450006190	6.421920006245

C	2.665309995915	-2.987719994732	4.171609952215
C	1.885009991258	-3.317759996856	2.905020011502
C	3.669740001809	-4.098370005755	4.488129996334
C	-3.209109999766	0.646829996626	0.912190001703
C	-3.723189997020	1.946500001424	0.303349998227
C	-3.111730001137	-0.817360002226	-1.042229998473
C	-3.711640000448	-2.101889997518	-0.500570000088
C	0.029049998183	0.364090008416	-4.349059983489
C	1.256460002596	0.414579994121	-5.052120004614
C	1.251599998884	0.773369998945	-6.398079997576
C	0.082420000705	1.058160007774	-7.083339996833
C	-1.121269990832	0.964889979569	-6.394319992021
C	-1.168990040616	0.624900029302	-5.046590096689
C	2.592940005402	0.064660003197	-4.473940018058
C	2.975919992171	-1.278640000119	-4.333139979696
C	4.293000000046	-1.569389996378	-3.964349986421
C	5.228620008066	-0.577790006732	-3.720200064316
C	4.823549997260	0.746170006807	-3.853479976802
C	3.527279992599	1.087359995412	-4.241799985543
C	2.010630008714	-2.429829998725	-4.608880003258
C	1.922389994921	-3.400750001089	-3.429509998979
C	2.394229997124	-3.189020001960	-5.878000003141
C	6.663159989762	-0.913490005175	-3.340739927123
C	7.011339998109	-0.422230004391	-1.943800023078
C	7.648170011854	-0.354089995124	-4.360360016996
C	3.149830007189	2.555400000592	-4.347730003055
C	2.774869999738	3.098860001213	-2.963269998915
C	4.230589995451	3.406469997750	-5.002050001298
C	-2.541699910287	0.468159965531	-4.459789798867
C	-3.127200049776	-0.807479984645	-4.465210068536
C	-4.460260051564	-0.941229980383	-4.057780182856
C	-5.206079916558	0.159619974458	-3.656919724185
C	-4.610380062186	1.403660024826	-3.682010200106
C	-3.279750007977	1.612120006397	-4.094010018819
C	-2.376139964774	-2.015330008015	-4.983659939486
C	-2.468799995917	-3.260559991623	-4.088410001440
C	-2.831280017082	-2.340420007078	-6.421970008146
C	-2.665179998914	2.987759995299	-4.171659948286
C	-1.884869991705	3.317709994903	-2.905110014082
C	-3.669610003996	4.098400004771	-4.488169996927
C	3.209200002247	-0.646839992547	-0.912160002751
C	3.723319999971	-1.946470002460	-0.303400000165
C	3.111850002611	0.817310005548	1.042129999844
C	3.711729997651	2.101869999921	0.500610000568
H	-2.209273926833	-0.806666634943	6.917306850216
H	-0.103483368310	-1.326069194237	8.137827410491
H	2.063639893911	-1.152721953451	6.908923042131
H	-4.598024204246	2.613301807745	3.876587134460
H	-5.541744937509	-1.543326287420	3.664986235922
H	-1.014160310959	1.998169278774	4.758791050855
H	-2.887354509390	3.882176261799	3.226034275355
H	-1.583689401020	2.895214381158	2.518161135834
H	-1.197729080309	4.195287843435	3.648093829611
H	-1.666812320838	3.984375713395	6.085021071236
H	-2.427618476892	2.523014308561	6.749246452674

H	-3.382745428337	3.655808177796	5.776053573804
H	-6.749139961181	2.010434145504	3.347673593451
H	-6.926376879698	-0.670719975646	1.875607640991
H	-6.348485851613	0.855071702996	1.186083246181
H	-8.041930289166	0.689770987970	1.677450407555
H	-8.675533621705	0.654129743649	4.117442712770
H	-7.419118571283	0.707519022061	5.372778407405
H	-7.618612454227	-0.742899749470	4.377676433934
H	-2.252451027619	-2.621233280447	4.974379391965
H	-2.414891151317	-4.133433599619	3.031167826233
H	-1.985454767285	-2.495679560204	2.499017158952
H	-3.646485719348	-3.086114395519	2.297118673584
H	-5.128185310147	-3.487986997586	4.375876070427
H	-4.534578732974	-2.995933759439	5.972850735150
H	-3.861249276411	-4.426677221680	5.165298955167
H	4.930191044877	1.925300096470	4.073731981706
H	5.200844003310	-2.273916861952	3.393833002239
H	1.314848318169	1.744929749110	5.033785621416
H	3.499335297023	3.626580634862	3.991885036802
H	1.870607815724	4.072696029007	4.518921494916
H	2.075827930235	3.055150252035	3.085384960451
H	2.692622741197	1.476096745119	7.081693965961
H	2.253477720108	3.179288981951	6.829948225104
H	3.894518524468	2.614226463902	6.441646002615
H	1.942999055070	-2.961612515066	4.998147415612
H	1.144818939588	-2.545656377774	2.670760581567
H	1.356460258173	-4.273972467098	3.006669806124
H	2.559454340183	-3.395491534883	2.043819041539
H	4.360336848177	-4.272863619030	3.652501958991
H	3.138333366536	-5.040593448385	4.667890152590
H	4.266838591547	-3.866741861939	5.378935295307
H	-2.591613115966	0.833920313045	1.794883406328
H	-4.020842035872	-0.017650277905	1.226398334611
H	-2.882549021377	2.602860708325	0.056190486978
H	-4.356153578994	2.456471851745	1.038264710488
H	-4.314312419538	1.772781023730	-0.602514185657
H	-2.377783681225	-1.009990898882	-1.827980708526
H	-3.859325316183	-0.136337525383	-1.457515982161
H	-2.926449533598	-2.759995247761	-0.115025383205
H	-4.222998846804	-2.613526423788	-1.324258140984
H	-4.446963094966	-1.918247851238	0.291157170894
H	2.209403462474	0.806597675502	-6.917418323614
H	0.103559589634	1.326114377046	-8.137864076628
H	-2.063513285133	1.152630154951	-6.908988302479
H	4.598154752184	-2.613265004657	-3.876662015994
H	5.541813191391	1.543329667763	-3.664921744050
H	1.014286165657	-1.998145206160	-4.758825159346
H	2.887490519664	-3.882244020030	-3.226174801313
H	1.583820315901	-2.895346736690	-2.518218459946
H	1.197863960446	-4.195332369984	-3.648263484919
H	1.666936593129	-3.984339562356	-6.085066052102
H	2.427766618159	-2.522992890959	-6.749293588184
H	3.382870133650	-3.655789183110	-5.776083684206
H	6.749261166259	-2.010397147970	-3.347764760792
H	6.926410669708	0.670678331892	-1.875670652707

H	6.348501554260	-0.855129747633	-1.186233731303
H	8.041960777470	-0.689796791680	-1.677503997843
H	8.675663788627	-0.654088757239	-4.117477479522
H	7.419250638358	-0.707480848077	-5.372817460707
H	7.618740049267	0.742940392059	-4.377716860701
H	2.252573416554	2.621303308896	-4.974422133702
H	2.415042019087	4.133472098806	-3.031218931183
H	1.985575307910	2.495714094968	-2.499072809568
H	3.646611712659	3.086122823136	-2.297161882781
H	5.128280696159	3.487936203998	-4.375846640817
H	4.534685020113	2.995960261730	-5.972837235144
H	3.861375496934	4.426676566404	-5.165240224001
H	-4.930022355005	-1.925293472894	-4.073775703119
H	-5.200833244942	2.273877705116	-3.394013182170
H	-1.314713243011	-1.744953492449	-5.033868635567
H	-3.499214349476	-3.626501420932	-3.991858287287
H	-1.870537548057	-4.072715834386	-4.518945635943
H	-2.075631802170	-3.055076194564	-3.085467494452
H	-2.692459401429	-1.476054902049	-7.081721093525
H	-2.253389096590	-3.179269299717	-6.830040462839
H	-3.894400660393	-2.614155457992	-6.441716963787
H	-1.942892035819	2.961627567686	-4.998216636848
H	-1.144730800159	2.545542303839	-2.670877267809
H	-1.356236437429	4.273881642668	-3.006732532709
H	-2.559294742185	3.395486864114	-2.043895118489
H	-4.360222176368	4.272872656666	-3.652548728588
H	-3.138212062037	5.040632905200	-4.667909507002
H	-4.266692104577	3.866776749307	-5.378986832675
H	2.591732847920	-0.833992595069	-1.794862944528
H	4.020938754483	0.017614591154	-1.226432669012
H	2.882695421789	-2.602846767331	-0.056219432177
H	4.356258994329	-2.456425730116	-1.038347399695
H	4.314479419244	-1.772796345800	0.602452838594
H	2.377938862632	1.009866393597	1.827925563311
H	3.859487115048	0.136313824733	1.457389284019
H	2.926555565345	2.760032278848	0.115128667260
H	4.223107993270	2.613427665099	1.324337526542
H	4.447039182457	1.918308917104	-0.291148675510
C	6.654234979319	0.007135567360	3.234234224663
C	6.855454550793	-0.323448879482	1.755283090234
C	7.600188707517	-0.805774639638	4.118208892047
C	-6.654208092500	-0.007207292395	-3.234440937262
C	-6.855457508325	0.323231921614	-1.755460337877
C	-7.600141810496	0.805791768103	-4.118352269487
H	6.902262581910	1.070189761692	3.372008728104
H	6.611601935133	-1.373610413968	1.546734873616
H	7.897673583965	-0.160009800185	1.452695034159
H	6.220553589938	0.301882782618	1.116690339971
H	7.419713404064	-1.882954683335	4.008928261580
H	7.467555068557	-0.551948828378	5.176447069963
H	8.646606955565	-0.615767545896	3.847478233798
H	-6.902232300208	-1.070248076476	-3.372327059954
H	-6.611595539873	1.373371519879	-1.546809450081
H	-7.897684415382	0.159775184562	-1.452906173916
H	-6.220575239985	-0.302171696798	-1.116917853515

H	-7.419675566229	1.882961305388	-4.008956008474
H	-7.467482691599	0.552075354385	-5.176612957029
H	-8.646566001265	0.615752602309	-3.847665826308

Complex 2

U	-0.000634535450	0.000067980849	-0.001974106892
U	1.273903175983	7.462586724278	-0.119716668362
H	1.623614174163	-0.957572560769	-1.305817333865
H	-0.350441431809	8.420201281459	1.184016398081
H	-1.976574550398	-1.070938716198	-0.462941669803
H	3.249764475893	8.534002711375	0.341014436412
H	0.560121944962	1.000266467713	-2.074365136950
H	0.713410411001	6.462218723129	1.951079979902
H	2.162130886735	0.862042400444	-0.071568037661
H	-0.888819987323	6.600754092340	-0.050182704112
K	0.638553704104	3.732384812771	-2.588174462824
K	0.634715021789	3.730359942436	2.466373722478
S	0.516510841220	0.623387019881	2.568929302026
S	0.756757793649	6.839267689312	-2.690620077126
C	-0.261066409252	-0.137925096818	3.975134295357
C	1.534335037052	7.600719788723	-4.096785012921
C	-0.051538781674	-1.497299154530	4.277588954239
C	1.324747455101	8.960043882338	-4.399309736550
C	-0.585262995764	-2.008232918118	5.468129367662
C	1.858541736048	9.470987664922	-5.589930092371
H	-0.381286129893	-3.046600161180	5.719454150428
H	1.654553032570	10.509356662526	-5.841243509385
C	-1.324382819638	-1.218822056959	6.328224601695
C	2.597651551773	8.681576803268	-6.450015333309
H	-1.732575605360	-1.640934536176	7.245108722890
H	3.005876447260	9.103658586683	-7.366899121128
C	-1.504363938463	0.126731404352	6.040696847512
C	2.777582610585	7.336003321632	-6.162417629880
H	-2.038807646777	0.771798224979	6.737017583563
H	3.312023819484	6.690917309150	-6.858724106428
C	-0.964057528440	0.691950265781	4.884793193494
C	2.237276197272	6.770784464224	-5.006513975168
C	0.798075036433	-2.419720601716	3.450704363152
C	0.475203696460	9.882475342826	-3.572505090322
C	2.197559144386	-2.303707386933	3.496093703347
C	-0.924340482057	9.766452100689	-3.617804484883
C	2.976001640205	-3.244669087258	2.772447674781
C	-1.702673036338	10.707383752559	-2.894088376576
H	4.061885463467	-3.159850937239	2.811268596200
H	-2.788562160341	10.622585081436	-2.932870248951
C	2.391963272795	-4.271720543175	2.005322395382
C	-1.118684546378	11.734475272800	-2.127113110091
C	1.013450584190	-4.357474613534	2.003811121017
C	0.259828048160	11.820269288961	-2.125471817529
H	0.542949221136	-5.149904160500	1.423748954465
H	0.730342092536	12.612698663759	-1.545428552358
C	0.202932226130	-3.475056164176	2.723099335646
C	1.070396469816	10.937730878600	-2.844870048901

C	2.877067146392	-1.262589150328	4.358817155861
C	-1.603728452371	8.725403861733	-4.480557826766
H	2.124933063741	-0.523915265218	4.656264260532
H	-0.851572241691	7.986738807345	-4.777964157312
C	4.003671587076	-0.512246589809	3.638943093037
C	-2.730342896674	7.975061305908	-3.760683772075
H	4.830713254590	-1.181195566536	3.370869761081
H	-3.557387584621	8.644029955161	-3.492669258783
H	4.413063955394	0.270795207309	4.288869698856
H	-3.139711545659	7.191992869559	-4.410590137663
H	3.635005229751	-0.038123589956	2.721695186282
H	-2.361696718734	7.500987278231	-2.843403237865
C	3.404835847339	-1.934086315642	5.618189432432
C	-2.131567215974	9.396741027883	-5.739880217918
H	2.599799194178	-2.434537599584	6.170257185239
H	-1.326608988386	9.897255272322	-6.292016403341
H	3.871772956455	-1.202077997975	6.290743498989
H	-2.598446953358	8.664662225724	-6.412393887442
H	4.158831658871	-2.692420745878	5.371026441541
H	-2.885625662580	10.155028584514	-5.492755164337
C	3.202665055465	-5.316093856365	1.248149423648
C	-1.929446389392	12.778838560319	-1.369860182057
H	2.487809313014	-5.861766298651	0.614948948878
H	-1.214536696216	13.324485583226	-0.736690912638
C	4.251835199568	-4.723690937819	0.382833220032
C	-2.978506506969	12.186505636822	-0.504573871101
H	3.834535525865	-4.002702052799	-0.329620553788
H	-2.561210948383	11.465506594524	0.207873606041
H	4.776047930731	-5.498542304776	-0.191945635480
H	-3.502674688869	12.961368606544	0.070234698371
H	5.013648793361	-4.195434988852	0.972021080416
H	-3.740380829009	11.658271765790	-1.093706054126
C	3.809336839884	-6.307456199640	2.240043374102
C	-2.536058212492	13.770250881622	-2.361694059824
H	4.516998768625	-5.799717139237	2.908499681771
H	-3.243742198185	13.262595555993	-3.030191141087
H	4.354275802675	-7.104737383615	1.717665770662
H	-3.080956071003	14.567544436667	-1.839288888261
H	3.038242351852	-6.772670071016	2.865302064123
H	-1.764938985720	14.235466783060	-2.986924924356
C	-1.289834003240	-3.712976414192	2.681680317742
C	2.563162698096	11.175781106599	-2.803420973758
H	-1.790289906353	-2.775212295251	2.958337982618
H	3.063694002792	10.238043478221	-3.080041480576
C	-1.807508780500	-4.122566508375	1.286502568758
C	3.080777409345	11.585361165480	-1.408163269133
H	-1.528144063014	-5.153884266414	1.039446166186
H	2.801413632785	12.616682453266	-1.161115265159
H	-1.420108066889	-3.469668569712	0.498672784742
H	2.693332160379	10.932445606455	-0.620375946017
H	-2.902103881732	-4.066241201738	1.266308506054
H	4.175372916342	11.529035113847	-1.387930684698
C	-1.714299247326	-4.786485005687	3.691838274058
C	2.987577993632	12.249239735928	-3.813638979227
H	-2.798793054848	-4.948154704844	3.648229347724

H	4.072060192525	12.410998182731	-3.770003654962
H	-1.457080235587	-4.516034228987	4.720097686543
H	2.730427430463	11.978684963323	-4.841885702000
H	-1.220402893285	-5.740472851752	3.465251557699
H	2.493614427325	13.203220037504	-3.587157924699
C	-1.073578036565	2.180579008346	4.731326324572
C	2.346906724497	5.282235706965	-4.853056989437
C	-0.135268870511	3.011203352981	5.378041923879
C	1.408477527850	4.451531375937	-5.499752712765
C	-0.289665881629	4.401856821609	5.305111753439
C	1.562944600328	3.060897926956	-5.426902482076
H	0.434266892056	5.041244973661	5.805877483694
H	0.839013915064	2.421515073948	-5.927672839383
C	-1.362716876655	4.985257851980	4.622970286296
C	2.636045567261	2.477416879135	-4.744741009970
C	-2.256516848842	4.147846684961	3.975925769090
C	3.529845539545	3.314828046610	-4.097696491425
H	-3.082746073996	4.593944664386	3.423583592625
H	4.356105582323	2.868790190807	-3.545359111669
C	-2.152562028300	2.756280573112	4.025197258041
C	3.425770692433	4.706454156852	-4.146918045702
C	1.057139443823	2.444664467168	6.133080819613
C	0.216189250804	5.018010268474	-6.254851540999
H	0.870454926277	1.374295867993	6.283422885589
H	0.402878772826	6.088378401795	-6.405220080827
C	2.336085831570	2.576459440015	5.322724621130
C	-1.062817118651	4.886285323908	-5.444515365647
H	2.263249343673	2.041425347030	4.366775121726
H	-0.989967806642	5.421361612263	-4.488590046932
H	3.188432642772	2.149005824430	5.865468829247
H	-1.915124585096	5.313760120989	-5.987303312809
H	2.575882172145	3.631247125046	5.122881960460
H	-1.302642819987	3.831513898444	-5.244639738375
C	1.219631680376	3.084916097037	7.513161227860
C	0.053697030541	4.377758639299	-7.634941948307
H	1.501727202794	4.142741844617	7.443596306747
H	-0.228468356388	3.319953060175	-7.565399080985
H	2.010324590155	2.574453335870	8.076277171504
H	-0.736929225112	4.888295753834	-8.198082425461
H	0.292752256400	3.021391502195	8.095177175771
H	0.980609211801	4.441234632447	-8.216914359217
C	-1.604298708534	6.490968195536	4.566900002016
C	2.877627309077	0.971746480463	-4.688540710548
H	-1.818996908694	6.727144319194	3.511832345172
H	3.092289326558	0.735575635275	-3.633472370434
C	-0.433004906495	7.319223184577	4.996431510159
C	1.706273632564	0.143531559819	-5.118222251970
H	-0.206442444541	7.183687310598	6.062842715946
H	1.479850570228	0.279160090725	-6.184648107610
H	-0.643290534433	8.385100598836	4.847758510875
H	1.916502651859	-0.922360015432	-4.969585850992
H	0.472403239876	7.085205176296	4.425757704013
H	0.800832279873	0.377582689738	-4.547618808546
C	-2.865362321170	6.830709943575	5.378608683093
C	4.138580985822	0.632024781928	-5.500329463253

H	-3.737123762467	6.266838164039	5.029400119486
H	5.010390856427	1.195863985161	-5.151175959663
H	-3.098720699710	7.899800575029	5.301328287707
H	4.371955794689	-0.437069060034	-5.423143637576
H	-2.713174063663	6.590999190305	6.439009891475
H	3.986321620283	0.871808198917	-6.560704227520
C	-3.230002032625	1.892879690440	3.399102869609
C	4.503270754025	5.569875056951	-3.520893602550
H	-2.786862256200	0.912472076765	3.179908116978
H	4.060140584244	6.550285824454	-3.301688955377
C	-3.783001338146	2.469968835631	2.094713600999
C	5.056219990015	4.992765879859	-2.216424379733
H	-4.389284034219	3.367477692505	2.269376016546
H	5.662516250266	4.095262823956	-2.391061476679
H	-4.426421440797	1.731859863057	1.602726016903
H	5.699620598586	5.730882691933	-1.724417890670
H	-2.984204973665	2.736606824492	1.391968116520
H	4.257382412668	4.726143915104	-1.513725325771
C	-4.371207205359	1.694804857547	4.404947194414
C	5.644535900664	5.768009860542	-4.526687857040
H	-4.019997755144	1.214990530191	5.324907670687
H	5.293360124184	6.247858817179	-5.446644453690
H	-5.157691103468	1.062799781347	3.974154102100
H	6.430965904542	6.400031744964	-4.095818468198
H	-4.817844421770	2.660658339123	4.674068256186
H	6.091233366134	4.802190807509	-4.795828927104
B	1.296449524113	-2.008790876335	-0.710534065631
B	-0.023180881728	9.471445579971	0.588843297997
H	1.414240256921	-1.753531258232	0.508277373105
H	-0.140840757633	9.216303376907	-0.630002994861
H	0.053664331870	-2.089241904914	-0.896542748853
H	1.219589087829	9.551850793781	0.774985434928
H	1.891855872683	-2.978848742482	-1.087683570651
H	-0.618584134238	10.441508071893	0.965995690824
B	-2.312651231254	-0.691424167471	0.679853247292
B	3.585929973207	8.154178911086	-0.801653975483
H	-2.127831894429	0.544949658466	0.674907011019
H	3.401256430410	6.917785599352	-0.796331069969
H	-1.403309569246	-1.139328090854	1.413144175434
H	2.676556933486	8.601757733720	-1.535106267126
H	-3.421269402486	-1.023676271197	0.999200473566
H	4.694518739077	8.486477021798	-1.121066536787
B	-0.670099897605	0.981180926132	-2.268660393249
B	1.943378616551	6.481573825072	2.146869660805
H	-0.973984958309	-0.216593577980	-2.161637301414
H	2.247270180528	7.679335899750	2.039730935327
H	-1.181394273962	1.566821405893	-1.289230573149
H	2.456001432617	5.895487679341	1.168398002480
H	-0.999511523868	1.468931879800	-3.321837176071
H	2.271613020242	5.994403326886	3.200681590392
B	1.959484736868	2.092182671716	-0.003727375953
B	-0.686156046370	5.370622050906	-0.118013295657
H	0.716456086251	2.274091652427	0.086054296780
H	0.556865972287	5.188690190676	-0.207785602600
H	2.379306265462	2.607844652269	-1.021151480825

H	-1.105991537430	4.854970396042	0.899406467932
H	2.511572667162	2.508520825507	0.995027957882
H	-1.238237175883	4.954285536818	-1.116768985693

Complex 3

U	-0.001697495634	-0.000222671502	-0.007831445404
S	2.878319254532	0.009597763076	-0.125845129200
C	1.345116363347	0.422029228619	2.524661712700
C	4.856397063763	1.303404343316	1.312698899613
C	-0.313292730703	-1.357118188691	2.532430234163
H	-0.544346404104	-2.418572019577	2.527964444808
C	-1.365233515465	-0.426247620165	2.517413239562
C	1.035032418363	-0.962819146592	2.543592340777
C	3.535228405329	0.828390130891	1.327434442193
C	5.646555505559	1.379359986045	0.045146885066
C	5.708029356143	2.537120980319	-0.720690565545
C	6.578651443162	2.608567949062	-1.803754473653
H	6.637909993283	3.514037997387	-2.403619541597
C	7.385478741558	1.521552885464	-2.119532938436
C	7.321823569449	0.364030772257	-1.351977014296
H	7.957083516274	-0.486309709252	-1.594078408821
C	6.453522420165	0.293284841763	-0.270361097482
C	4.680040255001	1.790514928165	3.680655081747
H	5.134405832603	2.150518517127	4.601687657183
C	0.305114399650	1.387768914456	2.542417680460
C	2.772714283895	0.876381617047	2.516367002783
C	-1.028240117844	0.928907144725	2.520460660158
H	-1.830361638742	1.665124536287	2.505528809578
C	3.362148899835	1.343878326979	3.678134507836
H	2.783657745522	1.363366094840	4.600192720813
C	-3.073858581690	-2.322157447696	2.308427212652
H	-2.666000558791	-2.933384948673	3.124237339768
H	-2.631003887372	-2.662024888521	1.364908415738
H	-4.149781396641	-2.523025310102	2.259470365947
C	2.141604090372	-1.993077875139	2.704469930670
H	3.016810564497	-1.625449853721	2.154889376849
C	0.607080748255	2.872761719029	2.682161473982
H	1.655366868252	3.022770612164	2.398055750793
C	5.406962136343	1.769652735656	2.501046993304
H	6.436039392372	2.126823318449	2.487774401795
C	-0.255539367796	3.789555054158	1.809779328185
H	-1.323496034015	3.703560245624	2.043171312547
H	-0.125363201799	3.578700568145	0.743886186572
H	0.033099743467	4.832362434769	1.985262139348
C	-2.838397467298	-0.838098507576	2.549511195269
H	-3.328750348275	-0.279994059193	1.737556079726
C	2.508023638487	-2.075776189310	4.191999680281
H	2.830106970233	-1.108223859412	4.589829969316
H	3.327821947636	-2.788788969953	4.340669213337
H	1.648698715365	-2.420905023198	4.781194978307
C	6.410629233226	-1.019293975137	0.548637733123
H	5.696032817903	-0.879394301335	1.368690537978
C	0.442553926605	3.264763164179	4.161067763748

H	0.730881967616	4.312013399300	4.310205912915
H	1.055853968109	2.646699856235	4.824359536815
H	-0.603651728209	3.154174526019	4.474150922002
C	-3.477115670821	-0.401716080167	3.870592576302
H	-3.012989274391	-0.924582626105	4.716877650656
H	-4.547077304279	-0.640140690992	3.871136393290
H	-3.374415354922	0.675446925416	4.043925181158
C	4.842297726921	3.760665807001	-0.408040838589
H	3.946345592200	3.393630985104	0.112660932558
C	7.860394399192	0.975736285985	-4.551060726575
H	7.744026688777	-0.102224554321	-4.380162717368
H	8.558057300789	1.115053006602	-5.386369125823
H	6.882265807004	1.370758527748	-4.850550445755
C	1.809488273574	-3.396711302583	2.179746394630
H	1.029188370981	-3.882642115878	2.779062138815
H	2.704150665613	-4.025832600116	2.243854403822
H	1.483914703853	-3.376568061755	1.134282244007
B	1.998021948106	1.406245405137	-1.141356876455
C	5.577264345041	4.713145896939	0.543240515915
H	5.842957872343	4.221823267023	1.485127478054
H	4.953057015025	5.584195928826	0.781205280085
H	6.504026620150	5.077884978001	0.081116013147
C	7.777088005442	-1.345339142934	1.174002921929
H	8.539199402621	-1.510241209745	0.402167819683
H	7.714121026641	-2.257227888743	1.780921112299
H	8.125137401351	-0.528501283217	1.817752553171
C	5.908058579886	-2.187474552212	-0.313657734259
H	4.930041742690	-1.958649558248	-0.750389080841
H	5.813328415201	-3.099017454758	0.290518668783
H	6.604979343866	-2.402351986317	-1.133782181617
B	-0.297399944813	-2.291224166871	-1.164586133130
C	4.365808368655	4.527611712514	-1.645236324699
H	5.187484280953	5.069725489303	-2.130137985872
H	3.615577473201	5.272070403565	-1.352927092242
H	3.910636312736	3.860958060082	-2.385224569526
B	-1.978812305074	1.348752494943	-1.028028378307
C	9.775039889479	1.092939654067	-2.962028565592
H	10.468918844247	1.309373940861	-3.783228011789
H	9.738955033515	0.003045422189	-2.843072975457
H	10.191239578976	1.518627698840	-2.040813546802
C	8.383882417309	1.664904963784	-3.277302807578
H	8.488543633995	2.739440442994	-3.481616971247
H	-1.162207694202	0.933024686099	-1.875092405709
H	-1.196963238942	-2.009705508669	-0.336210220647
H	1.273896212762	0.742214634634	-1.904558722404
H	1.258307780519	2.011002214311	-0.336231947028
H	2.726245350344	2.146612086905	-1.740477094890
H	-2.873646810317	2.007210961626	-1.490532117560
H	-2.363082592153	0.324840263116	-0.418283451186
H	-1.297081220527	1.972802320972	-0.181711667194
H	-0.454744449200	-3.359908952374	-1.694253016565
H	-0.278506380742	-1.324475095518	-1.954733472082
H	0.769204094632	-2.200919659874	-0.515632529486

Complex 4a

U	0.061744487712	-0.065182348385	-0.055782124454
S	2.750691358713	-0.194526552572	-0.289902342255
C	5.009550303680	0.028724123204	1.252843962020
C	3.599937898011	-0.052792605708	1.253139841013
C	2.905425542897	0.001807895888	2.475328948915
C	3.602770977142	0.154645346158	3.676627853407
H	3.045989392483	0.232100704892	4.610272058633
C	4.992369419577	0.230622381508	3.679655879684
H	5.533625641677	0.349188881156	4.616410461350
C	5.681905981700	0.164806596888	2.470500308257
H	6.769242169007	0.230003628893	2.454695832145
C	1.419135020021	-0.017569071639	2.457234487427
C	0.704311779060	-1.245880217820	2.478707047462
C	-0.702511299024	-1.207305001674	2.427042158152
H	-1.243196963352	-2.148759793820	2.434090185925
C	-1.425315542277	-0.004766957418	2.383126759008
C	-0.691521062539	1.195830935273	2.368117310158
H	-1.229641675825	2.142603039516	2.339589752468
C	0.714890174829	1.216035990936	2.416222525879
C	1.435506217704	-2.567477111698	2.644156264335
H	2.388403671021	-2.467986726181	2.105506434131
C	0.701238041717	-3.791329279980	2.102042096425
H	-0.192032048919	-4.027560534203	2.695044641681
H	1.362779382927	-4.662715508372	2.161402983971
H	0.401324158061	-3.669788221235	1.056713667182
C	1.747543827797	-2.783365584352	4.132239272155
H	0.819176296058	-2.849887585362	4.714413141486
H	2.357395165342	-1.975300057769	4.546215795903
H	2.298537081787	-3.721635413401	4.266943719660
C	-2.944728286084	0.036891236770	2.388445067028
H	-3.210348303713	0.673551032751	3.247906186501
C	-3.497529200087	0.713576190575	1.131468912637
H	-3.286941701363	0.114134437269	0.234568726196
H	-3.076515393742	1.710586978483	0.967132349734
H	-4.586468664420	0.815806449093	1.202866206391
C	-3.596239152416	-1.323687733379	2.604191001131
H	-4.683249382178	-1.206390972775	2.674978008684
H	-3.253737559801	-1.804238019936	3.528184815053
H	-3.396136611360	-2.002389811014	1.764640743237
C	1.447345533676	2.545184942564	2.450769980433
H	2.515341961074	2.315539620293	2.531665104823
C	1.250935428432	3.353639842050	1.169253408495
H	1.603237434594	2.802496920873	0.288854321200
H	1.831549330919	4.282543811496	1.224380749161
H	0.201818482395	3.624036637775	1.002746378569
C	1.048740123495	3.345545307144	3.691353176013
H	-0.008793326735	3.638065397355	3.663398496605
H	1.642764637137	4.264912539458	3.752844037409
H	1.216563968407	2.771674713482	4.610618215723
C	5.957902581716	1.193508799435	-0.750321292799
C	6.715602825773	1.158817064475	-1.920520582447
H	6.860141776480	2.083734050562	-2.480076485078
C	7.284927785352	-0.023152161820	-2.399035367523

C	7.078116811299	-1.191825896126	-1.667781346258
H	7.505733193500	-2.122973919943	-2.040912755566
C	6.335972605197	-1.205844976112	-0.483411199400
C	5.780291886758	0.001255476472	-0.021046004920
C	6.083227340611	-2.517455986128	0.235823354090
H	5.705762920394	-2.274230181677	1.237531012798
C	4.989538951429	-3.312394574110	-0.483562910821
H	5.313356092613	-3.592540828599	-1.494628549616
H	4.072544305409	-2.719220628890	-0.580012350758
H	4.753487971732	-4.235002594937	0.063204323852
C	7.350503021203	-3.351605898051	0.417714412698
H	7.137504299950	-4.243018066079	1.020983332974
H	8.137753880985	-2.778231516778	0.921972903434
H	7.752291538810	-3.698352252024	-0.542645578523
C	8.100655552010	-0.037669604637	-3.675023097675
H	8.401447396328	-1.081716236177	-3.848054487056
C	7.275492804854	0.411985336809	-4.881579147310
H	6.957528579625	1.457349911833	-4.777512877741
H	6.372526789828	-0.199400886225	-4.992867877418
H	7.861028424285	0.333131956664	-5.806343170302
C	9.377028129253	0.793196422052	-3.528240102342
H	9.991472006826	0.731879304229	-4.435555125937
H	9.979760888997	0.445329507129	-2.681150828984
H	9.140828427076	1.851387086237	-3.356328250634
C	5.357558086141	2.505895530135	-0.278364790619
H	4.701948100102	2.277264181634	0.571701853332
C	4.484550516374	3.155009677047	-1.351721529598
H	3.993399190909	4.053582987260	-0.956701433821
H	3.707902590612	2.459783026223	-1.690011125960
H	5.073369873865	3.460623281271	-2.226140094136
C	6.445224175461	3.454376448445	0.229463137929
H	7.148622909270	3.715050917958	-0.572101193877
H	7.021728245061	2.994264839406	1.040996095840
H	6.006233550468	4.386831945378	0.607314954479
B	-0.980745530974	1.981066209812	-1.570730625937
H	-1.564297947166	0.890672153030	-1.755576866631
H	-1.029204934093	2.147757431674	-0.322346188843
H	-1.425542009854	2.907164819037	-2.197875624125
H	0.234522837951	1.737507905493	-1.789003950770
B	-0.617845503996	-2.259346256844	-1.118532851713
H	0.493295468832	-2.304608754162	-0.553059748276
H	-0.952121709697	-3.317679358347	-1.583356131875
H	-1.423043275291	-1.854609295273	-0.246417698635
H	-0.537548137481	-1.342586869810	-1.956810944652

Complex 4b

U	0.001197269104	0.000697838217	-0.002366110622
S	2.722626891282	-0.000243386272	-0.003444503043
U	-1.858277796117	-4.341836442140	-2.205199154303
S	-4.579707418308	-4.340895148634	-2.204120854736
C	1.137145891056	-0.003689248562	2.647368748799
C	0.186196459744	-1.080800544013	2.680745715599
C	-1.158939482576	-0.776560299069	2.501619488565

H	-1.896436581262	-1.589012973217	2.533309676134
C	-1.635558787132	0.521803411440	2.304055256801
C	-0.682768771832	1.574738016774	2.318499798817
H	-1.032067560486	2.605288546361	2.176399675810
C	0.681322495540	1.334885135296	2.480089331518
C	0.622951520390	-2.485355599409	3.036003422187
H	1.706016080130	-2.560696580719	2.825854490104
C	-0.100390089786	-3.592928468300	2.282827724981
H	0.104245016891	-4.563008667025	2.771788134708
H	0.196631504804	-3.730837260235	1.227866502314
H	-1.198713048225	-3.473939619574	2.295134477576
C	0.409440824476	-2.675441672157	4.537743527728
H	-0.667799682477	-2.632686669570	4.785532973806
H	0.918807251292	-1.898128573855	5.132736114508
H	0.792101032171	-3.658767964247	4.864686943825
C	-3.113770252692	0.841850168004	2.223427329261
H	-3.639395218303	-0.134920285144	2.230152533113
C	-3.488094546647	1.536459718672	0.950326817368
H	-4.569233288172	1.742360569743	0.880050106493
H	-3.215536362954	0.763760701529	0.200389207756
H	-2.921942700776	2.461949898179	0.756776200017
C	-3.558620762839	1.623915430170	3.432425659432
H	-3.089367059202	2.625368492004	3.460096617573
H	-3.301884246984	1.112976385135	4.378406584493
H	-4.653135734630	1.775893486846	3.417036171157
C	1.645956146736	2.497671535040	2.609688285898
H	2.657275602912	2.093175101607	2.421759027678
C	1.395040702598	3.624282442968	1.612711976897
H	1.032339121960	3.211902943747	0.650698581474
H	2.314257585327	4.200961827446	1.416238663441
H	0.622249876643	4.329744942107	1.971771435727
C	1.583617859449	3.019195348169	4.026314455419
H	0.568494637154	3.389560851552	4.263823983396
H	2.287020120800	3.860233949676	4.167738014088
H	1.839964510732	2.241845992907	4.768773010997
C	3.377384715847	-0.414256415538	1.588747094747
C	2.603805211811	-0.278793437579	2.759069977155
C	3.194602463762	-0.489051377473	3.995359517133
H	2.588038802280	-0.382055742068	4.903756866255
C	4.535159006618	-0.813355345035	4.092565955440
H	4.994904369958	-0.962783892043	5.077075534309
C	5.285393775245	-0.983793195476	2.940824481745
H	6.338582182674	-1.286322550976	3.006529361267
C	4.725397538310	-0.801997118243	1.681458869646
C	5.514626182627	-1.114134605443	0.436042098953
C	5.493258184606	-2.378116868750	-0.096038141026
C	6.142241329612	-2.599688443108	-1.333392517815
H	6.078802857249	-3.593667718681	-1.798944938875
C	6.854303757524	-1.595635248297	-1.976717912624
C	6.906211995165	-0.385799578821	-1.396780003450
H	7.454681588628	0.430074557004	-1.886200510961
C	6.253976099926	-0.100880849622	-0.196422570960
C	4.745834167420	-3.528100497792	0.544475386363
H	4.375894862006	-3.176879555218	1.526201071252
C	3.533840870504	-3.962261100164	-0.270585393756

H	2.864168158220	-3.107928919884	-0.475599816751
H	2.946493436428	-4.737658198538	0.256409863138
H	3.838642144548	-4.384971510662	-1.246557984433
C	5.666902096784	-4.714561651469	0.792150547692
H	6.048077624743	-5.136410636883	-0.156208839866
H	5.131733050649	-5.525553432196	1.320220793357
H	6.543691148411	-4.431672723418	1.402632106817
C	7.481290185887	-1.834646781939	-3.341967602440
H	6.806404016748	-2.590792237487	-3.808116790497
C	7.424206022159	-0.642579605831	-4.291940906135
H	8.117716517125	0.164163751327	-3.985119497183
H	6.410664098692	-0.208485832663	-4.351175451823
H	7.727114399523	-0.955567465632	-5.307411450336
C	8.752450709632	-2.387323178829	-3.262493790383
H	8.791980438243	-3.306568495171	-2.645745041744
H	9.488256802787	-1.692387351472	-2.803167053578
H	9.172245817043	-2.658298719373	-4.252484690636
C	6.332330340709	1.293344542830	0.338655376758
H	5.689695971899	1.339847109109	1.239610166138
C	5.777587661968	2.356571385549	-0.646362179816
H	6.420765684407	2.452051812057	-1.540822357504
H	5.731012006570	3.346480688579	-0.157340960215
H	4.761805875009	2.079845452503	-0.979627068341
C	7.735345790355	1.638924391844	0.756901607054
H	8.125274638601	0.928412989026	1.509162543549
H	7.788279119561	2.653787604549	1.194150135729
H	8.432292495315	1.616138192893	-0.102438203572
B	-0.576161858856	1.877474925922	-1.637982641010
H	-0.895928452903	2.201864207565	-0.481660539889
H	-1.089494881571	0.770084156296	-1.898811712669
H	0.646246850573	1.649663278941	-1.622206080464
H	-0.899113644090	2.713971466160	-2.423113831978
B	-0.065911732987	-2.255732901786	-1.311066561823
H	0.088902803926	-1.195096790457	-1.939503650047
H	0.453376445797	-3.151294821535	-1.884129015924
H	0.570399666001	-2.193939915742	-0.256950616158
C	-2.994226336194	-4.337449379561	-4.854944060700
C	-2.043276871022	-3.260338099579	-4.888311015049
C	-0.698140942509	-3.564578379924	-4.709184740647
H	0.039339052044	-2.752116167246	-4.740833950756
C	-0.221521681433	-4.862942112794	-4.511630468450
C	-1.174311725796	-5.915876678094	-4.526065023921
H	-0.825039891060	-6.946436754831	-4.383946601003
C	-2.538412977342	-5.676023759015	-4.687654601064
C	-2.480031880714	-1.855783047166	-5.243578758544
H	-3.563103859561	-1.780460231412	-5.033434140234
C	-1.756690256035	-0.748210174235	-4.490393059879
H	-1.961616253759	0.221904889022	-4.979157347265
H	-2.053539055607	-0.610536724516	-3.435355585628
H	-0.658356819343	-0.867031791468	-4.502975556910
C	-2.266521129649	-1.665697009707	-6.745318860711
H	-1.189296709249	-1.708520182897	-6.993143113083
H	-2.775959354624	-2.442995192787	-7.340277753103
H	-2.649164893101	-0.682365888498	-7.072261260247
C	1.256689774565	-5.182988908513	-4.430992481572

H	1.782302754467	-4.206219855166	-4.437718687927
C	1.631014003857	-5.877598449404	-3.157891945332
H	2.712154551162	-6.083433953762	-3.087527834175
H	1.358305897050	-5.104841815579	-2.408051657135
H	1.064871918857	-6.803100219683	-2.964355815169
C	1.701540289435	-5.965054211924	-5.639990783325
H	1.232191777465	-6.966470600245	-5.667676528738
H	1.444832764300	-5.454109537346	-6.585974886541
H	2.796037590670	-6.117126939912	-5.624585007902
C	-3.503036649763	-6.838810140215	-4.817253572328
H	-4.514372780689	-6.434343267589	-4.629324819509
C	-3.252121265493	-7.965421039833	-3.820277238867
H	-2.889482689926	-7.553056891193	-2.858229431664
H	-4.171334520594	-8.542154525325	-3.623901221712
H	-2.479294281608	-8.670865782074	-4.179311200077
C	-3.440698334267	-7.360333992432	-6.233889727002
H	-2.425581264359	-7.730698786480	-6.471404970740
H	-4.144104870876	-8.201380958485	-6.375291855307
H	-3.697085939562	-6.583001954284	-6.976347211080
C	-5.234465180590	-3.926882139397	-3.796322481753
C	-4.460885639388	-4.062345144035	-4.966635338073
C	-5.051682846834	-3.852087207078	-6.202924900080
H	-4.445159366375	-3.959179915619	-7.111333506961
C	-6.392239378498	-3.527783208846	-6.300131387231
H	-6.852026308132	-3.378470634746	-7.284637966027
C	-7.142474179385	-3.357345323109	-5.148389940296
H	-8.195693684355	-3.054914321079	-5.214099339265
C	-6.582477985860	-3.539141395785	-3.889024308495
C	-7.371706666642	-3.227003871701	-2.643607570154
C	-7.350338658804	-1.963021601663	-2.111527347203
C	-7.999321843863	-1.741449995924	-0.874172997056
H	-7.935907033597	-0.747472918517	-0.408619913641
C	-8.711384323398	-2.745503174665	-0.230857607543
C	-8.763292569004	-3.955338850462	-0.810795503357
H	-9.311783977400	-4.771211850193	-0.321383519955
C	-8.111056632070	-4.240257599105	-2.011142911439
C	-6.602914594785	-0.813038008858	-2.752050864756
H	-6.232996992103	-1.164280219882	-3.733773265778
C	-5.390921310443	-0.378877415095	-1.936980049900
H	-4.721384060424	-1.233278338447	-1.731801024260
H	-4.803469123387	0.396380370712	-2.464049189999
H	-5.695769398105	0.043988992002	-0.961087694468
C	-7.523982483500	0.373423164561	-2.999726082685
H	-7.905215072014	0.795228430184	-2.051368200968
H	-6.988835298769	1.184438049262	-3.527770810294
H	-8.400755616678	0.090517357216	-3.610230931976
C	-9.338370793312	-2.506491597625	1.134402055821
H	-8.663476533586	-1.750364164617	1.600555891607
C	-9.281286689248	-3.698558761883	2.084375377972
H	-9.974816269297	-4.505292603106	1.777551752613
H	-8.267760298612	-4.132680673901	2.143604885786
H	-9.584213352987	-3.385588384894	3.099847505178
C	-10.609551260559	-1.953715159568	1.054938099006
H	-10.649041684952	-1.034473161730	0.438192200650
H	-11.345398041442	-2.648617720311	0.595615070371

H	-11.029326832398	-1.682742955558	2.044939815431
C	-8.189410885758	-5.634483004914	-2.546230849592
H	-7.546798371046	-5.681001471928	-3.447193310598
C	-7.634668263152	-6.697709838226	-1.561203254844
H	-8.277954968332	-6.793280754066	-0.666825413512
H	-7.587993302236	-7.687600896311	-2.050259263797
H	-6.618944335661	-6.420941320516	-1.227813834301
C	-9.592426320696	-5.980062822180	-2.964467125286
H	-9.982378450357	-5.269592323895	-3.716752276014
H	-9.645381101633	-6.994946371891	-3.401676256604
H	-10.289384730824	-5.957257722482	-2.105131300168
B	-1.280938756203	-6.218523486302	-0.569562634722
H	-0.960931165450	-6.542475987083	-1.725917770738
H	-0.767886898773	-5.111113849543	-0.308240616969
H	-2.503430953644	-5.991096653130	-0.585574584906
H	-0.957929975126	-7.055428664698	0.215120387255
B	-1.791168796574	-2.085405711186	-0.896508709665
H	-1.946281392330	-3.146003880769	-0.267884574335
H	-2.310637325508	-1.190074824478	-0.323211451150
H	-2.427709585113	-2.147539081097	-1.950421460143

Complex 5

U	0.000876144424	-0.000255578190	-0.000250701032
S	-1.218539057763	-2.508416309711	0.005289824162
S	-1.218088567501	2.516983673397	0.005312860771
C	-1.413785238952	-3.343871735993	-1.538116473367
C	-1.114170559720	-2.709343004561	-2.754494396617
C	-1.326061123785	-3.357578451797	-3.966830921595
H	-1.090833762469	-2.842952945231	-4.898280557665
C	-1.846045454503	-4.646011760403	-3.985140082356
H	-2.028580589295	-5.151140259322	-4.931933045523
C	-2.107279809868	-5.290023853751	-2.787069742419
H	-2.517305823148	-6.299845791008	-2.792387670441
C	-1.897052940366	-4.670108350606	-1.556613445463
C	-0.577647812104	-1.315101246768	-2.719473688776
C	0.809470606580	-1.075655080092	-2.828883859439
C	1.267449628572	0.256913802216	-2.820468574920
H	2.334444647379	0.439106209171	-2.884412935124
C	0.389412480628	1.342282225086	-2.751929733722
C	-0.985236543548	1.072685094056	-2.676118862615
H	-1.676053257374	1.913698585747	-2.640438659028
C	-1.487904375622	-0.230149931468	-2.659766960054
C	1.768204310962	-2.240149716060	-3.111887981001
H	1.240019991903	-2.839417082208	-3.867584049004
C	2.021505464813	-3.167397815907	-1.926066013340
H	2.717636997928	-2.707008035631	-1.218115160017
H	2.461171751854	-4.107495354693	-2.280580092264
H	1.105059085136	-3.407360717050	-1.380594494097
C	3.096901693586	-1.813025874802	-3.723891071571
H	3.713220636377	-1.264112403092	-3.001911126161
H	2.961654505319	-1.186978938552	-4.614902000258
H	3.661200153922	-2.703493767244	-4.024589745554
C	0.871769603052	2.786409543439	-2.871872271178

H	0.243702898442	3.371942674866	-2.183723524477
C	2.327250988594	2.972037252510	-2.476808351070
H	2.527311427015	2.604594682374	-1.464765715711
H	2.593374258526	4.033836510877	-2.508751124619
H	3.005698618818	2.451860825944	-3.165234826747
C	0.641024608080	3.284296702276	-4.285264753919
H	1.242616262511	2.715197519032	-5.006558808133
H	0.925163158922	4.340762269795	-4.371103561223
H	-0.410676155346	3.198956584045	-4.585000049117
C	-2.990103122920	-0.480405011571	-2.717544191820
H	-3.167781638281	-1.464637334208	-2.264778351805
C	-3.797760207668	0.551463864717	-1.942111140138
H	-3.417809712401	0.691178254582	-0.924583725562
H	-4.845250145329	0.234931630875	-1.873542677077
H	-3.787354659407	1.535346395045	-2.427519639074
C	-3.435914553239	-0.529558388460	-4.170932938586
H	-3.239605934736	0.428576475924	-4.670051360581
H	-4.513450948331	-0.726440112018	-4.233617525969
H	-2.920451085240	-1.316952903854	-4.730206444185
C	0.894806746704	-2.765548730974	2.838003802182
H	0.456329311626	-3.280056536491	1.969204632057
C	-1.366039375597	3.376949921199	1.536047347697
C	-1.032442724204	2.758304429222	2.754125859727
C	-1.194578949716	3.437673438264	3.960058603650
H	-0.941055275928	2.935745977543	4.893878063284
C	-1.660602201226	4.747825168928	3.970884797593
H	-1.784226749632	5.279276689977	4.912655623895
C	-1.974699944260	5.365668836857	2.768707535585
H	-2.352360494411	6.388027934079	2.765546652891
C	-1.835190824605	4.711161906387	1.552283456822
C	-0.518297320788	1.351788438740	2.730952295504
C	-1.435720320473	0.280300884622	2.682958708949
C	-0.940998575965	-1.036593471983	2.686254312508
H	-1.648689329960	-1.863400174684	2.661568879892
C	0.420872750847	-1.317335797575	2.741210118939
C	1.302595179378	-0.237155155234	2.809821444905
H	2.366702913599	-0.434954092313	2.871037333059
C	0.868173750030	1.095563731088	2.824199787864
C	-2.939007573754	0.516825043828	2.814963655005
H	-3.136024972179	1.543739342863	2.482047023498
C	-3.799809511749	-0.427984065467	1.991624214021
H	-3.509609975407	-0.434580616344	0.935782860785
H	-4.850949226721	-0.121867257193	2.049615418412
H	-3.740325392936	-1.462558940283	2.349914216970
C	-3.301302052017	0.403700552496	4.293750012723
H	-3.088180499092	-0.605740645497	4.668828843841
H	-4.370023791993	0.601232356775	4.444200236181
H	-2.739070890290	1.118220673564	4.905656956564
C	1.853929990327	2.239944198617	3.079838072690
H	1.331141821964	2.906137444781	3.779847679704
C	2.167303261796	3.065111773591	1.839661870483
H	2.673494520049	3.994872447579	2.127063818068
H	1.263422716735	3.332066307278	1.281864869030
H	2.827229194095	2.512074281497	1.163600673267
C	3.144725928078	1.786825343233	3.759218537647

H	2.949897977069	1.201762199385	4.666491706956
H	3.733543903903	2.665915293591	4.046328184300
H	3.765529448526	1.186053945490	3.083251006064
C	-2.220267577257	5.397022294419	0.273611971106
C	-1.259848225171	6.066579747423	-0.497773544111
C	-1.648999505771	6.642440167969	-1.703535793381
H	-0.902450121095	7.145583094427	-2.317479618345
C	-2.949585633797	6.571668920661	-2.176169286690
C	-3.896633686058	5.933840161775	-1.380622770627
H	-4.928626268208	5.869745321811	-1.725284380085
C	-3.561244275872	5.351744465057	-0.154403990827
C	0.186842437714	6.179237953048	-0.037320517157
H	0.416163773023	5.262731316851	0.523615097591
C	1.176665248377	6.272964581522	-1.201036802552
H	1.130748938794	7.251471973040	-1.695736243869
H	0.985078524373	5.502089614214	-1.953861132510
H	2.202341147268	6.144366626263	-0.834169003052
C	0.382499206596	7.367986140489	0.897407432567
H	0.131013176866	8.305953757943	0.384901507022
H	1.425956859133	7.432821163690	1.232133486686
H	-0.251235328496	7.290940213409	1.787649432206
C	-3.270373091636	7.196113895485	-3.529976936214
H	-2.363859851200	7.031007451813	-4.136262823460
C	-4.401804789556	6.539658680879	-4.268142207482
H	-5.368626123222	6.707291870185	-3.773598400980
H	-4.256048563374	5.455731620891	-4.350762599125
H	-4.489289855164	6.947393164533	-5.282922711455
C	-3.443438616246	8.676568813257	-3.401831109822
H	-4.323004141223	8.919834788655	-2.789240851704
H	-3.581210716128	9.152370079578	-4.382240334540
H	-2.575947433050	9.144403270376	-2.921422167430
C	-4.641577747840	4.667037560604	0.660133784465
H	-4.183683000782	4.331932564247	1.599093723972
C	-5.184662770956	3.438019482063	-0.045926584882
H	-5.927807373839	2.924154848104	0.577968393526
H	-4.376522981781	2.732864315110	-0.268335146130
H	-5.671860103370	3.701246443271	-0.994336589373
C	-5.764373685589	5.633076591800	1.012386420913
H	-5.383918708320	6.513957456772	1.543797027708
H	-6.509649558130	5.145482618585	1.653997196521
H	-6.285591220805	5.988913613148	0.114291264341
B	2.867984680366	-0.128732554697	-0.000027913414
C	-2.315700848477	-5.395336823126	-0.260513551056
C	-3.627767185977	-5.362791322064	0.196772689290
C	-3.960069554748	-5.984757065258	1.394308556066
H	-4.983773040206	-5.956454759801	1.764557009868
C	-2.982715667780	-6.640958106070	2.133668042311
C	-1.671469235204	-6.673883528851	1.674461758817
H	-0.908223693511	-7.188004256452	2.256027234610
C	-1.336736951537	-6.051998000380	0.478056038692
C	-4.736802289221	-4.618538385376	-0.555534845214
H	-4.367946632093	-4.432736305055	-1.572962489446
C	-4.966532345286	-3.258280721620	0.100476521731
H	-5.336752803676	-3.377434629766	1.127826153201
H	-5.706348467861	-2.668071947335	-0.457235927683

H	-4.030371705175	-2.690939801744	0.146148919949
C	-6.037595691933	-5.414171044918	-0.680551993121
H	-5.862880192455	-6.400255317557	-1.127549434256
H	-6.750602178926	-4.873177103396	-1.315063980651
H	-6.521471239928	-5.567834242692	0.292275620418
H	2.150887288448	0.897566005675	-0.111453050702
H	2.077787012127	-1.095992389866	0.136929247772
H	3.551586258979	-0.024866583706	0.997931195474
H	3.510158459733	-0.270788581489	-1.019134565720
C	-3.359698299577	-7.270700549555	3.480961021755
H	-4.451426920661	-7.124213722419	3.547062946897
C	-2.784772351076	-6.511787278440	4.610071216180
H	-1.685865008861	-6.558104319900	4.628618627833
H	-3.137863190425	-6.892542211430	5.578417188443
H	-3.058086365950	-5.451685873270	4.547286121077
C	-3.176653638479	-8.781196951097	3.481975644407
H	-3.715592087578	-9.231262969471	2.641173669210
H	-3.557891584230	-9.222817356690	4.411985783674
H	-2.119822538381	-9.068799993284	3.391165360439
C	0.107970358347	-6.132185017273	-0.061151923603
H	0.286486883625	-5.185895346025	-0.588047684309
C	1.160520768876	-6.230934526102	1.043955000197
H	1.005757970094	-5.466252391051	1.812301004263
H	2.163284851562	-6.089854452175	0.622426150921
H	1.149820425733	-7.213352348223	1.534156477093
C	0.295573586903	-7.261477220877	-1.069625101841
H	0.106624900841	-8.236819182640	-0.601464178488
H	1.322953553220	-7.263740642933	-1.455845185198
H	-0.383408814421	-7.160088091064	-1.922714763149
C	0.327736690234	-3.424413696812	4.104514526868
H	0.707248007308	-2.926052305209	5.006204477778
H	0.629097848146	-4.478047629334	4.147012345334
H	-0.767348612583	-3.398051005648	4.134357546816
C	2.391541594834	-2.953612770193	2.811468663286
H	2.852913624976	-2.502765145829	1.924788380698
H	2.632493730334	-4.022451539050	2.802640763329
H	2.870811997073	-2.520390740990	3.700120716602

S9. References

(1) Fetrow, T. V.; Grabow, J. P.; Leddy, J.; Daly, S. R. Convenient Syntheses of Trivalent Uranium Halide Starting Materials without Uranium Metal. *Inorg. Chem.* **2021**, *60* (11), 7593-7601. DOI: https://doi.org/10.1021/acs.inorgchem.1c00598

(2) Patel, D.; Wooles, A. J.; Hashem, E.; Omorodion, H.; Baker, R. J.; Liddle, S. T. Comments on reactions of oxide derivatives of uranium with hexachloropropene to give UCl_4. *New J. Chem.* **2015**, *39* (10), 7559-7562. DOI: https://doi.org/10.1039/C5NJ00476D

(3) Barnett, B. R.; Mokhtarzadeh, C. C.; Lummis, P.; Wang, S.; Queen, J. D.; Gavenonis, J.; Schüwer, N.; Tilley, T. D.; Boynton, J. N.; Weidemann, N.; Agnew, D. W.; Smith, P. W.; Ditri, T. B.; Carpenter, A. E.; Pratt, J. K.; Mendelson, N. D.; Figueroa, J. S.; Power, P. P. TERPHENYL LIGANDS AND COMPLEXES. Inorganic Syntheses, 2018; pp 85-122.

(4) Twamley, B.; Haubrich, S. T.; Power, P. P. Element Derivatives of Sterically Encumbering Terphenyl Ligands. In *Advances in Organometallic Chemistry Volume 44*, Advances in Organometallic Chemistry, 1999; pp 1-65.

(5) Schnaars, D. D.; Wu, G.; Hayton, T. W. Reactivity of UH_3 with mild oxidants. *Dalton Trans.* **2008**, (44), 6121-6126. DOI: https://doi.org/10.1039/B809184F

(6) Carmichael, C. D.; Jones, N. A.; Arnold, P. L. Low-valent uranium iodides: straightforward solution syntheses of UI_3 and UI_4 etherates. *Inorg. Chem.* **2008**, *47* (19), 8577-8579. DOI: https://doi.org/10.1021/ic801138e

(7) Ortu, F. Rare Earth Starting Materials and Methodologies for Synthetic Chemistry. *Chem. Rev.* **2022**, *122* (6), 6040-6116. DOI: https://doi.org/10.1021/om060262v

(8) Emerson-King, J.; Gransbury, G. K.; Whitehead, G. F. S.; Vitorica-Yrezabal, I. J.; Rouzières, M.; Clérac, R.; Chilton, N. F.; Mills, D. P. Isolation of a Bent Dysprosium Bis(amide) Single-Molecule Magnet. *J. Am. Chem. Soc.* **2024**. DOI: https://doi.org/10.1021/jacs.3c12427

(9) Goodwin, C. A. P.; Reta, D.; Ortu, F.; Liu, J.; Chilton, N. F.; Mills, D. P. Terbocenium: completing a heavy lanthanide metallocenium cation family with an alternative anion

abstraction strategy. *Chem. Commun.* **2018**, *54* (66), 9182-9185. DOI: https://doi.org/10.1039/c8cc05261a

(10) Arnold, P. L.; Stevens, C. J.; Bell, N. L.; Lord, R. M.; Goldberg, J. M.; Nichol, G. S.; Love, J. B. Multi-electron reduction of sulfur and carbon disulfide using binuclear uranium(III) borohydride complexes. *Chem. Sci.* **2017**, *8* (5), 3609-3617. DOI: https://doi.org/10.1039/C7SC00382J

(11) Arliguie, T.; Belkhiri, L.; Bouaoud, S.-E.; Thuéry, P.; Villiers, C.; Boucekkine, A.; Ephritikhine, M. Lanthanide(III) and Actinide(III) Complexes $[M(BH_4)_2(THF)_5][BPh_4]$ and $[M(BH_4)_2(18\text{-crown-}6)][BPh_4]$ (M = Nd, Ce, U): Synthesis, Crystal Structure, and Density Functional Theory Investigation of the Covalent Contribution to Metal-Borohydride Bonding. *Inorg. Chem.* **2009**, *48* (1), 221-230. DOI: https://doi.org/10.1021/ic801685v

(12) Boronski, J. T.; Doyle, L. R.; Seed, J. A.; Wooles, A. J.; Liddle, S. T. f-Element Half-Sandwich Complexes: A Tetrasilylcyclobutadienyl–Uranium(IV)–Tris(tetrahydroborate) Anion Pianostool Complex. *Angew. Chem., Int. Ed.* **2020**, *59* (1), 295-299. DOI: https://doi.org/10.1002/anie.201913640

(13) Ephritikhine, M. Synthesis, Structure, and Reactions of Hydride, Borohydride, and Aluminohydride Compounds of the f-Elements. *Chem. Rev.* **1997**, *97* (6), 2193-2242. DOI: https://doi.org/10.1021/cr960366n

(14) Arnold, P. L.; Stevens, C. J.; Farnaby, J. H.; Gardiner, M. G.; Nichol, G. S.; Love, J. B. New chemistry from an old reagent: mono- and dinuclear macrocyclic uranium(III) complexes from $[U(BH_4)_3(THF)_2]$. *J. Am. Chem. Soc.* **2014**, *136* (29), 10218-10221. DOI: https://doi.org/10.1021/ja504835a

(15) Baudry, D.; Bulot, E.; Charpin, P.; Ephritikhine, M.; Lance, M.; Nierlich, M.; Vigner, J. Arene uranium borohydrides: synthesis and crystal structure of $(\eta\text{-}C_6Me_6)U(BH_4)_3$. *J. Organomet. Chem.* **1989**, *371* (2), 155-162. DOI: https://doi.org/10.1016/0022-328X(89)88022-2

(16) Haaland, A.; Shorokhov, D. J.; Tutukin, A. V.; Volden, H. V.; Swang, O.; McGrady, G. S.; Kaltsoyannis, N.; Downs, A. J.; Tang, C. Y.; Turner, J. F. C. Molecular Structures of Two Metal Tetrakis(tetrahydroborates), $Zr(BH_4)_4$ and $U(BH_4)_4$: Equilibrium Conformations and

Barriers to Internal Rotation of the Triply Bridging BH_4 Groups. *Inorg. Chem.* **2002**, *41* (25), 6646-6655. DOI: https://doi.org/10.1021/ic020357z

(17) Bernstein, E. R.; Hamilton, W. C.; Keiderling, T. A.; La Placa, S. J.; Lippard, S. J.; Mayerle, J. J. 14-Coordinate uranium(IV). Structure of uranium borohydride by single-crystal neutron diffraction. *Inorg. Chem.* **1972**, *11* (12), 3009-3016. DOI: https://doi.org/10.1021/ic50118a027

(18) Bernstein, E. R.; Keiderling, T. A.; Lippard, S. J.; Mayerle, J. J. Structure of uranium borohydride by single-crystal x-ray diffraction. *J. Am. Chem. Soc.* **1972**, *94* (7), 2552-2553. DOI: https://doi.org/10.1021/ja00762a082

(19) Charpin, P.; Marquet-Ellis, H.; Folcher, G. Uranium(IV) borohydride: A new crystalline form. *J. Inorg. Nucl. Chem.* **1979**, *41* (8), 1143-1144. DOI: https://doi.org/10.1016/0022-1902(79)80472-8

(20) Charpin, P.; Nierlich, M.; Vigner, D.; Lance, M.; Baudry, D. Structure of the second crystalline form of uranium(IV) tetrahydroborate. *Acta Crystallogr. C* **1987**, *43* (8), 1465-1467. DOI: https://doi.org/10.1107/S0108270187091431

(21) Schlesinger, H. I.; Brown, H. C. Uranium(IV) Borohydride. *J. Am. Chem. Soc.* **1953**, *75* (1), 219-221. DOI: https://doi.org/10.1021/ja01097a058

(22) Fazakerley, G. V.; Folcher, G.; Marquet-Ellis, H. Dissociation of uranium(III) and uranium(IV) borohydrides in solution: ^{11}B, ^{1}H NMR study. *Polyhedron* **1984**, *3* (4), 457-461. DOI: https://doi.org/10.1016/S0277-5387(00)84518-0

(23) *Rigaku Oxford Diffraction, (2023), CrysAlisPro Software system, Rigaku Corporation, Wroclaw, Poland.*; Rigaku Oxford Diffraction: 2023.

(24) Dolomanov, O. V.; Bourhis, L. J.; Gildea, R. J.; Howard, J. A. K.; Puschmann, H. OLEX2: a complete structure solution, refinement and analysis program. *J. Appl. Crystallogr.* **2009**, *42* (2), 339-341. DOI: https://doi.org/10.1107/s0021889808042726

(25) Sheldrick, G. M. Crystal structure refinement with SHELXL. *Acta Crystallogr. C* **2015**, *71* (Pt 1), 3-8. DOI: https://doi.org/10.1107/S2053229614024218

(26) Sheldrick, G. M. A short history of SHELX. *Acta Crystallogr. A* **2008**, *64* (Pt 1), 112-122. DOI: https://doi.org/10.1107/S0108767307043930

(27) *Persistence of Vision Raytracer (Version 3.6)*; Persistence of Vision Pty. Ltd.: 2004. https://www.povray.org.

(28) *GNU Image Manipulation Program (GIMP) (Version 2.10.38)*; The GIMP Team: 2024. https://www.gimp.org.

(29) *Inkscape: Open Source Scalable Vector Graphics Editor. https://inkscape.org/.*

(30) Clegg, W.; Blake, A. J.; Cole, J. M.; Evans, J. S. O.; Main, P.; Parsons, S.; Watkin, D. J. *Crystal Structure Analysis*; Oxford University Press, 2009. DOI: http://doi.org/10.1093/acprof:oso/9780199219469.001.0001.

(31) Ketkaew, R.; Tantirungrotechai, Y.; Harding, P.; Chastanet, G.; Guionneau, P.; Marchivie, M.; Harding, D. J. OctaDist: a tool for calculating distortion parameters in spin crossover and coordination complexes. *Dalton Trans.* **2021**, *50* (3), 1086-1096. DOI: https://doi.org/10.1039/D0DT03988H

(32) Buron-Le Cointe, M.; Hébert, J.; Baldé, C.; Moisan, N.; Toupet, L.; Guionneau, P.; Létard, J. F.; Freysz, E.; Cailleau, H.; Collet, E. Intermolecular control of thermoswitching and photoswitching phenomena in two spin-crossover polymorphs. *Phys. Rev. B* **2012**, *85* (6). DOI: https://doi.org/10.1103/PhysRevB.85.064114

(33) Lufaso, M. W.; Woodward, P. M. Jahn-Teller distortions, cation ordering and octahedral tilting in perovskites. *Acta Crystallogr. B* **2004**, *60* (Pt 1), 10-20. DOI: https://doi.org/10.1107/S0108768103026661

(34) McCusker, J. K.; Rheingold, A. L.; Hendrickson, D. N. Variable-Temperature Studies of Laser-Initiated $^5T_2 \rightarrow {}^1A_1$ Intersystem Crossing in Spin-Crossover Complexes: Empirical Correlations between Activation Parameters and Ligand Structure in a Series of Polypyridyl Ferrous Complexes. *Inorg. Chem.* **1996**, *35* (7), 2100-2112. DOI: https://doi.org/10.1021/ic9507880

(35) Marchivie, M.; Guionneau, P.; Letard, J. F.; Chasseau, D. Photo-induced spin-transition: the role of the iron(II) environment distortion. *Acta Crystallogr. B* **2005**, *61* (Pt 1), 25-28. DOI: https://doi.org/10.1107/S0108768104029751

(36) *SHAPE 2.1: Program for the Stereochemical Analysis of Molecular Fragments by Means of Continuous Shape Measures and Assosciated Tools*; 2013. http://www.ee.ub.edu.

(37) Poleschner, H.; Ellrodt, S.; Malischewski, M.; Nakatsuji, J.-y.; Rohner, C.; Seppelt, K. Trip2C6H3SeF: The First Isolated Selenenyl Fluoride. *Angew. Chem., Int. Ed.* **2012**, *51* (2), 419-422. DOI: https://doi.org/10.1002/anie.201106708

(38) Kabova, E. A.; Blundell, C. D.; Muryn, C. A.; Whitehead, G. F. S.; Vitorica-Yrezabal, I. J.; Ross, M. J.; Shankland, K. SDPD-SX: combining a single crystal X-ray diffraction setup with advanced powder data structure determination for use in early stage drug discovery. *CrystEngComm* **2022**, *24* (24), 4337-4340. DOI: https://doi.org/10.1039/D2CE00387B

(39) Coelho, A. An indexing algorithm independent of peak position extraction for X-ray powder diffraction patterns. *J. Appl. Crystallogr.* **2017**, *50* (5), 1323-1330. DOI: https://doi.org/10.1107/S1600576717011359

(40) Petříček, V.; Dušek, M.; Palatinus, L. Crystallographic Computing System JANA2006: General features. *Zeitschrift für Kristallographie - Crystalline Materials* **2014**, *229* (5), 345-352. DOI: https://doi.org/10.1515/zkri-2014-1737

(41) Macrae, C. F.; Sovago, I.; Cottrell, S. J.; Galek, P. T. A.; McCabe, P.; Pidcock, E.; Platings, M.; Shields, G. P.; Stevens, J. S.; Towler, M.; Wood, P. A. Mercury 4.0: from visualization to analysis, design and prediction. *J. Appl. Crystallogr.* **2020**, *53* (1), 226-235. DOI: https://doi.org/10.1107/S1600576719014092

(42) Ketkaew, R.; Tantirungrotechai, Y.; Harding, P.; Chastanet, G.; Guionneau, P.; Marchivie, M.; Harding, D. J. OctaDist: A tool for calculating distortion parameters in spin crossover and coordination complexes. *Dalton Trans.* **2021**, DOI: 10.1039/D1030DT03988H. DOI: https://doi.org/10.1039/D0DT03988H

(43) Neese, F. Software update: The ORCA program system—Version 5.0. *WIRES Comput. Mol. Sci.* **2022**, *12* (5), e1606. DOI: https://doi.org/10.1002/wcms.1606

(44) Perdew, J. P.; Ernzerhof, M.; Burke, K. Rationale for mixing exact exchange with density functional approximations. *J. Chem. Phys.* **1996**, *105* (22), 9982-9985. DOI: https://doi.org/10.1063/1.472933

(45) Adamo, C.; Barone, V. Toward reliable density functional methods without adjustable parameters: The PBE0 model. *J. Chem. Phys.* **1999**, *110* (13), 6158-6170. DOI: https://doi.org/10.1063/1.478522

(46) Grimme, S.; Antony, J.; Ehrlich, S.; Krieg, H. A consistent and accurate ab initio parametrization of density functional dispersion correction (DFT-D) for the 94 elements H-Pu. *J. Chem. Phys.* **2010**, *132* (15), 154104. DOI: https://doi.org/10.1063/1.3382344

(47) Grimme, S. Semiempirical GGA-type density functional constructed with a long-range dispersion correction. *J. Comput. Chem.* **2006**, *27* (15), 1787-1799. DOI: https://doi.org/10.1002/jcc.20495

(48) Neese, F.; Wennmohs, F.; Hansen, A.; Becker, U. Efficient, approximate and parallel Hartree–Fock and hybrid DFT calculations. A 'chain-of-spheres' algorithm for the Hartree–Fock exchange. *Chem. Phys.* **2009**, *356* (1-3), 98-109. DOI: https://doi.org/10.1016/j.chemphys.2008.10.036

(49) Izsak, R.; Neese, F. An overlap fitted chain of spheres exchange method. *J. Chem. Phys.* **2011**, *135* (14), 144105. DOI: https://doi.org/10.1063/1.3646921

(50) Pantazis, D. A.; Neese, F. All-Electron Scalar Relativistic Basis Sets for the Actinides. *J. Chem. Theory. Comput.* **2011**, *7* (3), 677-684. DOI: https://doi.org/10.1021/ct100736b

(51) Weigend, F.; Ahlrichs, R. Balanced basis sets of split valence, triple zeta valence and quadruple zeta valence quality for H to Rn: Design and assessment of accuracy. *Phys. Chem. Chem. Phys.* **2005**, *7* (18), 3297-3305. DOI: https://doi.org/10.1039/B508541A

(52) Weigend, F. Accurate Coulomb-fitting basis sets for H to Rn. *Phys. Chem. Chem. Phys.* **2006**, *8* (9), 1057-1065. DOI: https://doi.org/10.1039/B515623H

(53) Pantazis, D. A.; Chen, X. Y.; Landis, C. R.; Neese, F. All-Electron Scalar Relativistic Basis Sets for Third-Row Transition Metal Atoms. *J. Chem. Theory. Comput.* **2008**, *4* (6), 908-919. DOI: https://doi.org/10.1021/ct800047t

(54) Rolfes, J. D.; Neese, F.; Pantazis, D. A. All-electron scalar relativistic basis sets for the elements Rb-Xe. *J. Comput. Chem.* **2020**, *41* (20), 1842-1849. DOI: https://doi.org/10.1002/jcc.26355

(55) Pantazis, D. A.; Neese, F. All-Electron Scalar Relativistic Basis Sets for the Lanthanides. *J. Chem. Theory. Comput.* **2009**, *5* (9), 2229-2238. DOI: https://doi.org/10.1021/ct900090f

(56) Rajabi, A.; Grotjahn, R.; Rappoport, D.; Furche, F. A DFT perspective on organometallic lanthanide chemistry. *Dalton Trans.* **2024**, *53* (2), 410-417. DOI: https://doi.org/10.1039/D3DT03221C

(57) *AIMAll (Version 19.10.12)*; Todd A. Keith, TK Gristmill Software, Overland Park KS, USA: 2019 (aim.tkgristmill.com).

(58) Glendening, E. D.; Landis, C. R.; Weinhold, F. NBO 7.0: New vistas in localized and delocalized chemical bonding theory. *J. Comput. Chem.* **2019**, *40* (25), 2234-2241. DOI: https://doi.org/10.1002/jcc.25873
